# Fentanyl-Type Antagonist of the μ-Opioid Receptor: Important Role of Axial Chirality in the Active Conformation

**DOI:** 10.1021/acs.jmedchem.4c00935

**Published:** 2024-06-13

**Authors:** Hironobu Arita, Ryoko Tanaka, Shuntaro Kikukawa, Tsukasa Tomizawa, Haruka Sakata, Masahiko Funada, Kenichi Tomiyama, Masaru Hashimoto, Tomohiko Tasaka, Hidetsugu Tabata, Kayo Nakamura, Kosho Makino, Tetsuta Oshitari, Hideaki Natsugari, Hideyo Takahashi

**Affiliations:** † Faculty of Pharmaceutical Sciences, Tokyo University of Science, Noda-shi, Chiba 278-8510, Japan; ‡ Faculty of Pharmaceutical Sciences, Shonan University of Medical Sciences, Yokohama-shi, Kanagawa 224-0806, Japan; § Section of Addictive Drug Research, Department of Drug Dependence Research, National Institute of Mental Health, National Center of Neurology and Psychiatry, Kodaira-shi, Tokyo 187-8533, Japan; ∥ Faculty of Agriculture and Life Science, Hirosaki University, Hirosaki-shi, Aomori 036-8561, Japan; ⊥ Affinity Science Corporation, Shinagawa-ku, Tokyo 141-0031, Japan; # Faculty of Pharma Sciences, Teikyo University, Itabashi-ku, Tokyo 173-8605, Japan; ¶ Research Institute of Pharmaceutical Sciences, Musashino University, Nishitokyo-shi, Tokyo 202-8585, Japan; ∇ Graduate School of Pharmaceutical Science, The University of Tokyo, Bunkyo-ku, Tokyo 113-0033, Japan

## Abstract

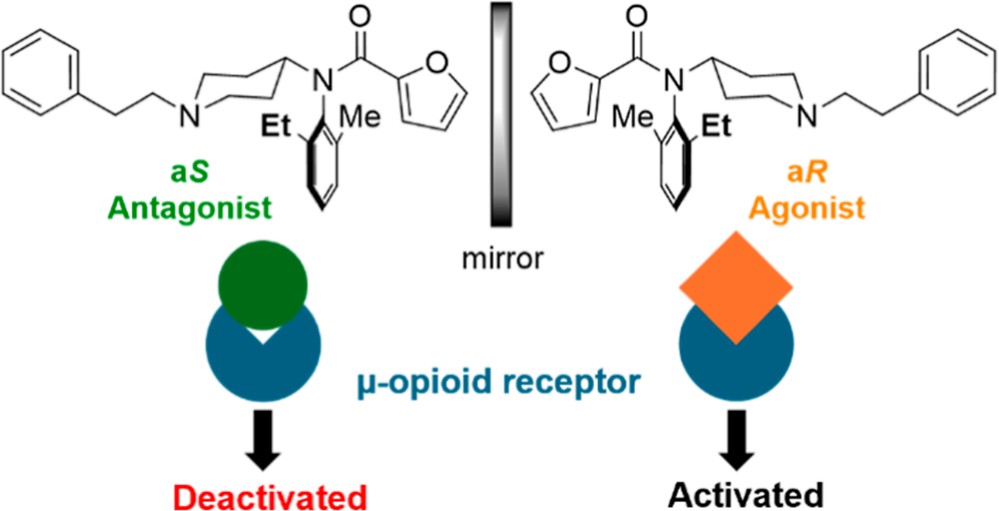

In recent years, synthetic opioids have emerged as a predominant cause of drug-overdose-related fatalities, causing the “opioid crisis.” To design safer therapeutic agents, we accidentally discovered μ-opioid receptor (MOR) antagonists based on fentanyl with a relatively uncomplicated chemical composition that potentiates structural modifications. Here, we showed the development of novel atropisomeric fentanyl analogues that exhibit more potent antagonistic activity against MOR than naloxone, a morphinan MOR antagonist. Derivatives displaying stable axial chirality were synthesized based on the amide structure of fentanyl. The a*S*- and a*R*-enantiomers exerted antagonistic and agonistic effects on the MOR, respectively, and each atropisomer interacted with the MOR by assuming a distinct binding mode through molecular docking. These findings suggest that introducing atropisomerism into fentanyl may serve as a key feature in the molecular design of future MOR antagonists to help mitigate the opioid crisis.

## Introduction

Opioid analgesics are conventionally administered in clinical practice. Apart from their medical applications, opioids are frequently misused for their euphoric effects. Substance abuse among young individuals is rapidly escalating and presenting a global cause of concern. According to the Centers for Disease Control and Prevention, over 106,000 people in the USA succumbed to drug overdose in 2021, with 75.4% of the annual overdose-related fatalities attributable to opioids.^1^ Currently, detoxification and maintenance therapy are the two most prevalent approaches used to treat opioid-use disorders.^2^ Naloxone, the μ-opioid receptor (MOR) antagonist approved by the Food and Drug Administration, has demonstrated the ability to efficaciously manage opioid misuse and overdose and mitigate relapse. However, naloxone has certain drawbacks, including a brief duration of action^3^ and weak affinity for δ- and κ-opioid receptors.^4^ To facilitate the provision of alternative therapeutics, it is necessary to develop MOR antagonists with more favorable profiles. To date, numerous antagonists possessing morphinan skeletons have been extensively investigated;^3,5−7^ however, little compound superior to naloxone has been obtained. We anticipated that further studies based on the morphinan skeleton, which has already been thoroughly investigated, would not deliver improved results. Therefore, the development of a novel framework that facilitates the assessment of the structure–activity relationships (SARs) concerning antagonistic activity against the MOR will likely prove more fruitful. 4-Anilidopiperidines, μ agonists, have a prominent place due to their high potency, low cardiovascular toxicity, fast onset, and often short duration of action.^8^ Fentanyl, discovered in 1962 by Janssen, is the prototype of the 4-anilidopiperidine class of synthetic opioid analgesics.^9^ It is a full agonist that binds with high affinity to the MOR. It is about 50–100 times more potent than morphine and is characterized by a rapid onset of analgesia and relatively short duration of action.^10,11^ However, fentanyl exhibits serious adverse effects, including respiratory depression, muscle rigidity, nausea, sedation, and, with prolonged use, tolerance and addiction.^10^ Based on the precedent SAR studies of fentanyl analogues, the potent factors for the analgesic activity are elucidated (Table 1).^12−20^

**Table 1:**
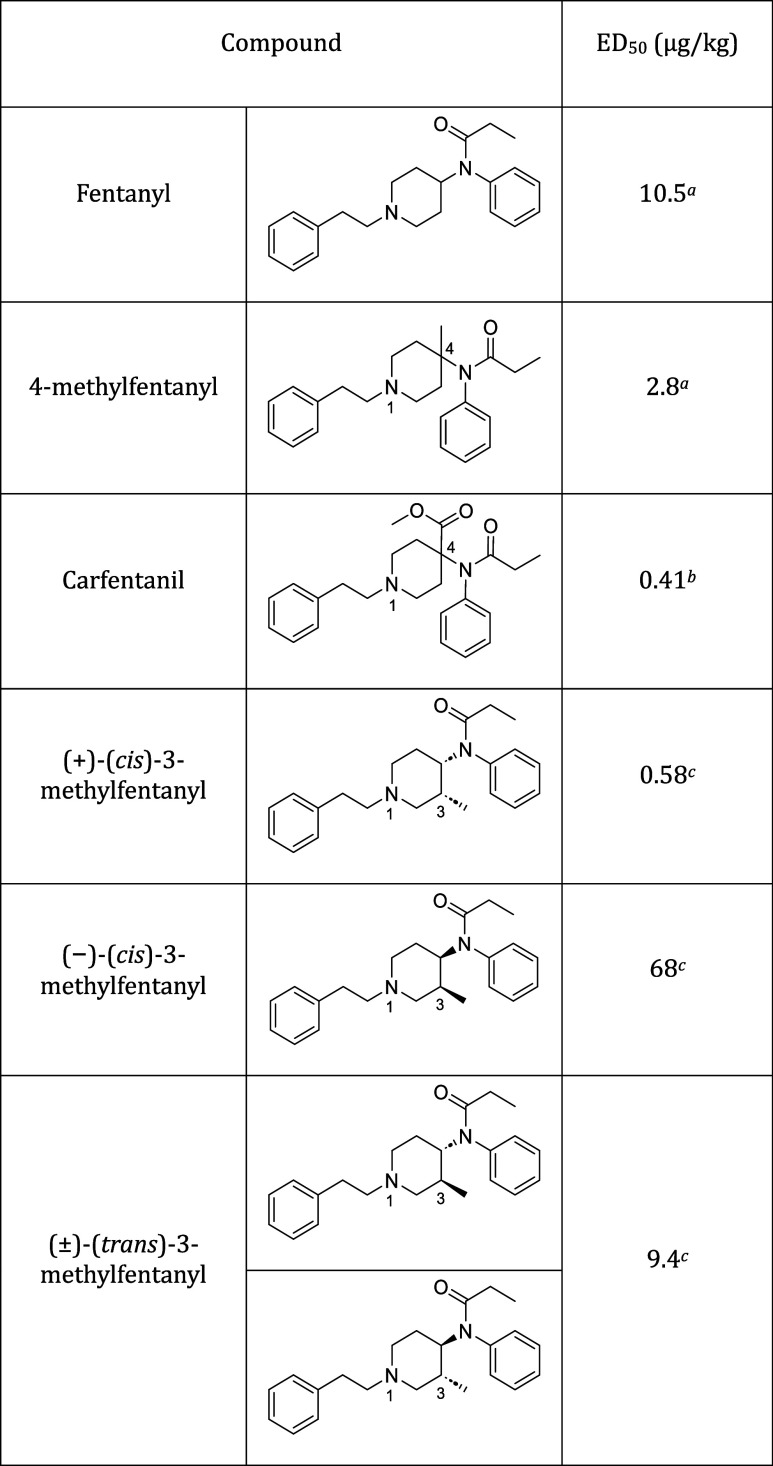
Structure and Pharmacological Activity of Fentanyl and Its Derivatives

a ED_50_ value according to ref (17).

b ED_50_ value according to ref (18).

c ED_50_ value according to ref (19).

As shown in 4-methyl fentanyl and carfentanil, substitution at the 4-position of the piperidine ring should increase the binding affinity by 3.8–30-fold in comparison to fentanyl.^17,18^ Additionally, the stereochemistry of the substituents of the piperidine ring plays a crucial role in the potency of these analogues. For example, 3-methylfentanyl, which has two chiral centers, should exist in four stereoisomers. The (+)-*cis* isomer is 16 times more active than *trans* isomers and about 100 times more active than its (−)-enantiomer.^19^

**Figure 1:**
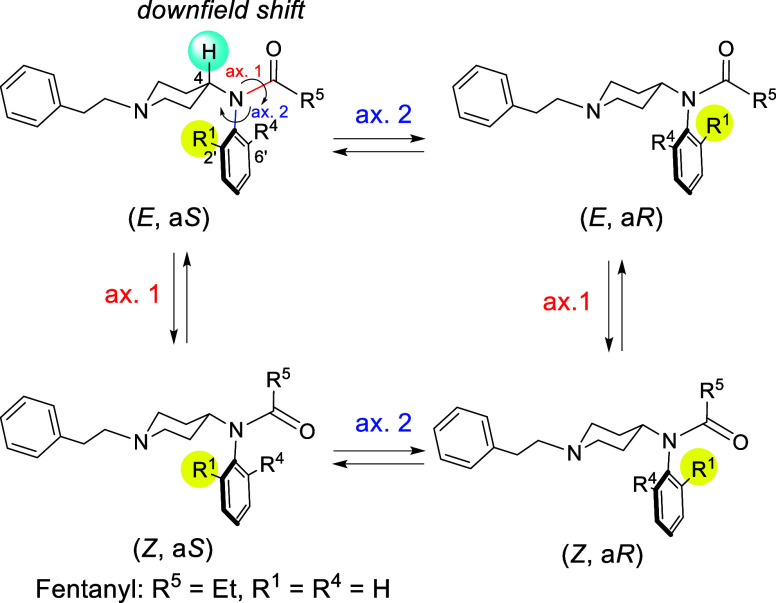
Stereoisomers of fentanyl analogues.

There is one other thing that is important for the design of potent fentanyl analogues. Recently, we have been interested in axial chirality derived from the Ar−N(CO) axis and its relationship with bioactivities.^21−24^ We presumed that fentanyl analogues should exhibit *E*/*Z*-isomerism around the N−(C=O) bond (axis 1) and atropisomerism based on the Ar−N(CO) bond (axis 2). Theoretically, they should exist in four stereoisomers as shown in Figure 1. While fentanyl was known to exist predominantly in the *E*-form, the atropisomeric property has not been investigated.^25,26^ We anticipated that the stereoelectronic effect at the 2′ or 6′-positions of the anilino phenyl moiety should restrict the rotation about N−(C=O) and Ar−N(CO) axes to provide atropisomers. We aimed at discussing the relationship between the structure and the MOR interaction in the series of fentanyl analogues substituted in the position 2′ or 6′ of an anilino phenyl ring. This study focused on the stereochemical property of fentanyl analogues to provide atropisomers for the first time. Also, SAR studies and docking studies afford a clue to the future drug design of fentanyl analogues with agonistic/antagonistic activity against MOR. These findings suggest that the introduction of atropisomerism into fentanyl may serve as a key feature in the molecular design of future MOR antagonists to help mitigate the opioid crisis.

## Results and Discussion

### Chemistry

**Scheme 1:**
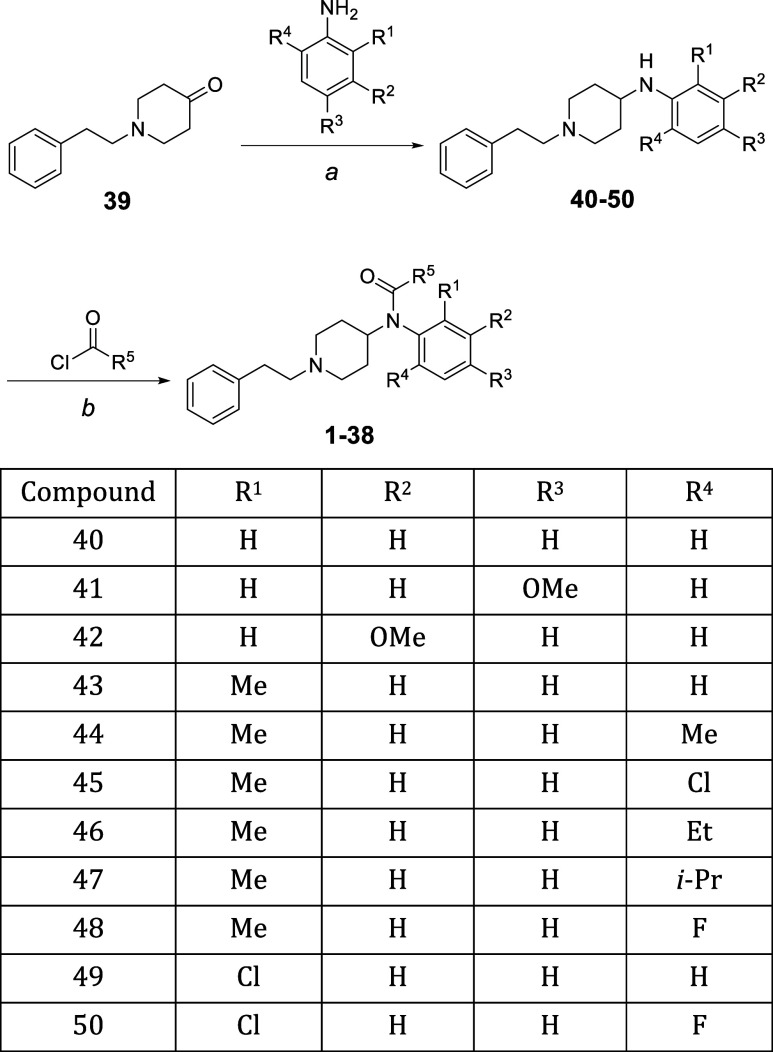
Preparation of Fentanyl Analogues Reagents and conditions: (a) AcOH, EtOH, reflux; then, NaBH_3_CN, reflux; (b) NaH, DMF, 25 °C.

Derivatives of fentanyl (**1–38**) were prepared from 1-(2-phenylethyl)-4-piperidone (**39**), as shown in Scheme 1. The reductive amination of commercially available compound **39** furnished compounds **40–50**, which were successively treated with various acid chlorides to provide fentanyl derivatives **1–38**.

First, the conformational properties of fentanyl analogues **1–38** were characterized using ^1^H NMR spectroscopy (Table 2). Methyl carbamate derivatives **3**, and **4**, *N*-alkanoyl derivatives **5–10**, **13**, **14**, **22**, **25–30**, and **36–38**, 4-substituted benzoyl derivatives **2**, **11**, **24**, and **34**, and *N*-heteroaryl derivatives **1**, **15**, **23**, **31–33**, and **35** were observed as single isomers at 25 °C irrespective of the substituents of the anilino moiety. However, *N*-2-substituted-benzoyl derivatives **12** and **16–20**, and *N*-2,6-disubstituted-benzoyl derivative **21** existed as an equilibrium mixture of *E*/*Z*-diastereomers in solution (CDCl_3_) at the ratio of 89:11–67:33. In each spectrum, the H-4 proton resonance of the piperidine ring in the major diastereomer was located at approximately 4.77–4.27 ppm, 1.22–0.53 ppm downfield from its partner, respectively.^27^ This downfield shift was ascribed to the anisotropic effect of the carbonyl of the acyl group (Figure 1). Therefore, we presumed that compounds **12** and **16–21** exist in *E*-amide in preference to *Z*-amide. Meanwhile, the H-4 proton resonance of the piperidine ring in single isomers **1–11**, **13–15**, and **22–38** was located at 4.79–4.16 ppm, corresponding to *E*-amide in **12** and **16–21**. Additionally, considering that fentanyl exists in the *E*-form in solution,^25,26^ the conformations of **1–11**, **13–15**, and **22–38** observed as single isomers were presumed to be *E*-amide.

**Table 2:**
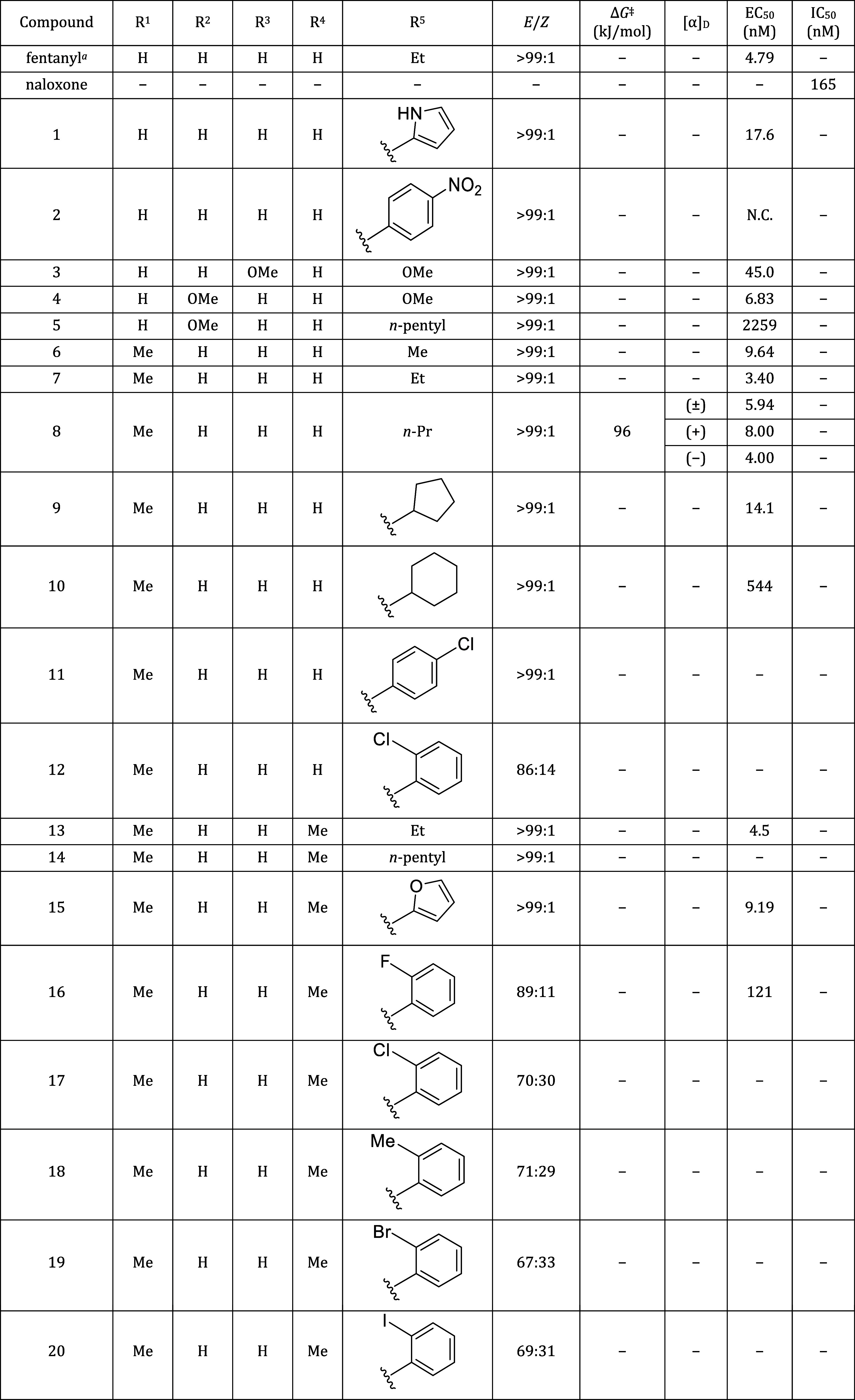

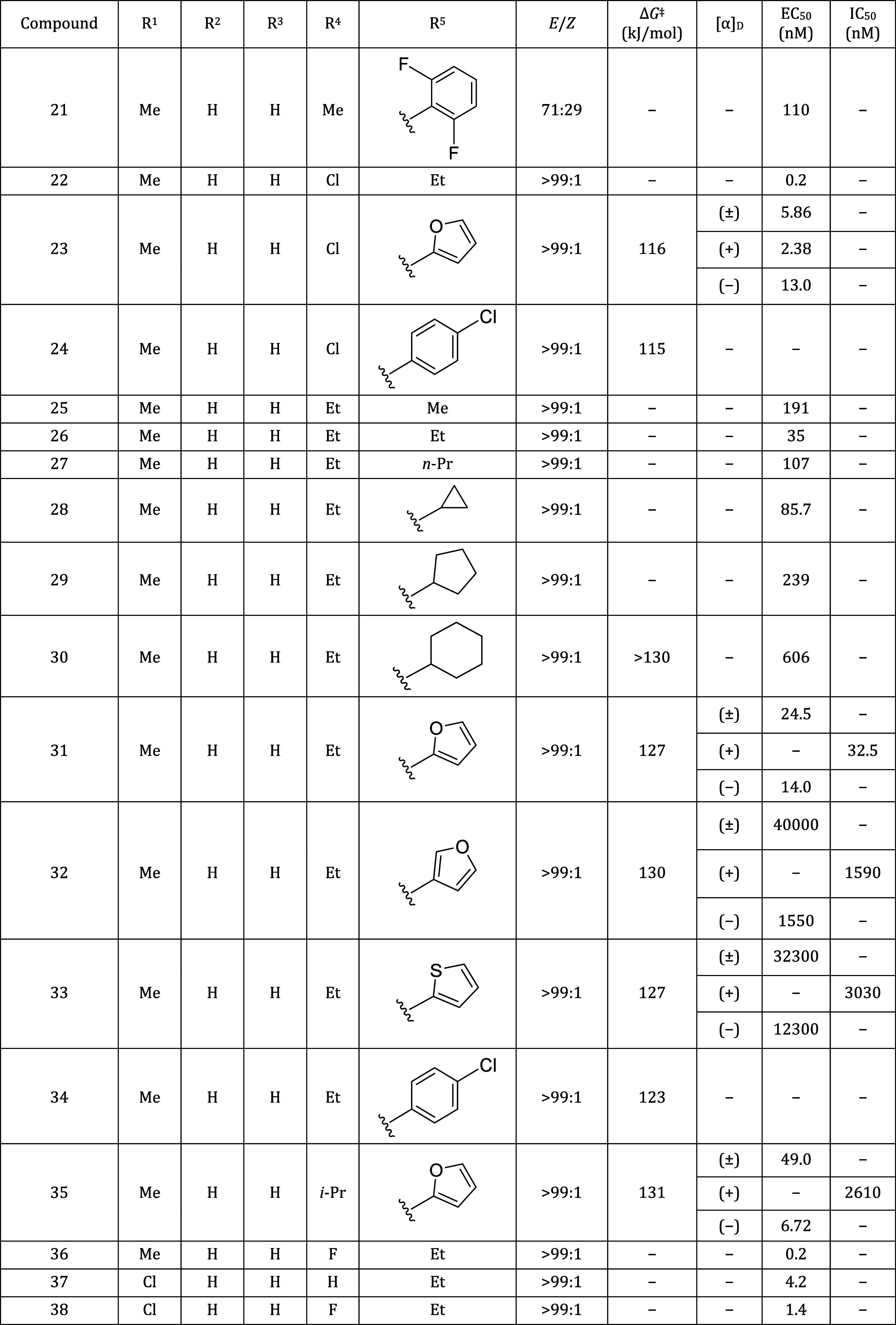
Physicochemical Properties of Fentanyl Derivatives and Their Activities against the MOR

a The NMR data of fentanyl adopted from ref (26).

**Figure 2:**
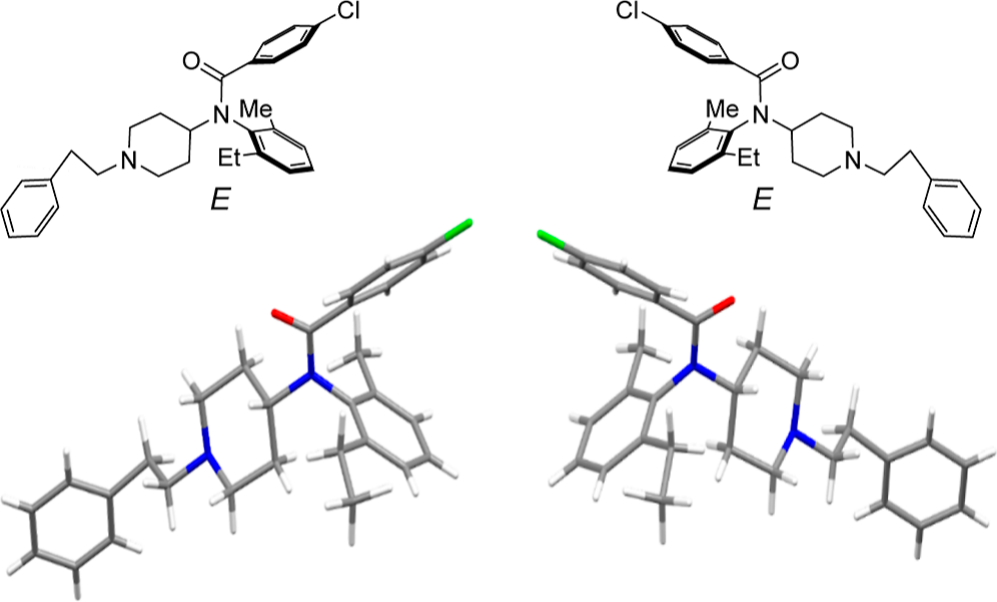
X-ray crystallographic structure of compound **34**.

To determine the *E*/*Z*-amide stereochemistry, an investigation using NOESY spectroscopy was conducted. A correlation between the 6-H of the benzoyl group and 2′-CH_3_ of the anilino moiety was observed in the major peak of **17** (see Supporting Information Figures S1 and S2). Therefore, the preference for *E*-amide was determined. Fortunately, a single crystal for the X-ray crystal structure analysis of compound **34** was obtained, in which **34** possessed the *E*-form (Figure 2 and see the Supporting Information). Based on these results, the preference for *E*-amide observed in compounds **1–38** was confirmed. Bulky substituents at the 2-position of the *N*-benzoyl group increased the ratio of *Z*-isomers (Table 2). Unfortunately, the separation of *E*/*Z*-amide in compounds **12** and **16–21** using nonchiral HPLC failed because the rotational barrier of the N–(C=O) bond (axis 1) was less than that required for the isolation.

As shown in Figure 1, four stereoisomers [*i.e.*, *E*/*Z*-amide rotamers around N–(C=O) bond (axis 1) and (a*S*)/(a*R*)-axial isomers based on the Ar−N(CO) bond (axis 2)] were assumed to exist. Although the rotation of axis 1 was too rapid to isolate *E*/*Z*-amide, the presence of atropisomers derived from axis 2 was confirmed by isolating the enantiomers of compounds **8**, **23**, **24**, and **30–35***via* chiral HPLC. The separated atropisomers have opposite [α]_D_ values from each other. After obtaining the atropisomerically pure isomers, the activation-free energy barrier to rotation (Δ*G*^⧧^) was estimated (Table 2) (see Supporting Information Figures S3–S11). The Δ*G*^⧧^ value of 96 kJ/mol at 37 °C for compound **8**, which was stable enough to be isolated, was assumed to be due to the lower bulkiness of the 2′-Me substitute of the anilino moiety. Considering this, it is plausible that the atropisomers of compound **11** with 2′-Me monosubstituted were not isolated *via* chiral HPLC. Both 2′ and 6′ substitutions in the anilino moiety were necessary to freeze the rotation of the Ar−N(CO) bond (axis 2). The enantiomers of compound **34** at 80 °C with a Δ*G*^⧧^ value of 123 kJ/mol were more stable than those of compound **24** (Δ*G*^⧧^ = 115 kJ/mol at 80 °C). Similarly, the enantiomers of compound **35** with a Δ*G*^⧧^ value of 131 kJ/mol were more stable than those of compound **23** (Δ*G*^⧧^ = 116 kJ/mol at 80 °C) and **31** (Δ*G*^⧧^ = 127 kJ/mol at 80 °C). The increased energy barrier was ascribed to the bulky substituents in the anilino moiety (Cl < Et < *i*-Pr).

The heteroaryl-substituted series were also investigated. We changed the 2-furoyl of compound **31** to the regioisomer 3-furoyl of compound **32** and the isosteric five-membered heteroaromatic ring 2-thenoyl of compound **33**. The enantiomers of **32** and **33** (Δ*G*^⧧^ = 130 and 127 kJ/mol at 100 °C, respectively) were equally as stable as those of compound **31**. Meanwhile, the higher stability with a Δ*G*^⧧^ value of approximately >130 kJ/mol was estimated in *N*-cyclohexanoyl **30** because no racemization was observed at 80 °C for 54 h and decomposition at 100 °C was observed. Considering that the atropisomers of *N*-butanoyl compound **8** were isolatable with a Δ*G*^⧧^ value of 96 kJ/mol, the *N*-alkanoyl-substituted compounds are in more sterically restricted form than the *N*-aroyl-substituted compounds.

### SAR Targeting the μ-Opioid Receptor

Fentanyl and its derivatives **1–10**, **13**, **15**, **16**, **21–23**, **25–33**, and **35–38** were evaluated as MOR agonists using opioid-μ-receptor-expressing CHO cells (Table 2, see Supporting Information Figures S12–S15). Fentanyl was used as a control compound. In this case, those with axial chirality were evaluated as racemates. First, the EC_50_s of the *N*-alkanoyl- and *N*-aroyl-substituted series were compared. Among compounds **6–10**, the activity was the highest in *N*-propionyl compound **7** (EC_50_: 3.40 nM), which is comparable to fentanyl. With the increasing bulkiness of the *N*-alkanoyl group, the potency decreases considerably. Thus, **8** (EC_50_: 5.94 nM), **9** (EC_50_: 14.1 nM), and **10** (EC_50_: 544 nM) were about 1.7, 4.1, and 160 times less potent than **7**, respectively. A similar trend was observed in compounds **26–30**. In particular, the marked decrease in the activity of compounds **10** (EC_50_: 544 nM) and **30** (EC_50_: 606 nM) indicated that the size of cyclohexane significantly impairs binding to the receptor.

As to *N*-aroyl-substituted compounds, compound **2** with a 4-nitro substituent at the *N*-benzoyl group was inactive since the steric and electronic effects of the 4-nitro group at the benzoyl site appear to be a fatal obstacle for the MOR receptor. Considering that compound **16** with a *N*-2-fluorobenzoyl group (EC_50_: 121 nM) and compound **21** with a *N*-2,6-difluorobenzoyl group (EC_50_: 110 nM) showed low activity, *N*-benzoyl derivatives seemed unpromising. Therefore, we focused on five-membered heterocycles. While the activity of *N*-2-pyranoyl-substituted **1** (EC_50_: 17.6 nM) and *N*-2-furoyl-substituted **15** (EC_50_: 9.19 nM) is more potent than that of *N*-benzoyl derivatives, 2-thenoyl compound **33** showed a marked decrease in potency (EC_50_: 32,300 nM). Comparing the substitution position of the furanoyl group, a large difference in the potency of 2-furoyl **31** (EC_50_: 24.5 nM) and 3-furoyl **32** (EC_50_: 40,000 nM) indicates the importance of the position of oxygen on the furan ring. Comparing the *N*-propionyl-substituted compounds **7** (EC_50_: 3.40 nM) and **37** (EC_50_: 4.2 nM) revealed that the 2′-methyl or 2′-chloro monosubstitute of the anilino moiety produced agonistic activity equal to that of fentanyl. Among the *N*-propionyl-substituted compounds, in which both *ortho*-positions of the anilino moiety were substituted, **22** with 2′-chloro,6′-methyl (EC_50_: 0.2 nM), **36** with 2′-fluoro,6′-methyl (EC_50_: 0.2 nM), **38** with 2′-fluoro,6′-chloro (EC_50_: 1.4 nM) showed excellent agonistic activity. In contrast, a decreasing trend in potency was observed in **13** with 2′,6′-dimethyl (EC_50_: 4.5 nM) and **26** with 2′-ethyl,6′-methyl (EC_50_: 35 nM). Based on this, a halogen substituent at either *ortho*-position enhanced the agonistic activity. Comparing the *N*-2-furoyl-substituted compounds **15** with 2′,6′-dimethyl (EC_50_: 9.19 nM), **23** with 2′-chloro,6′-methyl (EC_50_: 5.86 nM), **31** with 2′-ethyl,6′-methyl (EC_50_: 24.5 nM), and **35** with 2′-isopropyl,6′-methyl (EC_50_: 49.0 nM), increasing the bulkiness at the *ortho*-position of the anilino moiety (Me < Et < *i*-Pr) decreases potency.

Next, the separated enantiomers of **8**, **23**, **31–33**, and **35** were subjected to *in vitro* assays to examine the difference in potency between the enantiomers (Table 2, see Supporting Information Figures S12–S15). The separated enantiomer (−)-**31** exhibited a slight increase in agonistic activity (EC_50_: 14.0 nM) than its racemate (EC_50_: 24.5 nM), whereas (+)-**31** showed no agonistic activity (Table 2). Therefore, we examined the antagonist activity of (+)-**31** against the MOR and observed antagonist activity with an IC_50_ value of 32.5 nM, 5-fold more potent than naloxone (IC_50_: 165 nM), which is a control compound as an antagonist for MOR (Table 2, see Supporting Information Figure S16). The distinct way each enantiomer of compound **31** interacted with the target molecule was unusual. The *in vitro* binding affinities of compound **31** enantiomers to the MOR were examined *via* a competitive radioligand binding assay to determine whether the agonistic or antagonistic activity could be attributed to the binding of the agonist and antagonist, respectively, to the orthosteric site of the MOR (see Supporting Information Table S1 and Figure S17). Both (+)-**31** and (−)-**31** exhibited remarkable affinity in the binding experiment at the nanomolar level for the MOR (*K*_i_ = 1.86 and 4.96 nM, respectively). They were more potent than [d-Ala2,NMe-Phe4-Gly5-ol]-enkephalin (DAMGO), which is a full agonist at MOR (*K*_i_ = 16.5 nM). We confirmed that the agonist/antagonist potency of each atropisomer of **31** was due to the binding affinity for the orthosteric site of the MOR. Therefore, we readdressed the calcium flux assay conducted using MOR-CHO cells (Table 2). The bioactivity of **35** was investigated in which an isopropyl group, instead of an ethyl group, was substituted at the 6′-position of the anilino moiety. (+)-**35** did not exhibit agonist activity as strongly as (+)-**31**. Therefore, we examined the MOR antagonist activity of (+)-**35**, which exhibited low antagonistic activity (IC_50_: 2610 nM). We postulated that substitution with the 6′-isopropyl group, which is bulkier than the ethyl group in (+)-**31**, might block the binding site, decreasing the antagonist’s activity. In contrast to the antagonist effect, (−)-**35** exhibited more potent agonist activity (EC_50_: 6.72 nM) than (−)-**31** (EC_50_: 14.0 nM). Fentanyl derivatives differ in their binding mode to MOR depending on their steric structure, resulting in exactly the opposite activity. Interestingly, compound **23**, in which a chloro atom, instead of an ethyl group, was substituted at the 6′-position, showed agonist activity in the racemate form, with both atropisomers exhibiting agonistic activity. The electronic properties of the 6′-chloro substituent might have altered the binding configuration of **23** with the MOR, so the antagonistic activity was lost. In addition, in compound **8**, racemic and enantiomeric forms showed comparable agonist activity because atropisomers with 2′-methyl monosubstitution were unstable (Δ*G*^⧧^ = 96 kJ/mol).

Changes in the *N*-acyl substitution were also investigated. Maintaining the two *ortho*-position substitutions with ethyl and methyl groups, the 3-furoylated compound **32** and 2-thenoylated compound **33** were examined. While the (−)-atropisomers of these compounds showed weak MOR agonistic activity [(−)-**32**: EC_50_ = 1550 nM, (−)-**33**: EC_50_ = 12,300 nM], the (+)-enantiomers of **32** and **33** exhibited antagonistic activity, albeit at a low level [(+)-**32**: IC_50_ = 1590 nM, (+)-**33**: IC_50_ = 3030 nM]. Although the activity of compounds (+)-**31**, (+)-**32**, (+)-**33**, (+)-**35** is weak, it is apparent that no agonistic activity was observed (Figures S14 and S15). These results revealed that the *N*-2-furoyl and alkyl substitutions at the 2′ and 6′ positions of the anilino moiety are important for eliciting antagonistic activity. The methyl and ethyl groups at the *ortho*-positions on the anilino moiety are the optimal combination to achieve MOR antagonistic activity. Intriguingly, the MOR distinguishes the difference of only one carbon between the methyl and ethyl groups at the *ortho*-position in each atropisomer and exhibits different responses (agonistic/antagonistic) accordingly.

**Figure 3:**
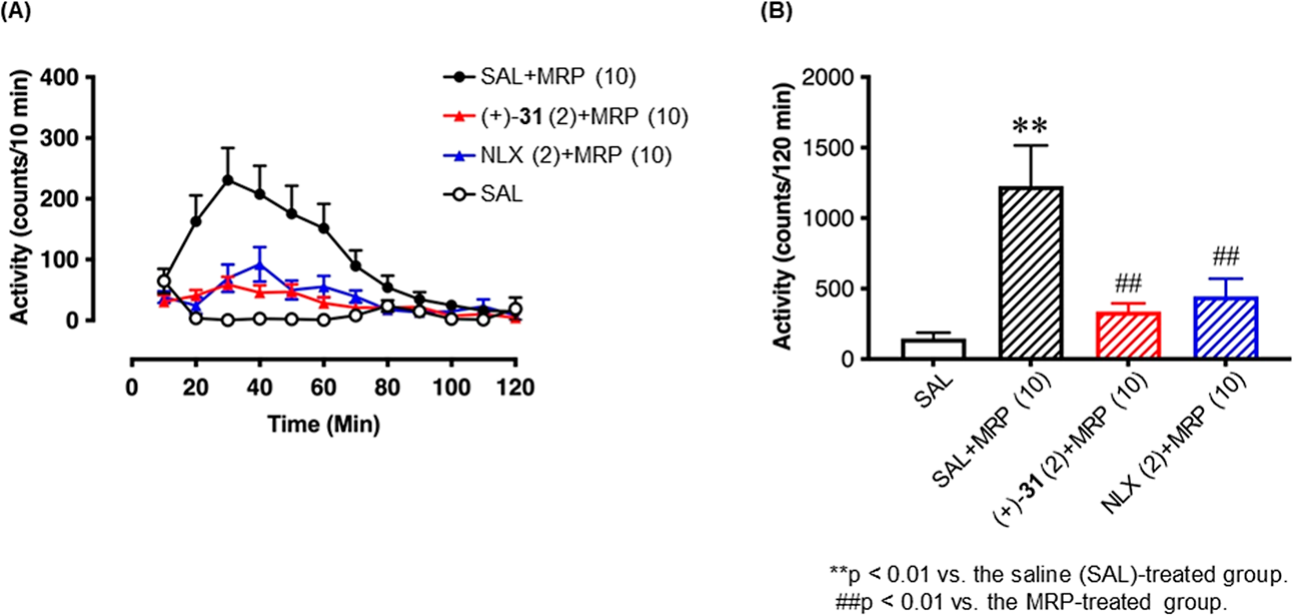
Ethopharmacological analyses of (+)-**31** and naloxone (NLX). (A) Time-dependent alterations after acute administration of morphine (MRP; 10 mg/kg; i.p.) and pretreatment with the opioid receptor antagonist NLX (2 mg/kg; i.p.; pre 30 min) or (+)-**31** (2 mg/kg, i.p.; pre 30 min) in MRP-induced hyperlocomotion in mice. Each point represents the mean activity counts with standard error of the mean (SEM) for 10 min (*n* = 12). (B) Each column represents the mean total locomotor activity counts with the SEM for 120 min (*n* = 12). Dunnett’s post-test was also applied to each graph.

Murine models were used for the subsequent ethopharmacological analysis of (+)-**31** and naloxone. A prior *in vitro* assay established (+)-**31** as a potent MOR antagonist. Morphine injection triggered spontaneous locomotor activity in mice that peaked at 30 min, and the intraperitoneal administration of (+)-**31** antagonized the effect of morphine for 120 min as with naloxone (Figure 3A). This antagonistic effect of (+)-**31** was comparable to that of naloxone (Figure 3B).

### Determination of Absolute Configuration

**Figure 4:**
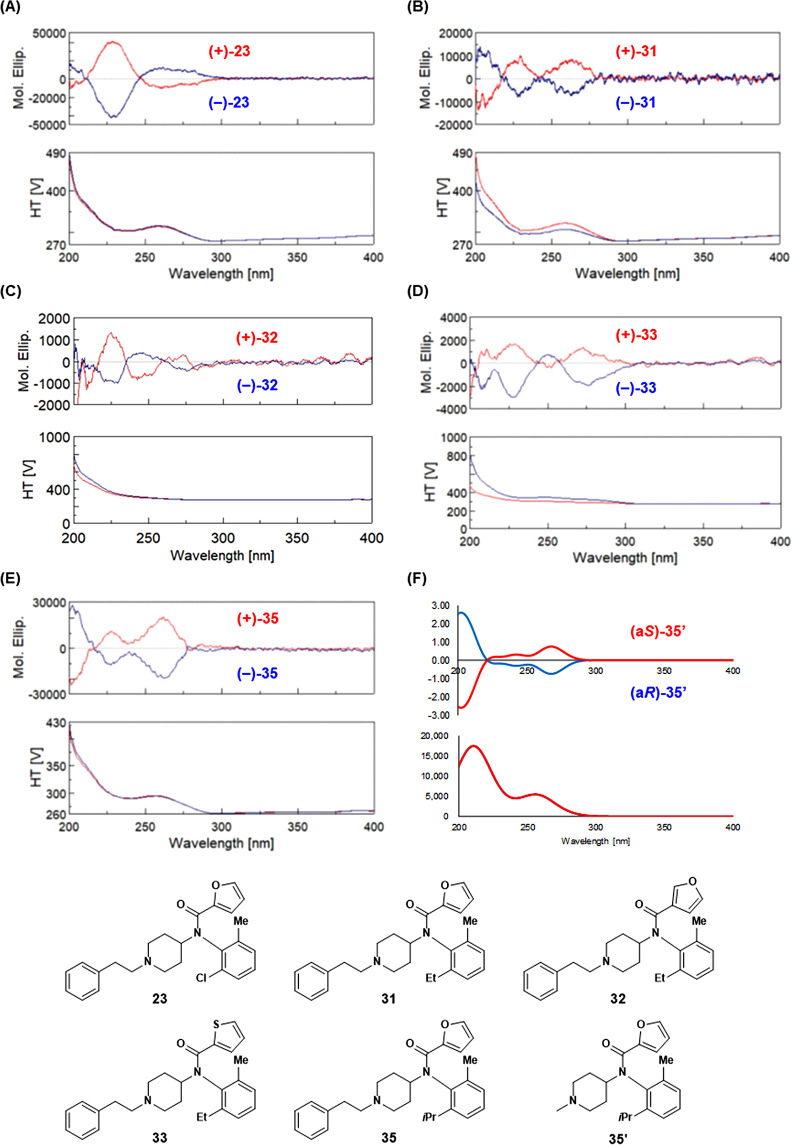
Comparison of calculated and experimental ECD. The calculated ECD values of the a*R* and a*S* isomers are similar to the experimental ECD values of the (−)- and (+)-enantiomers, respectively. (A) Experimental ECD of **23**: (+)-**23** (red) and (−)-**23** (blue). (B) Experimental ECD of **31**: (+)-**31** (red) and (−)-**31** (blue). (C) Experimental ECD of **32**: (+)-**32** (red) and (−)-**32** (blue). (D) Experimental ECD of **33**: (+)-**33** (red) and (−)-**33** (blue). (E) Experimental ECD of **35**: (+)-**35** (red) and (−)-**35** (blue). (F) Calculated TD-DFT-based ECD of **35**′: a*S*-**35**′ (red) and a*R*-**35**′ (blue).

Although optically resolved, **23**, **31–33**, and **35** did not provide single crystals suitable for X-ray diffraction analysis; (+)-**31** exhibited characteristic positive Cotton effects at 230 and 265 nm with similar intensities. Compounds (+)-**32**, (+)-**33**, and (+)-**35** showed similar electronic circular dichroism (ECD) spectral profiles (Figure 4), suggesting that the chirality at the Ar−N(CO) bond (axis 2) correlates with these Cotton effects. Considering the computation costs, we performed the ECD spectral calculations using time-dependent density-functional theory (DFT).^28^ These spectral calculations were performed on the simplified model compound **35**′, in which the phenylethyl group of **35** was replaced with a methyl group. The CAM-B3LYP/def2-TZVP level of theory^29^ was selected based on our previous data.^30,31^ The ECD spectra were constructed considering the Boltzmann distributions calculated using the free energies based on the same calculation conditions as above. The DFT calculations of the a*R* enantiomer of **35**′ (Figure 4F) reproduced the experimental ECD spectra of **35** well (see Supporting Information Tables S2–S5). Therefore, the absolute configurations of (+)-**35**/(−)-**35** were determined to be a*S*/a*R*. Accordingly, (+)-**31**/(−)-**31** were also determined to be a*S*/a*R*. Similarly, we determined the absolute configurations of the (+)- and (−)-enantiomers of **32** and **33** and concluded that the enantiomers displaying MOR antagonistic activities were in the a*S* form.

### Docking Studies and Molecular Dynamics Simulations

**Figure 5:**
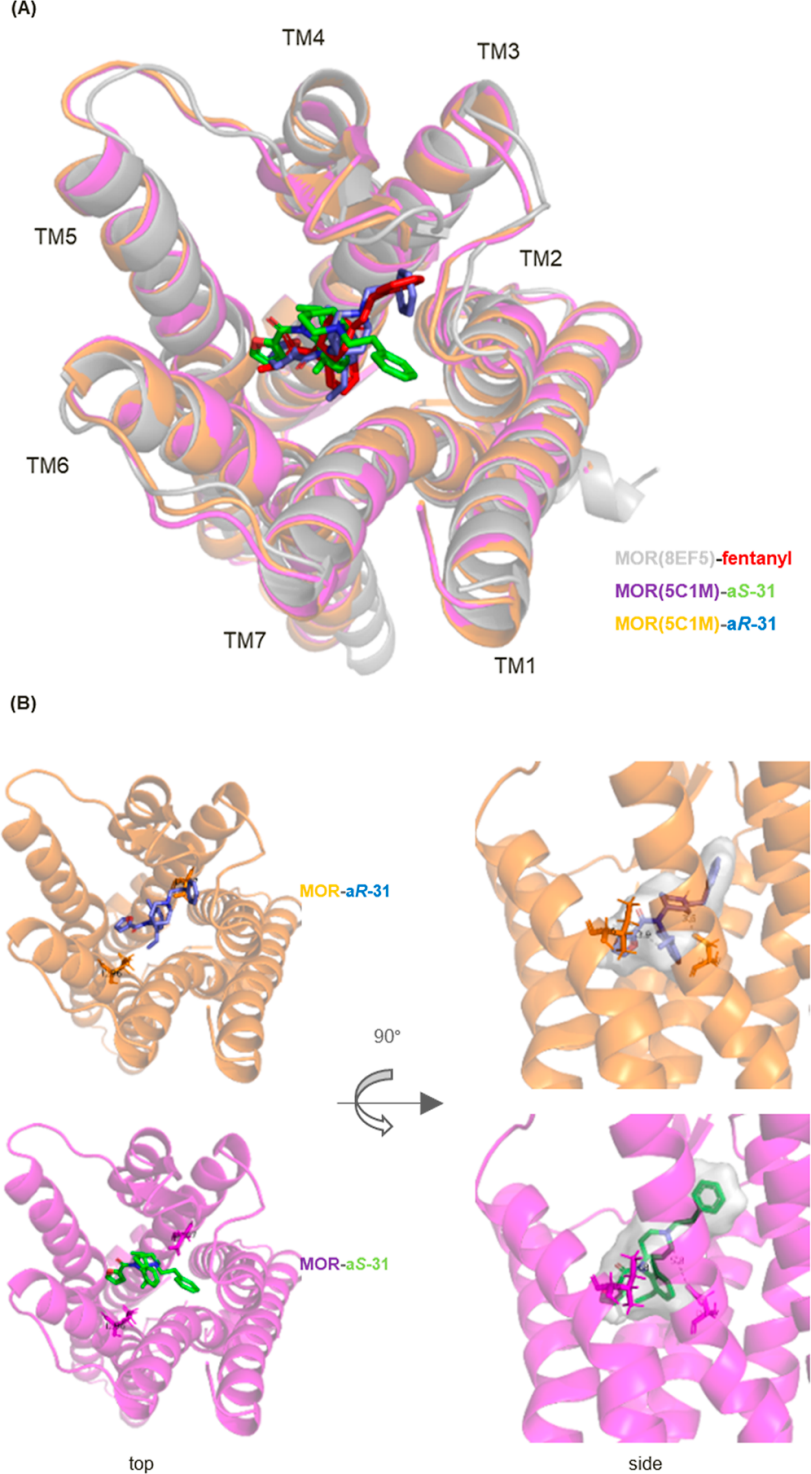
Superposition of the predicted a*R*-**31** (blue) and a*S*-**31** (green) binding configurations in the MOR (PDB: 5C1M) and that of fentanyl (red) in the MOR (PDB: 8EF5).

To obtain structural insights into the enantiomers of a*R*/a*S*-**31** and MOR interactions, we conducted molecular docking studies and molecular dynamics (MD) simulations of the complexes of each of the **31** enantiomers and the MOR (Protein Data Bank [PDB]: 5C1M).^32^ Recently, Zhuang *et al.* obtained a cocrystal with fentanyl and MOR (PDB: 8EF5)^33^ and reported that fentanyl binds to the same orthosteric site of MOR as morphine does. Therefore, each a*R*-**31** and a*S*-**31** was added to replace fentanyl at the same orthosteric site. The predicted binding positions of the **31** enantiomers and the reported binding conformation of fentanyl were superimposed, and the three binding positions were compared (Figure 5A, see Supporting Information Figures S18 and S19 and Tables S6 and S7). These enantiomers existed as *E*-conformers similar to fentanyl; however, each enantiomer interacted with the MOR amino acid residues in a distinct manner. The phenylethyl chain of the agonist a*R*-**31** was located between transmembrane (TM)2 and TM3, consistent with the binding position of fentanyl. This confirmed that the interaction of a*R*-**31** with the MOR active site was similar to that of fentanyl.

Conversely, a*S*-**31** also bound to the orthosteric site; nonetheless, the phenylethyl group extended in a different direction, between TM2 and TM7, toward TM1. Considering that the distinction between the two *ortho*-substituents (one carbon difference between the methyl and ethyl groups) causing axial chirality in compound **31** is minute, it is intriguing that the orientation of the phenylethyl group that interacts with MOR differs markedly. To identify the occurrence of such striking differences in the MOR interactions, we investigated the extent to which each enantiomer interacts with the amino acid residues of the MOR *via* MD pharmacophore analysis. Like fentanyl, both enantiomers exhibited strong ionic interactions with Asp147. In the case of a*R*, an *ortho*-methyl group interacted with Tyr148 (100%) and Met151 (95%), and an *ortho*-ethyl group interacted hydrophobically with Ile296 (100%), Ile322 (99%), and Trp293 (99%). However, in the case of a*S*, the *ortho*-methyl group interacted hydrophobically with Ile296 (90%) and Ile322 (94%), and the *ortho*-ethyl group interacted with Tyr148 (96%), Val236 (92%), and Met151 (86%). On comparing the interactions between the ethyl groups in each enantiomer and the amino acid residues of the MOR, Trp293 (99%) of a*R* and Val236 (92%) of a*S* did not interact with the methyl group of the paired enantiomer. This implies that the different interactions with these amino acid residues are key for mediating the agonistic and antagonistic effects.

## Conclusions

We successfully created atropisomers of fentanyl analogues by introducing substituents at the 2′ or 6′ position of the anilino moiety and separated the enantiomeric forms. Examination of the affinity at the MOR revealed that one of the atropisomers, the a*R*-form, of the fentanyl analogues serves as an agonist and the other, the a*S*-form, as an MOR antagonist. Notably, the atropisomers differ by a single carbon (methyl and ethyl). Docking studies of **31** also revealed that the binding poses of the a*R* and a*S* forms occupied the orthosteric sites. Each atropisomer assumes a distinct conformation and interaction pattern owing to its unique chemical structure. This pioneering discovery was achieved by introducing atropisomerism into the fentanyl framework. Although atropisomeric properties are often overlooked, many drugs exhibit latent chirality. Hence, collectively, this study provides a novel perspective and valuable insights for developing pharmaceuticals.

## Experimental Section

### General Remarks

Experimental materials were procured from commercial suppliers. The nuclear magnetic resonance (NMR) spectra were recorded on a spectrometer (JEOL Ltd., Tokyo, Japan, and Bruker, Billerica, MA, USA) operating at 600 MHz for ^1^H NMR and 150 MHz for ^13^C NMR. Chemical shifts are expressed in ppm relative to tetramethylsilane as an internal standard and coupling constants (*J*) are reported in hertz. The abbreviated representations for the splitting patterns are as follows: singlet (s), doublet (d), triplet (t), quartet (q), quintet (quin), multiplet (m), and broad (br). Infrared (IR) spectra were acquired using a Fourier-transform IR spectrometer equipped with an attenuated total reflectance accessory (ATR; diamond). The high-resolution mass spectra were obtained using the electrospray ionization mode. Melting points were recorded on a melting point apparatus and retained as is. Optical rotations were determined using a digital polarimeter. Analytical thin-layer and column chromatography was performed on precoated, glass-backed silica gel plates (Merck silica gel 60 F_254_: Merck KGaA, Darmstadt, Germany) and silica gel (60 μm), respectively. Extracted solutions were dried over anhydrous Na_2_SO_4_. Solvents were evaporated under reduced pressure. All compounds are >95% pure by HPLC.

#### *N*-Phenyl-1-(2-phenylethyl)-4-piperidinamine (**40**)^34^

Compound **40** was prepared according to a modified literature procedure.^34^ Acetic acid (686 μL, 12 mmol) and aniline (1.82 mL, 20 mmol) were added to a stirred solution of 1-(2-phenylethyl)-4-piperidone (2.03 g, 10 mmol) in ethanol (50 mL). The mixture was stirred under reflux for 2.5 h and cooled to 25 °C, followed by the careful, slow addition of sodium cyanoborohydride (1.26 g, 20 mmol). After stirring under reflux for 5 h, the mixture was treated with 2 N NaOH and concentrated. The concentrate was extracted with ethyl acetate. The extract was washed with 2 N NaOH and brine, dried over Na_2_SO_4_, and concentrated. The crude product was purified *via* column chromatography (methanol/dichloromethane = 1:15) to produce **40** as a white solid; yield: 1.81 g (6.45 mmol, 65%). Spectral data were consistent with the results reported in the literature.^34^

Compounds **41–50** were prepared from the corresponding 1-(2-phenylethyl)-4-piperidone (**39**) according to a procedure similar to that for preparing **40** from **39**.

#### *N*-(4-Methoxyphenyl)-1-(2-phenylethyl)-4-piperidinamine (**41**)

White solid; yield: 275.9 mg (89%). MP: 97 °C. IR (ATR): 3384 cm^–1^ (NH). ^1^H NMR (600 MHz, CDCl_3_): δ 7.30–7.27 (m, 2H), 7.22–7.18 (m, 3H), 6.77 (dt, *J* = 9.0, 3.0 Hz, 2H), 6.58 (dt, *J* = 9.0, 3.0 Hz, 2H), 3.74 (s, 3H), 3.23 (tt, *J* = 10.2, 3.6 Hz, 1H), 2.98–2.96 (m, 2H), 2.83–2.80 (m, 2H), 2.63–2.60 (m, 2H), 2.19 (t, *J* = 10.2 Hz, 2H), 2.08–2.06 (m, 2H), 1.48 (ddd, *J* = 24.0, 10.2, 3.6 Hz, 2H). An NH peak was not observed. ^13^C NMR (150 MHz, CDCl_3_): δ 152.1, 141.2, 140.3, 128.6, 128.4, 126.0, 115.0, 114.9, 60.6, 55.8, 52.5, 51.0, 50.7, 33.8, 32.6, 30.9. HRMS (ESI): *m*/*z* [M + H]^+^ calcd for C_20_H_27_N_2_O, 311.2118; found, 311.2118.

#### *N*-(3-Methoxyphenyl)-1-(2-phenylethyl)-4-piperidinamine (**42**)

White solid; yield: 210.1 mg (68%). MP: 80–81 °C. IR (ATR): 3265 cm^–1^ (NH). ^1^H NMR (600 MHz, CDCl_3_): δ 7.31–7.28 (m, 2H), 7.22–7.19 (m, 3H), 7.07 (t, *J* = 8.4 Hz, 1H), 6.26 (ddd, *J* = 8.4, 2.4, 0.6 Hz, 1H), 6.23 (ddd, *J* = 8.4, 2.4, 0.6 Hz, 1H), 6.17 (t, *J* = 2.4 Hz, 1H), 3.78 (s, 3H), 3.56 (br s, 1H), 3.30 (tt, *J* = 10.2, 3.6 Hz, 1H), 2.97–2.96 (m, 2H), 2.84–2.81 (m, 2H), 2.63–2.61 (m, 2H), 2.20 (td, *J* = 10.2, 1.8 Hz, 2H), 2.10–2.08 (m, 2H), 1.51 (ddd, *J* = 24.0, 10.2, 3.6 Hz, 2H). ^13^C NMR (150 MHz, CDCl_3_): δ 160.9, 148.5, 140.4, 130.0, 128.7, 128.4, 126.0, 106.4, 102.1, 99.3, 60.6, 55.1, 52.4, 50.0, 33.9, 32.6. HRMS (ESI): *m*/*z* [M + H]^+^ calcd for C_20_H_27_N_2_O, 311.2118; found, 311.2118.

#### *N*-(2-Methylphenyl)-1-(2-phenylethyl)-4-piperidinamine (**43**)

White solid; yield: 1.13 g (77%). MP: 61 °C. IR (ATR): 3411 cm^–1^ (NH). ^1^H NMR (600 MHz, CDCl_3_): δ 7.31–7.28 (m, 2H), 7.22–7.19 (m, 3H), 7.10 (td, *J* = 7.2, 1.2 Hz, 1H), 7.06 (d, *J* = 7.2 Hz, 1H), 6.65–6.62 (m, 2H), 3.39 (tt, *J* = 10.8, 3.6 Hz, 1H), 3.39 (br s, 1H), 2.98–2.96 (m, 2H), 2.84–2.82 (m, 2H), 2.64–2.62 (m, 2H), 2.25 (td, *J* = 10.8, 3.6 Hz, 2H), 2.13 (s, 3H), 2.13–2.12 (m, 2H), 1.55 (ddd, *J* = 24.0, 10.8, 3.6 Hz, 2H). ^13^C NMR (150 MHz, CDCl_3_): δ 145.0, 140.4, 130.4, 128.7, 128.4, 127.1, 126.0, 121.9, 116.7, 110.3, 60.6, 52.5, 49.7, 33.9, 32.7, 17.5. HRMS (ESI): *m*/*z* [M + H]^+^ calcd for C_20_H_27_N_2_, 295.2169; found, 295.2170.

#### *N*-(2,6-Dimethylphenyl)-1-(2-phenylethyl)-4-piperidinamine (**44**)

White solid; yield: 944.5 mg (61%). MP 70 °C. IR (ATR): 3367 cm^–1^ (NH). ^1^H NMR (600 MHz, CDCl_3_): δ 7.28 (t, *J* = 7.8 Hz, 2H), 7.21–7.18 (m, 3H), 6.98 (d, *J* = 7.8 Hz, 2H), 6.80 (t, *J* = 7.8 Hz, 1H), 3.03–2.98 (m, 3H), 2.82–2.79 (m, 2H), 2.61–2.58 (m, 2H), 2.27 (s, 6H), 2.05 (td, *J* = 12.0, 2.4 Hz, 2H), 1.96–1.94 (m, 2H), 1.50 (ddd, *J* = 24.0, 12.0, 2.4 Hz, 2H). ^13^C NMR (150 MHz, CDCl_3_): δ 144.7, 140.4, 129.2, 128.8, 128.7, 128.4, 126.0, 121.5, 60.5, 54.3, 53.0, 34.0, 33.9, 19.0. HRMS (ESI): *m*/*z* [M + H]^+^ calcd for C_21_H_29_N_2_, 309.2325; found, 309.2325.

#### *N*-(2-Chloro-6-methylphenyl)-1-(2-phenylethyl)-4-piperidinamine (**45**)

White solid; yield: 311.4 mg (19%). MP 62–63 °C. IR (ATR): 3358 cm^–1^ (NH). ^1^H NMR (600 MHz, CDCl_3_): δ 7.30–7.27 (m, 2H), 7.21–7.17 (m, 3H), 7.18 (dd, *J* = 7.8, 1.2 Hz, 1H), 7.02 (d, *J* = 7.8 Hz, 1H), 6.80 (t, *J* = 7.8 Hz, 1H), 3.59 (br s, 1H), 3.16 (tt, *J* = 10.8, 3.6 Hz, 1H), 2.98–2.96 (m, 2H), 2.82–2.79 (m, 2H), 2.61–2.58 (m, 2H), 2.31 (s, 3H), 2.09 (t, *J* = 10.8 Hz, 2H), 1.94–1.92 (m, 2H), 1.56 (ddd, *J* = 24.0, 10.8, 3.6 Hz, 2H). ^13^C NMR (150 MHz, CDCl_3_): δ 142.9, 140.4, 131.3, 129.8, 128.7, 128.4, 127.2, 126.8, 126.0, 122.0, 60.6, 54.0, 52.7, 33.9, 33.5, 19.4. HRMS (ESI): *m*/*z* [M + H]^+^ calcd for C_20_H_26_ClN_2_, 329.1779; found, 329.1779.

#### *N*-(2-Ethyl-6-methylphenyl)-1-(2-phenylethyl)-4-piperidinamine (**46**)

Oil; yield: 1.04 g (64%). IR (ATR): 3373 cm^–1^ (NH). ^1^H NMR (600 MHz, CDCl_3_): δ 7.28 (t, *J* = 7.2 Hz, 2H), 7.20–7.18 (m, 3H), 7.02 (d, *J* = 7.2 Hz, 1H), 6.99 (d, *J* = 7.2 Hz, 1H), 6.86 (t, *J* = 7.2 Hz, 1H), 3.00–2.95 (m, 3H), 2.82–2.79 (m, 2H), 2.64 (q, *J* = 7.8 Hz, 2H), 2.60–2.57 (m, 2H), 2.28 (s, 3H), 2.04 (td, *J* = 12.0, 2.4 Hz, 2H), 1.96–1.94 (m, 2H), 1.50 (ddd, *J* = 24.0, 12.0, 2.4 Hz, 2H), 1.23 (t, *J* = 7.2 Hz, 3H). An NH peak was not observed. ^13^C NMR (150 MHz, CDCl_3_): δ 144.0, 140.4, 135.2, 129.6, 128.71, 128.67, 128.4, 126.6, 126.0, 121.8, 60.5, 54.9, 53.1, 33.9, 24.5, 19.2, 14.4; several signals overlapped. HRMS (ESI): *m*/*z* [M + H]^+^ calcd for C_22_H_31_N_2_, 323.2482; found, 323.2483.

#### *N*-(2-Isopropyl-6-methylphenyl)-1-(2-phenylethyl)-4-piperidinamine (**47**)

Oil; yield: 214.0 mg (64%). IR (ATR): 3373 cm^–1^ (NH). ^1^H NMR (600 MHz, CDCl_3_): δ 7.30–7.27 (m, 2H), 7.20–7.18 (m, 3H), 7.09 (dd, *J* = 7.8, 1.2 Hz, 1H), 6.98 (dt, *J* = 7.8 Hz, 1H), 6.92 (t, *J* = 7.8 Hz, 1H), 3.22 (sep, *J* = 7.2 Hz, 1H), 3.01–2.99 (m, 2H), 2.91 (tt, *J* = 10.8, 3.6 Hz, 1H), 2.81–2.79 (m, 2H), 2.60–2.57 (m, 2H), 2.29 (s, 3H), 2.01 (td, *J* = 11.4, 1.8 Hz, 2H), 1.96–1.94 (m, 2H), 1.51 (ddd, *J* = 23.4, 12.0, 3.6 Hz, 2H), 1.22 (d, *J* = 7.2 Hz, 6H). An NH peak was not observed. ^13^C NMR (150 MHz, CDCl_3_): δ 143.0, 140.8, 140.4, 130.3, 128.6, 128.4, 128.3, 126.0, 123.8, 122.3, 60.5, 55.8, 53.2, 34.0, 33.8, 27.6, 24.0, 19.3. HRMS (ESI): *m*/*z* [M + H]^+^ calcd for C_23_H_33_N_2_, 337.2638; found, 337.2636.

#### *N*-(2-Fluoro-6-methylphenyl)-1-(2-phenylethyl)-4-piperidinamine (**48**)

Oil; yield: 87.5 mg (28%). IR (ATR): 3369 cm^–1^ (NH). ^1^H NMR (600 MHz, CDCl_3_): δ 7.28 (m, 2H), 7.20 (m, 3H), 6.87 (t, *J* = 7.8 Hz, 2H), 6.74 (td, *J*_H–H_ = 7.8 Hz, *J*_H–F_ = 6.0 Hz, 1H), 3.30 (tt, *J* = 12.0, 4.8 Hz, 1H), 2.96 (m, 2H), 2.81 (m, 2H), 2.60 (m, 2H), 2.24 (s, 3H), 2.13 (t, *J* = 12.0 Hz, 2H), 2.00 (m, 2H), 1.48 (ddd, *J* = 24.0, 10.2, 3.6 Hz, 2H). NH peak was not observed. ^13^C NMR (150 MHz, CDCl_3_): δ 154.5 (d, ^1^*J*_C–F_ = 238.4 Hz), 140.4, 133.6 (d, ^1^*J*_C–F_ = 10.0 Hz), 129.8 (d, ^1^*J*_C–F_ = 2.9 Hz), 128.7, 128.4, 126.06, 126.04, 126.02, 120.3 (d, ^1^*J*_C–F_ = 8.6 Hz), 113.6 (d, ^1^*J*_C–F_ = 21.6 Hz), 60.6, 53.3, 52.6, 33.9, 33.7, 18.1; One C–F coupling is not described. HRMS (ESI): *m*/*z* [M + H]^+^ calcd for C_20_H_26_FN_2_, 313.2075; found, 313.2074.

#### *N*-(2-Chlorophenyl)-1-(2-phenylethyl)-4-piperidinamine (**49**)

Oil; yield: 470.0 mg (30%). IR (ATR): 3415 cm^–1^ (NH). ^1^H NMR (600 MHz, CDCl_3_): δ 7.29 (m, 2H), 7.25 (dd, *J* = 7.8, 1.8 Hz, 1H), 7.21 (m, 3H), 7.12 (td, *J* = 7.8, 1.8 Hz, 1H), 6.68 (dd, *J* = 7.8, 1.8 Hz, 1H), 6.61 (td, *J* = 7.8, 1.8 Hz, 1H), 4.24 (d, *J* = 7.8 Hz, 1H), 3.38 (m, 1H), 2.95 (m, 2H), 2.83 (m, 2H), 2.63 (m, 2H), 2.25 (t, *J* = 10.8 Hz, 2H), 2.10 (m, 2H), 1.59 (ddd, *J* = 24.0, 10.8, 3.6 Hz, 2H). ^13^C NMR (150 MHz, CDCl_3_): δ 142.9, 140.4, 129.3, 128.7, 128.4, 127.7, 126.0, 119.2, 116.9, 111.7, 60.6, 52.2, 49.6, 33.9, 32.3. HRMS (ESI): *m*/*z* [M + H]^+^ calcd for C_19_H_24_ClN_2_, 315.1623; found, 315.1623.

#### *N*-(2-Chloro-6-fluorophenyl)-1-(2-phenylethyl)-4-piperidinamine (**50**)

Oil: 46.3 mg (6%). IR (ATR): 3383 cm^–1^ (NH). ^1^H NMR (600 MHz, CDCl_3_): δ 7.29 (m, 2H), 7.20 (m, 3H), 7.08 (dt, *J* = 8.4, 1.2 Hz, 1H), 6.92 (ddd, *J*_H–H_ = 24.0, 1.2 Hz, *J*_H–F_ = 8.4 Hz, 1H), 6.69 (td, *J*_H–H_ = 8.4 Hz, *J*_H–F_ = 5.4 Hz, 1H), 3.80 (s, 1H), 3.59 (m, 1H), 2.93 (m, 2H), 2.81 (m, 2H), 2.61 (m, 2H), 2.19 (t, *J* = 10.2 Hz, 2H), 2.02 (m, 2H), 1.52 (ddd, *J* = 24.0, 10.2, 3.6 Hz, 2H). ^13^C NMR (150 MHz, CDCl_3_): δ 153.4 (d, ^1^*J*_C–F_ = 241.4 Hz) 140.4, 132.8 (d, ^1^*J*_C–F_ = 12.9 Hz), 128.7, 128.4, 126.0, 125.0, 124.0 (d, ^1^*J*_C–F_ = 5.7 Hz), 119.0 (d, ^1^*J*_C–F_ = 8.7 Hz), 115.0 (d, ^1^*J*_C–F_ = 21.6 Hz), 60.5, 52.4, 52.2, 33.9, 33.5. HRMS (ESI): *m*/*z* [M + H]^+^ calcd for C_19_H_23_ClFN_2_, 333.1528; found, 333.1528.

#### *N*-Phenyl-*N*-[1-(2-phenylethyl)-4-piperidinyl]-1*H*-pyrrole-2-carboxamide (**1**)

Oxalyl chloride (137 μL, 1.6 mmol) was added dropwise to a solution of pyrrole-2-carboxylic acid (133.3 mg, 1.2 mmol) in THF containing DMF (2 drops) at 25 °C. After 14 h, the reaction was concentrated. The resulting oily residue was redissolved in CH_2_Cl_2_ (2 mL). To a stirred solution of *N*-phenyl-1-(2-phenylethyl)-4-piperidinamine (**40**) (122.2 mg, 0.4 mmol) in CH_2_Cl_2_ (2 mL), sodium hydride (60% in oil) (48 mg, 1.2 mmol) was added at 0 °C under an argon atmosphere. The mixture was stirred at 25 °C for 30 min and treated with the above-mentioned resulting oily residue. After stirring at 25 °C for 3.5 h, the reaction was quenched with water and extracted with CH_2_Cl_2_. The organic layer was washed with 2 N NaOH and brine, dried over Na_2_SO_4_, and concentrated. The concentrate was purified by silica gel column chromatography (ethyl acetate/hexane = 1:4) to produce **1** as a white solid; yield: 53.3 mg (36%); MP: 204–205 °C. IR (ATR): 1613 cm^–1^ (CO). ^1^H NMR (600 MHz, CDCl_3_): δ 9.58 (m, 1H), 7.45–7.42 (m, 3H), 7.26 (t, *J* = 7.2 Hz, 2H), 7.22–7.16 (m, 5H), 6.79 (dd, *J* = 6.0, 3.0 Hz, 1H), 5.87 (dd, *J* = 6.0, 3.0 Hz, 1H), 4.83 (tt, *J* = 12.0, 2.4 Hz, 1H), 4.60 (s, 1H), 3.06–3.04 (m, 2H), 2.76–2.73 (m, 2H), 2.58–2.55 (m, 2H), 2.21 (td, *J* = 12.0, 2.4 Hz, 2H), 1.90–1.88 (m, 2H), 1.58 (ddd, *J* = 24.0, 12.0, 4.2 Hz, 2H). ^13^C NMR (150 MHz, CDCl_3_): δ 160.9, 140.2, 138.6, 131.3, 129.3, 128.9, 128.6, 128.4, 126.0, 125.5, 120.6, 113.2, 109.7, 60.5, 53.1, 53.0, 33.8, 30.4. HRMS: *m*/*z* [M + H]^+^ calcd for C_24_H_28_N_3_O, 374.2227; found, 374.2228.

#### 4-Nitro-*N*-phenyl-*N*-[1-(2-phenylethyl)-4-piperidinyl]benzamide (**2**)

Sodium hydride (60% in oil) (48 mg, 1.2 mmol) was added to a stirred solution of *N*-phenyl-1-(2-phenylethyl)-4-piperidinamine (**40**) (122.2 mg, 0.40 mmol) in CH_2_Cl_2_ (4 mL) at 0 °C. The mixture was stirred at 25 °C for 20 min and treated with 4-nitrobenzoyl (222.7 mg, 1.2 mmol). After stirring at 25 °C for 8.5 h, the reaction was quenched with 2 N NaOH and extracted with diethyl ether. The organic layer was washed with 2 N NaOH and brine, dried over Na_2_SO_4_, and concentrated. The concentrate was purified by silica gel column chromatography (dichloromethane/methanol = 20:1) to produce **2** as a yellow solid; 155.7 mg (91%). MP: 152–153 °C. IR (ATR): 1641 cm^–1^ (CO). ^1^H NMR (600 MHz, CDCl_3_): δ 7.98 (d, *J* = 7.8 Hz, 2H), 7.38 (d, *J* = 7.8 Hz, 2H), 7.27 (t, *J* = 7.8 Hz, 2H), 7.22–7.20 (m, 3H), 7.18 (t, *J* = 7.8 Hz, 3H), 7.00 (d, *J* = 7.8 Hz, 2H), 4.79 (t, *J* = 12.0 Hz, 1H), 3.10–3.08 (m, 2H), 2.78–2.76 (m, 2H), 2.61–2.58 (m, 2H), 2.26 (t, *J* = 12.0 Hz, 2H), 1.97 (d, *J* = 12.0 Hz, 2H), 1.65–1.63 (m, 2H). ^13^C NMR (150 MHz, CDCl_3_): δ 168.7, 147.5, 143.1, 138.3, 130.7, 129.1, 128.9, 128.6, 128.4, 128.3, 126.1, 122.9, 60.3, 53.8, 53.0, 33.7, 30.4; several signals overlapped. HRMS: *m*/*z* [M + H]^+^ calcd for C_26_H_28_N_3_O_3_, 430.2125; found, 430.2125.

Compounds **3–38** were prepared from the corresponding fentanyl analogue intermediates (**40–50**) according to a procedure similar to that described for preparing **2** from **40**.

#### Methyl *N*-(4-Methoxyphenyl)-*N*-[1-(2-phenylethyl)-4-piperidinyl]carbamate (**3**)

White solid; yield: 107.8 mg (73%); MP: 150–151 °C. IR (ATR): 1684 cm^–1^ (CO). ^1^H NMR (600 MHz, CDCl_3_): δ 7.27–7.25 (m, 2H), 7.17 (dt, *J* = 8.4, 1.2 Hz, 1H), 7.16 (dd, *J* = 8.4, 1.2 Hz, 2H), 6.98 (d, *J* = 8.4 Hz, 2H), 6.86 (dt, *J* = 8.4, 2.4 Hz, 2H), 4.23 (m, 1H), 3.80 (s, 3H), 3.63 (s, 3H), 3.01–2.99 (m, 2H), 2.74–2.72 (m, 2H), 2.55–2.52 (m, 2H), 2.12 (t, *J* = 12.0 Hz, 2H), 1.84–1.81 (m, 2H), 1.49 (ddd, *J* = 25.2, 12.0, 3.0 Hz, 2H). ^13^C NMR (150 MHz, CDCl_3_): δ 158.8, 156.5, 140.2, 131.0, 128.6, 128.4, 126.0, 114.0, 60.4, 55.4, 54.9, 53.1, 52.8, 33.8, 30.8; several signals overlapped. HRMS: *m*/*z* [M + H]^+^ calcd for C_22_H_29_N_2_O_3_, 369.2173; found, 369.2173.

#### Methyl *N*-(3-Methoxyphenyl)-*N*-[1-(2-phenylethyl)-4-piperidinyl]carbamate (**4**)

White solid; yield: 50.8 mg (34%). MP: 113–114 °C. IR (ATR): 1703 cm^–1^ (CO). ^1^H NMR (600 MHz, CDCl_3_): δ 7.28–7.24 (m, 2H), 7.19 (d, *J* = 7.2 Hz, 1H), 7.16 (d, *J* = 7.2 Hz, 2H), 6.86 (dd, *J* = 8.4, 2.4 Hz, 2H), 6.68 (d, *J* = 8.4 Hz, 1H), 6.62 (t, *J* = 2.4 Hz, 1H), 4.23 (t, *J* = 12.0 Hz, 1H), 3.80 (s, 3H), 3.64 (s, 3H), 3.13–2.93 (m, 2H), 2.82–2.64 (m, 2H), 2.63–2.43 (m, 2H), 2.24–2.03 (m, 2H), 1.86 (d, *J* = 12.0 Hz, 2H), 1.61–1.54 (m, 2H). ^13^C NMR (150 MHz, CDCl_3_): δ 159.8, 156.1, 139.0, 129.3, 128.6, 128.4, 126.1, 122.4, 116.2, 113.0, 60.3, 55.3, 53.1, 52.8, 33.7, 30.7; several signals overlapped. HRMS: *m*/*z* [M + H]^+^ calcd for C_22_H_29_N_2_O_3_, 369.2173; found, 369.2170.

#### *N*-(3-Methoxyphenyl)-*N*-[1-(2-phenylethyl)-4-piperidinyl]hexanamide (**5**)

Oil; yield: 148.2 mg (91%). IR (ATR): 1650 cm^–1^ (CO). ^1^H NMR (600 MHz, CDCl_3_): δ 7.29 (t, *J* = 7.8 Hz, 1H), 7.25–7.24 (m, 2H), 7.18–7.17 (m, 1H), 7.15 (dd, *J* = 7.8, 1.2 Hz, 2H), 6.90 (ddd, *J* = 7.8, 2.4, 1.2 Hz, 1H), 6.66 (d, *J* = 7.8, 2.4, 1.2 Hz, 1H), 6.60 (t, *J* = 2.4 Hz, 1H), 4.66 (tt, *J* = 12.0, 4.2 Hz, 1H), 3.81 (s, 3H), 3.04–3.02 (m, 2H), 2.75–2.73 (m, 2H), 2.57–2.54 (m, 2H), 2.20–2.16 (m, 2H), 1.95 (t, *J* = 7.2 Hz, 2H), 1.80 (t, *J* = 7.2 Hz, 2H), 1.58–1.52 (m, 2H), 1.47 (tdd, *J* = 24.0, 12.0, 2.4 Hz, 1H), 1.36–1.12 (m, 6H), 0.82 (t, *J* = 7.2 Hz, 2H). ^13^C NMR (150 MHz, CDCl_3_): δ 172.8, 160.1, 140.1, 139.9, 129.8, 128.6, 128.4, 126.0, 122.7, 116.4, 113.4, 60.3, 55.4, 52.9, 52.0, 34.9, 33.6, 31.5, 30.4, 30.3, 25.2, 22.4, 13.9; several signals overlapped. HRMS: *m*/*z* [M + H]^+^ calcd for C_26_H_37_N_2_O_2_, 409.2850; found, 409.2849.

#### *N*-(2-Methylphenyl)-*N*-[1-(2-phenylethyl)-4-piperidinyl]acetamide (**6**)

Oil; yield: 111.5 mg (83%). IR (ATR): 1651 cm^–1^ (CO). ^1^H NMR (600 MHz, CDCl_3_): δ 7.28 (dd, *J* = 7.8, 1.2 Hz, 1H), 7.26 (t, *J* = 7.8 Hz, 3H), 7.21 (td, *J* = 7.8, 1.2 Hz, 1H), 7.19–7.15 (m, 3H), 7.05 (dd, *J* = 7.8, 1.2 Hz, 1H), 4.61 (tt, *J* = 12.0, 4.2 Hz, 1H), 3.03 (ddd, *J* = 10.8, 5.4, 3.6 Hz, 1H), 2.95 (ddd, *J* = 10.8, 5.4, 3.6 Hz, 1H), 2.74–2.72 (m, 2H), 2.55–2.52 (m, 2H), 2.24 (s, 3H), 2.17 (td, *J* = 12.0, 2.4 Hz, 1H), 2.12 (td, *J* = 12.0, 2.4 Hz, 1H), 2.04–2.01 (m, 1H), 1.74–1.70 (m, 1H), 1.71 (s, 3H), 1.63 (ddd, *J* = 24.0, 12.0, 3.6 Hz, 1H), 1.29 (ddd, *J* = 24.0, 12.0, 3.6 Hz, 1H). ^13^C NMR (150 MHz, CDCl_3_): δ 170.5, 140.2, 138.6, 137.0, 131.4, 130.3, 128.6, 128.4, 128.4, 126.9, 126.0, 60.5, 53.5, 53.10, 53.07, 33.8, 31.0, 29.4, 23.0, 18.2. HRMS (ESI): *m*/*z* [M + H]^+^ calcd for C_22_H_29_N_2_O, 337.2274; found, 337.2275.

#### *N*-(2-Methyphenyl)-*N*-[1-(2-phenylethyl)-4-piperidinyl]propionamide (**7**)

Oil; yield: 97.7 mg (70%). IR (ATR): 1651 cm^–1^ (CO). ^1^H NMR (600 MHz, CDCl_3_): δ 7.29–7.24 (m, 4H), 7.20 (dt, *J* = 7.8, 1.2 Hz, 1H), 7.16 (t, *J* = 7.8 Hz, 3H), 7.04 (dd, *J* = 7.8, 1.2 Hz, 1H), 4.63 (tt, *J* = 12.0, 3.6 Hz, 1H), 3.03 (ddd, *J* = 11.4, 5.4, 3.0 Hz, 1H), 2.95 (ddd, *J* = 11.4, 5.4, 3.0 Hz, 1H), 2.74–2.72 (m, 2H), 2.55–2.52 (m, 2H), 2.22 (s, 3H), 2.17 (td, *J* = 12.0, 2.4 Hz, 1H), 2.12 (td, *J* = 12.0, 2.4 Hz, 1H), 2.03–2.00 (m, 1H), 1.85 (ddq, *J* = 34.2, 16.8, 7.8 Hz, 2H), 1.73–1.69 (m, 1H), 1.62 (ddd, *J* = 24.0, 12.0, 3.6 Hz, 1H), 1.28 (ddd, *J* = 24.0, 12.0, 3.6 Hz, 1H), 1.02 (t, *J* = 7.8 Hz, 3H). ^13^C NMR (150 MHz, CDCl_3_): δ 173.7, 140.2, 138.1, 137.2, 131.3, 130.5, 128.6, 128.35, 128.30, 126.8, 126.0, 60.5, 53.6, 53.2, 53.1, 33.8, 31.0, 29.4, 28.1, 18.3, 9.4. HRMS: *m*/*z* [M + H]^+^ calcd for C_23_H_31_N_2_O, 351.2431; found, 351.2432.

#### *N*-(2-Methylphenyl)-*N*-[1-(2-phenylethyl)-4-piperidinyl]butyramide (**8**)

Oil; yield: 94.5 mg (65%). IR (ATR): 1650 cm^–1^ (CO). ^1^H NMR (600 MHz, CDCl_3_): δ 7.29–7.24 (m, 4H), 7.20 (td, *J* = 7.8, 1.2 Hz, 1H), 7.16 (t, *J* = 7.8 Hz, 3H), 7.03 (d, *J* = 7.8 Hz, 1H), 4.63 (tt, *J* = 12.0, 3.6 Hz, 1H), 3.02 (ddd, *J* = 10.8, 5.4, 3.6 Hz, 1H), 2.95 (ddd, *J* = 10.8, 5.4, 3.6 Hz, 1H), 2.74–2.72 (m, 2H), 2.55–2.52 (m, 2H), 2.22 (s, 3H), 2.17 (td, *J* = 12.0, 2.4 Hz, 1H), 2.12 (td, *J* = 12.0, 2.4 Hz, 1H), 2.03–2.00 (m, 1H), 1.88–1.77 (m, 2H), 1.71–1.68 (m, 1H), 1.62 (ddd, *J* = 24.0, 12.0, 4.2 Hz, 1H), 1.56 (dtd, *J* = 21.0, 7.8, 1.8 Hz, 2H), 1.27 (ddd, *J* = 24.0, 12.0, 4.2 Hz, 1H), 0.80 (t, *J* = 7.8 Hz, 3H). ^13^C NMR (150 MHz, CDCl_3_): δ 172.9, 140.3, 138.2, 137.2, 131.3, 130.6, 128.7, 128.5, 128.4, 126.8, 126.1, 60.5, 53.6, 53.2, 53.1, 36.5, 33.8, 31.1, 29.3, 18.6, 18.3, 13.8. HRMS (ESI): *m*/*z* [M + H]^+^ calcd for C_24_H_33_N_2_O, 365.2587; found, 365.2587. Chiral HPLC separation: less polar: [α]_D_^20^ −19.8 (*c* 0.080, CHCl_3_) as 99% ee; more polar: [α]_D_^20^ +19.9 (*c* 0.070, CHCl_3_) as 94% ee. [CHIRALPAK IG, 2-propanol/hexane = 2:8].

#### *N*-(2-Methyphenyl)-*N*-[1-(2-phenylethyl)-4-piperidinyl]cyclopentanecarboxamide (**9**)

White solid; yield: 141.4 mg (91%). MP: 112–113 °C. IR (ATR): 1646 cm^–1^ (CO). ^1^H NMR (600 MHz, CDCl_3_): δ 7.28–7.24 (m, 4H), 7.21 (dd, *J* = 7.8, 1.8 Hz, 1H), 7.18 (d, *J* = 7.8 Hz, 1H), 7.15 (d, *J* = 7.8 Hz, 2H), 7.04 (d, *J* = 7.8 Hz, 1H), 4.62 (tt, *J* = 12.6, 4.2 Hz, 1H), 3.05–2.95 (m, 2H), 2.76–2.73 (m, 2H), 2.56–2.53 (m, 2H), 2.22 (quin, *J* = 8.4 Hz 1H), 2.22 (s, 3H), 2.18–2.11 (m, 2H), 2.03–2.01 (m, 1H), 1.94 (dq, *J* = 12.0, 8.4 Hz, 1H), 1.76–1.62 (m, 4H), 1.59–1.48 (m, 3H), 1.43–1.25 (m, 3H). ^13^C NMR (150 MHz, CDCl_3_): δ 176.9, 138.2, 137.3, 131.1, 130.7, 128.6, 128.4, 128.2, 126.6, 126.0, 60.4, 53.6, 53.2, 53.1, 42.7, 33.7, 31.8, 30.9, 30.2, 29.3, 26.4, 26.1, 18.5; several signals overlapped. HRMS (ESI): *m*/*z* [M + H]^+^ calcd for C_26_H_35_N_2_O, 391.2744; found, 391.2744.

#### *N*-(2-Methyphenyl)-*N*-[1-(2-phenylethyl)-4-piperidinyl]cyclohexanecarboxamide (**10**)

White solid; yield: 89.1 mg (55%); MP: 154–155 °C. IR (ATR): 1647 cm^–1^ (CO). ^1^H NMR (600 MHz, CDCl_3_): δ 7.29–7.24 (m, 4H), 7.20 (dd, *J* = 7.2, 1.8 Hz, 1H), 7.18 (d, *J* = 7.2 Hz, 1H), 7.15 (d, *J* = 7.2 Hz, 2H), 7.04 (d, *J* = 7.2 Hz, 1H), 4.60 (tt, *J* = 12.0, 3.6 Hz, 1H), 3.04–3.02 (m, 1H), 2.96–2.94 (m, 1H), 2.77–2.72 (m, 2H), 2.55–2.52 (m, 2H), 2.21 (s, 3H), 2.17 (td, *J* = 12.0, 2.4 Hz, 1H), 2.14 (td, *J* = 12.0, 2.4 Hz, 1H), 2.02–1.99 (m, 1H), 1.79 (tt, *J* = 12.0, 3.6 Hz, 1H), 1.70–1.51 (m, 8H), 1.34 (ddd, *J* = 24.0, 12.0, 3.6 Hz, 1H), 1.25 (ddd, *J* = 24.0, 12.0, 3.6 Hz, 1H), 1.16 (ddt, *J* = 24.0, 12.0, 3.6 Hz, 1H), 0.98 (ddt, *J* = 24.0, 12.0, 3.6 Hz, 1H), 0.82 (ddt, *J* = 24.0, 12.0, 3.6 Hz, 1H). ^13^C NMR (150 MHz, CDCl_3_): δ 176.3, 140.2, 138.0, 137.2, 131.1, 130.3, 128.6, 128.3, 128.2, 126.6, 126.0, 60.4, 53.4, 53.1, 42.5, 33.8, 31.0, 29.9, 29.3, 28.9, 25.7, 25.6, 25.3, 18.4; several signals overlapped. HRMS: *m*/*z* [M + H]^+^ calcd for C_27_H_37_N_2_O, 405.2900; found, 405.2901.

#### 4-Chloro-*N*-(2-methylphenyl)-*N*-[1-(2-phenylethyl)-4-piperidinyl]benzamide (**11**)

White solid; yield: 117.8 mg (68%); MP: 110–111 °C. IR (ATR): 1639 cm^–1^ (CO). ^1^H NMR (600 MHz, CDCl_3_): δ 7.27 (t, *J* = 7.2 Hz, 2H), 7.24–7.12 (m, 8H), 7.06 (d, *J* = 7.2 Hz, 2H), 7.01 (d, *J* = 7.2 Hz, 1H), 4.72 (t, *J* = 12.0 Hz, 1H), 3.11–3.09 (m, 1H), 3.00–2.99 (m, 1H), 2.77–2.74 (m, 2H), 2.59–2.56 (m, 2H), 2.26–2.16 (m, 3H), 2.01 (s, 3H), 1.85 (ddd, *J* = 24.0, 12.0, 3.6 Hz, 1H), 1.73–1.71 (m, 1H), 1.41 (ddd, *J* = 24.0, 12.0, 3.6 Hz, 1H). ^13^C NMR (150 MHz, CDCl_3_): δ 169.3, 140.2, 138.3, 136.7, 135.4, 135.3, 131.4, 130.9, 129.7, 128.6, 128.4, 128.3, 127.6, 126.3, 126.0, 60.4, 55.1, 53.2, 53.1, 33.8, 31.5, 29.2, 18.5. HRMS: *m*/*z* [M + H]^+^ calcd for C_27_H_30_ClN_2_O, 433.2041; found, 433.2042.

#### 2-Chloro-*N*-(2-methylphenyl)-*N*-[1-(2-phenylethyl)-4-piperidinyl]benzamide (**12**)

White solid; yield: 20.0 mg (83%); *E*/*Z* = 6.1:1; MP: 102–105 °C. IR (ATR): 1638 cm^–1^ (CO). ^1^H NMR (600 MHz, CDCl_3_): δ(*E*-isomer) 7.33–7.30 (m, 1H), 7.27 (t, *J* = 7.8 Hz, 2H), 7.20–7.16 (m, 4H), 7.10–7.09 (m, 1H), 7.07–7.02 (m, 4H), 6.98 (td, *J* = 7.8, 1.8 Hz, 1H), 4.77 (tt, *J* = 12.0, 3.6 Hz, 1H), 3.10–3.08 (m, 1H), 3.01–2.99 (m, 1H), 2.77–2.74 (m, 2H), 2.59–2.56 (m, 2H), 2.29 (s, 3H), 2.28–2.22 (m, 2H), 2.18 (td, *J* = 12.0, 2.4 Hz, 1H), 1.85–1.79 (m, 2H), 1.41 (ddd, *J* = 24.6, 12.0, 4.2 Hz, 1H). δ(*Z*-isomer) 7.49–7.46 (m, 1H), 7.43–7.35 (m, 3H), 7.33–7.30 (m, 1H), 7.23 (t, *J* = 7.8 Hz, 3H), 7.20–7.16 (m, 1H), 7.14 (d, *J* = 7.8 Hz, 1H), 7.10–7.09 (m, 2H), 7.07–7.02 (m, 1H), 3.55 (tt, *J* = 12.0, 3.6 Hz, 1H), 2.92–2.84 (m, 2H), 2.66–2.64 (m, 2H), 2.44 (s, 3H), 2.43–2.41 (m, 2H), 1.97–1.88 (m, 2H), 1.78–1.73 (m, 2H), 1.70–1.65 (m, 2H). ^13^C NMR (150 MHz, CDCl_3_): δ 168.14, 168.09, 140.2, 140.1, 137.4, 137.2, 137.0, 136.9, 131.4, 131.2, 130.8, 130.5, 130.3, 130.1, 130.04, 129.96, 129.7, 129.6, 129.5, 128.6, 128.5, 128.4, 128.2, 127.9, 127.2, 127.1, 126.9, 126.8, 126.6, 126.5, 126.0, 125.8, 60.4, 60.2, 59.0, 54.8, 53.1, 53.0, 52.9, 33.82, 33.77, 32.34, 32.32, 30.6, 29.8, 29.3, 19.2, 18.9; two diastereomers, not distinguished, several signals overlapped. HRMS: *m*/*z* [M + H]^+^ calcd for C_27_H_30_ClN_2_O, 433.2041; found, 433.2042.

#### *N*-(2,6-Dimethyphenyl)-*N*-[1-(2-phenylethyl)-4-piperidinyl]propionamide (**13**)

Oil: 144.3 mg (99%). IR (ATR): 1654 cm^–1^ (CO). ^1^H NMR (600 MHz, CDCl_3_): δ 7.26 (t, *J* = 7.8 Hz, 2H), 7.19–7.13 (m, 4H), 7.09 (d, *J* = 7.8 Hz, 2H), 4.20 (tt, *J* = 12.0, 3.6 Hz, 1H), 2.99–2.97 (m, 2H), 2.76–2.73 (m, 2H), 2.55–2.53 (m, 2H), 2.21 (s, 6H), 2.11 (td, *J* = 12.0, 1.8 Hz, 2H), 1.99–1.97 (m, 2H), 1.80 (q, *J* = 7.8 Hz, 2H), 1.42 (ddd, *J* = 24.6, 12.0, 3.6 Hz, 2H), 1.02 (t, *J* = 7.8 Hz, 3H). ^13^C NMR (150 MHz, CDCl_3_): δ 174.2, 140.3, 138.5, 137.3, 128.7, 128.6, 128.3, 127.9, 126.0, 60.5, 55.7, 53.2, 33.8, 30.4, 27.9, 19.0, 9.3. HRMS: *m*/*z* [M + H]^+^ calcd for C_24_H_33_N_2_O, 365.2587; found, 365.2587.

#### *N*-(2,6-Dimethylphenyl)-*N*-[1-(2-phenylethyl)-4-piperidinyl]hexanamide (**14**)

Oil; yield: 148.5 mg (91%). IR (ATR): 1652 cm^–1^ (CO). ^1^H NMR (600 MHz, CDCl_3_): δ 7.26 (t, *J* = 7.8 Hz, 2H), 7.16–7.13 (m, 4H), 7.09 (d, *J* = 7.8 Hz, 2H), 4.20 (tt, *J* = 12.0, 3.6 Hz, 1H), 2.99–2.97 (m, 2H), 2.76–2.73 (m, 2H), 2.55–2.53 (m, 2H), 2.21 (s, 6H), 2.11 (td, *J* = 12.0, 1.8 Hz, 2H), 1.98–1.96 (m, 2H), 1.78 (t, *J* = 7.8 Hz, 2H), 1.55 (dt, *J* = 15.0, 7.8 Hz, 2H), 1.42 (ddd, *J* = 24.6, 12.0, 3.6 Hz, 2H), 1.25–1.19 (m, 2H), 1.18–1.13 (m, 2H), 0.83 (t, *J* = 7.8 Hz, 3H). ^13^C NMR (150 MHz, CDCl_3_): δ 173.5, 140.3, 138.5, 137.3, 128.7, 128.6, 128.3, 127.9, 126.0, 60.4, 55.7, 53.2, 34.5, 33.8, 31.6, 30.4, 24.6, 22.5, 19.1, 13.9. HRMS (ESI): *m*/*z* [M + H]^+^ calcd for C_27_H_39_N_2_O, 407.3057; found, 407.3057.

#### *N*-(2,6-Dimethyphenyl)-*N*-[1-(2-phenylethyl)-4-piperidinyl]-2-furancarboxamide (**15**)

White solid: 195.4 mg (61%): MP: 130–131 °C. IR (ATR): 1641 cm^–1^ (CO). ^1^H NMR (600 MHz, CDCl_3_): δ 7.36 (dd, *J* = 1.8, 0.6 Hz, 1H), 7.28–7.26 (m, 2H), 7.21 (t, *J* = 7.8 Hz, 1H), 7.19–7.17 (m, 3H), 7.11 (d, *J* = 7.8 Hz, 2H), 6.13 (dd, *J* = 3.6, 1.8 Hz, 1H), 5.28 (dd, *J* = 3.6, 1.8 Hz, 1H), 4.33 (tt, *J* = 12.0, 3.6 Hz, 1H), 3.04–3.02 (m, 2H), 2.78–2.75 (m, 2H), 2.59–2.56 (m, 2H), 2.20 (s, 6H), 2.17 (td, *J* = 12.0, 1.8 Hz, 2H), 2.06–2.04 (m, 2H), 1.60 (ddd, *J* = 24.0, 12.0, 3.6 Hz, 2H). ^13^C NMR (150 MHz, CDCl_3_): δ 159.4, 147.4, 144.4, 140.3, 138.4, 138.1, 128.8, 128.64, 128.56, 128.4, 126.0, 114.5, 111.1, 60.4, 56.8, 53.2, 33.8, 30.1, 19.0. HRMS: *m*/*z* [M + H]^+^ calcd for C_26_H_31_N_2_O_2_, 403.2380; found, 403.2379.

#### 2-Fluoro-*N*-(2,6-dimethylphenyl)-*N*-[1-(2-phenylethyl)-4-piperidinyl]benzamide (**16**)

Solid; yield: 98.6 mg (57%); *E*/*Z* = 8.1:1; MP: 81–82 °C. IR (ATR): 1635 cm^–1^ (CO). ^1^H NMR (600 MHz, CDCl_3_): δ(*E*-isomer) 7.29–7.27 (m, 2H), 7.20–7.18 (m, 3H), 7.14–7.10 (m, 1H), 7.07 (td, *J* = 7.2, 1.2 Hz, 1H), 6.97 (dd, *J* = 7.2, 6.6 Hz, 1H), 6.90 (d, *J* = 7.2 Hz, 2H), 6.88–6.83 (m, 2H), 4.27 (tt, *J* = 12.0, 3.6 Hz, 1H), 3.06–3.04 (m, 2H), 2.79–2.76 (m, 2H), 2.60–2.57 (m, 2H), 2.31 (s, 6H), 2.17 (td, *J* = 12.0, 1.8 Hz, 2H), 2.08–2.06 (m, 2H), 1.68 (ddd, *J* = 24.6, 12.6, 3.6 Hz, 2H). δ(*Z*-isomer) 7.48 (td, *J* = 7.2, 1.2 Hz, 1H), 7.45–7.42 (m, 1H), 7.23 (t, *J* = 7.2 Hz, 3H), 7.17–7.15 (m, 3H), 7.14–7.10 (m, 4H), 3.74 (tt, *J* = 12.0, 3.6 Hz, 1H), 2.90–2.88 (m, 2H), 2.67–2.64 (m, 2H), 2.43–2.41 (m, 2H), 2.35 (s, 6H), 1.84 (m, 4H), 1.60–1.57 (m, 2H). ^13^C NMR (150 MHz, CDCl_3_): δ 167.3, 166.5, 158.7 (d, ^1^*J*_C–F_ = 248.4 Hz), 140.3, 140.1, 138.1, 137.6, 136.1, 131.0, 130.9, 130.5, 130.4, 128.7, 128.5, 128.4, 128.3, 127.8, 127.8, 127.8, 126.0, 125.8, 125.7, 124.7, 123.0 (d, ^1^*J*_C–F_ = 4.2 Hz), 116.1, 115.9, 115.8, 115.6, 60.4, 60.1, 59.5, 57.4, 53.2, 53.1, 33.7, 30.1, 30.0, 19.4, 19.4; two diastereomers, not distinguished, some C–F couplings are not described and several signals overlapped. HRMS: *m*/*z* [M + H]^+^ calcd for C_28_H_32_FN_2_O, 431.2493; found, 431.2494.

#### 2-Chloro-*N*-(2,6-dimethylphenyl)-*N*-[1-(2-phenylethyl)-4-piperidinyl]benzamide (**17**)

Yellow solid; yield: 121.2 mg (68%); *E*/*Z* = 2.3:1; MP 125–126 °C. IR (ATR): 1644 cm^–1^ (CO). ^1^H NMR (600 MHz, CDCl_3_): δ(*E*-isomer) 7.29–7.27 (m, 2H), 7.19–7.18 (m, 3H), 7.16–7.12 (m, 1H), 7.06 (td, *J* = 7.2, 1.2 Hz, 1H), 6.98 (dd, *J* = 8.4, 7.2 Hz, 1H), 6.92 (d, *J* = 7.2 Hz, 2H), 6.87 (td, *J* = 7.2, 1.2 Hz, 1H), 6.83 (dd, *J* = 7.2, 1.2 Hz, 1H), 4.35 (tt, *J* = 12.0, 3.6 Hz, 1H), 3.05–3.04 (m, 2H), 2.80–2.77 (m, 2H), 2.61–2.58 (m, 2H), 2.34 (s, 6H), 2.20 (t, *J* = 12.0 Hz, 2H), 2.14–2.12 (m, 2H), 1.63–1.56 (m, 2H). δ(*Z*-isomer) 7.49–7.47 (m, 1H), 7.45–7.44 (m, 1H), 7.39–7.37 (m, 2H), 7.32–7.29 (m, 1H), 7.23 (t, *J* = 7.2 Hz, 2H), 7.16–7.12 (m, 3H), 7.10–7.09 (m, 2H), 3.67 (tt, *J* = 12.0, 3.6 Hz, 1H), 2.92–2.87 (m, 2H), 2.66–2.63 (m, 2H), 2.44 (s, 3H), 2.43–2.40 (m, 2H), 2.35 (s, 3H), 2.04–2.03 (m, 1H), 1.82–1.76 (m, 2H), 1.72–1.56 (m, 3H). ^13^C NMR (150 MHz, CDCl_3_): δ 168.0, 167.9, 140.3, 140.1, 138.0, 137.9, 137.4, 136.9, 136.4, 135.8, 131.5, 130.3, 130.2, 130.1, 130.04, 130.00, 129.6, 129.5, 129.0, 128.72, 128.67, 128.5, 128.4, 128.3, 127.9, 127.8, 127.7, 127.2, 127.0, 126.1, 126.0, 125.3, 60.4, 60.1, 59.9, 56.9, 53.2, 53.1, 33.8, 33.7, 31.1, 30.8, 30.1, 19.9, 19.6, 19.5; two diastereomers, not distinguished, several signals overlapped. HRMS: *m*/*z* [M + H]^+^ calcd for C_28_H_32_ClN_2_O, 447.2198; found, 447.2198.

#### 2-Methyl-*N*-(2,6-dimethylphenyl)-*N*-[1-(2-phenylethyl)-4-piperidinyl]benzamide (**18**)

White solid; yield: 100.1 mg (59%); *E*/*Z* = 2.4:1; MP: 119–120 °C. IR (ATR): 1636 cm^–1^ (CO). ^1^H NMR (600 MHz, CDCl_3_): δ(*E*-isomer) 7.28 (t, *J* = 7.8 Hz, 3H), 7.21–7.19 (m, 3H), 7.10–7.08 (m, 2H), 6.91 (d, *J* = 7.8 Hz, 2H), 6.75 (t, *J* = 7.8 Hz, 1H), 6.72 (dd, *J* = 7.8, 1.8 Hz, 1H), 4.32 (tt, *J* = 12.0, 4.0 Hz, 1H), 3.14–3.10 (m, 2H), 2.83 (m, 2H), 2.66–2.63 (m, 2H), 2.48 (s, 3H), 2.27 (s, 6H), 2.27–2.25 (m, 2H), 2.10–2.09 (m, 2H), 1.74–1.72 (m, 2H). δ(*Z*-isomer) 7.38 (d, *J* = 7.8 Hz, 1H), 7.32 (td, *J* = 7.8, 1.2 Hz, 1H), 7.23 (t, *J* = 7.8 Hz, 2H), 7.17–7.12 (m, 3H), 7.02 (td, *J* = 7.8, 1.2 Hz, 3H), 6.97 (dd, *J* = 7.8, 6.6 Hz, 2H), 3.75 (tt, *J* = 12.0, 4.0 Hz, 1H), 2.88–2.86 (m, 2H), 2.6–2.63 (m, 2H), 2.49 (s, 3H), 2.43–2.40 (m, 2H), 2.38 (s, 6H), 1.74–1.72 (m, 6H). ^13^C NMR (150 MHz, CDCl_3_): δ 170.9, 170.8, 140.0, 138.7, 137.6, 137.4, 137.1, 136.5, 136.2, 135.6, 134.6, 130.8, 128.83, 128.81, 128.7, 128.5, 128.43, 128.37, 128.3, 127.6, 126.15, 126.06, 126.0, 125.6, 125.4, 124.1, 60.2, 60.1, 59.3, 56.7, 53.2, 53.0, 33.7, 33.5, 33.4, 29.9, 29.8, 20.3, 19.8, 19.6; two diastereomers, not distinguished, several signals overlapped. HRMS: *m*/*z* [M + H]^+^ calcd for C_29_H_35_N_2_O, 427.2744; found, 427.2745.

#### 2-Bromo-*N*-(2,6-dimethylphenyl)-*N*-[1-(2-phenylethyl)-4-piperidinyl]benzamide (**19**)

White solid; yield: 90.2 mg (46%); *E*/*Z* = 2.0:1; MP: 140–142 °C. IR (ATR): 1642 cm^–1^ (CO). ^1^H NMR (600 MHz, CDCl_3_): δ(*E*-isomer) 7.27 (t, *J* = 7.8 Hz, 2H), 7.19–7.17 (m, 3H), 7.16–7.11 (m, 2H), 7.00–6.96 (m, 2H), 6.92 (d, *J* = 7.8 Hz, 2H), 6.79 (dd, *J* = 7.8, 1.2 Hz, 1H), 4.35 (tt, *J* = 12.0, 3.6 Hz, 1H), 3.06–3.04 (m, 2H), 2.79–2.77 (m, 2H), 2.60–2.58 (m, 2H), 2.35 (s, 6H), 2.20 (t, *J* = 12.0 Hz, 2H), 2.15–2.13 (m, 2H), 1.63–1.52 (m, 2H). δ(*Z*-isomer) 7.66 (d, *J* = 7.8 Hz, 1H), 7.47 (dd, *J* = 7.8, 1.2 Hz, 2H), 7.44–7.42 (m, 2H), 7.31–7.29 (m, 1H), 7.25–7.23 (m, 2H), 7.09 (d, *J* = 7.8 Hz, 2H), 6.90 (d, *J* = 7.8 Hz, 2H), 3.69 (tt, *J* = 12.0, 3.6 Hz, 1H), 2.93–2.86 (m, 2H), 2.66–2.63 (m, 2H), 2.48 (s, 3H), 2.43–2.40 (m, 2H), 2.35 (s, 3H), 2.10–2.07 (m, 1H), 1.79 (ddd, *J* = 24.0, 12.0, 2.4 Hz, 2H), 1.71 (td, *J* = 12.0, 2.4 Hz, 3H). ^13^C NMR (150 MHz, CDCl_3_): δ 168.6, 168.5, 140.3, 140.1, 139.5, 138.0, 137.9, 137.4, 136.9, 135.7, 133.5, 133.2, 130.2, 129.7, 129.1, 128.72, 128.67, 128.5, 128.4, 128.3, 127.9, 127.8, 127.7, 127.5, 127.2, 126.1, 126.0, 125.8, 120.6, 119.3, 60.4, 60.2, 60.0, 56.9, 53.2, 53.1, 33.8, 30.9, 30.2, 20.1, 19.9, 19.4; two diastereomers, not distinguished. HRMS: *m*/*z* [M + H]^+^ calcd for C_28_H_32_BrN_2_O, 491.1693; found, 491.1693.

#### 2-Iodo-*N*-(2,6-dimethylphenyl)-*N*-[1-(2-phenylethyl)-4-piperidinyl]benzamide (**20**)

White solid; yield: 40.9 mg (19%); *E*/*Z* = 2.2:1; MP 143–145 °C. IR (ATR): 1640 cm^–1^ (CO). ^1^H NMR (600 MHz, CDCl_3_): δ(*E*-isomer) 7.29–7.27 (m, 2H), 7.20–7.17 (m, 3H), 7.16–7.08 (m, 3H), 6.92 (d, *J* = 7.2 Hz, 2H), 6.80 (td, *J* = 7.2, 1.8 Hz, 1H), 6.74 (dd, *J* = 7.2, 1.8 Hz, 1H), 4.34 (tt, *J* = 12.0, 3.6 Hz, 1H), 3.06–3.04 (m, 2H), 2.79–2.77 (m, 2H), 2.60–2.58 (m, 2H), 2.35 (s, 6H), 2.24–2.10 (m, 4H), 1.62–1.58 (m, 2H). δ(*Z*-isomer) 7.91 (dd, *J* = 7.8, 1.2 Hz, 1H), 7.77 (dd, *J* = 7.8, 1.2 Hz, 2H), 7.44 (td, *J* = 7.8, 1.2 Hz, 1H), 7.40 (dd, *J* = 7.8, 1.2 Hz, 1H), 7.24 (t, *J* = 7.8 Hz, 2H), 7.16–7.08 (m, 1H), 6.99 (dd, *J* = 7.8, 1.2 Hz, 2H), 6.93–6.90 (m, 2H), 3.69 (tt, *J* = 12.0, 3.6 Hz, 1H), 2.93–2.86 (m, 2H), 2.67–2.64 (m, 2H), 2.55 (s, 3H), 2.43–2.40 (m, 2H), 2.35 (s, 3H), 1.81–1.69 (m, 5H), 1.48 (ddd, *J* = 24.0, 12.0, 3.6 Hz, 1H). ^13^C NMR (150 MHz, CDCl_3_): δ 170.0, 169.6, 143.6, 140.44, 140.34, 140.1, 139.9, 138.1, 138.0, 137.8, 136.9, 135.8, 130.1, 129.8, 129.2, 128.7, 128.6, 128.4, 128.3, 128.04, 127.99, 127.9, 127.7, 127.2, 126.9, 126.4, 126.1, 126.0, 94.6, 92.9, 60.4, 60.2, 60.0, 57.0, 53.2, 53.1, 33.8, 31.4, 30.6, 30.3, 20.3, 19.4; two diastereomers, not distinguished, several signals overlapped. HRMS: *m*/*z* [M + H]^+^ calcd for C_28_H_32_IN_2_O, 539.1554; found, 539.1554.

#### 2,6-Difluoro-*N*-(2,6-dimethylphenyl)-*N*-[1-(2-phenylethyl)-4-piperidinyl]benzamide (**21**)

White solid; yield: 117.5 mg (65%); *E*/*Z* = 2.4:1; MP 131–132 °C. IR (ATR): 1652 cm^–1^ (CO). ^1^H NMR (600 MHz, CDCl_3_): δ(*E*-isomer) 7.27 (t, *J* = 7.8 Hz, 1H), 7.20–7.17 (m, 3H), 7.16–7.12 (m, 1H), 7.07 (tt, *J* = 7.8, 7.2 Hz, 1H), 6.98 (dd, *J* = 7.8, 7.2 Hz, 1H), 6.92 (d, *J* = 7.8 Hz, 2H), 6.66 (t, *J* = 7.8 Hz, 2H), 4.27 (tt, *J* = 12.0, 3.6 Hz, 1H), 3.06–3.04 (m, 2H), 2.79–2.76 (m, 2H), 2.60–2.57 (m, 2H), 2.30 (s, 6H), 2.17 (td, *J* = 12.0, 1.8 Hz, 2H), 2.15–2.12 (m, 2H), 1.67 (ddd, *J* = 24.0, 12.0, 3.6 Hz, 2H). δ(*Z*-isomer) 7.40 (tt, *J* = 7.8, 7.2 Hz, 1H), 7.25–7.23 (m, 3H), 7.20–7.17 (m, 1H), 7.16–7.12 (m, 2H), 7.11–7.10 (m, 2H), 7.02 (dd, *J* = 7.8, 7.2 Hz, 2H), 3.69 (tt, *J* = 12.0, 3.6 Hz, 1H), 2.92–2.91 (m, 2H), 2.67–2.65 (m, 2H), 2.44–2.42 (m, 2H), 2.35 (s, 6H), 1.90–1.87 (m, 2H), 1.80 (td, *J* = 12.0, 1.8 Hz, 2H), 1.61 (ddd, *J* = 24.0, 12.0, 3.6 Hz, 2H). ^13^C NMR (150 MHz, CDCl_3_): δ 163.0, 161.5, 159.8, 159.7, 158.11, 158.05, 158.0, 140.4, 140.1, 138.3, 137.2, 136.8, 135.8, 130.8, 130.23, 130.16, 130.1, 128.8, 128.7, 128.6, 128.4, 128.1, 128.0, 127.9, 126.1, 126.0, 112.00, 111.98, 111.9, 111.8, 111.24, 111.22, 111.10, 111.07, 60.4, 60.2, 60.0, 57.8, 53.2, 53.1, 33.8, 31.2, 30.0, 19.3, 19.1, 19.1; two diastereomers, not distinguished, C–F couplings are not described and several signals overlapped. HRMS: *m*/*z* [M + H]^+^ calcd for C_28_H_31_F_2_N_2_O, 449.2399; found, 449.2399.

#### *N*-(2-Chloro-6-methyphenyl)-*N*-[1-(2-phenylethyl)-4-piperidinyl]propionamide (**22**)

Oil; yield: 105.8 mg (69%). IR (ATR): 1659 cm^–1^ (CO). ^1^H NMR (600 MHz, CDCl_3_): δ 7.31 (t, *J* = 4.8 Hz, 1H), 7.27–7.25 (m, 2H), 7.19 (d, *J* = 4.8 Hz, 2H), 7.20–7.16 (m, 3H), 4.28 (tt, *J* = 12.0, 3.6 Hz, 1H), 3.01–2.96 (m, 2H), 2.76–2.73 (m, 2H), 2.56–2.53 (m, 2H), 2.30 (s, 3H), 2.16–2.08 (m, 3H), 1.97–1.94 (m, 1H), 1.89 (dq, *J* = 16.2, 7.2 Hz, 1H), 1.78 (dq, *J* = 16.2, 7.2 Hz, 1H), 1.51 (ddd, *J* = 24.0, 12.0, 3.6 Hz, 1H), 1.43 (ddd, *J* = 24.0, 12.0, 3.6 Hz, 1H), 1.04 (t, *J* = 7.2 Hz, 3H). ^13^C NMR (150 MHz, CDCl_3_): δ 174.0, 140.3, 139.9, 137.2, 135.4, 129.6, 129.0, 128.6, 128.3, 127.9, 126.0, 60.4, 55.9, 53.2, 53.1, 33.8, 30.8, 29.7, 27.8, 19.4, 9.2. HRMS: *m*/*z* [M + H]^+^ calcd for C_23_H_30_ClN_2_O, 385.2041; found, 385.2042.

#### *N*-(2-Chloro-6-methyphenyl)-*N*-[1-(2-phenylethyl)-4-piperidinyl]-2-furancarboxamide (**23**)

White solid; 165.0 mg (67%); MP: 108–109 °C. IR (ATR): 1646 cm^–1^ (CO). ^1^H NMR (600 MHz, CDCl_3_): δ 7.32–7.30 (m, 2H), 7.28–7.25 (m, 2H), 7.24 (t, *J* = 7.8 Hz, 1H), 7.20–7.17 (m, 4H), 6.18 (dd, *J* = 3.6, 1.8 Hz, 1H), 5.70 (dd, *J* = 3.6, 0.6 Hz, 1H), 4.38 (tt, *J* = 12.0, 3.6 Hz, 1H), 3.06–3.01 (m, 2H), 2.78–2.76 (m, 2H), 2.59–2.56 (m, 2H), 2.30 (s, 3H), 2.21–2.14 (m, 2H), 2.03–2.00 (m, 1H), 1.71 (td, *J* = 12.0, 3.6 Hz, 1H), 1.65 (ddd, *J* = 24.0, 12.0, 3.6 Hz, 2H). ^13^C NMR (150 MHz, CDCl_3_): δ 159.4, 147.4, 144.4, 140.5, 140.3, 137.3, 135.8, 129.4, 129.3, 128.6, 128.3, 127.9, 126.0, 114.7, 111.0, 60.4, 57.2, 53.2, 53.1, 33.8, 30.3, 29.5, 19.4. HRMS: *m*/*z* [M + H]^+^ calcd for C_25_H_28_ClN_2_O_2_, 432.1834; found, 423.1834. Chiral HPLC separation: less polar: [α]_D_^20^ +13.81 (*c* 0.215, CHCl_3_) as 98.8% ee; more polar: [α]_D_^20^ −8.81 (*c* 0.110, CHCl_3_) as 99.1% ee. [CHIRALPAK IG, ethanol/hexane = 3:7].

#### 4-Chloro-*N*-(2-chloro-6-methylphenyl)-*N*-[1-(2-phenylethyl)-4-piperidinyl]benzamide (**24**)

White solid; yield: 60.6 mg (32%); MP: 145 °C. IR (ATR): 1645 cm^–1^ (CO). ^1^H NMR (600 MHz, CDCl_3_): δ 7.30 (dt, *J* = 9.0, 2.4 Hz, 2H), 7.29–7.27 (m, 2H), 7.20–7.12 (m, 4H), 7.10 (dt, *J* = 9.0, 2.4 Hz, 2H), 7.06 (t, *J* = 7.8 Hz, 1H), 7.01 (d, *J* = 7.8 Hz, 1H), 4.23 (tt, *J* = 12.0, 3.6 Hz, 1H), 3.06–3.04 (m, 2H), 2.80–2.77 (m, 2H), 2.60–2.57 (m, 2H), 2.28 (s, 3H), 2.16 (ddd, *J* = 19.2, 12.0, 1.8 Hz, 2H), 2.04–1.98 (m, 2H), 1.86–1.77 (m, 2H). ^13^C NMR (150 MHz, CDCl_3_): δ 170.4, 140.3, 139.4, 138.2, 135.7, 135.4, 135.0, 129.5, 129.0, 128.7, 128.44, 128.37, 127.9, 127.8, 126.0, 60.4, 57.8, 53.2, 33.8, 30.0, 29.7, 19.6; several signals overlapped. HRMS: *m*/*z* [M + H]^+^ calcd for C_27_H_29_Cl_2_N_2_O, 467.1651; found, 467.1652. Chiral HPLC separation: less polar: [α]_D_^20^ −48.6 (*c* 0.085, CHCl_3_) as >99.9% ee; more polar: [α]_D_^20^ +48.4 (*c* 0.080, CHCl_3_) as 99% ee. [CHIRALPAK IG, 2-propanol/hexane = 5:5].

#### *N*-(2-Ethyl-6-methylphenyl)-*N*-[1-(2-phenylethyl)-4-piperidinyl]acetamide (**25**)

White solid; yield: 139.6 mg (98%); MP: 71–72 °C. IR (ATR): 1637 cm^–1^ (CO). ^1^H NMR (600 MHz, CDCl_3_): δ 7.26 (t, *J* = 7.8 Hz, 2H), 7.22 (t, *J* = 7.8 Hz, 1H), 7.17 (td, *J* = 7.8, 1.2 Hz, 4H), 7.10 (dd, *J* = 7.8, 1.2 Hz, 1H), 4.16 (tt, *J* = 12.0, 3.6 Hz, 1H), 2.98–2.96 (m, 2H), 2.75–2.73 (m, 2H), 2.58 (q, *J* = 7.8 Hz, 2H), 2.55–2.52 (m, 2H), 2.23 (s, 3H), 2.11 (tt, *J* = 12.0, 3.6 Hz, 2H), 1.99–1.94 (m, 2H), 1.68 (s, 3H), 1.42 (ddd, *J* = 24.0, 12.0, 3.6 Hz, 2H), 1.24 (t, *J* = 7.8 Hz, 3H). ^13^C NMR (150 MHz, CDCl_3_): δ 171.5, 142.8, 140.5, 138.5, 137.1, 128.8, 128.7, 128.5, 128.4, 126.6, 126.1, 60.6, 56.0, 53.4, 53.3, 33.9, 30.5, 24.2, 23.3, 19.2, 14.3; several signals overlapped. HRMS (ESI): *m*/*z* [M + H]^+^ calcd for C_24_H_33_N_2_O, 365.2587; found, 365.2588.

#### *N*-(2-Ethyl-6-methyphenyl)-*N*-[1-(2-phenylethyl)-4-piperidinyl]propionamide (**26**)

Oil; yield: 77.9 mg (52%). IR (ATR): 1652 cm^–1^ (CO). ^1^H NMR (600 MHz, CDCl_3_): δ 7.26 (t, *J* = 7.2 Hz, 2H), 7.22 (t, *J* = 7.2 Hz, 1H), 7.17 (td, *J* = 7.2, 1.8 Hz, 4H), 7.10 (dd, *J* = 7.2, 1.8 Hz, 1H), 4.18 (tt, *J* = 12.0, 3.6 Hz, 1H), 2.98–2.96 (m, 2H), 2.75–2.73 (m, 2H), 2.61–2.51 (m, 4H), 2.21 (s, 3H), 2.11 (tt, *J* = 12.0, 3.0 Hz, 2H), 1.99–1.93 (m, 2H), 1.81 (ddd, *J* = 15.0, 7.2, 3.0 Hz, 2H), 1.41 (ddd, *J* = 24.0, 12.0, 3.0 Hz, 2H), 1.23 (t, *J* = 7.2 Hz, 3H), 1.02 (t, *J* = 7.2 Hz, 3H). ^13^C NMR (150 MHz, CDCl_3_): δ 174.4, 142.7, 140.3, 137.9, 137.0, 128.6, 128.5, 128.3, 128.1, 126.3, 126.0, 60.4, 55.8, 53.23, 53.18, 33.8, 30.3, 27.9, 24.0, 19.1, 14.1, 9.3; several signals overlapped. HRMS: *m*/*z* [M + H]^+^ calcd for C_25_H_35_N_2_O, 379.2744; found, 379.2745.

#### *N*-(2-Methylphenyl)-*N*-[1-(2-phenylethyl)-4-piperidinyl]butyramide (**27**)

Oil; yield: 136.6 mg (87%). IR (ATR): 1651 cm^–1^ (CO). ^1^H NMR (600 MHz, CDCl_3_): δ 7.26 (t, *J* = 7.8 Hz, 2H), 7.22 (t, *J* = 7.8 Hz, 1H), 7.17 (td, *J* = 7.8, 1.2 Hz, 4H), 7.10 (dd, *J* = 7.8, 1.2 Hz, 1H), 4.18 (tt, *J* = 12.0, 3.6 Hz, 1H), 2.98–2.96 (m, 2H), 2.75–2.72 (m, 2H), 2.61–2.51 (m, 4H), 2.21 (s, 3H), 2.11 (tt, *J* = 12.0, 3.6 Hz, 2H), 1.98–1.92 (m, 2H), 1.81–1.72 (m, 2H), 1.62–1.53 (m, 2H), 1.40 (ddd, *J* = 24.0, 12.0, 3.6 Hz, 2H), 1.24 (t, *J* = 7.8 Hz, 3H), 0.82 (t, *J* = 7.8 Hz, 3H). ^13^C NMR (150 MHz, CDCl_3_): δ 173.5, 142.7, 140.3, 137.8, 137.0, 128.6, 128.5, 128.3, 128.1, 126.3, 126.0, 60.4, 55.8, 53.22, 53.17, 36.5, 33.7, 30.3, 23.9, 19.1, 18.2, 14.0, 13.9; several signals overlapped. HRMS: *m*/*z* [M + H]^+^ calcd for C_26_H_37_N_2_O, 393.2900; found, 393.2901.

#### *N*-(2-Ethyl-6-methyphenyl)-*N*-[1-(2-phenylethyl)-4-piperidinyl]cyclopropanecarboxamide (**28**)

Oil; yield: 155.5 mg (quant.). IR (ATR): 1646 cm^–1^ (CO). ^1^H NMR (600 MHz, CDCl_3_): δ 7.26 (t, *J* = 7.8 Hz, 2H), 7.21 (*J* = 7.8 Hz, 1H), 7.17 (td, *J* = 7.8, 1.2 Hz, 4H), 7.11 (dd, *J* = 7.8, 1.2 Hz, 1H), 4.19 (tt, *J* = 12.0, 3.6 Hz, 1H), 2.98–2.96 (m, 2H), 2.75–2.72 (m, 2H), 2.66 (dt, *J* = 22.8, 7.8 Hz, 1H), 2.58 (dt, *J* = 22.8, 7.8 Hz, 1H), 2.55–2.52 (m, 2H), 2.27 (s, 3H), 2.09 (tt, *J* = 12.0, 3.0 Hz, 2H), 1.99–1.93 (m, 2H), 1.43 (ddt, *J* = 24.0, 12.0, 3.6 Hz, 2H), 1.23 (t, *J* = 7.8 Hz, 3H), 1.08 (tt, *J* = 7.8, 3.6 Hz, 1H), 0.95 (dd, *J* = 3.6, 2.4 Hz, 2H), 0.57–0.49 (m, 2H). ^13^C NMR (150 MHz, CDCl_3_): δ 174.1, 143.5, 140.3, 137.9, 137.8, 128.6, 128.4, 128.3, 128.1, 126.5, 126.0, 60.4, 56.0, 53.25, 53.21, 33.8, 30.4, 24.5, 19.2, 14.4, 12.5, 8.2, 7.8; several signals overlapped. HRMS: *m*/*z* [M + H]^+^ calcd for C_26_H_35_N_2_O, 391.2744; found, 391.2744.

#### *N*-(2-Ethyl-6-methyphenyl)-*N*-[1-(2-phenylethyl)-4-piperidinyl]cyclopentanecarboxamide (**29**)

Oil: 144.1 mg (88%). IR (ATR): 1646 cm^–1^ (CO). ^1^H NMR (600 MHz, CDCl_3_): δ 7.25 (t, *J* = 7.8 Hz, 2H), 7.22 (t, *J* = 7.8 Hz, 1H), 7.17 (t, *J* = 7.8 Hz, 4H), 7.10 (dd, *J* = 7.8, 1.2 Hz, 1H), 4.18 (tt, *J* = 12.0, 3.6 Hz, 1H), 2.98–2.96 (m, 2H), 2.75–2.72 (m, 2H), 2.62–2.52 (m, 4H), 2.22 (quin, *J* = 8.4 Hz, 1H), 2.22 (s, 3H), 2.10 (ddt, *J* = 12.0, 3.6, 1.8 Hz, 2H), 1.97–1.90 (m, 2H), 1.79–1.68 (m, 4H), 1.58–1.50 (m, 2H), 1.43–1.35 (m, 4H), 1.24 (t, *J* = 7.8 Hz, 3H). ^13^C NMR (150 MHz, CDCl_3_): δ 178.1, 142.9, 140.4, 138.1, 137.2, 128.6, 128.3, 128.0, 126.0, 60.4, 55.9, 53.3, 53.2, 42.6, 33.7, 31.5, 31.4, 30.4, 26.4, 24.0, 19.4, 13.9; several signals overlapped. HRMS: *m*/*z* [M + H]^+^ calcd for C_28_H_39_N_2_O, 419.3057; found, 419.3058.

#### *N*-(2-Ethyl-6-methyphenyl)-*N*-[1-(2-phenylethyl)-4-piperidinyl]cyclohexanecarboxamide (**30**)

White solid; yield: 127.1 mg (74%). MP 88–90 °C. IR (ATR): 1642 cm^–1^ (CO). ^1^H NMR (600 MHz, CDCl_3_): δ 7.25 (t, *J* = 7.8 Hz, 2H), 7.22 (t, *J* = 7.8 Hz, 1H), 7.19 (dd, *J* = 7.8, 1.2 Hz, 1H), 7.16 (dd, *J* = 7.8, 1.2 Hz, 3H), 7.10 (d, *J* = 7.8 Hz, 1H), 4.16 (tt, *J* = 12.0, 3.6 Hz, 1H), 2.98–2.95 (m, 2H), 2.75–2.72 (m, 2H), 2.57 (q, *J* = 7.8 Hz, 2H), 2.54–2.51 (m, 2H), 2.22 (s, 3H), 2.09 (ddd, *J* = 21.0, 12.0, 2.4 Hz, 2H), 1.97–1.94 (m, 1H), 1.88–1.85 (m, 1H), 1.80 (tt, *J* = 12.0, 3.6 Hz, 1H), 1.65–1.63 (m, 2H), 1.58–1.46 (m, 5H), 1.44–1.34 (m, 2H), 1.24 (t, *J* = 7.8 Hz, 3H), 1.17 (ddt, *J* = 25.2, 13.2, 3.6 Hz, 1H), 0.92 (ddt, *J* = 25.2, 13.2, 3.6 Hz, 2H). ^13^C NMR (150 MHz, CDCl_3_): δ 177.3, 142.9, 140.4, 137.9, 137.1, 128.6, 128.4, 128.3, 127.9, 126.1, 126.0, 60.4, 55.8, 53.3, 53.2, 42.9, 33.7, 30.3, 30.2, 29.8, 29.7, 25.6, 25.55, 25.52, 23.9, 19.5, 14.0. HRMS (ESI): *m*/*z* [M + H]^+^ calcd for C_29_H_41_N_2_O, 433.3213; found, 433.3214. Chiral HPLC separation: less polar: [α]_D_^20^ +4.15 (*c* 0.250, CHCl_3_) as 99% ee; more polar: [α]_D_^20^ −4.32 (*c* 0.080, CHCl_3_) as 96% ee. [CHIRALPAK IF, ethanol/hexane = 5:95].

#### *N*-(2-Ethyl-6-methyphenyl)-*N*-[1-(2-phenylethyl)-4-piperidinyl]-2-furancarboxamide (**31**)

White solid; 144.5 mg (87%). MP: 141–142 °C. IR (ATR): 1630 cm^–1^ (CO). ^1^H NMR (600 MHz, CDCl_3_): δ 7.35 (d, *J* = 1.8 Hz, 1H), 7.29–7.25 (m, 3H), 7.20–7.17 (m, 4H), 7.12 (d, *J* = 7.2 Hz, 1H), 6.12 (dd, *J* = 3.6, 1.8 Hz, 1H), 5.27 (dd, *J* = 3.6, 1.8 Hz, 1H), 4.31 (tt, *J* = 12.0, 3.6 Hz, 1H), 3.03–3.01 (m, 2H), 2.78–2.75 (m, 2H), 2.61–2.51 (m, 4H), 2.21 (s, 3H), 2.17 (td, *J* = 12.0, 1.8 Hz, 2H), 2.06–2.01 (m, 2H), 1.59 (dtt, *J* = 24.0, 12.0, 3.6 Hz 2H), 1.07 (t, *J* = 7.8 Hz, 3H). ^13^C NMR (150 MHz, CDCl_3_): δ 159.6, 147.4, 144.4, 143.5, 140.3, 137.9, 137.8, 128.73, 128.67, 128.6, 128.3, 126.5, 126.0, 114.8, 111.0, 60.4, 57.0, 53.2, 33.8, 30.1, 30.0, 24.1, 19.1, 14.0; several signals overlapped. HRMS: *m*/*z* [M + H]^+^ calcd for C_27_H_33_N_2_O_2_, 417.2537; found, 417.2537. Chiral HPLC separation: less polar: [α]_D_^20^ +4.50 (*c* 0.03, CHCl_3_) as 98% ee; more polar: [α]_D_^20^ −4.43 (*c* 0.15, CHCl_3_) as 97% ee. [CHIRALPAK IG, ethanol/hexane = 3:7].

#### *N*-(2-Ethyl-6-methyphenyl)-*N*-[1-(2-phenylethyl)-4-piperidinyl]-3-furancarboxamide (**32**)

White solid; 251.8 mg (63%). MP 39–40 °C. IR (ATR): 1622 cm^–1^ (CO). ^1^H NMR (600 MHz, CDCl_3_): δ 7.31–7.25 (m, 3H), 7.21–7.17 (m, 4H), 7.14 (t, *J* = 1.8 Hz, 1H), 7.13 (d, *J* = 7.2 Hz, 1H), 6.36 (dd, *J* = 1.2, 0.6 Hz, 1H), 6.25 (dd, *J* = 1.8, 0.6 Hz, 1H), 4.29 (tt, *J* = 12.0, 3.6 Hz, 1H), 3.03–3.02 (m, 2H), 2.77–2.75 (m, 2H), 2.60–2.52 (m, 4H), 2.20 (s, 3H), 2.18–2.14 (m, 2H), 2.06–2.03 (m, 2H), 1.60–1.54 (m, 2H), 1.10 (t, *J* = 7.2 Hz, 3H). ^13^C NMR (150 MHz, CDCl_3_): δ 163.5, 144.7, 143.5, 141.9, 140.3, 137.9, 129.0, 128.7, 128.6, 128.3, 126.6, 126.0, 122.6, 111.3, 60.4, 56.9, 53.23, 53.20, 33.7, 30.1, 24.1, 19.1, 13.9; several signals overlapped. HRMS: *m*/*z* [M + H]^+^ calcd for C_27_H_33_N_2_O_2_, 417.2464; found, 417.2538. Chiral HPLC separation: less polar: [α]_D_^20^ +4.93 (*c* 0.12, MeOH) as 97% ee; more polar: [α]_D_^20^ −5.81 (*c* 0.215, MeOH) as 97% ee. [CHIRALPAK IG, ethanol/hexane = 2:8].

#### *N*-(2-Ethyl-6-methyphenyl)-*N*-[1-(2-phenylethyl)-4-piperidinyl]-2-thiophenecarboxamide (**33**)

White solid; 823.2 mg (85%). MP 50–51 °C. IR (ATR): 1608 cm^–1^ (CO). ^1^H NMR (600 MHz, CDCl_3_) δ 7.31 (t, *J* = 7.8 Hz 1H), 7.28–7.25 (m, 2H), 7.25 (dd, *J* = 6.0, 1.2 Hz, 1H), 7.21 (d, *J* = 7.8 Hz, 1H), 7.19–7.17 (m, 3H), 7.13 (d, *J* = 7.8 Hz, 1H), 6.78–6.76 (m, 2H), 4.33 (tt, *J* = 12.0, 3.6 Hz, 1H), 3.03–3.01 (m, 2H), 2.77–2.75 (m, 2H), 2.62–2.53 (m, 4H), 2.23 (s, 3H), 2.16 (td, *J* = 12.0, 1.8 Hz, 2H), 2.06–2.01 (m, 2H), 1.58 (ddd, *J* = 24.0, 12.0, 3.6 Hz, 2H), 1.09 (t, *J* = 7.8 Hz, 3H). ^13^C NMR (150 MHz, CDCl_3_): δ 162.7, 143.9, 140.3, 138.7, 138.4, 137.8, 131.9, 130.9, 129.1, 128.8, 128.7, 128.3, 126.7, 126.6, 126.0, 60.4, 57.5, 53.3, 53.2, 33.8, 30.2, 30.1, 24.1, 19.2, 13.7. HRMS: *m*/*z* [M + H]^+^ calcd for C_27_H_33_N_2_OS, 433.2308; found, 433.2303. Chiral HPLC separation: less polar: [α]_D_^20^ +11.2 (*c* 0.060, MeOH) as 99% ee; more polar: [α]_D_^20^ −10.8 (*c* 0.160, MeOH) as 99% ee. [CHIRALPAK IG, ethanol/hexane = 2:8].

#### 4-Chloro-*N*-(2-ethyl-6-methylphenyl)-*N*-[1-(2-phenylethyl)-4-piperidinyl]benzamide (**34**)

White solid; yield: 118.2 mg (64%). MP: 141–143 °C. IR (ATR): 1635 cm^–1^ (CO). ^1^H NMR (600 MHz, CDCl_3_): δ 7.26 (t, *J* = 7.2 Hz, 2H), 7.18 (dt, *J* = 9.0, 2.4 Hz, 4H), 7.16 (t, *J* = 2.4 Hz, 1H), 7.11 (t, *J* = 7.2 Hz, 1H), 7.07 (dt, *J* = 9.0, 2.4 Hz, 2H), 7.04 (d, *J* = 7.2 Hz, 1H), 6.97 (d, *J* = 7.2 Hz, 1H), 4.16 (tt, *J* = 12.0, 3.9 Hz, 1H), 3.05–3.03 (m, 2H), 2.78–2.76 (m, 2H), 2.63–2.48 (m, 4H), 2.28 (s, 3H), 2.14 (td, *J* = 12.0, 1.8 Hz, 2H), 1.99–1.92 (m, 2H), 1.75 (quin-d, *J* = 12.0, 3.6 Hz, 2H), 1.16 (t, *J* = 7.2 Hz, 3H). ^13^C NMR (150 MHz, CDCl_3_): δ 169.9, 142.3, 140.4, 139.0, 136.7, 135.6, 135.3, 129.0, 128.7, 128.5, 128.3, 128.2, 127.6, 126.1, 126.0, 60.3, 58.1, 53.3, 33.8, 29.8, 24.0, 19.6, 13.6. HRMS: *m*/*z* [M + H]^+^ calcd for C_29_H_34_ClN_2_O, 461.2354; found, 461.2355. Chiral HPLC separation: less polar: [α]_D_^20^ +9.76 (*c* 0.050, CHCl_3_) as >99.9% ee; more polar: [α]_D_^20^ −10.3 (*c* 0.060, CHCl_3_) as 99% ee. [CHIRALPAK IA, 2-propanol/hexane = 3:7].

#### *N*-(2-Isopropyl-6-methyphenyl)-*N*-[1-(2-phenylethyl)-4-piperidinyl]-2-furancarboxamide (**35**)

White solid; 106.0 mg (38%). MP: 94–95 °C. IR (ATR): 1626 cm^–1^ (CO). ^1^H NMR (600 MHz, CDCl_3_): δ 7.33 (dd, *J* = 1.8, 0.6 Hz, 1H), 7.31 (t, *J* = 7.8 Hz, 1H), 7.27–7.25 (m, 2H), 7.21 (dd, *J* = 7.8, 1.2 Hz, 1H), 7.19–7.17 (m, 3H), 7.12 (ddd, *J* = 7.8, 1.8, 0.6 Hz, 1H), 6.12 (dd, *J* = 3.6, 1.8 Hz, 1H), 5.31 (dd, *J* = 3.6, 0.6 Hz, 1H), 4.24 (tt, *J* = 12.0, 3.6 Hz, 1H), 3.05–3.01 (m, 3H), 2.77–2.74 (m, 2H), 2.58–2.55 (m, 2H), 2.24 (s, 3H), 2.16 (tdd, *J* = 12.0, 6.0, 2.4 Hz, 2H), 2.08–2.01 (m, 2H), 1.64 (ddd, *J* = 24.0, 12.0, 3.6 Hz, 2H), 1.25 (d, *J* = 7.2 Hz, 3H), 0.82 (d, *J* = 7.2 Hz, 3H). ^13^C NMR (150 MHz, CDCl_3_): δ 159.6, 148.3, 147.4, 144.2, 140.5, 137.7, 137.1, 128.9, 128.7, 128.4, 128.3, 125.9, 124.9, 115.2, 110.9, 60.3, 57.6, 53.3, 53.2, 33.8, 30.00, 29.98, 28.2, 24.5, 24.2, 19.3. HRMS: *m*/*z* [M + H]^+^ calcd for C_28_H_35_N_2_O_2_, 431.2693; found, 431.2693. Chiral HPLC separation: less polar: [α]_D_^20^ +44.48 (*c* 1.00, CHCl_3_) as 99.9% ee; more polar: [α]_D_^20^ −46.18 (*c* 1.00, CHCl_3_) as 99.9% ee. [CHIRALPAK IG, ethanol/hexane = 3:7].

#### *N*-(2-Fluoro-6-methyphenyl)-*N*-[1-(2-phenylethyl)-4-piperidinyl]propionamide (**36**)

Oil; yield: 77.4 mg (86%). IR (ATR): 1660 cm^–1^ (CO). ^1^H NMR (600 MHz, CDCl_3_): δ 7.27–7.21 (m, 3H), 7.19–715 (m, 3H), 7.07 (d, *J* = 7.8 Hz, 1H), 6.98 (t, *J* = 7.8 Hz, 1H), 4.57 (tt, *J* = 12.0, 4.2 Hz, 1H), 3.01–2.97 (m, 2H), 2.75–2.71 (m, 2H), 2.55–2.52 (m, 2H), 2.26 (s, 3H), 2.15–2.11 (m, 2H), 2.03–2.01 (m, 1H), 1.92–1.79 (m, 3H), 1.50 (ddt, *J* = 24.0, 12.0, 4.2 Hz, 1H), 1.40 (ddd, *J* = 24.0, 12.0, 4.2 Hz, 1H), 1.03 (t, *J* = 7.8 Hz, 3H). ^13^C NMR (150 MHz, CDCl_3_): δ 174.0, 159.6 (d, ^1^*J*_C–F_ = 245.7 Hz), 140.3, 140.0, 129.3 (d, ^1^*J*_C–F_ = 8.7 Hz), 128.6, 128.3, 126.7, 126.6, 126.5, 126.0, 113.9 (d, ^1^*J*_C–F_ = 21.6 Hz), 60.5, 54.7, 53.2, 53.1, 33.8, 30.0, 29.7, 27.7, 18.3, 9.2; several signals overlapped. HRMS: *m*/*z* [M + H]^+^ calcd for C_23_H_30_FN_2_O, 369.2337; found, 369.2338.

#### *N*-(2-Chlorophenyl)-*N*-[1-(2-phenylethyl)-4-piperidinyl]propionamide (**37**)

White solid; yield: 46.1 mg (31%). MP: 83–84 °C. IR (ATR): 1659 cm^–1^ (CO). ^1^H NMR (600 MHz, CDCl_3_): δ 7.50–7.49 (m, 1H), 7.33–7.29 (m, 2H), 7.26 (t, *J* = 7.8 Hz, 2H), 7.21–7.19 (m, 1H), 7.17–7.15 (m, 3H), 4.66 (tt, *J* = 12.0, 4.2 Hz, 1H), 3.04–3.01 (m, 1H), 2.97–2.94 (m, 1H), 2.75–2.72 (m, 2H), 2.55–2.52 (m, 2H), 2.18 (td, *J* = 12.0, 1.8 Hz, 1H), 2.12 (td, *J* = 12.0, 1.8 Hz, 1H), 2.02–2.00 (m, 1H), 2.01 (dq, *J* = 16.8, 7.8 Hz, 1H), 1.93–1.90 (m, 1H), 1.86 (dq, *J* = 16.8, 7.8 Hz, 1H), 1.59 (ddd, *J* = 24.0, 12.0, 4.2 Hz, 1H), 1.26 (ddd, *J* = 24.0, 12.0, 4.2 Hz, 1H), 1.04 (t, *J* = 7.8 Hz, 3H). ^13^C NMR (150 MHz, CDCl_3_): δ 173.5, 140.2, 137.1, 135.1, 132.1, 130.7, 129.6, 128.6, 128.4, 127.7, 126.0, 60.5, 53.8, 53.13, 53.05, 33.8, 31.2, 29.1, 28.0, 9.3. HRMS: *m*/*z* [M + H]^+^ calcd for C_22_H_28_ClN_2_O, 371.1885; found, 371.1886.

#### *N*-(2-Chloro-6-fluorophenyl)-*N*-[1-(2-phenylethyl)-4-piperidinyl]propionamide (**38**)

Oil; yield: 28.8 mg (63%). IR (ATR): 1666 cm^–1^ (CO). ^1^H NMR (600 MHz, CDCl_3_): δ 7.32–7.29 (m, 2H), 7.28–7.25 (m, 2H), 7.19–7.15 (m, 3H), 7.13–7.10 (m, 1H), 4.60 (tt, *J* = 12.0, 3.6 Hz, 1H), 3.02–2.98 (m, 2H), 2.76–2.73 (m, 2H), 2.55–2.53 (m, 2H), 2.13 (ddd, *J* = 24.0, 12.0, 3.6 Hz, 2H), 2.06–2.03 (m, 1H), 2.01–1.97 (m, 1H), 1.93 (dq, *J* = 16.2, 7.2 Hz, 1H), 1.87 (dq, *J* = 16.2, 7.2 Hz, 1H), 1.52 (ddt, *J* = 24.0, 12.0, 3.6 Hz, 1H), 1.42 (ddd, *J* = 24.0, 12.0, 3.6 Hz, 1H), 1.05 (t, *J* = 7.2 Hz, 3H). ^13^C NMR (150 MHz, CDCl_3_): δ 173.5, 160.3 (d, ^1^*J*_C–F_ = 248.6 Hz), 140.2, 136.7, 130.1 (d, ^1^*J*_C–F_ = 8.6 Hz), 128.6, 128.4, 126.4, 126.3, 126.1, 126.06, 126.04, 126.01, 115.4 (d, ^1^*J*_C–F_ = 21.6 Hz), 60.4, 54.9, 53.2, 53.1, 33.8, 29.9, 29.6, 27.7, 9.1; some C–F couplings are not described and several signals overlapped. HRMS: *m*/*z* [M + H]^+^ calcd for C_22_H_27_ClFN_2_O: 389.1790; found: 389.1791.

### Single-Crystal X-ray Analysis

The crystal structures of compound **34** were obtained *via* single-crystal X-ray analysis. The typical crystal data for **34** are shown in the Supporting Information.

### Stereochemical Stability of **8**, **23**, **24**, and **30–35**

The racemization of **8**, **23**, **24**, and **30–35** at 37, 80, or 100 °C in toluene was examined as previously described.^35^ The method for determining the Δ*G*^⧧^ is shown in the Supporting Information.

### *In Vitro* MOR Assays

MOR assays were conducted to determine the agonistic/antagonistic efficacy of the compounds against the MOR. A stable MOR-expressing cell strain (MOR-CHO) was generated by transfecting a human MOR (CAT no. RG202243, OriGene Technologies, Inc., Rockville, MD, USA) and a human Gα16 (CAT no. RC202243, OriGene Technologies, Inc.) into the CHO cell line (CCL-61, ATCC) using the 4D-Nucleofector system (Lonza, Basel, Switzerland). The intracellular calcium concentration was measured. Cells were seeded (5 × 10^4^ cells/well) into 96-well black clear bottom plates (Greiner, Frickenhausen, Germany) and cultured at 37 °C under 5.0% CO_2_ conditions. After 24 h, the medium was replaced with component B (1× Hank’s balanced salt solution + 20 mM HEPES buffer, pH 7.4) with 2 mM probenecid, and a Ca^2+^ indicator dye (FLIPR Calcium 4 Assay kit R8141, Molecular Devices, San Jose, CA, USA) was added to the wells. After 1 h, the compounds investigated in component B were added to the wells.

To determine the MOR antagonistic effects of the test compounds, we examined the pretreatment effects of the compounds or the opioid antagonist naloxone on the fentanyl-mediated increase in calcium utilization. Nine concentrations (0.0000064, 0.000032, 0.00016, 0.0008, 0.004, 0.02, 0.1, 0.5, and 2.5 μM) of the test compounds and naloxone were added to the culture plates; after 10 min of incubation (at 37 °C), the effect of fentanyl (0.025 μM) was assessed. Fentanyl-analogue-induced fluorescence changes were measured *via* a Flexstation III and the SOFTMAX PRO software (Molecular Devices). The following measurement conditions were maintained: excitation wavelength: 485 nm, fluorescence wavelength: 525 nm, and cutoff value: 515 nm. The obtained values were calculated to determine the EC_50_ or IC_50_ values using GraphPad Prism 9 (GraphPad Software Inc., San Diego, CA, USA).

### *In Vitro* Competitive Radioligand Binding Assay

The *in vitro* binding affinities of the fentanyl analogues to MOR were screened at Eurofins Panlabs Discovery Services Taiwan, Ltd. (New Taipei City, Taiwan). Human recombinant MORs expressed in CHO-K1 cells were used in modified Tris-HCl buffer pH 7.4. An 11 μg aliquot was incubated with 0.6 nM [^3^H]-diprenorphine for 60 min at 25 °C (membrane proteins may vary from lot to lot, and the concentration can be adjusted if necessary.). Nonspecific binding was estimated in the presence of 10 μM naloxone. After incubation, the samples were rapidly filtrated through buffer-soaked glass fiber filters under vacuum and rinsed several times with an ice-cold buffer in a 48- or 96-sample cell harvester. The experimental conditions are outlined in Supporting Information Table S1. The residues were evaluated for radioactivity in a scintillation counter using a scintillation cocktail. The IC_50_ values (the concentration causing half-maximal inhibition of control-specific binding) were computed from the displacement data using a nonlinear, least-squares regression analysis (MathIQ: ID Business Solutions, Ltd., Woking, UK). The concentration and the Eurofins Cerep’s historical value for the dissociation constant of the radioligand were deduced. The relative affinities (*K*i) were derived from the IC_50_ values using the Cheng–Prusoff equation.^36^

### *In Vivo* Ethopharmacological Experiments

All animal experiments were approved by the Experimental Animal Care and Use Committee of the National Institute of Neuroscience, National Center of Neurology and Psychiatry (#2021001R4). Specific pathogen-free, 4 to 5 week-old male mice were supplied by CLEA Japan (Tokyo, Japan). The mice were of the well-characterized outbred strain Jcl/ICR. At the commencement of the experiment, the mice weighed 20–25 g. The animals were housed in sterile and ventilated individual cages, exposed to 12/12 h light/dark cycles with dawn/dusk phases, and provided sterilized food and water *ad libitum*. The mice were segregated into groups of 12 and treated with either the enantiomer of compound **31**, “(+)-**31**” (2 mg/kg), or naloxone (2 mg/kg) 30 min before morphine (10 mg/kg) administration. The control group was injected with saline, and all drugs were administered intraperitoneally. Test articles and comparators were reconstituted and diluted in 0.9% saline (OTSUKA, Tokyo, Japan). After allowing adaptation to the environment for 3 h, the locomotor activity under the influence of drugs was measured for 120 min using an animal-movement analysis system (ACTIMO-100 Bio Research Center, Tokyo, Japan).

### Calculation of DFT-Based ECD

Compound a*R*-**35**′ was built on Spartan’20 (Wave function, Inc., Irvine, CA, USA) and subjected to a conformational search with molecular mechanics MMFF94,^37^ with which the program is equipped, using the threshold at 40 kJ/mol from the global minimum conformer and successive conformer narrowing based on HF/3-21G (threshold: 40 kJ/mol) and ωB97X-D/6-31G* (threshold: 10 kJ/mol) to yield 23 stable conformers. Then, the free energies based on ωB97X-D/6-31G* and B3LYP/6-31G*^38^ of the remaining conformers were calculated with vibrational analyses (Supporting Information Tables S2–S4). ECD spectral calculations were performed with CAM-B3LYP/def2-TZVP^29^ for the stable conformers within 8 kJ/mol of free energy by either ωB97X-D/6-31G* or B3LYP/6-31G* (Supporting Information Table S5). The UV and ECD spectra were constructed based on the frequencies and oscillator and rotary strengths acquired by implementing the NORMDIST function in Microsoft Excel (Microsoft Corp., Redmond, WA, USA). The widths and signal intensities were adjusted to appropriately reproduce the spectra. The UV and ECD spectra of the individual conformers were averaged considering the Boltzmann distribution. The wavelengths of the UV and ECD spectra were calibrated to align with the experimental UV spectra (+27 nm), and the same value was used to correct the wavelength of the ECD spectra. The rotatory strength values of a*R*-**35**′ were multiplied by −1 to create the ECD spectra of compound a*S*-**35**′. The obtained oscillator and rotatory strengths and calculation details are described in Supporting Information Table S5.

### Molecular Docking Studies

A 3D model structure of the target protein was engineered for the docking analysis of the ligand and MOR (Protein Data Bank: 5C1M).^32^ First, the chemical structure of compound **31** was generated, and a simplified molecular input line entry system (SMILES) file was created that was used as input in OpenBabel’s Confab method to generate 488 protein conformers.^39^ After performing structural optimization calculations at the HF/STO-3G level using Gaussian 16,^40^ the conformers that exceeded an energy threshold of 10 kJ/mol compared to the most stable conformer were excluded. The relaxed structures and the most stable conformer were classified into 50 clusters based on their root-mean-square deviation value (Supporting Information Table S6). The AutoDock Vina-based docking tool, integrated into Yet Another Scientific Artificial Reality Application (YASARA) structure, was used to perform rigid docking of the 50 conformers to the orthosteric site of the MOR.^41^ Candidate poses with docking scores <8.0 kcal/mol were excluded, yielding 23 binding poses.

### MD Simulation

Membrane protein MD simulations were performed for the 23 candidate poses using the YASARA structure to validate their binding poses.^41^ The force field used was Amber14, which conducts 10 ns of MD simulations in the fast mode (captures snapshots at 250 ps intervals). Using the boundary elements method with the YASARA2 force field, the average binding energies were obtained from snapshots of the second half of the entire MD simulation (Supporting Information Table S7). Subsequently, MD simulations were performed for the highest binding energies for a*S* and a*R* and prolonged to 100 ns under the same conditions. Finally, pharmacophore analyses of the 100 ns MD trajectories were conducted using LigandScout.^42^

## Supporting Information Available

NOESY spectra of compound **17**, X-ray structural analysis of compound **34**, stereochemical stability of compounds **8**, **23**, **24**, and **30–35**, *in vitro* MOR assays, *in vitro* competitive radioligand binding assay, calculation of DFT-based ECD spectra, docking study, MD simulation, ^1^H, ^13^C, 2D NMR, and HRMS spectra, and HPLC chromatograms of target compounds (PDF)
Predicted a*R*-**31** binding configurations in the MOR (PDB)
Predicted a*S*-**31** binding configurations in the MOR (PDB)
X-ray structural analysis of compound **34** (CIF)
SMILES molecular formula strings (CSV)

## Supplementary Material

jm4c00935_si_001.pdf

jm4c00935_si_003.pdb

jm4c00935_si_004.pdb

jm4c00935_si_005.cif

## Abbreviations

Ac: acetyl
Ar: aryl
ATR: attenuated total reflection
CHO: Chinese hamster ovary
DMF: *N*,*N*-dimethylformamide
ECD: electronic circular dichroism
Et: ethyl
*G*: Gibbs free energy
HPLC: high-performance liquid chromatography
HRMS: high-resolution mass spectroscopy
*i*-Pr: isopropyl
IR: infrared spectroscopy
M: mol/L
Me: methyl
MRP: morphine
NLX: naloxone
*n*-Pr: *n*-propyl
N.C.: not calculated
NOESY: nuclear Overhauser effect spectroscopy
ppm: parts per million
NMR: nuclear magnetic resonance
PDB: Protein Data Bank
UV: ultraviolet
Tyr: tyrosine
Met: methionine
Ile: isoleucine
Trp: tryptophan
Val: valine
