# Supplementary material for: Fentanyl-Type Antagonist of the μ-Opioid Receptor: Important Role of Axial Chirality in the Active Conformation
Source: J Med Chem. 2024 Jun 13;67(12):10447–63. doi: 10.1021/acs.jmedchem.4c00935 (PMC11215721; doi:10.1021/acs.jmedchem.4c00935)
Supplement: Supplementary file 1 — jm4c00935_si_001.pdf [file jm4c00935_si_001.pdf]

SUPPORTING INFORMATION

Fentanyl-type antagonist of the μ-opioid receptor: Important role of axial chirality in the active conformation

Hironobu Arita[1], Ryoko Tanaka[1], Shuntaro Kikukawa[1], Tsukasa Tomizawa[1], Haruka Sakata[1], Masahiko Funada[2], Kenichi Tomiyama[3], Masaru Hashimoto[4], Tomohiko Tasaka[5], Hidetsugu Tabata[6], Kayo Nakamura[1], Kosho Makino[7], Tetsuta Oshitari[6], Hideaki Natsugari[8], Hideyo Takahashi[1]*

[1]Faculty of Pharmaceutical Sciences, Tokyo University of Science, Noda-shi, Chiba 278-8510, Japan.

[2]Faculty of Pharmaceutical Sciences, Shonan University of Medical Sciences, Yokohama-shi, Kanagawa 224-0806, Japan.

[3]Section of Addictive Drug Research, Department of Drug Dependence Research, National Institute of Mental Health, National Center of Neurology and Psychiatry, Kodaira-shi, Tokyo 187-8533, Japan.

[4]Faculty of Agriculture and Life Science, Hirosaki University, Hirosaki-shi, Aomori 036-8561, Japan.

[5]Affinity Science Corp., Shinagawa-ku, Tokyo 141-0031, Japan.

[6]Faculty of Pharma Sciences, Teikyo University, Itabashi-ku, Tokyo 173-8605, Japan.

[7]Research Institute of Pharmaceutical Sciences, Musashino University, Nishitokyo-shi, Tokyo 202-8585, Japan.

[8]Graduate School of Pharmaceutical Science, The University of Tokyo, Bunkyo-ku, Tokyo 113-0033, Japan.

* Corresponding author. Hideyo Takahashi, E-mail: hide-tak@rs.tus.ac.jp

Table of Contents

1. **NOESY spectra of compound 17 for characterization of the *E* and *Z* Isomers.........S3**
2. **Typical crystal data of X−ray structure analysis of compound 34 ....S5**
3. **Stereochemical stability of compounds 8, 23, 24, 30–35...S6**
4. ***In vitro* μ−opioid receptor (MOR) assays (agonistic activity).....................................S15**
5. ***In vitro* μ−opioid receptor (MOR) assays (antagonistic activity)..............................S19**
6. ***In vitro* competitive radioligand binding assay S20**
7. **Calculation of DFT-based ECD spectra (conformational search)...............................S22**
8. **Docking study and MD simulation...S44**
9. **^{1}H-, ^{13}C-, 2D-NMR and HRMS Spectra S48**
10. **HPLC chromatograms for target compounds S121**

1. NOESY spectra of compound 17 for characterization of the *E* and *Z* Isomers

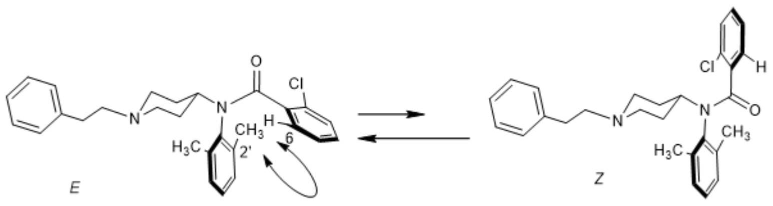

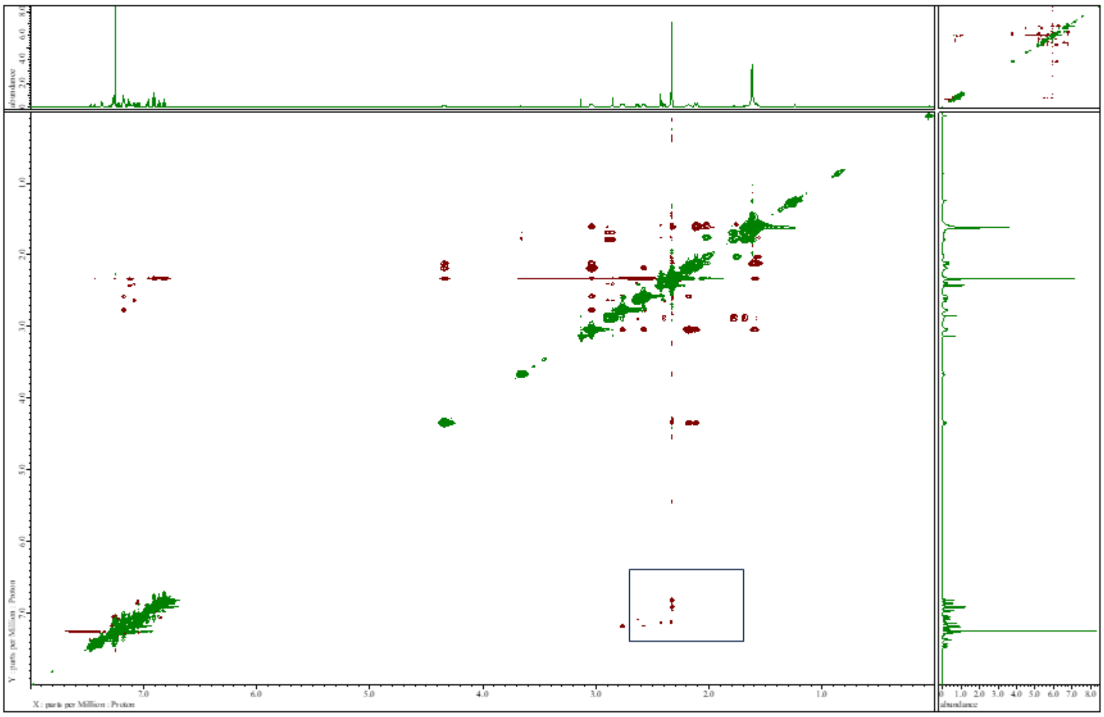

Figure S1. NOESY spectrum of compound **17**

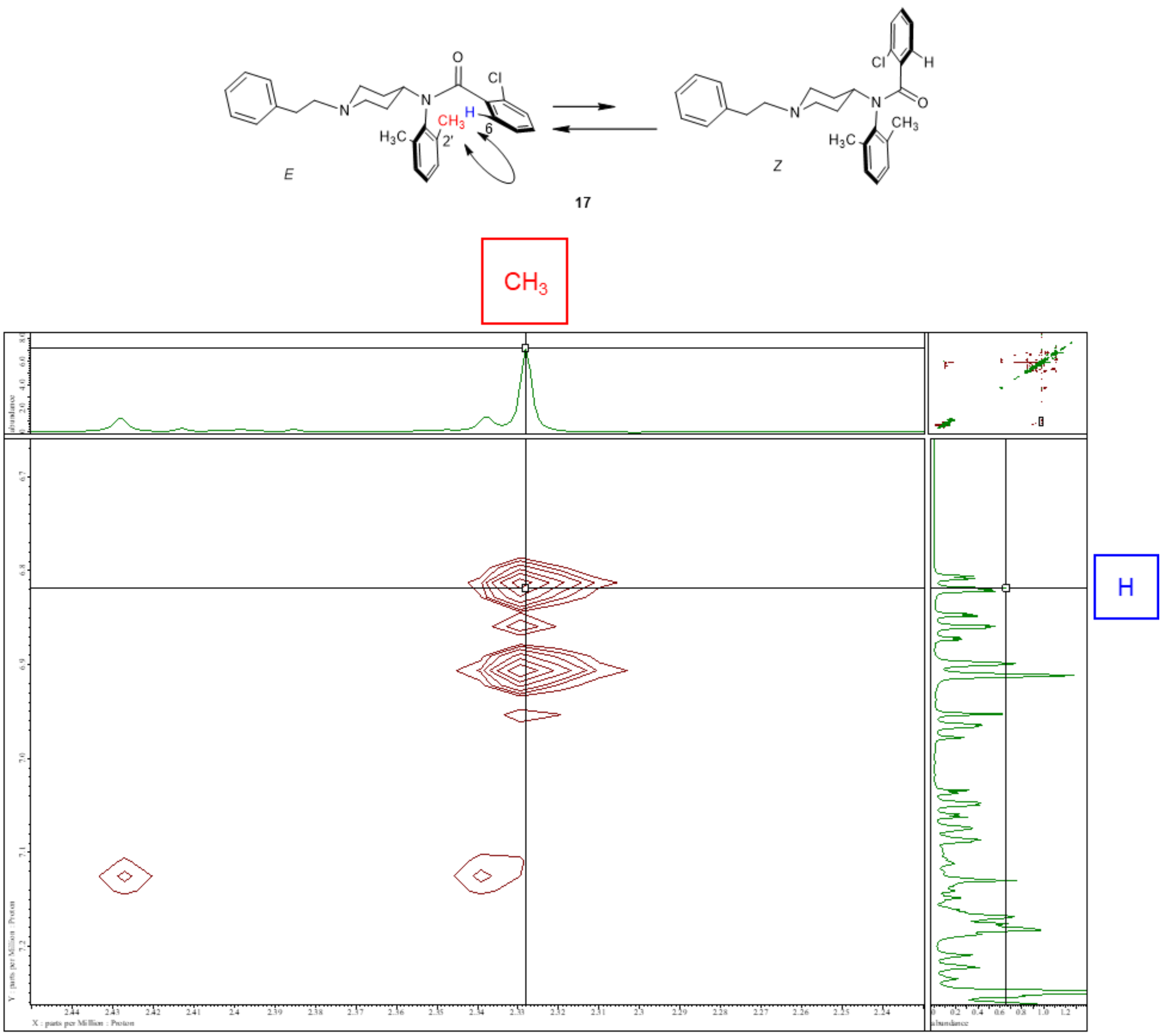

Figure S2. Enlarged NOESY spectrum of compound **17**

2. Typical crystal data of X–ray structure analysis of compound 34

The CIF data is available via the Internet at http://pubs.acs.org.

Crystal data of 34 (CCDC 2244304):

$C_{29}H_{33}ClN_2O$: mp 141–143 °C, Mr = 461.05, CuKα (λ = 1.54187 Å), triclinic, P−1 (#2), colorless, block, 0.150 × 0.100 × 0.070 mm, crystal dimensions a = 10.3654(4) Å, b = 11.0552(4) Å, c = 12.0292(4) Å, α = 95.2013(18)°, β = 109.6997(17)°, γ = 102.6380(17)°, T = 173 K, Z = 2, V = 1245.88(8) Å^3, D_{calc} = 1.229 g/cm^3, μCuKα = 15.285 cm^{-1}, F_{000} = 492.00, R_{int} = 0.0353, R_1 = 0.0652, wR_2 = 0.1785.

3. Stereochemical stability of compounds 8, 23, 24, 30–35

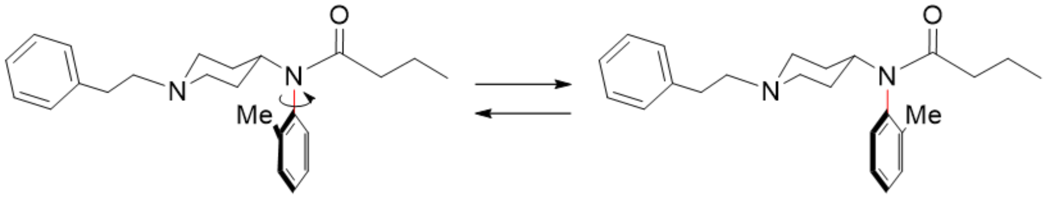

8

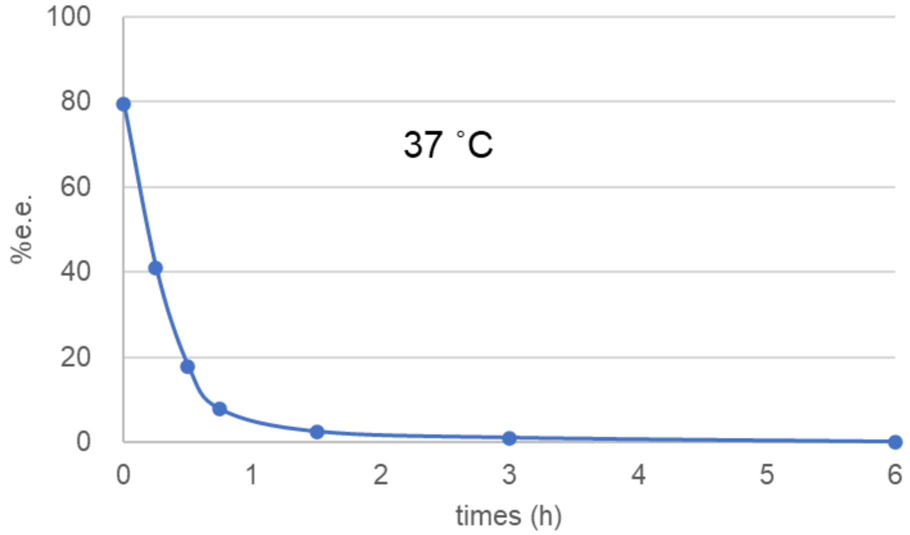

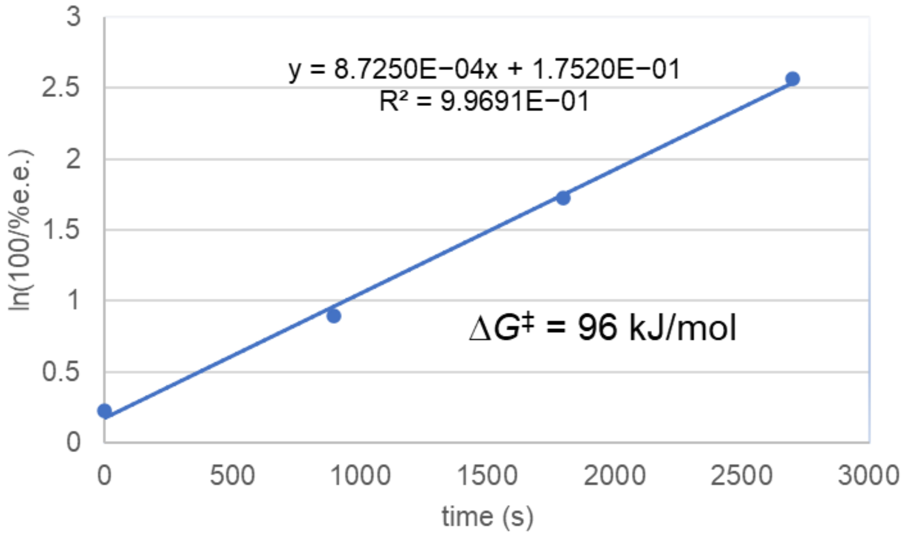

T = 310 K

k = 1/2slope = 4.3625 E−04

K = kh/kT = 6.7520 E−17

$\Delta G^{\ddagger}$ = −RTlnK = 96.0 kJ/mol

Figure S3. Measurement of rotation barriers of atropisomers of compound **8**.

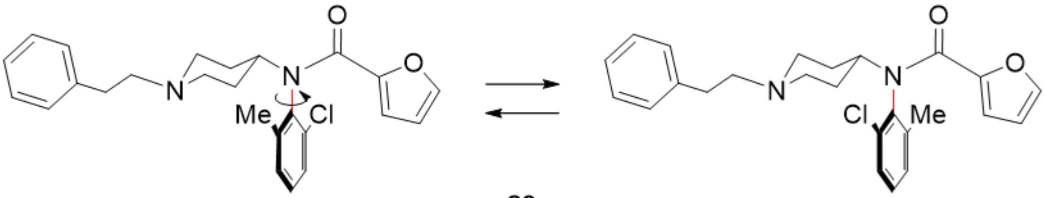

23

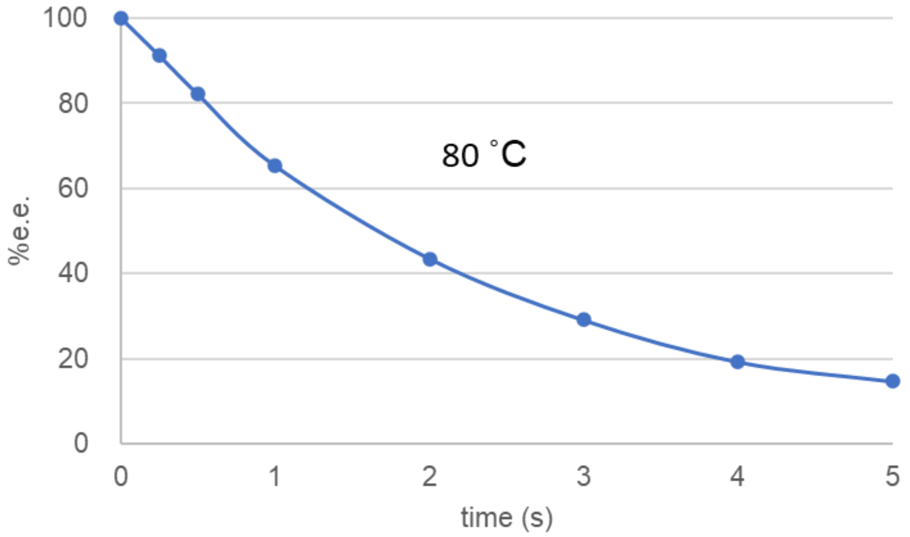

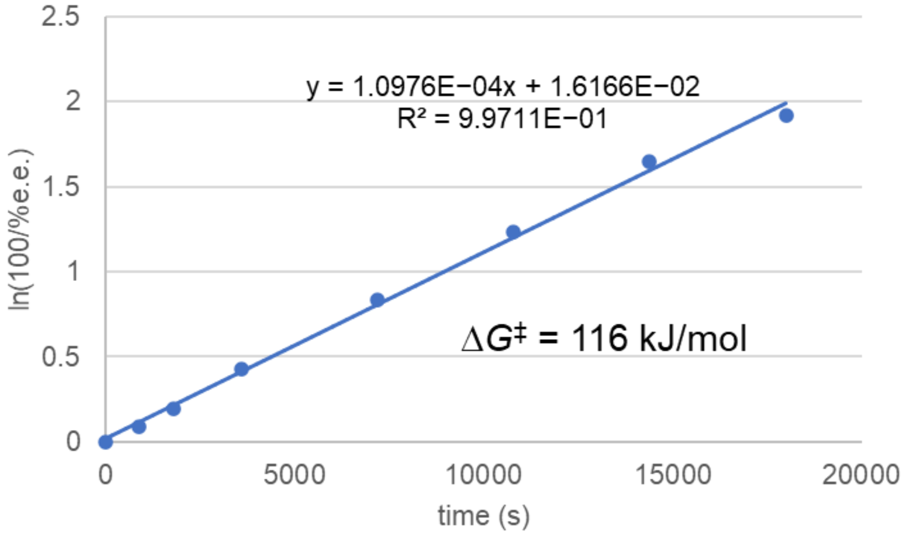

T = 353 K
k = 1/2slope = 5.4878 E−05
K = kh/*k*T = 7.4590 E−18
$\Delta G^{\ddagger}$ = −RTlnK = 115.7 kJ/mol

Figure S4. Measurement of rotation barriers of atropisomers of compound **23**.

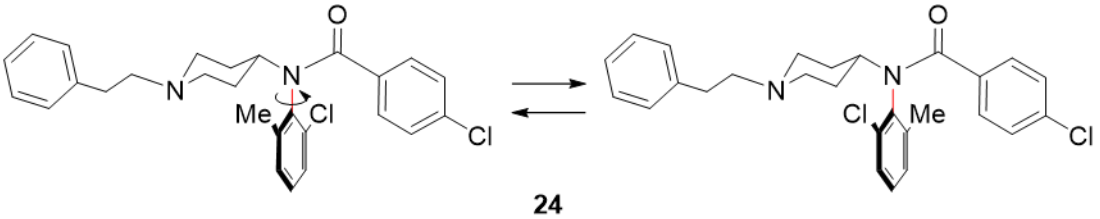

24

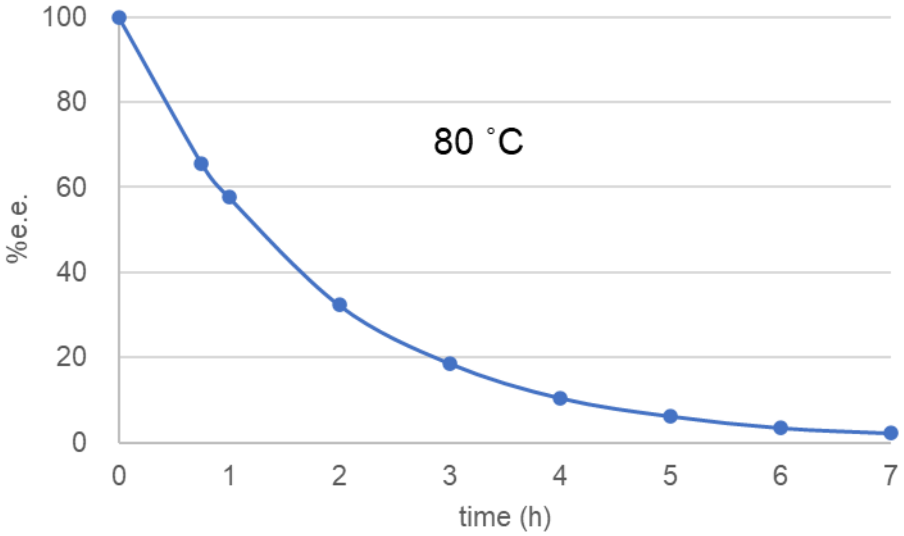

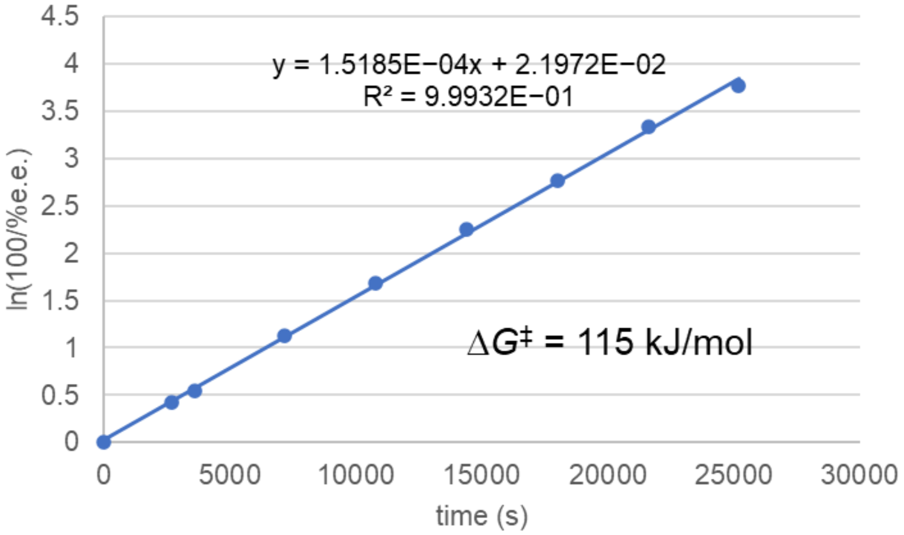

T = 353 K
k = 1/2slope = 7.5926 E−05
K = kh/*k*T = 1.0320 E−17
$\Delta G^{\ddagger}$ = −RTlnK = 114.8 kJ/mol

Figure S5. Measurement of rotation barriers of atropisomers of compound **24**.

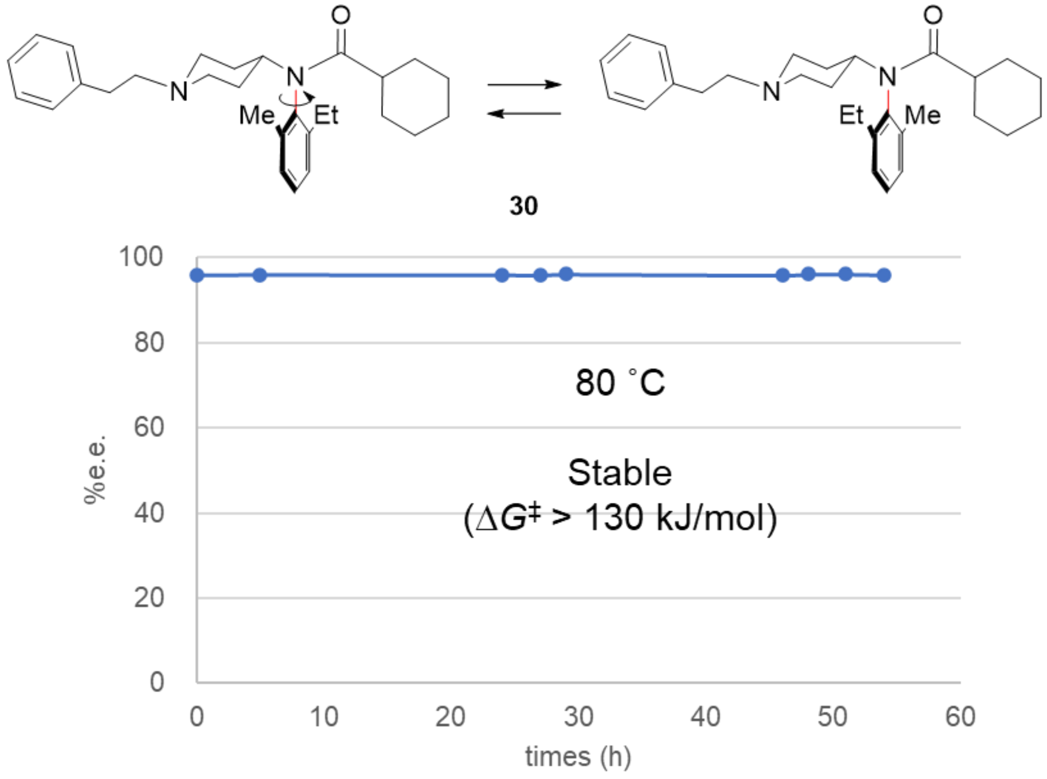

Figure S6. Measurement of rotation barriers of atropisomers of compound **30**.

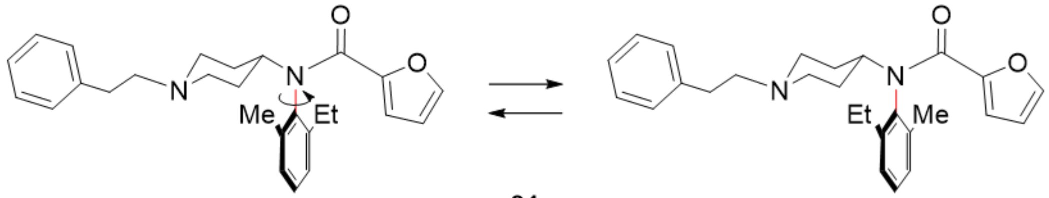

31

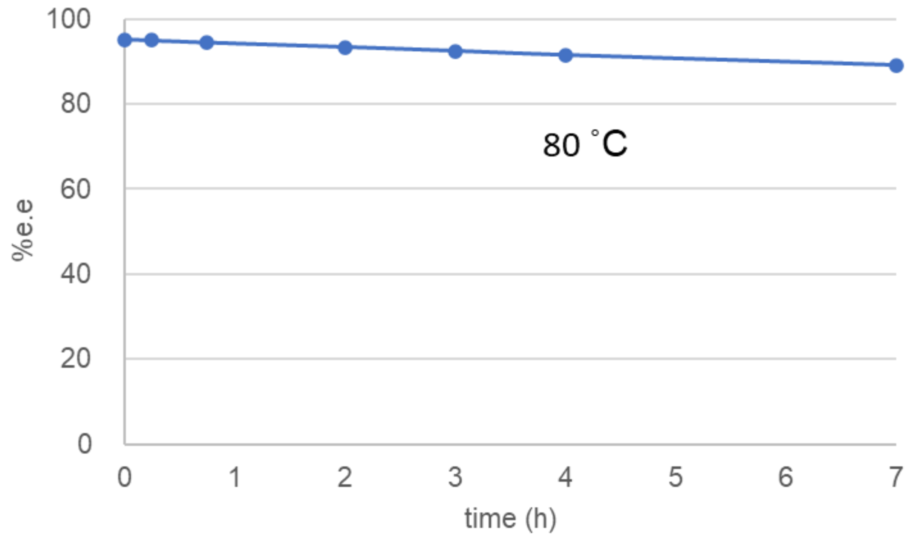

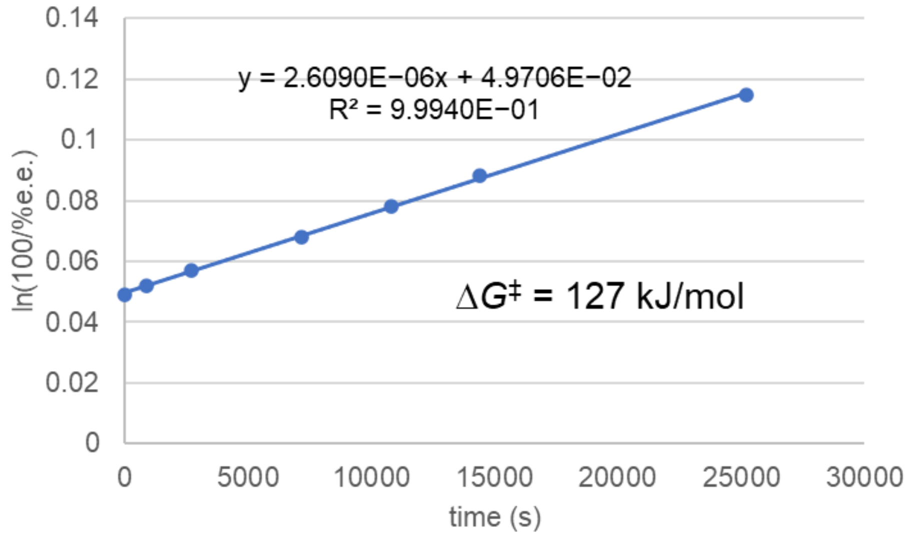

T = 353 K

k = 1/2slope = 1.3045 E−06

K = kh/*k*T = 1.7731E−19

$\Delta G^{\ddagger}$ = −RTlnK = 126.7 kJ/mol

Figure S7. Measurement of rotation barriers of atropisomers of compound **31**.

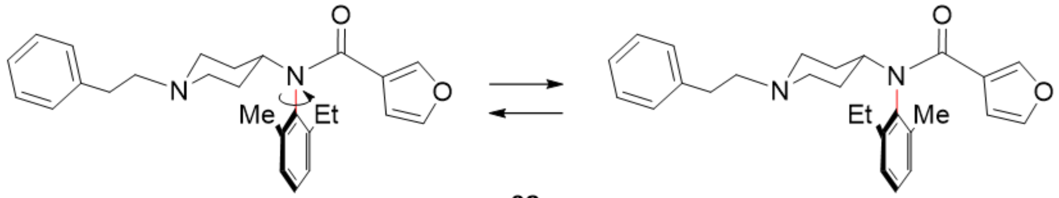

32

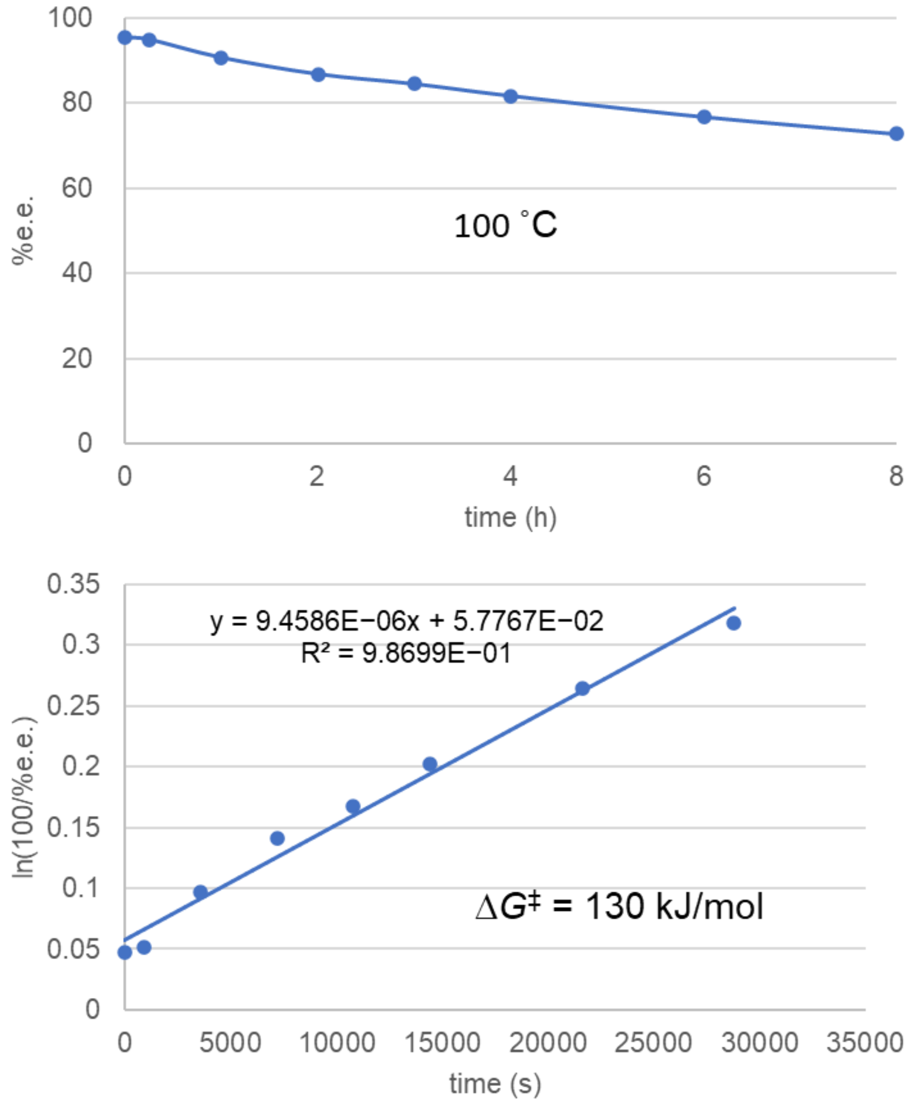

T = 373 K
k = 1/2slope = 4.7293 E−06
K = kh/kT = 6.0834E−19
$\Delta G^{\ddagger}$ = −RTlnK = 130.1 kJ/mol

Figure S8. Measurement of rotation barriers of atropisomers of compound **32**.

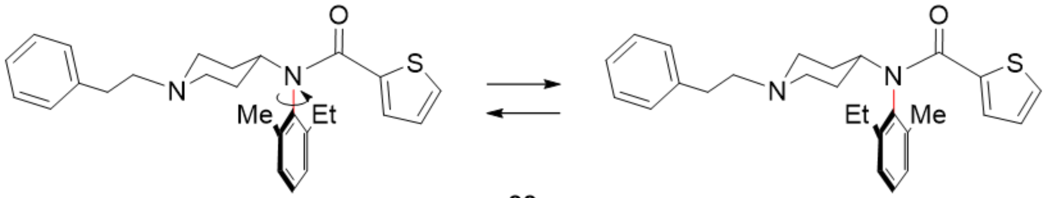

33

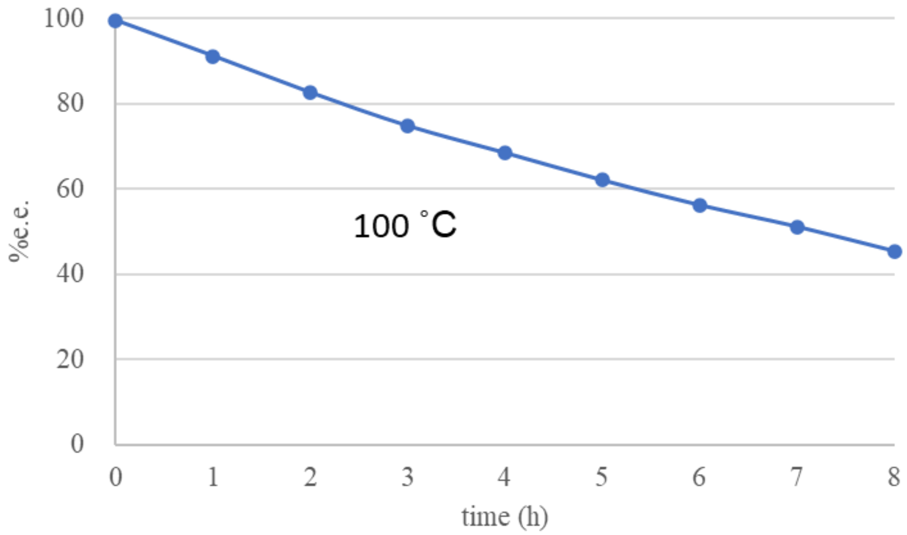

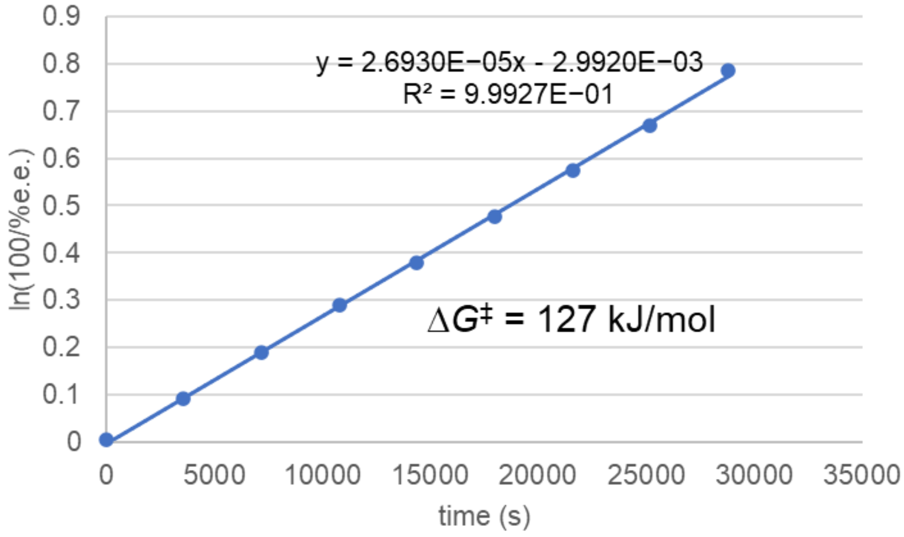

T = 373 K
k = 1/2slope = 1.3465E−05
K = kh/*k*T = 1.7320E−18
Δ*G*‡ = −RTlnK = 126.8 kJ/mol

Figure S9. Measurement of rotation barriers of atropisomers of compound **33**.

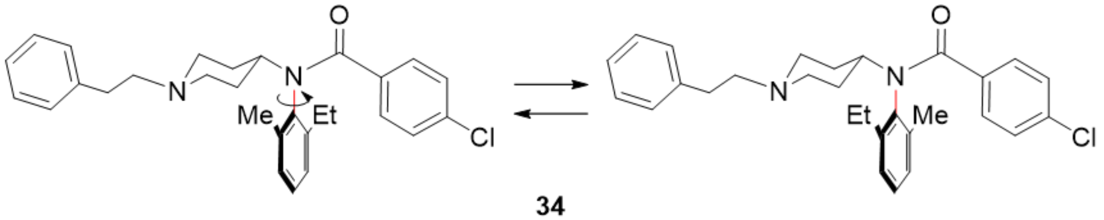

34

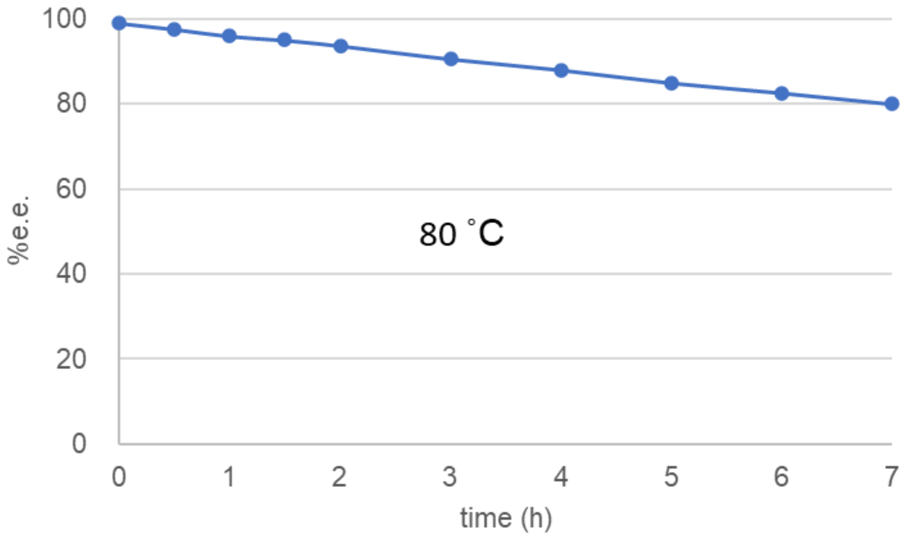

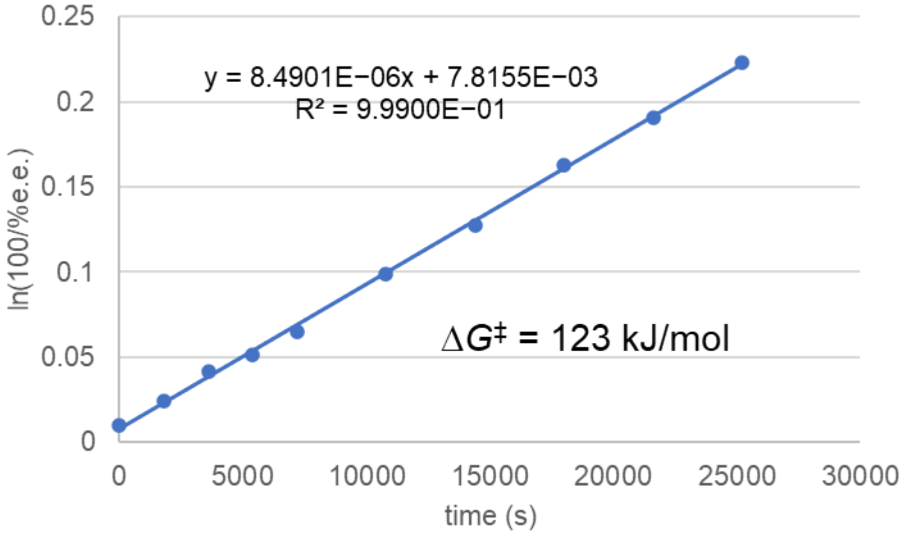

T = 353 K
k = 1/2slope = 4.2451 E−05
K = kh/*k*T = 5.7699 E−19
$\Delta G^{\ddagger}$ = −RTlnK = 123.3 kJ/mol

Figure S10. Measurement of rotation barriers of atropisomers of compound **34**.

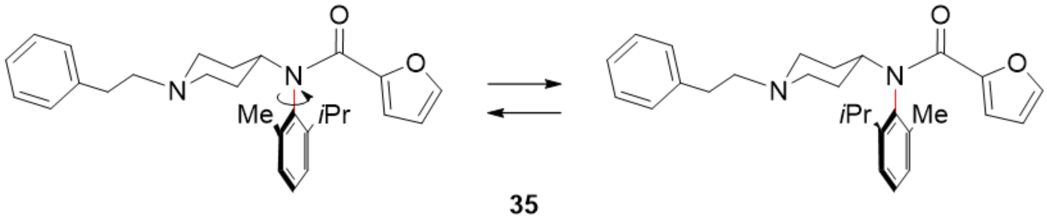

35

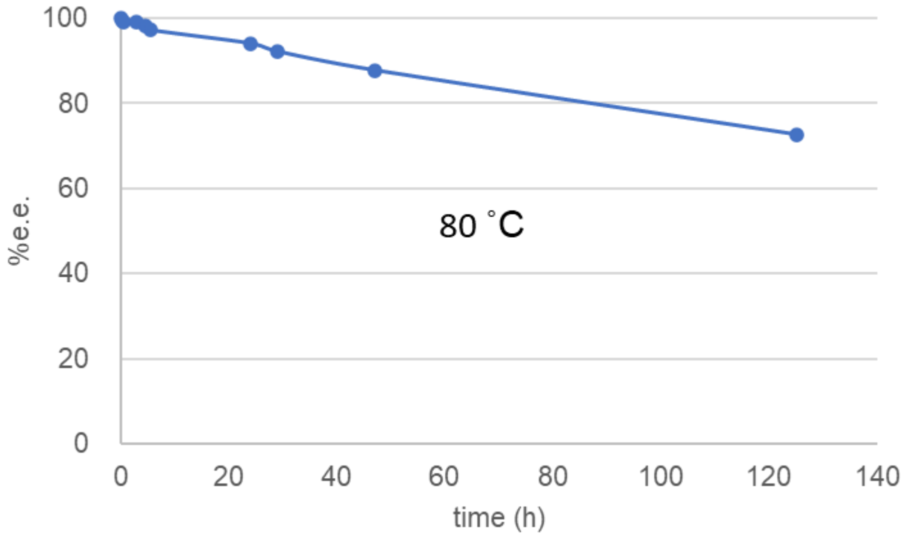

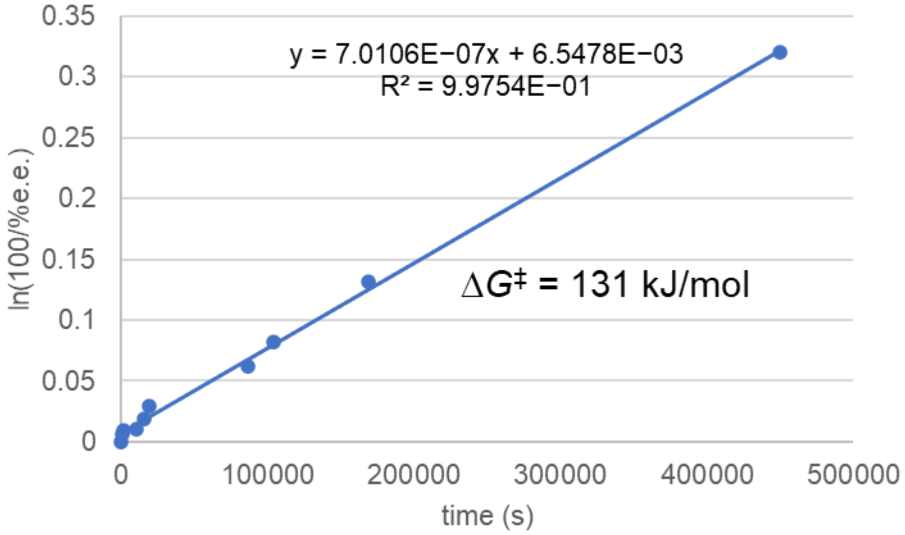

T = 353 K
k = 1/2slope = 3.5053E−07
K = kh/*k*T = 4.7644E−20
Δ*G*‡ = −RTlnK = 130.6 kJ/mol

Figure S11. Measurement of rotation barriers of atropisomers of compound **35**.

4. *In vitro* μ–opioid receptor (MOR) assays (agonistic activity)

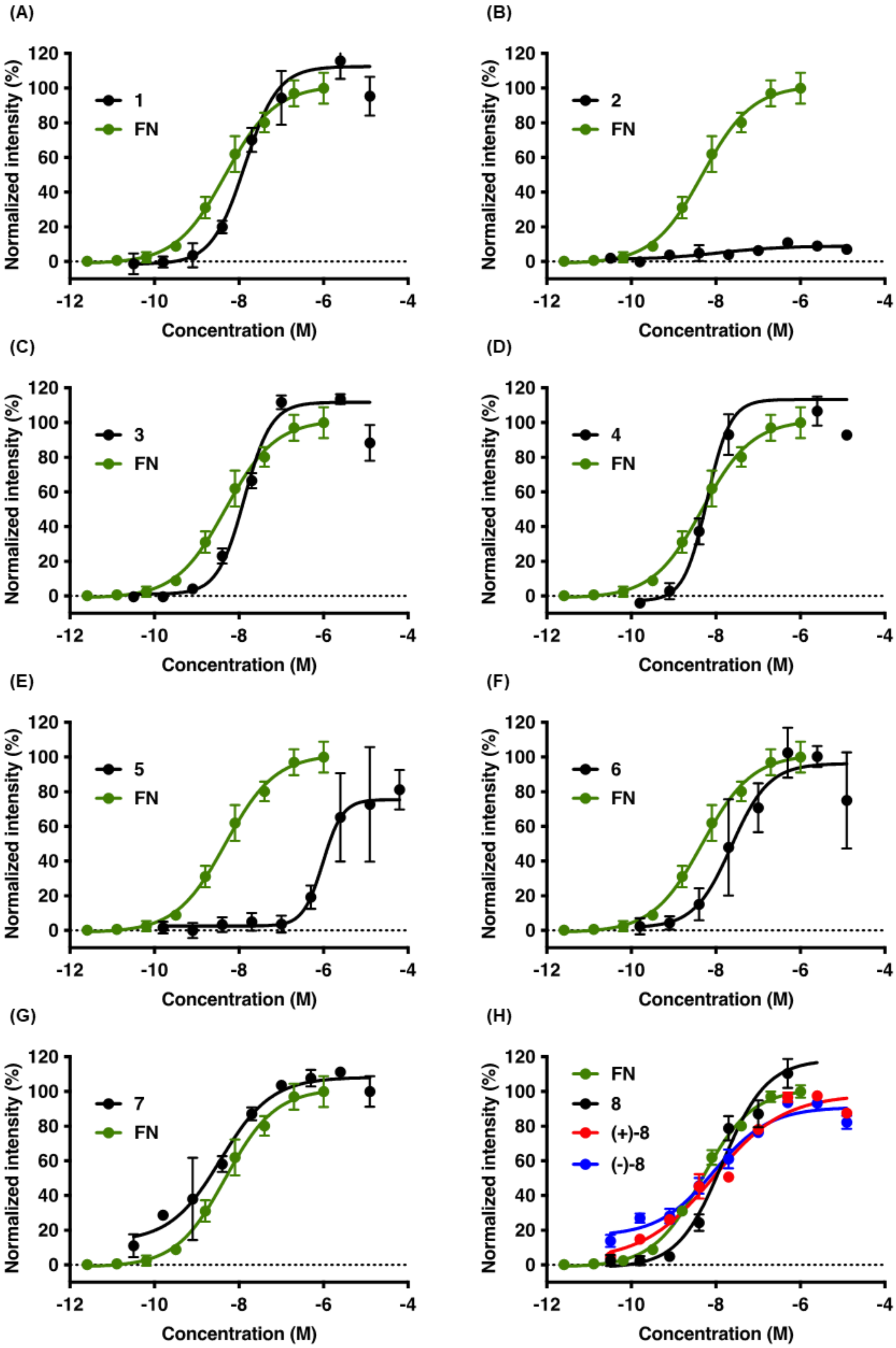

Figure S12. EC_{50} derivation from the concentration–response curves of test compounds **1**–**8**.

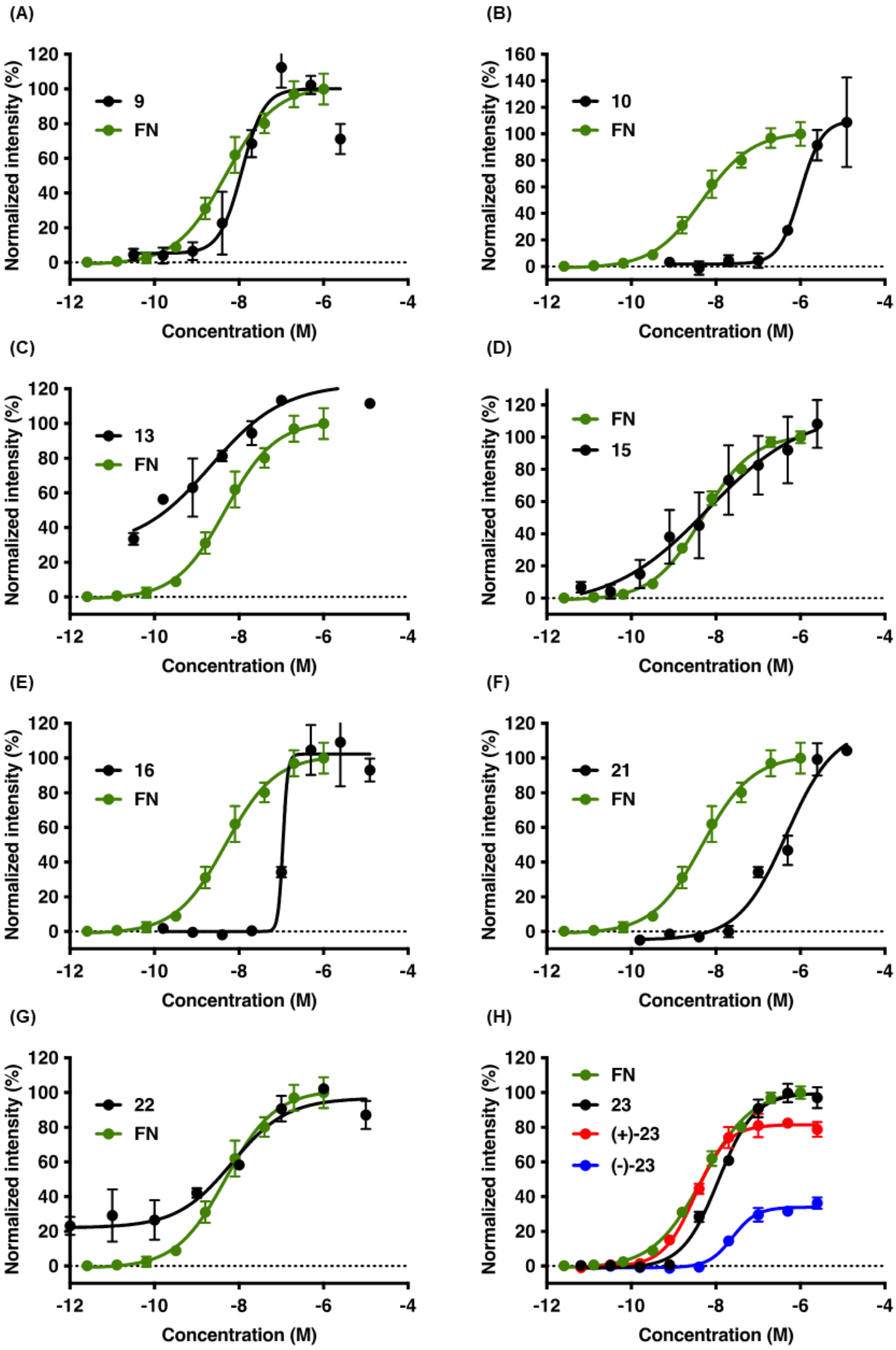

Figure S13. EC_{50} derivation from the concentration–response curves of test compounds **9, 10, 13, 15, 16 21–23**.

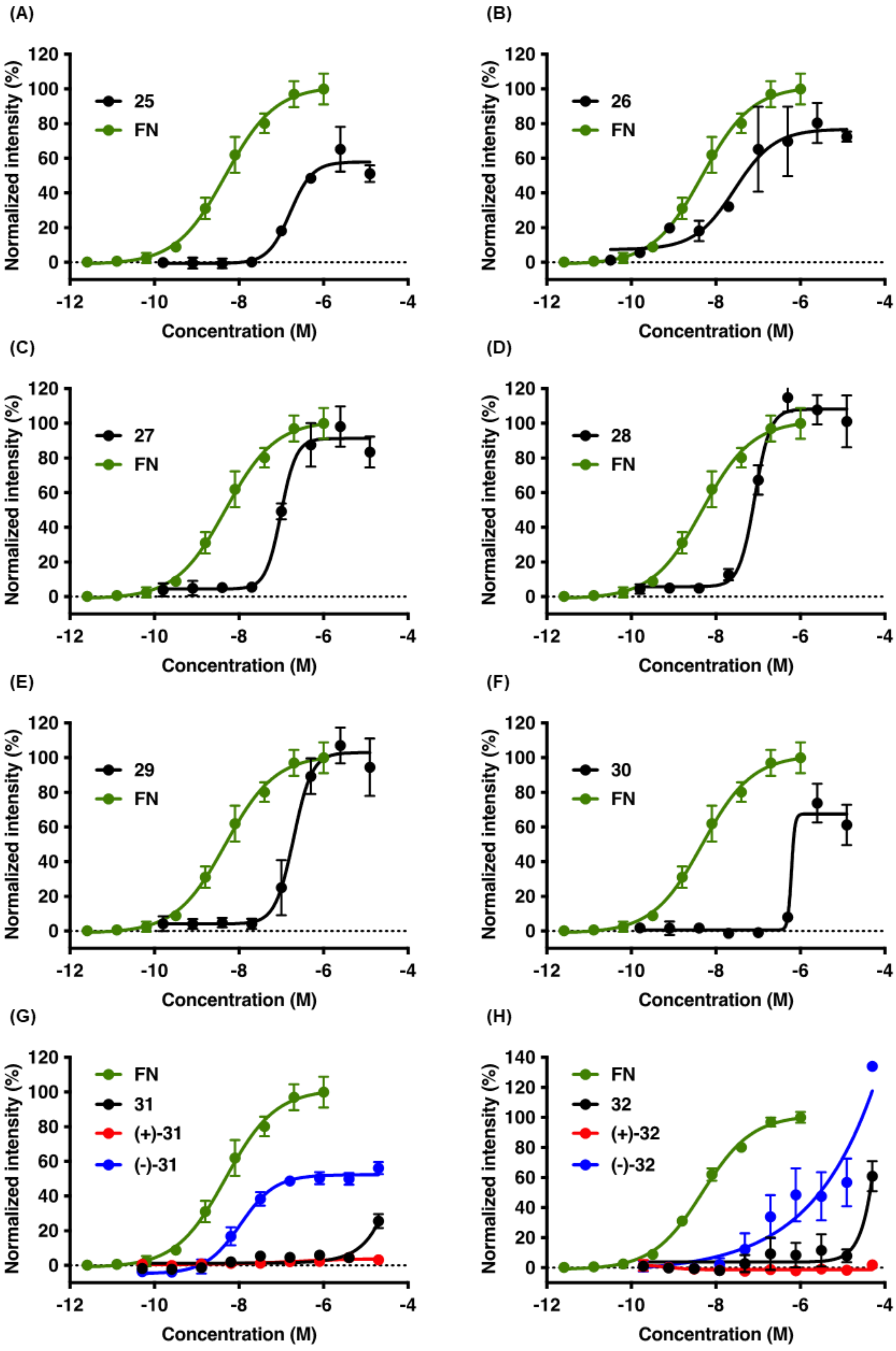

Figure S14. EC_{50} derivation from the concentration–response curves of test compounds **25**–**32**.

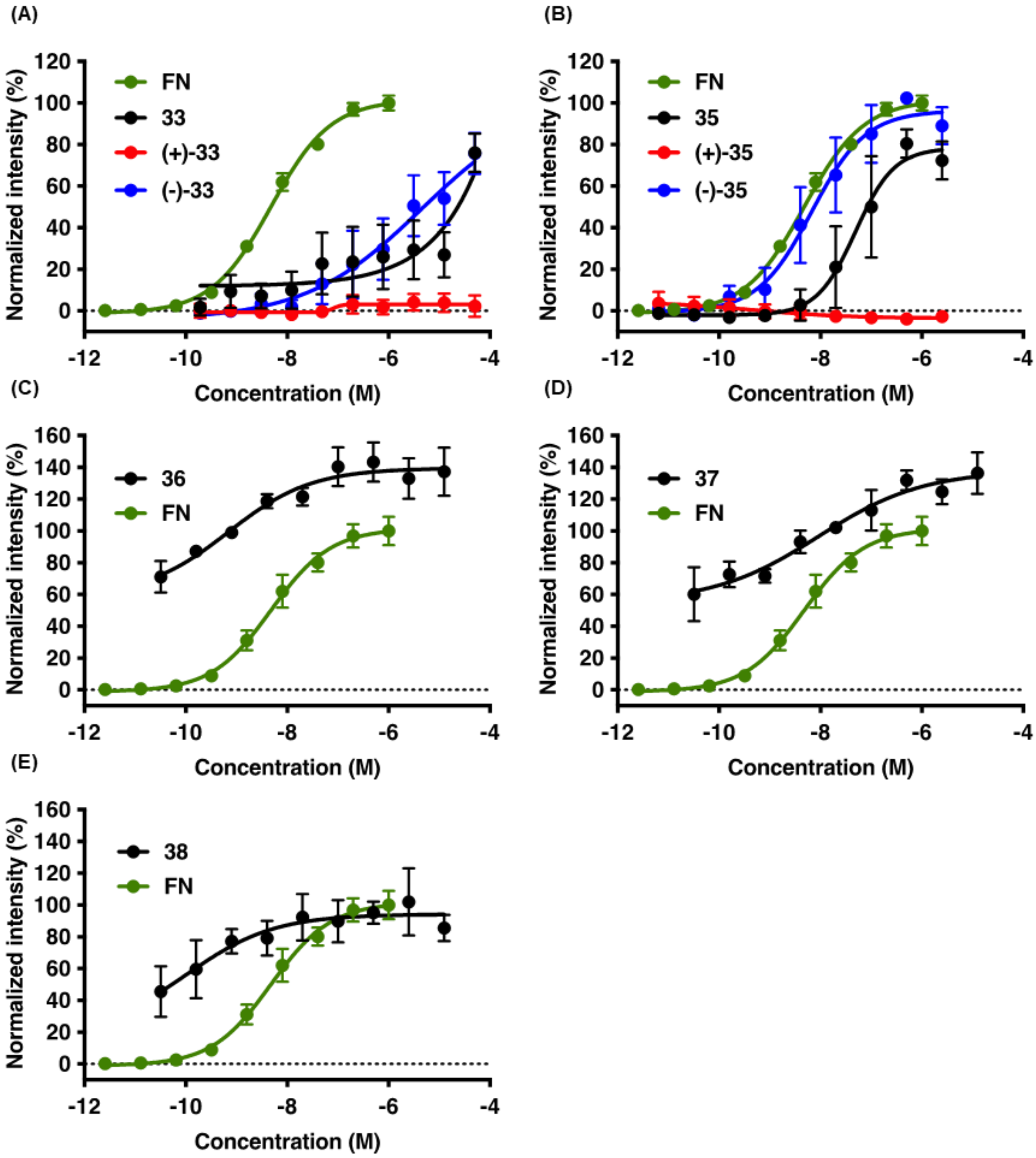

Figure S15. EC_{50} derivation from the concentration–response curves of test compounds **33, 35–38**.

5. *In vitro* μ–opioid receptor (MOR) assays (antagonistic activity)

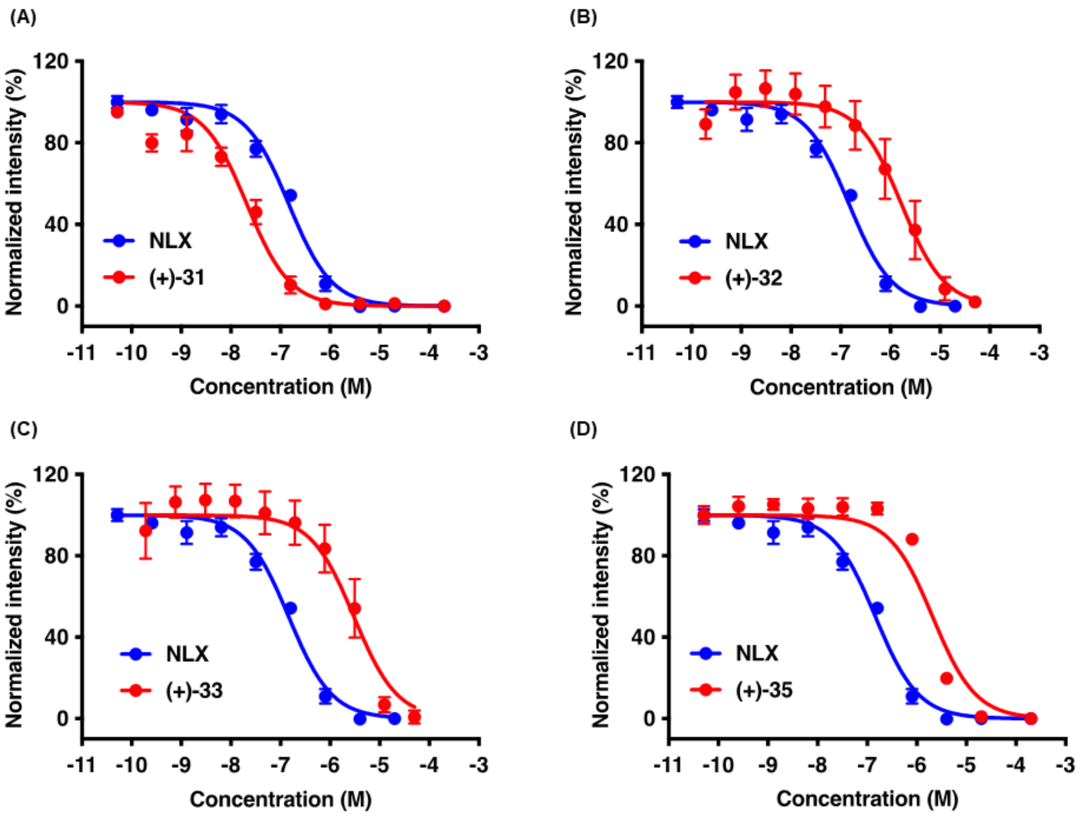

Figure S16. IC_{50} derivation from the concentration–response curves of test compounds.

6. *In vitro* competitive radioligand binding assay

Table S1. Scintillation counting conditions to assess the radioligand affinity.

Receptor	MOR
Source (cell line)	CHO-K1
Ligand	[^{3}H] Diprenorphine
Ligand concentration	0.6 nM
Non-specific ligand	10.0 μM Naloxone
Incubation buffer	50 mM Tris-HCl, pH 7.4
Incubation time and temperature	60 min @ 25 °C
Kd*	0.41 nM
Bmax*	3.80 pmol/mg protein

*Historical values (obtained experimentally at Eurofins Panlabs, Inc.). Data provided in the table were extracted from reports from Eurofins Panlabs Discovery Services Taiwan, Ltd. (New Taipei City, Taiwan). MOR, μ-opioid receptor; Kd, dissociation constant; Bmax, maximum number of binding sites

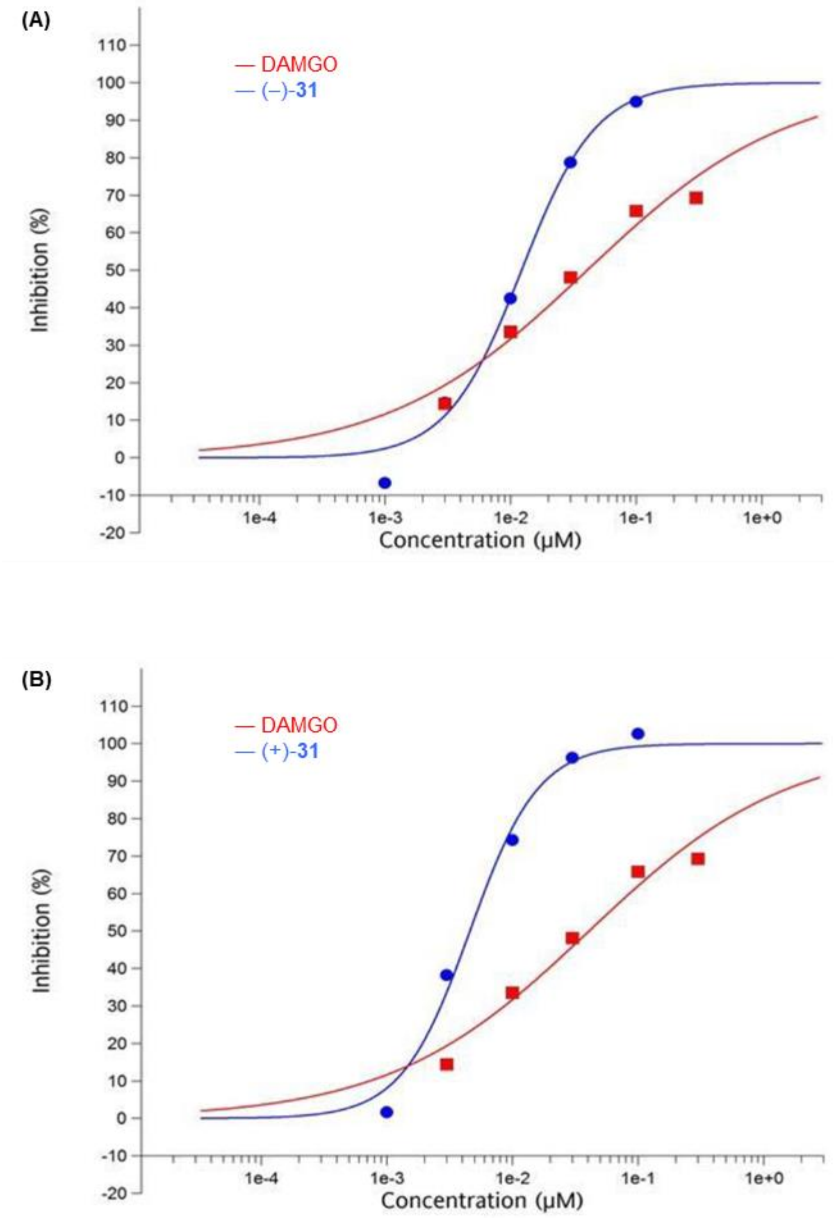

Figure S17. Determination of binding affinity from the concentration–response curves for (−)-**31** (A) and (+)-**31** (B).

7. Calculation of DFT-based ECD spectra (conformational search)

Table S2. Details of the conformation search and the subsequent conformer narrowing

Step	1	2		3			4		5′
Function	MMFF	HF/321G		ωB97X-D/6-31G*			B3LYP/6-31G*		ECD calc
	Rel E (kJ/mol)	Rel E (kJ/mol)		Rel E (kJ/mol)		Rel G (kJ/mol)	Rel E (kJ/mol)	Rel G (kJ/mol)	
M0001	−12.97	0.36		0.00		0.00	0.00	0.00	○
M0002	−11.80	3.19		0.08		0.39	1.14	−1.39	○
M0005	−10.96	0.00		1.99		1.34	6.74	7.30	○
M0004	−10.99	5.11		1.50		1.60	6.56	6.56	○
M0007	−10.65	0.26		3.40		2.70	0.78	−0.73	○
M0003	−11.43	0.50		0.82		2.99	6.42	5.07	○
M0006	−10.69	4.22		3.14		4.41	1.85	−0.13	○
M0008	−10.12	3.84		4.71		5.36	7.41	6.43	○
M0010	−4.71	14.67		4.79		5.38	2.63	1.60	○
M0013	−3.06	4.49		5.82		6.30	8.12	7.67	○
M0011	−3.25	17.67		5.16		6.56	3.95	2.62	○
M0015	−2.63	17.81		7.13		7.11	3.06	0.18	○
M0014	−2.77	17.56		6.20		7.74	3.84	1.44	○
M0012	−3.15	14.88		5.46		6.62	8.69	7.11	○
M0017	−2.42	15.90		8.06		9.00	11.98	10.58	
M0020	−1.74	7.81		9.06		9.35	15.06	12.92	
M0019	−1.85	16.84		8.58		9.71	8.81	6.72	
M0016	−2.56	8.90		7.93		10.08	9.80	8.51	
M0022	1.42	8.19		9.68		10.58	20.51	20.24	
M0018	−2.27	4.15		8.07		11.24	13.67	13.70	
M0023	3.00	10.92		9.71		11.45	20.39	20.66	
M0021	0.00	20.09		9.53		11.55	20.31	20.03	
M0024	3.56	7.80		9.98		12.78	13.83	13.99	
M0025	3.81	12.13		10.13	energy too high				
M0026	4.32	15.60		10.23	energy too high				
M0027	4.65	21.11		10.36	energy too high				
M0028	4.75	19.33		10.94	energy too high				
M0029	5.35	20.26		11.56	energy too high				
M0030	5.52	6.29		11.83	energy too high				
M0031	6.12	27.58		11.99	energy too high				
M0032	7.17	28.78		12.59	energy too high				
M0033	7.21	23.44		12.83	energy too high				
M0034	7.95	15.28		13.00	energy too high				
M0035	8.82	7.80		13.32	energy too high				
M0036	9.18	27.78		14.08	energy too high				
M0037	9.82	16.40		14.98	energy too high				
M0038	9.93	6.32		15.62	energy too high				
M0039	11.01	22.86		16.78	energy too high				
M0040	12.55	25.85		17.62	energy too high				
M0041	12.96	25.72		17.64	energy too high				
M0042	13.17	23.13		18.65	energy too high				
M0044	18.77	23.76		21.28	energy too high				
M0045	19.19	13.80		21.39	energy too high				
M0047	21.04	23.09		23.52	energy too high				
M0048	21.27	15.38		23.69	energy too high				
M0049	22.07	22.69		24.85	energy too high				
M0050	23.24	36.05		25.65	energy too high				
M0051	23.68	26.23		25.84	energy too high				
M0052	23.72	23.86		26.19	energy too high				
M0053	23.80	13.68		26.33	energy too high				
M0055	24.31	23.71		27.04	energy too high				
M0056	24.34	16.29		28.41	energy too high				
M0057	24.58	27.09		29.07	energy too high				
M0058	26.62	28.60		29.14	energy too high				
M0059	26.90	36.20		29.86	energy too high				
M0009	−5.00	0.01	duplicate						
M0043	13.98	41.69	energy too high						
M0054	23.93	43.76	energy too high						
M0046	19.90	44.72	energy too high						

Table S3. XYZ data for a*R*-35’

M0001	X	Y	Z	M0002	X	Y	Z	M0012	X	Y	Z
C	0.6304	−0.3239	0.9159	C	1.0572	0.2551	0.6749	C	1.0448	0.2403	0.6541
C	1.7168	−2.0476	2.8024	C	2.2029	2.0167	2.4884	C	2.2213	1.9055	2.5405
C	1.7615	−1.08	0.5821	C	0.4812	0.3999	1.9418	C	0.4825	0.3266	1.9346
C	0.033	−0.423	2.181	C	2.2151	0.9619	0.3113	C	2.1978	0.9682	0.3097
C	0.5926	−1.2906	3.1151	C	2.7753	1.8431	1.233	C	2.7726	1.7999	1.2669
C	2.2933	−1.9401	1.5459	C	1.0703	1.2954	2.8368	C	1.0901	1.1717	2.8681
H	0.1421	−1.3712	4.1009	H	3.6699	2.3977	0.9627	H	3.6653	2.365	1.0141
H	3.1681	−2.536	1.2962	H	0.6355	1.4142	3.8265	H	0.6702	1.2409	3.8684
H	2.1396	−2.7231	3.5403	H	2.6478	2.7085	3.1975	H	2.6812	2.5551	3.2789
C	2.4298	−0.9931	−0.7725	C	−0.7218	−0.4011	2.383	C	−0.7225	−0.4849	2.3504
H	1.7623	−0.524	−1.501	H	−0.8898	−1.2254	1.6854	H	−0.9002	−1.2796	1.6221
H	2.6184	−2.0117	−1.1334	H	−0.4904	−0.8576	3.3529	H	−0.4941	−0.9787	3.3023
C	3.7572	−0.2247	−0.7316	C	−1.9982	0.4373	2.5222	C	−1.9898	0.3613	2.5227
H	3.6038	0.8113	−0.4157	H	−2.3106	0.863	1.5633	H	−2.2818	0.8467	1.5863
H	4.225	−0.213	−1.7211	H	−2.822	−0.1784	2.8979	H	−2.8261	−0.2638	2.8512
H	4.4571	−0.6896	−0.029	H	−1.8503	1.2669	3.2219	H	−1.8397	1.1463	3.2715
C	−1.18	0.3963	2.5316	C	2.8551	0.7542	−1.0381	C	2.8317	0.8061	−1.0478
H	−1.0821	1.4236	2.1708	H	2.1308	0.8423	−1.8546	H	2.1024	0.8992	−1.858
H	−2.0864	−0.0232	2.0791	H	3.3006	−0.2446	−1.1149	H	3.2903	−0.1847	−1.1431
N	0.0482	0.5482	−0.0668	N	0.4567	−0.6129	−0.2994	N	0.4563	−0.6068	−0.3377
C	0.6225	1.7413	−0.4277	C	0.9928	−1.8283	−0.641	C	0.9493	−1.8543	−0.6407
C	1.6128	2.3895	0.4627	C	1.9947	−2.4701	0.2427	C	1.9936	−2.4566	0.2371
C	2.4229	3.444	0.1601	C	2.8004	−3.5273	−0.0618	C	2.6735	−2.1546	1.3885
C	3.1827	3.716	1.3343	C	3.5592	−3.8047	1.1115	C	3.5293	−3.2605	1.6564
C	2.7664	2.8122	2.2613	C	3.1479	−2.9001	2.0398	C	3.3128	−4.1454	0.6475
O	1.8176	2.0032	1.7498	O	2.2021	−2.0861	1.5305	O	2.3941	−3.6785	−0.2098
H	2.4552	3.9489	−0.7934	H	2.8263	−4.035	−1.0139	H	2.587	−1.2624	1.9863
H	3.9333	4.4803	1.4718	H	4.3053	−4.5736	1.2478	H	4.2132	−3.3714	2.4847
H	3.0363	2.6229	3.2886	H	3.4202	−2.7126	3.0667	H	3.7224	−5.1154	0.4124
O	0.3356	2.3142	−1.4734	O	0.6685	−2.4222	−1.6636	O	0.538	−2.4895	−1.601
C	−0.9715	0.0265	−1.005	C	−0.5642	−0.082	−1.2313	C	−0.5729	−0.077	−1.2592
C	−2.3361	−1.9093	−1.7937	C	−1.8619	1.8883	−2.06	C	−1.8496	1.9075	−2.0932
C	−3.2848	0.2875	−1.935	C	−2.9423	−0.2476	−2.026	C	−2.9537	−0.2161	−2.0472
N	−3.5544	−1.122	−1.6856	N	−3.1133	1.1849	−1.8236	N	−3.1095	1.2198	−1.858
C	−2.2701	0.8368	−0.9383	C	−1.9014	−0.8118	−1.0661	C	−1.9219	−0.7837	−1.0793
C	−1.2831	−1.4523	−0.7866	C	−0.7731	1.4255	−1.0951	C	−0.7666	1.4338	−1.1264
H	−1.9122	−1.8538	−2.8189	H	−1.5154	1.7479	−3.1057	H	−1.5026	1.7604	−3.1376
H	−2.91	0.4467	−2.9678	H	−2.6449	−0.4705	−3.0719	H	−2.6557	−0.4512	−3.0902
H	−2.6889	0.7727	0.0714	H	−2.2636	−0.6874	−0.0409	H	−2.2815	−0.637	−0.0563
H	−1.6661	−1.6173	0.2265	H	−1.0723	1.6833	−0.075	H	−1.0658	1.6936	−0.1062
H	−0.5595	0.1479	−2.0162	H	−0.1985	−0.29	−2.2449	H	−0.2198	−0.297	−2.2746
H	−2.5838	−2.9601	−1.6066	H	−2.0343	2.9613	−1.9216	H	−2.0093	2.9825	−1.9573
H	−4.2261	0.8413	−1.8476	H	−3.906	−0.7399	−1.8555	H	−3.923	−0.6964	−1.8757
H	−2.0603	1.8853	−1.1615	H	−1.7573	−1.8785	−1.2555	H	−1.7985	−1.8546	−1.2531
H	−0.3839	−2.0657	−0.903	H	0.152	1.9711	−1.3077	H	0.1619	1.9732	−1.3383
C	−4.585	−1.6268	−2.5698	C	−4.1771	1.7122	−2.6542	C	−4.1609	1.7514	−2.7012
H	−5.5051	−1.0497	−2.4323	H	−5.1152	1.1972	−2.4235	H	−5.1051	1.2442	−2.4785
H	−4.8008	−2.6731	−2.3293	H	−4.315	2.7787	−2.4484	H	−4.2934	2.8192	−2.4995
H	−1.3328	0.4196	3.6142	H	3.6483	1.4874	−1.2074	H	3.6127	1.5549	−1.2035
H	−4.3015	−1.5714	−3.6392	H	−3.9771	1.5934	−3.737	H	−3.95	1.6269	−3.7811
**M0010**	**X**	**Y**	**Z**	**M0026**	**X**	**Y**	**Z**	**M0003**	**X**	**Y**	**Z**
C	−0.6066	0.2972	0.9092	C	0.9072	0.4116	0.6897	C	0.7941	0.6186	0.8515

C	−1.7166	1.9387	2.8568	C	2.1874	2.0276	2.5521	C	1.6338	2.3627	2.8453
C	−1.7528	1.0457	0.6044	C	0.3459	0.5623	1.9656	C	0.2833	0.4694	2.1479
C	0.0028	0.3765	2.1718	C	2.0809	1.0914	0.3228	C	1.7364	1.6165	0.5475
C	−0.5702	1.2024	3.1376	C	2.7131	1.8921	1.2721	C	2.1439	2.4828	1.5563
C	−2.2968	1.8612	1.5989	C	1.0094	1.3739	2.8887	C	0.7199	1.3618	3.1325
H	−0.1127	1.2657	4.1213	H	3.6261	2.4157	1.0019	H	2.875	3.2547	1.3309
H	−3.1853	2.4462	1.3739	H	0.5887	1.493	3.8839	H	0.324	1.2656	4.1406
H	−2.1518	2.5779	3.6192	H	2.6903	2.6542	3.2826	H	1.9554	3.0477	3.6242
C	−2.4185	0.9979	−0.7536	C	−0.9188	−0.1522	2.3803	C	−0.687	−0.6231	2.5449
H	−1.7417	0.5628	−1.4947	H	−1.3866	0.4153	3.1928	H	−1.5634	−0.1592	3.0154
H	−2.6131	2.0284	−1.0753	H	−1.6332	−0.1569	1.5545	H	−1.0578	−1.1538	1.6652
C	−3.74	0.2181	−0.7562	C	−0.677	−1.5923	2.8447	C	−0.0718	−1.6335	3.5219
H	−3.5795	−0.8369	−0.5198	H	0.0353	−1.6204	3.6763	H	0.245	−1.1465	4.4501
H	−4.2153	0.2786	−1.7403	H	−1.6135	−2.0477	3.1817	H	−0.8008	−2.4085	3.7789
H	−4.4369	0.6249	−0.0155	H	−0.2712	−2.2046	2.0357	H	0.8033	−2.1224	3.0836
C	1.2356	−0.4269	2.492	C	2.6663	0.936	−1.057	C	2.3156	1.7275	−0.8383
H	1.1687	−1.4392	2.0839	H	1.9075	1.0545	−1.8372	H	2.617	0.7446	−1.2133
H	2.1308	0.0374	2.0624	H	3.1106	−0.0572	−1.188	H	3.1904	2.3835	−0.8414
N	−0.0397	−0.5625	−0.0864	N	0.2794	−0.4477	−0.2725	N	0.3275	−0.2148	−0.2214
C	−0.6164	−1.763	−0.4339	C	0.854	−1.6405	−0.6569	C	0.5515	−1.5664	−0.2741
C	−1.6369	−2.3589	0.4718	C	1.9268	−2.2401	0.1874	C	1.6815	−2.1369	0.4948
C	−2.1104	−2.17	1.7423	C	2.5515	−1.9954	1.3821	C	1.9106	−3.449	0.7842
C	−3.0996	−3.1716	1.9593	C	3.4941	−3.0465	1.5705	C	3.1228	−3.4879	1.5325
C	−3.1531	−3.8918	0.8078	C	3.3761	−3.8457	0.4774	C	3.5403	−2.1967	1.6282
O	−2.2787	−3.4181	−0.0937	O	2.4405	−3.3757	−0.3609	O	2.6805	−1.3678	1.0034
H	−1.8106	−1.4059	2.4415	H	2.3694	−1.1742	2.0566	H	1.2752	−4.2708	0.491
H	−3.6888	−3.3271	2.851	H	4.1659	−3.1811	2.4054	H	3.618	−4.3565	1.9411
H	−3.7428	−4.738	0.4903	H	3.8726	−4.7517	0.1658	H	4.395	−1.7242	2.0869
O	−0.3004	−2.3565	−1.4548	O	0.4974	−2.2273	−1.668	O	−0.1299	−2.3355	−0.9422
C	0.9768	−0.0463	−1.0289	C	−0.7258	0.0972	−1.2148	C	−0.6895	0.3937	−1.1028
C	2.3081	1.8993	−1.8512	C	−2.0388	2.0678	−2.0055	C	−3.1039	0.7967	−1.6195
C	3.2889	−0.2856	−1.964	C	−3.0166	−0.112	−2.2059	C	−1.5071	0.8608	−3.4117
N	3.5383	1.1316	−1.7372	N	−3.2712	1.2981	−1.9428	N	−2.8597	0.4934	−3.0212
C	2.2877	−0.8354	−0.9536	C	−2.0389	−0.693	−1.1897	C	−0.4706	0.1009	−2.588
C	1.2663	1.4403	−0.8332	C	−1.0217	1.5768	−0.9772	C	−2.1293	0.0541	−0.7078
H	1.8797	1.8232	−2.8732	H	−1.5875	2.022	−3.0194	H	−3.0253	1.8893	−1.4287
H	2.9122	−0.4666	−2.9924	H	−2.616	−0.2626	−3.2302	H	−1.3435	1.9556	−3.3028
H	2.712	−0.7484	0.0519	H	−2.4914	−0.6333	−0.1942	H	−0.5738	−0.9696	−2.7721
H	1.6517	1.626	0.1756	H	−1.4321	1.7285	0.0271	H	−2.2824	−1.0245	−0.7969
H	0.5668	−0.1891	−2.038	H	−0.3032	−0.0142	−2.2229	H	−0.5612	1.4741	−0.9601
H	2.5412	2.9561	−1.68	H	−2.2748	3.1192	−1.8076	H	−4.1313	0.5047	−1.3755
H	4.2389	−0.8234	−1.8725	H	−3.9683	−0.6517	−2.1524	H	−1.3776	0.6235	−4.4734
H	2.0932	−1.8903	−1.1588	H	−1.8401	−1.7413	−1.4189	H	0.5386	0.4058	−2.8863
H	0.3572	2.0382	−0.9548	H	−0.1122	2.1812	−1.0551	H	−2.3125	0.3499	0.3314
C	4.5575	1.6389	−2.6333	C	−4.2699	1.8327	−2.8459	C	−3.8457	1.1223	−3.875
H	5.4847	1.0726	−2.4979	H	−5.2032	1.2709	−2.7383	H	−3.671	0.8347	−4.9169
H	4.7642	2.6893	−2.4028	H	−4.4731	2.8797	−2.5977	H	−4.8488	0.7857	−3.5931
H	1.3848	−0.495	3.5731	H	3.45	1.6782	−1.2301	H	1.5911	2.1382	−1.5518
H	4.2673	1.5714	−3.7001	H	−3.9609	1.7855	−3.9084	H	−3.8257	2.2288	−3.8159
**M0005**	**X**	**Y**	**Z**	**M0014**	**X**	**Y**	**Z**	**M0006**	**X**	**Y**	**Z**
C	−0.4323	0.2573	1.0779	C	−0.3736	0.1597	1.1069	C	0.8555	0.227	0.8972
C	−1.2667	1.9841	3.0845	C	−1.3806	1.8548	3.0532	C	1.8545	1.91	2.8644
C	−1.4661	1.1613	0.8134	C	−1.4706	0.9779	0.7946	C	0.3051	0.1734	2.1845
C	0.1872	0.2009	2.3376	C	0.2219	0.1761	2.3776	C	1.9119	1.0967	0.5858
C	−0.2476	1.0711	3.3334	C	−0.2976	1.0345	3.3448	C	2.395	1.9392	1.5825

C	−1.866	2.0259	1.8348	C	−1.96	1.8257	1.7902	C	0.8263	1.0284	3.1601
H	0.2236	1.0371	4.3122	H	0.1505	1.0566	4.3341	H	3.2139	2.6164	1.3548
H	−2.6634	2.7391	1.6389	H	−2.8097	2.4674	1.5831	H	0.4129	0.9935	4.1655
H	−1.5893	2.6652	3.8667	H	−1.7767	2.5207	3.8139	H	2.2457	2.5686	3.6343
C	−2.1914	1.2003	−0.5112	C	−2.1104	0.9106	−0.5758	C	−0.8322	−0.7529	2.5627
H	−1.6355	0.6293	−1.2565	H	−1.9746	−0.1015	−0.9675	H	−0.9352	−1.5702	1.8452
H	−2.2321	2.2372	−0.8673	H	−1.558	1.5695	−1.2591	H	−0.5871	−1.2189	3.5244
C	−3.6148	0.6378	−0.4136	C	−3.5949	1.2718	−0.6214	C	−2.1816	−0.0343	2.6875
H	−3.5919	−0.4104	−0.1035	H	−4.1687	0.6664	0.088	H	−2.518	0.3477	1.7186
H	−4.1214	0.7018	−1.3821	H	−3.9922	1.0861	−1.6235	H	−2.9483	−0.7222	3.0583
H	−4.2111	1.192	0.3193	H	−3.7741	2.3267	−0.3915	H	−2.1184	0.812	3.3796
C	1.2905	−0.7889	2.6134	C	1.3717	−0.7423	2.7009	C	2.5315	1.0933	−0.7862
H	2.0691	−0.7561	1.844	H	2.1516	−0.7057	1.9341	H	2.7557	0.072	−1.107
H	1.7604	−0.5855	3.5795	H	1.8227	−0.4767	3.6605	H	3.4597	1.6717	−0.7932
N	0.0433	−0.6198	0.0424	N	0.1577	−0.7265	0.1163	N	0.3253	−0.614	−0.1399
C	−0.1919	−1.972	0.0599	C	−0.0912	−2.0797	0.1231	C	0.4855	−1.9782	−0.0976
C	−1.265	−2.5398	0.9112	C	−1.1719	−2.6117	1.0012	C	1.6227	−2.5493	0.6599
C	−1.5006	−3.8617	1.1502	C	−2.1548	−2.1307	1.8267	C	1.7729	−3.8298	1.1005
C	−2.6365	−3.9199	2.0076	C	−2.8754	−3.2628	2.3018	C	3.0456	−3.8866	1.7398
C	−3.0098	−2.6292	2.2156	C	−2.2747	−4.3438	1.7375	C	3.5731	−2.6387	1.6249
O	−2.1893	−1.7824	1.5616	O	−1.2527	−3.9697	0.9543	O	2.7246	−1.8176	0.9743
H	−0.9202	−4.6777	0.7471	H	−2.3553	−1.1013	2.0747	H	1.0512	−4.6219	0.9695
H	−3.1142	−4.8009	2.4106	H	−3.7203	−3.2645	2.9743	H	3.5071	−4.7391	2.2161
H	−3.8029	−2.1649	2.7812	H	−2.4537	−5.406	1.7975	H	4.504	−2.1939	1.9405
O	0.463	−2.7557	−0.619	O	0.5324	−2.8501	−0.5926	O	−0.2801	−2.7507	−0.6629
C	1.1652	−0.1867	−0.8257	C	1.227	−0.2723	−0.7986	C	−0.7989	−0.1272	−0.9707
C	2.022	0.1242	−3.1575	C	1.9485	0.1049	−3.1697	C	−1.7165	0.1305	−3.2905
C	2.8322	1.5701	−1.4234	C	2.8335	1.5247	−1.4529	C	−2.3069	1.7491	−1.6219
N	2.4698	1.4706	−2.8284	N	2.3855	1.4534	−2.8345	N	−2.0146	1.5321	−3.0299
C	1.6549	1.2258	−0.5135	C	1.7176	1.1401	−0.4831	C	−1.1261	1.348	−0.7413
C	0.8147	−0.2726	−2.3146	C	0.7993	−0.3364	−2.2694	C	−0.5278	−0.3458	−2.4627
H	2.8348	−0.6175	−3.0095	H	2.7851	−0.6194	−3.0848	H	−2.5953	−0.5123	−3.0738
H	3.6891	0.9059	−1.1809	H	3.7122	0.8676	−1.2815	H	−3.2105	1.185	−1.3058
H	0.8511	1.9515	−0.68	H	0.8979	1.8597	−0.5853	H	−0.2678	1.9763	−1.0007
H	−0.009	0.414	−2.5393	H	−0.0542	0.3293	−2.4326	H	0.3657	0.2187	−2.7526
H	1.9903	−0.8893	−0.6451	H	2.0634	−0.9711	−0.6687	H	−1.6813	−0.7241	−0.6972
H	1.7553	0.0984	−4.22	H	1.6223	0.0958	−4.2153	H	−1.4928	0.0167	−4.357
H	3.1576	2.5968	−1.2215	H	3.1557	2.5507	−1.2448	H	−2.5258	2.8123	−1.4729
H	1.97	1.3179	0.5309	H	2.0927	1.213	0.5427	H	−1.3693	1.5474	0.3058
H	0.5039	−1.2896	−2.5628	H	0.4995	−1.3554	−2.5217	H	−0.3463	−1.4049	−2.656
C	3.5615	1.8898	−3.6834	C	3.413	1.9173	−3.744	C	−3.0933	2.008	−3.8715
H	3.8409	2.9224	−3.4486	H	3.6848	2.9496	−3.5008	H	−3.2615	3.075	−3.6913
H	3.246	1.8526	−4.7313	H	3.0334	1.8993	−4.7706	H	−2.8245	1.8793	−4.925
H	0.9088	−1.8164	2.639	H	1.0337	−1.7828	2.7643	H	1.8623	1.5292	−1.5362
H	4.4636	1.2563	−3.5743	H	4.3321	1.3005	−3.7068	H	−4.0477	1.4747	−3.6934
**M0007**	**X**	**Y**	**Z**	**M0017**	**X**	**Y**	**Z**	**M0011**	**X**	**Y**	**Z**
C	−0.5298	0.5336	1.1715	C	0.7828	0.5865	0.8519	C	0.8213	0.2127	0.8381
C	−1.2509	2.2707	3.2199	C	1.7088	2.2673	2.8631	C	1.9555	1.8392	2.7823
C	−1.3724	1.6225	0.887	C	0.2937	0.425	2.1568	C	0.2875	0.2032	2.136
C	−0.0489	0.3041	2.4702	C	1.7347	1.5761	0.5458	C	1.9067	1.0374	0.4988
C	−0.4279	1.1868	3.4842	C	2.1884	2.4085	1.5652	C	2.4614	1.8489	1.4866
C	−1.7159	2.4829	1.9274	C	0.7744	1.2841	3.149	C	0.8786	1.0236	3.0997
H	−0.0617	1.017	4.4933	H	2.9291	3.1709	1.3393	H	3.3073	2.4841	1.2394
H	−2.3668	3.3282	1.719	H	0.3989	1.1767	4.1636	H	0.4931	1.013	4.1156
H	−1.5304	2.9511	4.0188	H	2.0668	2.9244	3.65	H	2.4057	2.4665	3.5454

C	−1.9552	1.8604	−0.4894	C	−0.7051	−0.6446	2.5461	C	−0.876	−0.6834	2.5278
H	−1.1702	1.8524	−1.2523	H	−1.5651	−0.1572	3.0229	H	−1.1969	−1.2876	1.6763
H	−2.3807	2.8701	−0.5091	H	−1.0935	−1.152	1.6604	H	−0.5317	−1.3916	3.2914
C	−3.0443	0.8544	−0.8789	C	−0.1256	−1.6883	3.5101	C	−2.0818	0.0946	3.0667
H	−2.6397	−0.1571	−0.9605	H	0.2205	−1.2243	4.4396	H	−2.4739	0.7873	2.3152
H	−3.4826	1.1246	−1.8452	H	−0.8873	−2.4311	3.7667	H	−2.8859	−0.5945	3.3419
H	−3.842	0.8375	−0.1303	H	0.7239	−2.2107	3.0618	H	−1.8244	0.6804	3.9547
C	0.8095	−0.8859	2.822	C	2.2804	1.7142	−0.8521	C	2.4816	1.0323	−0.8926
H	1.4555	−1.1986	1.9991	H	2.5203	0.7357	−1.28	H	2.5399	0.017	−1.2935
H	1.4423	−0.6581	3.6847	H	3.1857	2.3273	−0.8561	H	3.4835	1.4695	−0.8996
N	−0.1278	−0.3345	0.0994	N	0.2986	−0.2339	−0.2185	N	0.2707	−0.6511	−0.1614
C	−0.4035	−1.6789	0.0942	C	0.5249	−1.5885	−0.2827	C	0.4926	−2.0101	−0.1565
C	−1.5247	−2.1961	0.915	C	1.6555	−2.1348	0.514	C	1.572	−2.5657	0.7066
C	−1.8041	−3.5013	1.1942	C	2.8005	−1.6515	1.0838	C	2.5918	−2.1054	1.4976
C	−2.984	−3.4971	1.9925	C	3.4818	−2.7744	1.6344	C	3.2842	−3.2537	1.9755
C	−3.3332	−2.1898	2.129	C	2.6945	−3.8504	1.3661	C	2.6291	−4.3226	1.4497
O	−2.4611	−1.3906	1.4841	O	1.596	−3.4821	0.6857	O	1.6025	−3.9262	0.6838
H	−1.2238	−4.3465	0.8569	H	3.118	−0.6209	1.1208	H	2.8348	−1.079	1.7192
H	−3.5034	−4.349	2.4063	H	4.4235	−2.776	2.1633	H	4.1459	−3.2735	2.626
H	−4.1429	−1.6856	2.6334	H	2.7787	−4.9035	1.5863	H	2.7699	−5.3893	1.5279
O	0.2207	−2.4879	−0.5826	O	−0.1366	−2.3407	−0.982	O	−0.1633	−2.7681	−0.8568
C	0.8911	0.2013	−0.8272	C	−0.7095	0.3858	−1.1022	C	−0.8229	−0.1839	−1.0396
C	3.3017	0.4813	−1.4332	C	−3.1203	0.7958	−1.6313	C	−1.626	0.1871	−3.3847
C	1.6513	0.5637	−3.1759	C	−1.5105	0.8845	−3.411	C	−2.4146	1.6417	−1.65
N	2.9994	0.1498	−2.8168	N	−2.8661	0.5134	−3.0355	N	−2.0194	1.5484	−3.0466
C	0.6131	−0.1265	−2.2956	C	−0.4808	0.1127	−2.5899	C	−1.2726	1.2401	−0.7187
C	2.3269	−0.1899	−0.4673	C	−2.1523	0.0398	−0.7235	C	−0.4537	−0.2731	−2.5247
H	3.277	1.5814	−1.2732	H	−3.0429	1.8855	−1.4238	H	−2.4754	−0.5164	−3.2613
H	1.5375	1.6672	−3.096	H	−1.3467	1.9777	−3.2863	H	−3.3007	1.006	−1.4402
H	0.6685	−1.2053	−2.4477	H	−0.5842	−0.955	−2.7883	H	−0.4439	1.9439	−0.853
H	2.4304	−1.2766	−0.5246	H	−2.3034	−1.0376	−0.8296	H	0.4101	0.3678	−2.7278
H	0.8198	1.2894	−0.7111	H	−0.5843	1.4646	−0.9445	H	−1.6699	−0.8624	−0.8703
H	4.3228	0.151	−1.2127	H	−4.1495	0.5005	−1.399	H	−1.3398	0.1613	−4.4417
H	1.4766	0.3008	−4.225	H	−1.3731	0.6614	−4.4748	H	−2.7063	2.6764	−1.4399
H	−0.3934	0.2064	−2.574	H	0.5303	0.42	−2.8788	H	−1.608	1.3238	0.3191
H	2.558	0.1343	0.5537	H	−2.344	0.32	0.3185	H	−0.1892	−1.3018	−2.7764
C	3.9822	0.713	−3.7189	C	−3.8458	1.1545	−3.8876	C	−3.0697	2.0268	−3.9219
H	3.7622	0.4046	−4.7461	H	−3.6608	0.886	−4.9328	H	−3.3007	3.0707	−3.6866
H	4.9793	0.3426	−3.459	H	−4.8503	0.8102	−3.6208	H	−2.7344	1.9799	−4.963
H	0.1862	−1.7485	3.0876	H	1.556	2.1886	−1.525	H	1.8593	1.6178	−1.5787
H	4.0094	1.8205	−3.6909	H	−3.8291	2.2598	−3.8089	H	−4.0038	1.4382	−3.8356
**M0004**	**X**	**Y**	**Z**	**M0008**	**X**	**Y**	**Z**	**M0015**	**X**	**Y**	**Z**
C	0.6847	−0.3645	1.1895	C	0.9424	0.5276	0.8527	C	−0.6476	0.3639	1.1948
C	1.5241	−2.1773	3.1234	C	1.9285	2.2421	2.8028	C	−1.4813	2.1699	3.1406
C	1.6129	−1.3596	0.8455	C	0.4799	0.3993	2.1701	C	−1.5674	1.3707	0.8552
C	0.2009	−0.2424	2.5025	C	1.8912	1.5036	0.5012	C	−0.1613	0.2362	2.5079
C	0.6349	−1.1645	3.4559	C	2.3656	2.3613	1.4875	C	−0.5919	1.154	3.4659
C	2.0137	−2.2646	1.8271	C	1.0029	1.2663	3.1354	C	−1.9684	2.2694	1.8444
H	0.2644	−1.082	4.4745	H	3.102	3.1165	1.2261	H	−0.2226	1.0643	4.4842
H	2.736	−3.0359	1.5696	H	0.6735	1.1691	4.1666	H	−2.686	3.0464	1.5913
H	1.8466	−2.8889	3.8778	H	2.3177	2.9072	3.5681	H	−1.804	2.8753	3.9005
C	2.2266	−1.4486	−0.5332	C	−0.5407	−0.6368	2.5956	C	−2.178	1.4772	−0.5244
H	3.3094	−1.574	−0.4138	H	−0.86	−1.2368	1.741	H	−3.2575	1.6319	−0.408
H	2.0827	−0.5004	−1.0579	H	−0.0645	−1.3346	3.2959	H	−2.0563	0.527	−1.0518
C	1.6912	−2.6062	−1.3843	C	−1.7823	−0.0239	3.2529	C	−1.612	2.6237	−1.3709

H	1.8372	−3.566	−0.8773	H	−2.2738	0.6839	2.5771	H	−2.1298	2.6803	−2.3338
H	2.2125	−2.6483	−2.3463	H	−2.5031	−0.8061	3.5111	H	−0.5447	2.4904	−1.5725
H	0.6211	−2.4991	−1.5887	H	−1.5327	0.5175	4.1709	H	−1.7365	3.5851	−0.8612
C	−0.7133	0.8808	2.9246	C	2.4156	1.5931	−0.9073	C	0.7415	−0.8971	2.9233
H	−1.3922	1.193	2.1286	H	2.6876	0.6016	−1.282	H	1.4197	−1.2065	2.1262
H	−1.3121	0.5851	3.7909	H	3.2997	2.2351	−0.9513	H	1.3387	−0.6171	3.796
N	0.1982	0.5212	0.1688	N	0.4477	−0.3361	−0.1818	N	−0.1865	−0.5325	0.176
C	0.4276	1.8729	0.1882	C	0.6629	−1.6909	−0.1654	C	−0.4686	−1.8781	0.178
C	1.5805	2.3848	0.9656	C	1.8014	−2.214	0.6265	C	−1.6123	−2.3456	1.0083
C	1.7991	3.6593	1.3958	C	1.9856	−3.481	1.0926	C	−2.7074	−1.7912	1.6129
C	3.0609	3.6468	2.0586	C	3.2498	−3.4849	1.7506	C	−3.4522	−2.8712	2.1682
C	3.5154	2.3686	1.9651	C	3.7385	−2.2224	1.6218	C	−2.7534	−3.9964	1.8606
O	2.6305	1.5918	1.3082	O	2.8749	−1.4415	0.9418	O	−1.6479	−3.6969	1.1595
H	1.1285	4.4914	1.2426	H	1.2922	−4.2987	0.9659	H	−2.9564	−0.7427	1.6644
H	3.5644	4.4756	2.5344	H	3.7314	−4.3138	2.2481	H	−4.378	−2.8131	2.7217
H	4.4135	1.8734	2.3002	H	4.6494	−1.7416	1.9428	H	−2.9088	−5.0443	2.0666
O	−0.2635	2.6817	−0.4196	O	−0.0351	−2.4923	−0.775	O	0.1537	−2.6853	−0.4958
C	−0.8182	−0.0377	−0.7467	C	−0.5694	0.2359	−1.0851	C	0.8281	0.0033	−0.7561
C	−3.229	−0.3674	−1.3363	C	−2.9851	0.592	−1.6343	C	3.2402	0.2574	−1.3815
C	−1.595	−0.4032	−3.092	C	−1.3761	0.608	−3.4167	C	1.5813	0.3781	−3.1085
N	−2.9466	−0.0169	−2.7195	N	−2.7277	0.2418	−3.0227	N	2.9244	−0.0607	−2.7645
C	−0.5596	0.2971	−2.2166	C	−0.3378	−0.1083	−2.5574	C	0.5357	−0.2995	−2.2271
C	−2.2606	0.3172	−0.3737	C	−2.0087	−0.1069	−0.6908	C	2.2633	−0.4078	−0.4141
H	−3.1781	−1.4681	−1.1867	H	−2.9208	1.6917	−1.4826	H	3.2311	1.3568	−1.2142
H	−1.4606	−1.5052	−3.0206	H	−1.2241	1.7077	−3.3479	H	1.4874	1.4833	−3.0188
H	−0.6262	1.3763	−2.3661	H	−0.4287	−1.1863	−2.7015	H	0.563	−1.3778	−2.3918
H	−2.3882	1.4017	−0.4202	H	−2.1473	−1.1895	−0.7451	H	2.3529	−1.4951	−0.4797
H	−0.7156	−1.1225	−0.6317	H	−0.4517	1.3215	−0.9771	H	0.7672	1.0895	−0.6236
H	−4.2552	−0.0622	−1.1035	H	−4.0107	0.2965	−1.3868	H	4.2582	−0.0879	−1.1694
H	−1.4329	−0.1309	−4.1408	H	−1.2361	0.3327	−4.4678	H	1.3929	0.1279	−4.1584
H	0.4447	−0.0304	−2.5073	H	0.6697	0.1957	−2.8614	H	−0.4606	0.0657	−2.4996
H	−2.4767	−0.021	0.6459	H	−2.2041	0.2195	0.3365	H	2.5095	−0.0939	0.6064
C	−3.9267	−0.5896	−3.6187	C	−3.713	0.8305	−3.9055	C	3.9095	0.4921	−3.6705
H	−3.7172	−0.2737	−4.646	H	−3.5287	0.5063	−4.9349	H	3.6797	0.1886	−4.6971
H	−4.927	−0.2339	−3.3509	H	−4.7153	0.4961	−3.6182	H	4.9034	0.1086	−3.4172
H	−0.1292	1.764	3.211	H	1.6699	2.0076	−1.5963	H	0.1451	−1.7758	3.1975
H	−3.9383	−1.6974	−3.5959	H	−3.7025	1.9385	−3.887	H	3.9509	1.5992	−3.64
**M0013**	**X**	**Y**	**Z**	**M0022**	**X**	**Y**	**Z**	**M0019**	**X**	**Y**	**Z**
C	0.7499	0.8265	0.8072	C	−0.6335	0.3516	0.7098	C	0.9289	0.5081	0.8554
C	1.6098	2.5561	2.8053	C	−1.7557	2.0843	2.5676	C	2.0025	2.1925	2.7884
C	0.2452	0.674	2.1059	C	−1.7704	1.0905	0.3585	C	0.474	0.4081	2.1789
C	1.6955	1.8211	0.5033	C	−0.0482	0.4731	1.9788	C	1.907	1.4503	0.4883
C	2.1138	2.6794	1.5145	C	−0.6246	1.3455	2.8978	C	2.4297	2.29	1.4681
C	0.6916	1.5592	3.0922	C	−2.3208	1.9544	1.3084	C	1.0373	1.2591	3.1341
H	2.8484	3.448	1.2892	H	−0.1824	1.4438	3.8859	H	3.1918	3.015	1.1958
H	0.3003	1.4605	4.1019	H	−3.2011	2.5362	1.0448	H	0.7149	1.1788	4.1687
H	1.9395	3.2353	3.5859	H	−2.1926	2.7635	3.2939	H	2.4283	2.8421	3.547
C	−0.7299	−0.414	2.5039	C	−2.4257	0.9802	−1.0006	C	−0.5688	−0.5968	2.6202
H	−1.6018	0.054	2.9787	H	−1.741	0.5221	−1.7204	H	−0.8832	−1.2175	1.779
H	−1.1076	−0.9397	1.6243	H	−2.6343	1.992	−1.3698	H	−0.1117	−1.2785	3.3481
C	−0.1173	−1.4307	3.4761	C	−3.7361	0.1831	−0.9653	C	−1.8066	0.059	3.2424
H	0.2067	−0.9485	4.4042	H	−3.5627	−0.8465	−0.6388	H	−2.2803	0.7495	2.5368
H	−0.8502	−2.2018	3.7342	H	−4.194	0.1521	−1.9591	H	−2.542	−0.7019	3.5211
H	0.7528	−1.9242	3.0328	H	−4.4528	0.6382	−0.2733	H	−1.5564	0.6294	4.1425
C	2.2683	1.938	−0.8846	C	1.1718	−0.3269	2.3491	C	2.4127	1.5256	−0.9284

H	2.5575	0.9559	−1.2702	H	1.089	−1.3597	1.9999	H	2.6147	0.5265	−1.3263
H	3.1489	2.5864	−0.8872	H	2.0768	0.0986	1.8982	H	3.3326	2.1139	−0.9807
N	0.2727	0.0002	−0.2665	N	−0.0314	−0.5231	−0.2581	N	0.4109	−0.3577	−0.1606
C	0.4813	−1.3531	−0.3199	C	−0.5866	−1.7275	−0.6112	C	0.656	−1.711	−0.1799
C	1.6137	−1.9348	0.4356	C	−1.5664	−2.3859	0.2833	C	1.775	−2.235	0.6522
C	1.8334	−3.2484	0.7256	C	−2.3532	−3.4606	−0.0094	C	2.8613	−1.7354	1.3192
C	3.0568	−3.2979	1.4543	C	−3.1103	−3.7349	1.1654	C	3.5816	−2.8583	1.8172
C	3.49	−2.0111	1.5383	C	−2.7101	−2.8177	2.0862	C	2.8751	−3.9515	1.4243
O	2.6286	−1.1748	0.9257	O	−1.78	−1.9918	1.5669	O	1.7905	−3.5934	0.72
H	1.185	−4.0645	0.4449	H	−2.3721	−3.9762	−0.9577	H	3.1261	−0.6974	1.4447
H	3.5491	−4.1703	1.8582	H	−3.8459	−4.5126	1.3096	H	4.4941	−2.8478	2.3948
H	4.3574	−1.5467	1.9813	H	−2.9829	−2.6266	3.1125	H	3.0123	−5.0122	1.5663
O	−0.2157	−2.1172	−0.9802	O	−0.2885	−2.3043	−1.6516	O	0.0136	−2.4865	−0.8721
C	−0.752	0.6114	−1.1411	C	0.9988	−0.0011	−1.1876	C	−0.5991	0.218	−1.0703
C	−3.193	0.9261	−1.6696	C	2.3736	1.9379	−1.9759	C	−3.0158	0.5485	−1.6366
C	−1.617	0.9999	−3.4637	C	3.3358	−0.2489	−2.0802	C	−1.3885	0.6443	−3.3993
N	−2.9706	0.6238	−3.0772	N	3.6106	1.1727	−1.9048	N	−2.7383	0.2454	−3.0318
C	−0.5191	0.3227	−2.6284	C	2.2996	−0.8098	−1.0965	C	−0.3487	−0.0829	−2.5503
C	−2.1847	0.2641	−0.7156	C	1.287	1.4866	−0.9842	C	−2.0369	−0.1621	−0.704
H	−3.1349	2.0179	−1.5518	H	1.9821	1.8441	−2.9987	H	−2.9717	1.644	−1.4513
H	−1.5347	2.0919	−3.3612	H	2.9691	−0.3942	−3.1059	H	−1.2555	1.7438	−3.2957
H	−0.5254	−0.755	−2.8006	H	2.6859	−0.7644	−0.0718	H	−0.4259	−1.1569	−2.7241
H	−2.3043	−0.8231	−0.7179	H	1.6166	1.677	0.0441	H	−2.1533	−1.2452	−0.7936
H	−0.6253	1.6921	−0.9966	H	0.6006	−0.1376	−2.2029	H	−0.5022	1.3026	−0.9334
H	−4.2138	0.6311	−1.4027	H	2.6046	2.997	−1.8177	H	−4.0383	0.2281	−1.4086
H	−1.4772	0.7682	−4.5254	H	4.2779	−0.8017	−1.9975	H	−1.2322	0.4029	−4.4562
H	0.4661	0.6974	−2.9287	H	2.0945	−1.8579	−1.3273	H	0.6564	0.2403	−2.8398
H	−2.3618	0.6194	0.3065	H	0.3835	2.0853	−1.1414	H	−2.2517	0.1278	0.33
C	−3.3104	−0.7455	−3.4298	C	4.4064	1.4677	−0.728	C	−3.7234	0.845	−3.9072
H	−2.7158	−1.5191	−2.9152	H	3.9303	1.2125	0.2365	H	−3.5249	0.5521	−4.9431
H	−4.3662	−0.9217	−3.1977	H	4.6399	2.5377	−0.7096	H	−4.7234	0.4892	−3.6392
H	1.5438	2.3628	−1.5898	H	1.3165	−0.3357	3.4331	H	1.6793	1.9958	−1.5938
H	−3.1775	−0.8813	−4.5085	H	5.3519	0.9181	−0.7853	H	−3.7276	1.9519	−3.8571
**M0023**	**X**	**Y**	**Z**	**M0042**	**X**	**Y**	**Z**	**M0047**	**X**	**Y**	**Z**
C	1.0434	0.2614	0.4881	C	−0.3471	0.0866	1.1005	C	1.0283	0.246	0.4729
C	2.2788	1.9996	2.2646	C	−1.3379	1.8729	2.9823	C	2.2751	1.8808	2.3408
C	0.5298	0.3931	1.7826	C	−1.4683	0.8635	0.7818	C	0.5117	0.3164	1.7729
C	2.1879	0.9653	0.0795	C	0.2752	0.1805	2.3583	C	2.1747	0.9709	0.1008
C	2.7918	1.8369	0.9824	C	−0.2313	1.0857	3.2871	C	2.7836	1.7889	1.0486
C	1.1629	1.2765	2.6591	C	−1.9504	1.7564	1.7429	C	1.1536	1.1461	2.697
H	3.6753	2.3913	0.6769	H	0.2403	1.1675	4.2624	H	3.6711	2.3524	0.774
H	0.7775	1.3844	3.6703	H	−2.8265	2.3576	1.513	H	0.7697	1.2016	3.7126
H	2.7584	2.6833	2.959	H	−1.7258	2.5712	3.7177	H	2.7625	2.5188	3.0719
C	−0.6485	−0.4172	2.2687	C	−2.1928	0.7373	−0.5377	C	−0.6774	−0.5026	2.2177
H	−0.8848	−1.1949	1.5384	H	−3.2574	0.5679	−0.336	H	−0.9131	−1.2526	1.4595
H	−0.3533	−0.9352	3.1894	H	−1.8326	−0.1436	−1.0736	H	−0.4001	−1.0523	3.1254
C	−1.8924	0.4316	2.5493	C	−2.0423	1.9806	−1.4204	C	−1.9149	0.3521	2.5108
H	−2.2262	0.9505	1.6447	H	−2.4721	2.864	−0.9368	H	−2.229	0.9056	1.6201
H	−2.7181	−0.1934	2.9044	H	−2.5487	1.8391	−2.3803	H	−2.7525	−0.275	2.832
H	−1.6927	1.1943	3.3096	H	−0.988	2.1963	−1.6216	H	−1.7166	1.085	3.2998
C	2.7703	0.7609	−1.2962	C	1.4258	−0.7235	2.7188	C	2.7696	0.8155	−1.2749
H	2.0162	0.8682	−2.083	H	2.1912	−0.7524	1.9379	H	2.0198	0.9208	−2.0648
H	3.1956	−0.2446	−1.3986	H	1.8981	−0.3995	3.6498	H	3.2174	−0.1783	−1.39
N	0.3958	−0.5982	−0.4627	N	0.1781	−0.8424	0.1478	N	0.4042	−0.5884	−0.5071
C	0.9033	−1.8228	−0.817	C	−0.0899	−2.1906	0.1983	C	0.8767	−1.8391	−0.8296

C	1.9182	−2.4807	0.0399	C	−1.1618	−2.6862	1.109	C	1.9254	−2.4651	0.0262
C	2.6971	−3.5518	−0.2844	C	−2.0614	−2.183	2.013	C	2.6449	−2.1731	1.1559
C	3.483	−3.8407	0.8681	C	−2.8093	−3.2917	2.502	C	3.4784	−3.2993	1.4088
C	3.1078	−2.933	1.8085	C	−2.3138	−4.3817	1.8587	C	3.2084	−4.1867	0.4154
O	2.1646	−2.1015	1.3228	O	−1.3257	−4.0349	1.0211	O	2.2791	−3.7015	−0.4206
H	2.6888	−4.0605	−1.2365	H	−2.1871	−1.1544	2.309	H	2.5996	−1.2734	1.7472
H	4.2205	−4.6211	0.9853	H	−3.6058	−3.2722	3.231	H	4.1821	−3.4235	2.2186
H	3.4078	−2.7524	2.8291	H	−2.5528	−5.4334	1.8913	H	3.5838	−5.1702	0.1787
O	0.5421	−2.4125	−1.8295	O	0.5169	−2.9896	−0.5008	O	0.4416	−2.4593	−1.7894
C	−0.6381	−0.0521	−1.3729	C	1.2545	−0.4384	−0.7842	C	−0.6347	−0.042	−1.4092
C	−1.8942	1.9143	−2.2638	C	1.9921	−0.1575	−3.1653	C	−1.8784	1.9381	−2.2942
C	−2.9987	−0.2109	−2.2073	C	2.9741	1.2331	−1.4804	C	−2.9992	−0.1784	−2.2355
N	−3.1771	1.2354	−2.1492	N	2.6027	1.1372	−2.8843	N	−3.1662	1.2696	−2.1771
C	−1.9871	−0.758	−1.189	C	1.8274	0.949	−0.4917	C	−1.9915	−0.733	−1.2177
C	−0.8326	1.4588	−1.2454	C	0.8075	−0.5036	−2.2507	C	−0.8173	1.4712	−1.2801
H	−1.5094	1.732	−3.2773	H	2.7672	−0.927	−3.0453	H	−1.4979	1.7549	−3.3087
H	−2.6543	−0.4666	−3.2191	H	3.7806	0.5093	−1.2978	H	−2.6578	−0.4367	−3.2473
H	−2.3503	−0.5912	−0.1674	H	1.0475	1.7135	−0.5863	H	−2.3517	−0.5568	−0.1971
H	−1.1463	1.7216	−0.2286	H	−0.0135	0.2038	−2.4172	H	−1.127	1.7337	−0.2618
H	−0.2927	−0.2693	−2.3924	H	2.0572	−1.1767	−0.6568	H	−0.2978	−0.2651	−2.4302
H	−2.0583	2.9935	−2.1738	H	1.6824	−0.1783	−4.2155	H	−2.0326	3.0183	−2.2004
H	−3.9744	−0.6901	−2.0723	H	3.3891	2.2292	−1.2935	H	−3.9781	−0.6501	−2.0995
H	−1.8614	−1.8347	−1.3296	H	2.2123	1.0185	0.5309	H	−1.8768	−1.8107	−1.3539
H	0.1009	1.999	−1.4345	H	0.4407	−1.5077	−2.4766	H	0.1184	2.0067	−1.4708
C	−3.9733	1.678	−1.0208	C	1.8236	2.2644	−3.3591	C	−3.9561	1.7175	−1.0463
H	−3.5404	1.4632	−0.0275	H	0.8288	2.3784	−2.8927	H	−3.5213	1.4999	−0.0547
H	−4.1258	2.7605	−1.0888	H	1.6725	2.1675	−4.4393	H	−4.1023	2.8006	−1.1146
H	3.5688	1.4822	−1.4901	H	1.0747	−1.7522	2.8598	H	3.5528	1.5589	−1.4456
H	−4.9564	1.1973	−1.0632	H	2.3818	3.1894	−3.1806	H	−4.942	1.243	−1.086
**M0039**	**X**	**Y**	**Z**	**M0018**	**X**	**Y**	**Z**	**M0044**	**X**	**Y**	**Z**
C	−0.6112	0.3289	0.6961	C	−0.4733	0.7375	1.1197	C	0.9347	0.4058	0.4905
C	−1.756	2.0002	2.5982	C	−1.2009	2.4765	3.1645	C	2.307	1.9398	2.3572
C	−1.7556	1.0676	0.3616	C	−1.3214	1.8213	0.8334	C	0.3843	0.5825	1.7687
C	−0.0208	0.434	1.9659	C	0.0099	0.5136	2.4187	C	2.1415	1.0238	0.124
C	−0.6107	1.2741	2.9086	C	−0.3719	1.3974	3.4305	C	2.8202	1.7832	1.0756
C	−2.3175	1.8977	1.3337	C	−1.6688	2.6826	1.8722	C	1.0939	1.3522	2.6926
H	−0.1679	1.3564	3.8976	H	−0.0033	1.2321	4.4396	H	3.7599	2.257	0.805
H	−3.2055	2.4742	1.0857	H	−2.3236	3.5245	1.6621	H	0.6829	1.4912	3.6893
H	−2.205	2.6507	3.3428	H	−1.4818	3.1583	3.9617	H	2.8458	2.5323	3.0905
C	−2.4015	0.9922	−1.0047	C	−1.905	2.0549	−0.5433	C	−0.9243	−0.0502	2.1834
H	−1.7101	0.5525	−1.7292	H	−1.1188	2.0446	−1.3049	H	−1.2998	0.484	3.0633
H	−2.6013	2.0155	−1.3456	H	−2.3303	3.0645	−0.5664	H	−1.6714	0.0919	1.3977
C	−3.7161	0.2011	−1.0124	C	−2.9918	1.0465	−0.9326	C	−0.8173	−1.5431	2.5106
H	−3.5527	−0.8449	−0.7409	H	−2.5837	0.0365	−1.0165	H	−0.0768	−1.7182	3.2983
H	−4.1703	0.2283	−2.0078	H	−3.432	1.3171	−1.8981	H	−1.7819	−1.9269	2.8581
H	−4.4321	0.624	−0.2995	H	−3.789	1.0253	−0.1835	H	−0.5171	−2.1217	1.6337
C	1.2121	−0.3561	2.3178	C	0.8743	−0.6715	2.7718	C	2.715	0.8465	−1.2582
H	1.158	−1.3756	1.9259	H	1.5242	−0.9786	1.9496	H	1.9515	0.9638	−2.0337
H	2.111	0.1071	1.8936	H	1.5037	−0.4401	3.6359	H	3.1475	−0.1533	−1.3808
N	−0.0244	−0.5439	−0.276	N	−0.0678	−0.1315	0.0494	N	0.2661	−0.4206	−0.4724
C	−0.5909	−1.7511	−0.6169	C	−0.3315	−1.478	0.0502	C	0.7934	−1.6305	−0.8719
C	−1.6191	−2.3418	0.2833	C	−1.4522	−2.0007	0.8673	C	1.8411	−2.2801	−0.0347
C	−2.1063	−2.14	1.5465	C	−1.7267	−3.3065	1.149	C	2.4509	−2.0848	1.1764
C	−3.0902	−3.1458	1.7669	C	−2.9101	−3.3057	1.9426	C	3.362	−3.1652	1.3512
C	−3.1309	−3.8781	0.6225	C	−3.267	−1.9997	2.0726	C	3.2405	−3.9326	0.2356

O	−2.2506	−3.4101	−0.2764	O	−2.3959	−1.1984	1.4287	O	2.3305	−3.416	−0.604
H	−1.8173	−1.3656	2.2388	H	−1.1411	−4.1502	0.817	H	2.28	−1.2751	1.8677
H	−3.6851	−3.2961	2.6557	H	−3.4272	−4.1587	2.3568	H	4.0163	−3.3403	2.1925
H	−3.7134	−4.7307	0.3091	H	−4.0814	−1.4979	2.5715	H	3.7182	−4.8428	−0.0931
O	−0.2569	−2.3567	−1.6249	O	0.3061	−2.2873	−0.616	O	0.4088	−2.194	−1.8861
C	1.0154	−0.0399	−1.2027	C	0.9551	0.4022	−0.8773	C	−0.7436	0.1591	−1.3907
C	2.3821	1.8945	−2.0182	C	3.3815	0.6078	−1.5018	C	−2.0242	2.1706	−2.1528
C	3.3574	−0.2883	−2.0794	C	1.7435	0.6922	−3.2407	C	−3.0753	0.0283	−2.3179
N	3.6228	1.138	−1.9275	N	3.0977	0.2816	−2.8928	N	−3.2923	1.4563	−2.1133
C	2.3205	−0.8391	−1.0902	C	0.6568	0.0772	−2.3454	C	−2.0768	−0.5995	−1.3349
C	1.2933	1.4533	−1.0245	C	2.3867	0.0054	−0.4949	C	−0.9715	1.6529	−1.1568
H	1.9959	1.7815	−3.0408	H	3.3651	1.7034	−1.408	H	−1.6223	2.0812	−3.1718
H	2.996	−0.4533	−3.1037	H	1.7006	1.7888	−3.1677	H	−2.7018	−0.1095	−3.342
H	2.7039	−0.7746	−0.0658	H	0.6215	−1.0049	−2.482	H	−2.4818	−0.5684	−0.3171
H	1.6173	1.6638	0.0018	H	2.4663	−1.0851	−0.4744	H	−1.3081	1.8372	−0.13
H	0.6257	−0.1976	−2.218	H	0.8868	1.491	−0.762	H	−0.3468	0.0294	−2.4076
H	2.606	2.9573	−1.8762	H	4.4007	0.2832	−1.2646	H	−2.213	3.2348	−1.975
H	4.3024	−0.8337	−1.9835	H	1.5565	0.4352	−4.2891	H	−4.0407	−0.4856	−2.2591
H	2.1222	−1.8919	−1.3045	H	−0.3274	0.4732	−2.6226	H	−1.9084	−1.6477	−1.5909
H	0.3869	2.0439	−1.1965	H	2.6169	0.3807	0.5094	H	−0.0411	2.2165	−1.2876
C	4.4116	1.4592	−0.7531	C	3.3826	−1.1047	−3.2263	C	−4.0896	1.7584	−0.9392
H	3.9331	1.2187	0.2141	H	2.7833	−1.8485	−2.6744	H	−3.6418	1.4525	0.0239
H	4.6389	2.5305	−0.7529	H	4.4401	−1.3088	−3.0267	H	−4.2699	2.8377	−0.8931
H	1.3465	−0.4048	3.4019	H	0.2557	−1.5384	3.0344	H	3.5061	1.5777	−1.4448
H	5.3604	0.9142	−0.7965	H	3.2082	−1.2596	−4.2964	H	−5.0604	1.2593	−1.0262
**M0028**	**X**	**Y**	**Z**	**M0021**	**X**	**Y**	**Z**	**M0016**	**X**	**Y**	**Z**
C	0.7366	0.7988	0.81	C	−0.516	0.3684	1.1597	C	−0.6229	0.5763	1.1378
C	1.6815	2.4653	2.8258	C	−1.4695	2.1	3.108	C	−1.4447	2.3966	3.0722
C	0.252	0.6343	2.1169	C	−1.4805	1.3334	0.8108	C	−1.5487	1.5744	0.7967
C	1.6925	1.785	0.5046	C	−0.037	0.252	2.4733	C	−0.133	0.4546	2.4488
C	2.1558	2.6101	1.526	C	−0.5312	1.1336	3.4368	C	−0.558	1.3806	3.4023
C	0.7427	1.4859	3.1111	C	−1.9424	2.1955	1.8034	C	−1.9406	2.4833	1.7784
H	2.8993	3.3696	1.3003	H	−0.1698	1.0541	4.4588	H	−0.1829	1.2985	4.4192
H	0.3715	1.3758	4.1268	H	−2.6901	2.9443	1.5637	H	−2.6606	3.2572	1.5228
H	2.0472	3.1163	3.6139	H	−1.8405	2.7798	3.8694	H	−1.7599	3.1111	3.8268
C	−0.7529	−0.4295	2.5073	C	−2.0233	1.3809	−0.6031	C	−2.1681	1.6636	−0.5793
H	−1.606	0.0629	2.9912	H	−2.1326	0.3492	−0.9568	H	−3.2494	1.7976	−0.456
H	−1.1516	−0.9287	1.6221	H	−1.2761	1.837	−1.2667	H	−2.0332	0.7129	−1.1019
C	−0.1772	−1.4812	3.4645	C	−3.35	2.1169	−0.7781	C	−1.6279	2.8154	−1.4352
H	0.1701	−1.0243	4.3969	H	−4.1203	1.7123	−0.1127	H	−1.7671	3.7777	−0.9313
H	−0.9417	−2.2224	3.7166	H	−3.703	2.0082	−1.8079	H	−2.1499	2.8572	−2.3966
H	0.6704	−2.0042	3.0138	H	−3.2561	3.189	−0.5762	H	−0.5587	2.7012	−1.64
C	2.2322	1.9282	−0.8951	C	0.9296	−0.8285	2.8847	C	0.7784	−0.6719	2.868
H	2.4637	0.9513	−1.3307	H	1.6227	−1.097	2.0849	H	1.4606	−0.9786	2.0728
H	3.1407	2.5365	−0.9004	H	1.5122	−0.5142	3.7554	H	1.3738	−0.3818	3.7385
N	0.2459	−0.0161	−0.2621	N	−0.0151	−0.5014	0.1374	N	−0.1444	−0.3127	0.1161
C	0.4471	−1.3745	−0.3163	C	−0.2144	−1.8624	0.1539	C	−0.3764	−1.6632	0.1365
C	1.5801	−1.9323	0.4686	C	−1.3427	−2.3841	0.9726	C	−1.5242	−2.1753	0.9205
C	2.7353	−1.4596	1.0268	C	−2.4768	−1.8824	1.5509	C	−1.7462	−3.4528	1.3406
C	3.4082	−2.5877	1.5766	C	−3.1756	−2.9954	2.1013	C	−3.0009	−3.4394	2.0163
C	2.6151	−3.6584	1.3028	C	−2.415	−4.0856	1.8149	C	−3.4473	−2.157	1.9421
O	1.5138	−3.2798	0.6334	O	−1.3126	−3.7344	1.1326	O	−2.5651	−1.3793	1.2831
H	3.0623	−0.4321	1.0639	H	−2.7794	−0.8475	1.5908	H	−1.0832	−4.2878	1.1718
H	4.3508	−2.5956	2.1035	H	−4.1128	−2.9807	2.6383	H	−3.5048	−4.2701	2.4881
H	2.6949	−4.7132	1.5146	H	−2.5206	−5.1388	2.025	H	−4.3375	−1.659	2.2937

O	−0.2394	−2.124	−0.9968	O	0.4633	−2.6398	−0.5006	O	0.3085	−2.4743	−0.4781
C	−0.766	0.6048	−1.1432	C	0.9542	0.0879	−0.8089	C	0.8749	0.2371	−0.805
C	−3.2015	0.9267	−1.6935	C	3.3402	0.5127	−1.4392	C	3.3038	0.474	−1.4213
C	−1.6077	1.016	−3.4713	C	1.676	0.4662	−3.1674	C	1.6777	0.5099	−3.1698
N	−2.9652	0.6377	−3.1015	N	3.0496	0.1423	−2.815	N	3.0334	0.1192	−2.8075
C	−0.5175	0.3313	−2.6314	C	0.6884	−0.2758	−2.2711	C	0.5896	−0.1001	−2.2727
C	−2.2038	0.2536	−0.7365	C	2.4158	−0.2042	−0.4572	C	2.3124	−0.1267	−0.4104
H	−3.1432	2.017	−1.5645	H	3.248	1.6115	−1.2944	H	3.2707	1.5708	−1.3475
H	−1.5258	2.1068	−3.3588	H	1.4942	1.5618	−3.1005	H	1.6228	1.6071	−3.1138
H	−0.5201	−0.7446	−2.8132	H	0.8076	−1.3512	−2.4098	H	0.5547	−1.1839	−2.4012
H	−2.3265	−0.8329	−0.7524	H	2.5884	−1.2827	−0.5013	H	2.4107	−1.2147	−0.3689
H	−0.6418	1.6839	−0.9867	H	0.8102	1.1709	−0.708	H	0.7806	1.3234	−0.6941
H	−4.2249	0.6304	−1.4389	H	4.3815	0.2501	−1.2215	H	4.326	0.169	−1.1717
H	−1.4569	0.7924	−4.533	H	1.51	0.1795	−4.2116	H	1.4991	0.2355	−4.2152
H	0.4696	0.7103	−2.9192	H	−0.3366	−0.0058	−2.5476	H	−0.3897	0.2985	−2.5617
H	−2.3923	0.5979	0.2871	H	2.6343	0.1469	0.5577	H	2.5312	0.2715	0.5873
C	−3.2999	−0.7294	−3.4686	C	3.9891	0.7507	−3.7337	C	3.3322	−1.271	−3.1129
H	−2.6914	−1.5048	−2.9747	H	3.7845	0.4075	−4.7531	H	2.7271	−2.0086	−2.5594
H	−4.3509	−0.9187	−3.2253	H	5.0099	0.4523	−3.4731	H	4.3871	−1.4664	−2.8921
H	1.5069	2.4113	−1.5602	H	0.3867	−1.741	3.1592	H	0.1928	−1.5566	3.1466
H	−3.1815	−0.846	−4.5509	H	3.9437	1.8581	−3.7279	H	3.1769	−1.4431	−4.1833
**M0035**	**X**	**Y**	**Z**	**M0024**	**X**	**Y**	**Z**	**M0020**	**X**	**Y**	**Z**
C	0.3771	−0.9192	0.4694	C	−0.2416	0.2193	1.0456	C	0.8875	0.7358	0.8032
C	2.0297	−2.1647	2.321	C	−1.0652	1.9981	3.0117	C	1.8658	2.4424	2.7644
C	0.5049	−0.3692	1.7495	C	−1.2799	1.1135	0.7652	C	0.4187	0.603	2.1183
C	1.0612	−2.0908	0.1049	C	0.388	0.1988	2.3013	C	1.8388	1.7122	0.4599
C	1.8819	−2.7045	1.0477	C	−0.0415	1.0947	3.2767	C	2.3091	2.566	1.4516
C	1.346	−1.0077	2.6637	C	−1.6745	2.0045	1.766	C	0.9379	1.466	3.089
H	2.4174	−3.6106	0.7769	H	0.4377	1.0887	4.2521	H	3.0474	3.3215	1.1964
H	1.4611	−0.5845	3.6588	H	−2.4753	2.71	1.5573	H	0.604	1.3653	4.1183
H	2.6825	−2.6464	3.0431	H	−1.3835	2.699	3.7778	H	2.2519	3.1043	3.534
C	−0.2619	0.8573	2.1846	C	−2.0152	1.1158	−0.5544	C	−0.6029	−0.4355	2.536
H	0.4366	1.5798	2.6255	H	−1.4659	0.5218	−1.2862	H	−0.9291	−1.0225	1.6751
H	−0.7144	1.3397	1.3169	H	−2.0582	2.1422	−0.9399	H	−0.1245	−1.1442	3.2239
C	−1.3646	0.5302	3.1989	C	−3.4383	0.5578	−0.4307	C	−1.8397	0.1705	3.2083
H	−0.95	0.0644	4.0993	H	−3.4136	−0.4808	−0.0904	H	−2.3346	0.8869	2.5441
H	−1.8934	1.4402	3.5003	H	−3.9517	0.5939	−1.397	H	−2.5598	−0.6143	3.4605
H	−2.0895	−0.1642	2.7655	H	−4.0291	1.1337	0.2897	H	−1.5851	0.6998	4.1319
C	0.9017	−2.6822	−1.2727	C	1.4954	−0.7812	2.5949	C	2.3704	1.808	−0.9455
H	1.0511	−1.9333	−2.0578	H	2.2658	−0.7706	1.8168	H	2.6328	0.8171	−1.3286
H	−0.1029	−3.0978	−1.4146	H	1.9748	−0.5491	3.5498	H	3.2619	2.4403	−0.9798
N	−0.4329	−0.2635	−0.5208	N	0.2288	−0.6834	0.0303	N	0.3964	−0.1238	−0.2367
C	−1.6307	−0.7772	−0.9509	C	−0.0067	−2.0347	0.0821	C	0.6042	−1.4791	−0.2206
C	−2.3388	−1.8067	−0.1531	C	−1.0697	−2.5815	0.9595	C	1.7403	−2.0099	0.5685
C	−3.4336	−2.524	−0.5369	C	−1.3	−3.8967	1.2373	C	1.924	−3.2809	1.0242
C	−3.7688	−3.3661	0.562	C	−2.4314	−3.9336	2.1021	C	3.1869	−3.2903	1.6848
C	−2.8522	−3.0952	1.5291	C	−2.8024	−2.6378	2.2816	C	3.6757	−2.0268	1.5669
O	−1.9803	−2.1546	1.1123	O	−1.9886	−1.808	1.5981	O	2.8133	−1.2403	0.8924
H	−3.9281	−2.4373	−1.4923	H	−0.7216	−4.7227	0.8524	H	1.2311	−4.0977	0.8892
H	−4.5815	−4.0747	0.625	H	−2.9053	−4.8046	2.5302	H	3.6678	−4.1232	2.1763
H	−2.6896	−3.4712	2.5273	H	−3.5898	−2.1593	2.8429	H	4.5859	−1.5486	1.894
O	−2.1603	−0.4098	−1.9942	O	0.639	−2.8348	−0.5864	O	−0.0975	−2.2782	−0.8318
C	0.1801	0.7466	−1.42	C	1.3375	−0.2676	−0.8653	C	−0.6242	0.4462	−1.141
C	2.2147	2.0177	−2.1297	C	2.1644	−0.0008	−3.221	C	−3.0608	0.719	−1.7089
C	0.0803	3.0725	−2.3614	C	2.9956	1.4813	−1.5386	C	−1.4654	0.7327	−3.488

N	1.5041	3.2886	−2.1298	N	2.6751	1.339	−2.9519	N	−2.8212	0.3651	−3.1014
C	−0.5674	2.086	−1.3786	C	1.8345	1.1511	−0.5838	C	−0.373	0.0987	−2.6126
C	1.6647	0.9786	−1.1365	C	0.9647	−0.397	−2.3483	C	−2.0585	0.1008	−0.7203
H	2.1558	1.6019	−3.1454	H	2.9828	−0.7132	−3.0474	H	−3.0117	1.815	−1.6323
H	−0.0389	2.6877	−3.3837	H	3.8376	0.8104	−1.3175	H	−1.3897	1.8285	−3.4304
H	−0.5287	2.4978	−0.3634	H	1.02	1.8727	−0.7183	H	−0.3706	−0.986	−2.7377
H	1.8048	1.3301	−0.1075	H	0.1127	0.2553	−2.5732	H	−2.1694	−0.9864	−0.6853
H	0.0794	0.3518	−2.4405	H	2.1639	−0.9674	−0.679	H	−0.5082	1.5327	−1.0367
H	3.2736	2.2065	−1.922	H	1.9018	−0.0694	−4.2821	H	−4.0824	0.427	−1.4416
H	−0.4323	4.0397	−2.3228	H	3.3454	2.5035	−1.3582	H	−1.3125	0.4575	−4.5374
H	−1.6168	1.9315	−1.639	H	2.1787	1.255	0.4506	H	0.6129	0.467	−2.9181
H	2.2367	0.0495	−1.2316	H	0.6671	−1.4253	−2.5651	H	−2.2509	0.4898	0.2863
C	1.7835	4.1067	−0.9641	C	1.8184	2.3954	−3.4582	C	−3.1512	−1.0175	−3.408
H	1.4537	3.6787	−0.0003	H	0.8089	2.4351	−3.0108	H	−2.5465	−1.7696	−2.8736
H	2.8623	4.2833	−0.8973	H	1.6979	2.2718	−4.5396	H	−4.2041	−1.1968	−3.1644
H	1.6214	−3.4895	−1.4331	H	1.1149	−1.8076	2.6542	H	1.6325	2.2374	−1.6337
H	1.2915	5.0781	−1.0814	H	2.2976	3.3646	−3.2828	H	−3.0235	−1.1849	−4.4828
**M0027**	**X**	**Y**	**Z**	**M0041**	**X**	**Y**	**Z**	**M0025**	**X**	**Y**	**Z**
C	−0.6095	0.5576	1.1392	C	0.1839	−0.1153	1.0665	C	0.6542	0.1965	0.8701
C	−1.5177	2.3409	3.0732	C	1.1908	−1.8296	2.9972	C	1.6638	1.9183	2.7992
C	−1.5585	1.533	0.7895	C	1.2924	−0.9176	0.7537	C	0.108	0.172	2.1603
C	−0.1295	0.4501	2.4566	C	−0.4235	−0.1585	2.3307	C	1.7101	1.0592	0.5376
C	−0.5977	1.3559	3.4084	C	0.0963	−1.026	3.2897	C	2.1993	1.9204	1.5151
C	−1.997	2.4207	1.7729	C	1.7817	−1.7751	1.7406	C	0.6341	1.0459	3.1161
H	−0.2338	1.281	4.4298	H	−0.3621	−1.0684	4.274	H	3.0196	2.5901	1.2711
H	−2.7373	3.1731	1.5114	H	2.6408	−2.4041	1.532	H	0.2248	1.0326	4.1236
H	−1.8696	3.0375	3.8283	H	1.5872	−2.5023	3.752	H	2.0601	2.5905	3.5545
C	−2.1595	1.6193	−0.5954	C	1.9458	−0.8231	−0.6087	C	−1.0267	−0.7475	2.5628
H	−3.2438	1.7456	−0.4896	H	1.8115	0.1961	−0.9827	H	−1.1614	−1.5522	1.8365
H	−2.0078	0.672	−1.1199	H	1.4017	−1.4708	−1.3105	H	−0.7556	−1.2314	3.5087
C	−1.6159	2.7797	−1.4375	C	3.4314	−1.1799	−0.6479	C	−2.3627	−0.0163	2.7425
H	−1.7741	3.7383	−0.9321	H	3.9964	−0.5924	0.0836	H	−2.7177	0.3983	1.7939
H	−2.1212	2.8195	−2.4078	H	3.8398	−0.9675	−1.6403	H	−3.1287	−0.7049	3.1131
H	−0.5423	2.6764	−1.6229	H	3.6096	−2.2403	−0.443	H	−2.2714	0.8089	3.4565
C	0.8066	−0.6514	2.8833	C	−1.5881	0.7393	2.6583	C	2.323	1.0332	−0.8373
H	1.4965	−0.9462	2.0907	H	−2.354	0.7158	1.8768	H	2.497	0.0053	−1.1667
H	1.3922	−0.3454	3.7549	H	−2.0524	0.4442	3.603	H	3.2761	1.5693	−0.8467
N	−0.1125	−0.3278	0.1277	N	−0.3468	0.7792	0.0829	N	0.1234	−0.6691	−0.1452
C	−0.3516	−1.681	0.1315	C	−0.1341	2.1377	0.1264	C	0.2847	−2.0319	−0.0776
C	−1.4869	−2.1844	0.9513	C	0.929	2.6738	1.023	C	1.4113	−2.592	0.7034
C	−2.6012	−1.6634	1.5505	C	1.9202	2.1947	1.8394	C	1.5576	−3.869	1.1562
C	−3.3209	−2.7662	2.0934	C	2.613	3.3309	2.3466	C	2.8211	−3.9188	1.8133
C	−2.5889	−3.8701	1.786	C	1.9895	4.4122	1.8084	C	3.3476	−2.6706	1.6972
O	−1.4866	−3.537	1.0952	O	0.9801	4.0342	1.0097	O	2.5068	−1.8557	1.0294
H	−2.8793	−0.6225	1.6052	H	2.1422	1.1636	2.0616	H	0.8396	−4.6635	1.0203
H	−4.2523	−2.7365	2.6396	H	3.4555	3.3356	3.0225	H	3.2782	−4.7674	2.3008
H	−2.7174	−4.923	1.9849	H	2.1442	5.4766	1.8962	H	4.2727	−2.2214	2.0235
O	0.3025	−2.471	−0.5351	O	−0.7745	2.91	−0.5722	O	−0.4721	−2.8138	−0.6422
C	0.899	0.2293	−0.7987	C	−1.3929	0.3204	−0.8599	C	−0.9831	−0.1965	−1.0101
C	3.3266	0.4574	−1.4307	C	−2.0697	−0.0352	−3.2543	C	−1.8418	0.0457	−3.3618
C	1.6878	0.5382	−3.1644	C	−2.9613	−1.4929	−1.5811	C	−2.4881	1.6688	−1.7288
N	3.0431	0.1302	−2.8208	N	−2.5529	−1.3832	−2.9744	N	−2.2163	1.438	−3.1408
C	0.6019	−0.0808	−2.2706	C	−1.8714	−1.1025	−0.5674	C	−1.3353	1.276	−0.7891
C	2.3374	−0.1521	−0.4229	C	−0.9408	0.4287	−2.3227	C	−0.67	−0.4337	−2.4933
H	3.3042	1.553	−1.3368	H	−2.9208	0.6523	−3.1541	H	−2.7218	−0.5754	−3.1448

H	1.6423	1.6348	−3.0878	H	−3.8323	−0.8389	−1.4357	H	−3.3809	1.0866	−1.46
H	0.5594	−1.1617	−2.4161	H	−1.0339	−1.8073	−0.6304	H	−0.4657	1.9135	−0.984
H	2.4278	−1.2415	−0.3999	H	−0.0497	−0.1888	−2.4784	H	0.2479	0.1046	−2.7578
H	0.8149	1.3146	−0.669	H	−2.2426	1.0045	−0.7314	H	−1.8646	−0.8024	−0.7537
H	4.3477	0.1387	−1.194	H	−1.7441	0.0111	−4.299	H	−1.6093	−0.0914	−4.4233
H	1.4985	0.2847	−4.2133	H	−3.2957	−2.5186	−1.3913	H	−2.7402	2.7252	−1.5865
H	−0.3761	0.3278	−2.5492	H	−2.2753	−1.1846	0.4475	H	−1.6302	1.4643	0.2472
H	2.5685	0.2278	0.5791	H	−0.6726	1.4641	−2.5456	H	−0.4921	−1.4971	−2.6677
C	3.3281	−1.2568	−3.1532	C	−1.6342	−2.4263	−3.3902	C	−1.2791	2.3876	−3.711
H	2.7219	−1.9992	−2.6075	H	−0.6573	−2.4244	−2.8729	H	−0.2602	2.3564	−3.2834
H	4.3833	−1.4642	−2.9451	H	−1.4411	−2.3278	−4.4635	H	−1.1915	2.2052	−4.7872
H	0.2365	−1.5459	3.1625	H	−1.2618	1.7811	2.7556	H	1.6711	1.5062	−1.5812
H	3.1623	−1.4089	−4.2251	H	−2.097	−3.4044	−3.2206	H	−1.6658	3.4029	−3.5729
**M0029**	**X**	**Y**	**Z**	**M0040**	**X**	**Y**	**Z**	**M0033**	**X**	**Y**	**Z**
C	0.8741	0.7143	0.8053	C	0.6351	0.1765	0.8074	C	−0.4691	0.5786	1.1055
C	1.9422	2.3961	2.7442	C	1.7541	1.848	2.7224	C	−1.4224	2.2991	3.0644
C	0.4202	0.6073	2.1292	C	0.0907	0.1977	2.1009	C	−1.4391	1.5409	0.7643
C	1.8483	1.6619	0.4412	C	1.7233	0.9933	0.4584	C	0.0161	0.4599	2.417
C	2.3683	2.5001	1.424	C	2.27	1.8281	1.4309	C	−0.4781	1.3359	3.3857
C	0.9812	1.4572	3.087	C	0.6753	1.0393	3.0501	C	−1.9008	2.3975	1.7618
H	3.1272	3.2293	1.154	H	3.1173	2.4584	1.1755	H	−0.1124	1.2542	4.4057
H	0.6603	1.3719	4.1216	H	0.282	1.0523	4.0629	H	−2.6521	3.144	1.5276
H	2.3658	3.0447	3.5051	H	2.1977	2.4935	3.4743	H	−1.7933	2.9742	3.8296
C	−0.6167	−0.4048	2.5684	C	−1.0795	−0.675	2.5034	C	−1.9868	1.593	−0.6475
H	−0.9402	−1.0139	1.7222	H	−1.3994	−1.2931	1.6617	H	−2.084	0.5634	−1.0095
H	−0.1504	−1.0958	3.2815	H	−0.744	−1.3699	3.283	H	−1.2469	2.0643	−1.3084
C	−1.8497	0.2384	3.2123	C	−2.2858	0.1194	3.0169	C	−3.3233	2.3137	−0.8119
H	−2.3334	0.936	2.5206	H	−2.6703	0.7959	2.2469	H	−4.0853	1.8968	−0.1448
H	−2.5793	−0.5289	3.4885	H	−3.0942	−0.5604	3.3026	H	−3.68	2.2059	−1.8404
H	−1.5928	0.7975	4.1176	H	−2.0319	0.7243	3.893	H	−3.2413	3.3856	−0.6051
C	2.3531	1.7459	−0.9752	C	2.3106	0.954	−0.9274	C	0.9902	−0.6167	2.8216
H	2.5441	0.7489	−1.3838	H	2.376	−0.0713	−1.3013	H	1.6842	−0.8752	2.0197
H	3.2784	2.3261	−1.0234	H	3.3108	1.3951	−0.9377	H	1.5715	−0.303	3.6932
N	0.3588	−0.1491	−0.2142	N	0.0916	−0.7077	−0.1785	N	0.0313	−0.2861	0.0783
C	0.5891	−1.5046	−0.2248	C	0.2798	−2.0705	−0.1183	C	−0.1545	−1.6486	0.0966
C	1.7088	−2.0322	0.6035	C	1.3589	−2.6124	0.754	C	−1.275	−2.1816	0.9179
C	2.8039	−1.5352	1.2578	C	2.3866	−2.1385	1.5265	C	−2.4128	−1.69	1.4976
C	3.5164	−2.6591	1.7648	C	3.0706	−3.2784	2.0354	C	−3.0989	−2.8082	2.0513
C	2.8005	−3.7504	1.3833	C	2.4109	−4.3564	1.5345	C	−2.329	−3.8914	1.7622
O	1.7148	−3.3901	0.6817	O	1.3838	−3.9731	0.762	O	−1.2307	−3.5308	1.0795
H	3.0773	−0.4981	1.3729	H	2.6361	−1.1081	1.7214	H	−2.7256	−0.6582	1.537
H	4.4316	−2.6498	2.3382	H	3.9329	−3.2875	2.6855	H	−4.034	−2.8014	2.5915
H	2.9309	−4.8109	1.5327	H	2.5483	−5.4217	1.6357	H	−2.4245	−4.9453	1.9724
O	−0.0667	−2.2813	−0.9051	O	−0.4043	−2.8415	−0.776	O	0.529	−2.4216	−0.5598
C	−0.656	0.4252	−1.1227	C	−0.9884	−0.2513	−1.0819	C	1.0016	0.3036	−0.8699
C	−3.0919	0.6847	−1.7029	C	−1.7656	0.0653	−3.4498	C	3.412	0.6354	−1.5253
C	−1.4825	0.7699	−3.4669	C	−2.5634	1.5689	−1.7681	C	1.7554	0.6002	−3.2465
N	−2.8378	0.3776	−3.1038	N	−2.2198	1.4247	−3.1757	N	3.1325	0.2671	−2.9063
C	−0.3903	0.1201	−2.6023	C	−1.4334	1.1836	−0.7973	C	0.7102	−0.0576	−2.3318
C	−2.0895	0.0478	−0.726	C	−0.6027	−0.3969	−2.56	C	2.4596	0.0021	−0.4969
H	−3.0561	1.778	−1.5913	H	−2.6195	−0.6088	−3.2985	H	3.34	1.7301	−1.4536
H	−1.4186	1.864	−3.3753	H	−3.435	0.9302	−1.5697	H	1.6551	1.6938	−3.1897
H	−0.3773	−0.9598	−2.7574	H	−0.5922	1.8772	−0.9104	H	0.7309	−1.142	−2.4533
H	−2.1865	−1.0412	−0.7268	H	0.2914	0.2011	−2.7675	H	2.6026	−1.0809	−0.4525
H	−0.5579	1.5106	−0.9909	H	−1.8451	−0.9158	−0.9027	H	0.8616	1.387	−0.7676

H	−4.1114	0.3722	−1.4522	H	−1.4869	−0.0055	−4.5064	H	4.4482	0.3684	−1.2911
H	−1.3171	0.5281	−4.5224	H	−2.8752	2.6026	−1.5844	H	1.5717	0.3167	−4.2885
H	0.593	0.5051	−2.8934	H	−1.7896	1.2881	0.2325	H	−0.2933	0.2844	−2.6079
H	−2.2975	0.4012	0.2903	H	−0.361	−1.4409	−2.7712	H	2.6776	0.4128	0.4958
C	−3.1513	−0.9986	−3.4549	C	−1.3091	2.447	−3.6557	C	3.4812	−1.111	−3.2149
H	−2.542	−1.7599	−2.9401	H	−0.3086	2.4416	−3.1861	H	2.9098	−1.871	−2.6566
H	−4.2036	−1.196	−3.2243	H	−1.1696	2.3255	−4.7348	H	4.5448	−1.2661	−3.0052
H	1.6236	2.2304	−1.6346	H	1.6924	1.5188	−1.6351	H	0.4538	−1.5339	3.0923
H	−3.0152	−1.1309	−4.5334	H	−1.7513	3.434	−3.4837	H	3.3223	−1.2877	−4.2839
**M0030**	**X**	**Y**	**Z**	**M0036**	**X**	**Y**	**Z**	**M0038**	**X**	**Y**	**Z**
C	0.4082	1.0922	1.3886	C	0.5751	−0.8467	−0.2587	C	−0.9059	0.3907	1.5091
C	2.1257	2.3807	3.1464	C	1.5421	−3.018	1.1759	C	−1.9324	2.166	3.3784
C	1.0141	2.2928	0.9981	C	−0.1987	−1.3755	0.784	C	−2.05	1.1272	1.19
C	0.6274	0.5406	2.6571	C	1.7952	−1.4345	−0.6334	C	−0.2993	0.4885	2.7708
C	1.4948	1.2015	3.5253	C	2.2715	−2.5153	0.1051	C	−0.8215	1.3901	3.6944
C	1.8815	2.9223	1.8923	C	0.3132	−2.4607	1.4983	C	−2.5435	2.0242	2.1417
H	1.6732	0.785	4.513	H	3.2189	−2.971	−0.1697	H	−0.3545	1.482	4.6715
H	2.3627	3.8512	1.5961	H	−0.27	−2.887	2.3093	H	−3.4267	2.6116	1.9013
H	2.8023	2.8799	3.8335	H	1.9235	−3.8601	1.7457	H	−2.326	2.8724	4.1034
C	0.705	2.9462	−0.329	C	−1.597	−0.8748	1.0676	C	−2.8103	0.9271	−0.0983
H	0.4436	2.1913	−1.077	H	−1.7668	0.0404	0.4975	H	−2.1653	0.4707	−0.8512
H	1.6026	3.4579	−0.6957	H	−2.3004	−1.6123	0.6602	H	−3.1249	1.9017	−0.4913
C	−0.4444	3.9564	−0.2059	C	−1.9175	−0.635	2.5442	C	−4.0368	0.029	0.1118
H	−1.3431	3.4785	0.194	H	−1.2078	0.0642	2.9968	H	−3.7272	−0.9394	0.5141
H	−0.6827	4.3951	−1.1805	H	−2.9212	−0.2108	2.649	H	−4.5659	−0.1306	−0.8339
H	−0.1706	4.7673	0.4771	H	−1.8899	−1.5615	3.1266	H	−4.7364	0.4834	0.8218
C	−0.0474	−0.7363	3.082	C	2.563	−0.9334	−1.8312	C	0.8686	−0.3945	3.1273
H	−1.1024	−0.737	2.7955	H	2.8715	0.1111	−1.7184	H	1.6904	−0.3072	2.408
H	0.4303	−1.6108	2.625	H	1.957	−0.9902	−2.7424	H	1.2583	−0.1449	4.1181
N	−0.4489	0.4199	0.4454	N	0.1224	0.3083	−0.9906	N	−0.2973	−0.4873	0.5419
C	−1.7817	0.7341	0.5237	C	−0.4681	0.1715	−2.2265	C	−0.7095	−1.7939	0.5924
C	−2.7226	0.3407	−0.5555	C	−1.0508	−1.1234	−2.656	C	−0.0322	−2.8649	−0.1838
C	−4.0794	0.2558	−0.471	C	−1.4693	−1.4415	−3.9147	C	−0.4902	−4.1358	−0.3666
C	−4.5436	−0.1129	−1.7674	C	−1.9753	−2.7708	−3.8471	C	0.4933	−4.8164	−1.142
C	−3.4338	−0.2074	−2.5452	C	−1.8308	−3.1555	−2.5511	C	1.482	−3.9112	−1.3633
O	−2.322	0.0633	−1.8281	O	−1.2742	−2.1708	−1.8182	O	1.183	−2.7265	−0.7888
H	−4.6586	0.4447	0.4199	H	−1.4168	−0.7859	−4.7703	H	−1.422	−4.519	0.0201
H	−5.5643	−0.2776	−2.0795	H	−2.3885	−3.3607	−4.6519	H	0.4698	−5.8407	−1.4837
H	−3.2725	−0.4415	−3.586	H	−2.0641	−4.0615	−2.0138	H	2.4249	−3.9513	−1.8865
O	−2.2506	1.3535	1.4721	O	−0.5299	1.1008	−3.0241	O	−1.6562	−2.1442	1.2895
C	0.1223	−0.551	−0.5154	C	0.7495	1.6455	−0.7684	C	0.7689	0.0276	−0.3462
C	2.1689	−1.639	−1.4482	C	1.1253	1.8224	1.7672	C	1.5527	0.4609	−2.6832
C	0.1402	−2.9131	−1.3613	C	−0.8432	2.8357	0.8594	C	2.3608	1.8623	−0.9085
N	1.5901	−2.9587	−1.2515	N	0.2341	2.9712	1.8295	N	1.9844	1.7997	−2.3119
C	−0.4562	−1.9599	−0.3314	C	−0.2991	2.7525	−0.5621	C	1.2094	1.4498	0.0075
C	1.6465	−0.6371	−0.4206	C	1.7589	1.6991	0.3849	C	0.3708	0.0179	−1.8279
H	1.9614	−1.2595	−2.4714	H	1.9192	1.9593	2.5103	H	2.3775	−0.2759	−2.5783
H	−0.1767	−2.5995	−2.3789	H	−1.493	3.7148	0.9395	H	3.2423	1.219	−0.6994
H	−0.216	−2.3199	0.6736	H	−1.1113	2.631	−1.2813	H	0.3763	2.1486	−0.1201
H	1.9486	−0.9603	0.5809	H	2.4429	0.8474	0.373	H	−0.4564	0.7176	−1.9861
H	−0.1164	−0.2122	−1.5286	H	1.2763	1.8772	−1.7	H	1.6444	−0.6173	−0.2237
H	3.2571	−1.7199	−1.3526	H	0.6016	0.8843	2.0282	H	1.264	0.4707	−3.7401
H	−0.2531	−3.9228	−1.2008	H	−1.4737	1.9552	1.0878	H	2.6557	2.8916	−0.6764
H	−1.5452	−1.9548	−0.4307	H	0.1938	3.7049	−0.7907	H	1.5403	1.5195	1.0476
H	2.1063	0.3386	−0.604	H	2.3607	2.5989	0.2138	H	0.0398	−0.9756	−2.1417

C	2.1593	−3.9253	−2.168	C	−0.2796	3.1744	3.1677	C	3.0561	2.2676	−3.1663
H	1.7445	−4.9177	−1.9641	H	0.552	3.3079	3.8675	H	3.3266	3.2934	−2.8947
H	3.244	−3.9749	−2.0281	H	−0.8943	4.0803	3.193	H	2.7232	2.2691	−4.2094
H	0.0114	−0.8594	4.167	H	3.4631	−1.5325	−1.9904	H	0.5589	−1.4457	3.1395
H	1.96	−3.681	−3.23	H	−0.8997	2.3311	3.5288	H	3.9682	1.6424	−3.0974
**M0034**	**X**	**Y**	**Z**	**M0031**	**X**	**Y**	**Z**	**M0037**	**X**	**Y**	**Z**
C	1.5428	0.2134	1.0853	C	0.8413	−0.1906	0.3299	C	1.3871	0.2106	1.3287
C	2.9487	1.8458	2.8342	C	2.9459	0.5235	2.0033	C	2.5622	1.8016	3.2718
C	1.0692	0.3776	2.3901	C	1.2765	−1.0935	1.316	C	0.8367	0.2322	2.6157
C	2.7351	0.8175	0.656	C	1.4968	1.0333	0.1293	C	2.5441	0.9343	1.0112
C	3.4223	1.6426	1.5415	C	2.5384	1.3798	0.9895	C	3.116	1.7347	1.9974
C	1.7901	1.209	3.2525	C	2.3302	−0.7122	2.1481	C	1.4391	1.0468	3.578
H	4.342	2.1224	1.2179	H	3.0474	2.3295	0.8434	H	4.0117	2.3046	1.7652
H	1.4353	1.3447	4.2713	H	2.6743	−1.4036	2.9139	H	1.0221	1.0753	4.582
H	3.4943	2.4891	3.5181	H	3.7614	0.8069	2.6623	H	3.0199	2.4291	4.0309
C	−0.1706	−0.3166	2.9038	C	0.6683	−2.4697	1.5071	C	−0.3719	−0.5951	2.9979
H	−0.4812	−1.0919	2.1996	H	0.0693	−2.762	0.6411	H	−0.4945	−1.4316	2.3045
H	0.0881	−0.8348	3.8344	H	1.492	−3.1917	1.5622	H	−0.1842	−1.0344	3.9846
C	−1.3345	0.6441	3.1754	C	−0.185	−2.6043	2.7743	C	−1.678	0.2067	3.0557
H	−1.6441	1.1735	2.2681	H	−1.0819	−1.9801	2.7227	H	−1.9839	0.5423	2.0597
H	−2.2032	0.0993	3.5592	H	−0.5084	−3.6418	2.9058	H	−2.4883	−0.4082	3.4607
H	−1.0555	1.3989	3.9181	H	0.3786	−2.3085	3.6658	H	−1.5724	1.0926	3.6911
C	3.2774	0.5438	−0.7231	C	1.1709	1.9448	−1.026	C	3.1633	0.8279	−0.3567
H	2.539	0.7436	−1.507	H	2.0822	2.1632	−1.5919	H	3.3461	−0.2214	−0.6072
H	3.5687	−0.5086	−0.8137	H	0.7559	2.9003	−0.685	H	4.1159	1.3638	−0.3947
N	0.822	−0.5847	0.1276	N	−0.2973	−0.5365	−0.4843	N	0.7347	−0.573	0.314
C	1.2089	−1.9001	0.0571	C	−0.1576	−1.5022	−1.4592	C	1.1543	−1.878	0.2123
C	0.7237	−2.8008	−1.0211	C	1.1601	−1.7139	−2.103	C	0.2864	−2.8829	−0.4481
C	0.8204	−4.1598	−1.0401	C	1.5475	−2.7863	−2.8505	C	0.6445	−4.0656	−1.019
C	0.267	−4.5905	−2.2817	C	2.8818	−2.5195	−3.2747	C	−0.5639	−4.6799	−1.4635
C	−0.1153	−3.4582	−2.9275	C	3.1958	−1.3008	−2.7603	C	−1.5641	−3.8299	−1.113
O	0.1529	−2.3661	−2.1806	O	2.165	−0.7999	−2.0512	O	−1.0675	−2.7359	−0.4944
H	1.2479	−4.7607	−0.2523	H	0.9364	−3.6506	−3.0623	H	1.6546	−4.438	−1.0983
H	0.1712	−5.6029	−2.6453	H	3.5198	−3.1468	−3.8798	H	−0.6734	−5.6286	−1.968
H	−0.5707	−3.2673	−3.8867	H	4.0763	−0.6786	−2.8073	H	−2.6374	−3.8613	−1.2186
O	1.9738	−2.3904	0.8787	O	−1.087	−2.2147	−1.82	O	2.2206	−2.2609	0.6747
C	−0.2192	0.0369	−0.7187	C	−1.6663	−0.45	0.1044	C	−0.3482	0.0284	−0.4971
C	−1.3451	2.1346	−1.4821	C	−2.612	1.6135	−1.0687	C	−1.2882	0.4325	−2.7896
C	−2.6526	0.134	−1.3312	C	−1.7491	1.8973	1.1516	C	−1.6402	2.0839	−1.0891
N	−2.6493	1.5787	−1.1575	N	−2.7818	2.2952	0.2074	N	−1.428	1.8521	−2.5085
C	−1.6173	−0.521	−0.4239	C	−1.7511	0.3901	1.3848	C	−0.4859	1.5351	−0.2551
C	−0.2549	1.5607	−0.5795	C	−2.6879	0.1012	−0.9027	C	−0.1324	−0.1778	−2.0034
H	−1.0829	1.9475	−2.5453	H	−3.4143	1.9404	−1.7405	H	−2.2221	−0.1197	−2.5503
H	−2.4497	−0.1455	−2.387	H	−1.9431	2.4008	2.1062	H	−2.5933	1.6284	−0.7438
H	−1.88	−0.3135	0.6175	H	−0.9584	0.1371	2.0923	H	0.4363	2.0503	−0.5432
H	−0.4605	1.8479	0.4554	H	−2.5742	−0.3981	−1.8662	H	0.8044	0.307	−2.2987
H	0.0217	−0.1788	−1.764	H	−1.9601	−1.4788	0.3475	H	−1.2996	−0.4383	−0.2144
H	−1.3879	3.221	−1.3499	H	−1.6576	1.8965	−1.5522	H	−1.1094	0.3069	−3.8631
H	−3.6507	−0.244	−1.0852	H	−0.7478	2.2254	0.8201	H	−1.7241	3.1635	−0.9234
H	−1.6397	−1.6049	−0.5654	H	−2.704	0.1224	1.8564	H	−0.6655	1.7581	0.7992
H	0.7084	2.0011	−0.8537	H	−3.6842	−0.1515	−0.5208	H	−0.0508	−1.2368	−2.2596
C	−3.7006	2.2089	−1.9295	C	−2.8034	3.7328	0.0384	C	−2.4789	2.4497	−3.3059
H	−4.673	1.8086	−1.6255	H	−3.6246	4.015	−0.6287	H	−2.5308	3.525	−3.106
H	−3.7035	3.2872	−1.7403	H	−2.9669	4.2195	1.006	H	−2.2593	2.312	−4.3697
H	4.1598	1.1573	−0.9242	H	0.4532	1.4864	−1.7055	H	2.5126	1.2481	−1.1315

H	−3.5891	2.0515	−3.02	H	−1.8613	4.1315	−0.3891	H	−3.4765	2.0137	−3.1016
**M0048**	**X**	**Y**	**Z**	**M0056**	**X**	**Y**	**Z**	**M0045**	**X**	**Y**	**Z**
C	1.2856	0.6184	1.304	C	−1.0143	0.558	1.5548	C	−1.143	0.3961	1.1311
C	2.3412	2.3303	3.2122	C	−1.7926	2.3226	3.5504	C	−2.4849	2.0672	2.8949
C	0.7971	0.5605	2.6139	C	−1.9255	1.5772	1.2486	C	−2.2854	1.0791	0.6945
C	2.3266	1.4872	0.944	C	−0.5259	0.3783	2.8577	C	−0.6758	0.5202	2.4455
C	2.8395	2.3445	1.9134	C	−0.926	1.2776	3.8452	C	−1.3626	1.3647	3.3163
C	1.3372	1.4383	3.5576	C	−2.294	2.4615	2.2632	C	−2.9429	1.9199	1.5931
H	3.6471	3.0225	1.6493	H	−0.5508	1.1531	4.8576	H	−1.0115	1.468	4.3397
H	0.9621	1.4093	4.5781	H	−2.9973	3.2591	2.0365	H	−3.8273	2.4588	1.262
H	2.7478	3.008	3.9573	H	−2.0877	3.0207	4.3281	H	−3.0058	2.7242	3.5852
C	−0.2198	−0.4683	3.0512	C	−2.598	1.6789	−0.1014	C	−2.8468	0.8844	−0.6949
H	−0.909	−0.0113	3.7717	H	−1.9293	1.357	−0.9044	H	−2.0466	0.6333	−1.3982
H	−0.8199	−0.7979	2.1985	H	−2.8452	2.7279	−0.3021	H	−3.2867	1.8276	−1.0398
C	0.4557	−1.6897	3.6912	C	−3.8744	0.8271	−0.146	C	−3.9133	−0.2191	−0.7281
H	1.0208	−1.3983	4.5833	H	−3.6425	−0.2174	0.0808	H	−3.5084	−1.1657	−0.3597
H	−0.2912	−2.4341	3.9872	H	−4.3449	0.8838	−1.1335	H	−4.2861	−0.3677	−1.7469
H	1.1551	−2.1551	2.9906	H	−4.5966	1.1777	0.5988	H	−4.7615	0.0477	−0.0888
C	2.9006	1.4642	−0.4488	C	0.3528	−0.7951	3.2066	C	0.5438	−0.225	2.9191
H	3.1524	0.4391	−0.7386	H	1.156	−0.9443	2.4796	H	0.5668	−1.2424	2.5219
H	3.8104	2.0687	−0.5011	H	0.8011	−0.6655	4.1955	H	1.465	0.2836	2.6093
N	0.6843	−0.2143	0.2957	N	−0.5225	−0.3134	0.5194	N	−0.4424	−0.4364	0.1871
C	1.2066	−1.4779	0.1648	C	−1.0434	−1.583	0.4931	C	−0.831	−1.7511	0.1532
C	0.4344	−2.5181	−0.5487	C	−0.3223	−2.6649	−0.2151	C	−0.4021	−2.648	−0.9494
C	0.8815	−3.6107	−1.2249	C	−0.8215	−3.7645	−0.8417	C	−0.4106	−4.0102	−0.9452
C	−0.2797	−4.3093	−1.6698	C	0.3054	−4.5135	−1.2941	C	0.0378	−4.4225	−2.2338
C	−1.3421	−3.5995	−1.2077	C	1.4011	−3.8198	−0.889	C	0.2739	−3.2786	−2.9277
O	−0.9261	−2.5106	−0.5235	O	1.0373	−2.6979	−0.2287	O	0.0112	−2.1942	−2.1665
H	1.9187	−3.8692	−1.378	H	−1.8695	−3.9972	−0.956	H	−0.7127	−4.625	−0.1112
H	−0.3187	−5.2206	−2.2486	H	0.3005	−5.445	−1.8407	H	0.1609	−5.4321	−2.5973
H	−2.4108	−3.734	−1.2695	H	2.4631	−3.9866	−0.9781	H	0.6104	−3.0742	−3.9323
O	2.2826	−1.7917	0.6586	O	−2.0822	−1.8717	1.0751	O	−1.5496	−2.2394	1.0184
C	−0.2476	0.4859	−0.6108	C	0.4409	0.308	−0.4113	C	0.6396	0.1359	−0.6483
C	−2.5288	1.4008	−1.0586	C	2.7554	1.1226	−0.8792	C	1.909	2.1715	−1.3667
C	−1.0474	1.0102	−2.9081	C	1.2711	0.7075	−2.7213	C	3.0739	0.0854	−1.283
N	−2.4272	0.9706	−2.4461	N	2.6459	0.6375	−2.2479	N	3.1992	1.5313	−1.1488
C	−0.1691	0.0731	−2.0818	C	0.3549	−0.164	−1.864	C	1.9966	−0.5327	−0.3801
C	−1.7012	0.5102	−0.1343	C	1.8919	0.3003	0.0752	C	0.7785	1.6497	−0.4634
H	−2.1984	2.4555	−0.9439	H	2.4617	2.192	−0.8119	H	1.6295	2.0094	−2.4172
H	−0.6372	2.0423	−2.8646	H	0.899	1.7543	−2.7227	H	2.8264	−0.1344	−2.3312
H	−0.5304	−0.9449	−2.2343	H	0.6792	−1.1995	−1.9739	H	2.2612	−0.4009	0.6739
H	−2.1077	−0.5036	−0.1288	H	2.2647	−0.725	0.1249	H	0.9915	1.8883	0.5847
H	0.0971	1.5281	−0.5769	H	0.138	1.3629	−0.4251	H	0.3808	−0.0314	−1.6989
H	−3.5826	1.364	−0.7608	H	3.8054	1.0613	−0.5729	H	2.0246	3.2518	−1.2299
H	−1.0253	0.702	−3.9592	H	1.2436	0.3568	−3.7587	H	4.0476	−0.3735	−1.0814
H	0.8689	0.1243	−2.4301	H	−0.6778	−0.0912	−2.2242	H	1.9443	−1.6102	−0.5614
H	−1.7485	0.9072	0.8861	H	1.945	0.7393	1.0778	H	−0.1547	2.1609	−0.7198
C	−3.2918	1.7561	−3.3041	C	3.5432	1.3546	−3.1318	C	3.8454	1.9435	0.0842
H	−3.2446	1.3713	−4.3281	H	3.483	0.9338	−4.1407	H	3.2991	1.682	1.0083
H	−4.3281	1.6784	−2.9585	H	4.5742	1.2476	−2.7793	H	3.9794	3.0302	0.0742
H	2.1985	1.8589	−1.1938	H	−0.2427	−1.7155	3.2218	H	0.5583	−0.2834	4.0111
H	−3.0185	2.8294	−3.3265	H	3.3127	2.436	−3.1951	H	4.8372	1.4834	0.1425
**M0059**	**X**	**Y**	**Z**	**M0049**	**X**	**Y**	**Z**	**M0053**	**X**	**Y**	**Z**
C	0.4818	−0.9421	−0.1398	C	1.5191	0.2511	0.891	C	−0.7011	0.3543	1.4765
C	1.3892	−3.0836	1.3813	C	2.9705	1.8949	2.5906	C	−1.7323	2.1871	3.287

C	−0.3267	−1.4395	0.8918	C	1.0958	0.4034	2.2138	C	−1.862	1.058	1.1446
C	1.704	−1.5552	−0.4658	C	2.6885	0.8684	0.4197	C	−0.0834	0.5089	2.7272
C	2.151	−2.6181	0.3159	C	3.3976	1.7007	1.2809	C	−0.6085	1.438	3.6212
C	0.1561	−2.5089	1.6498	C	1.838	1.2413	3.0513	C	−2.3566	1.9865	2.0652
H	3.1001	−3.0907	0.0781	H	4.3002	2.1907	0.9254	H	−0.1335	1.5728	4.5894
H	−0.4593	−2.9085	2.4514	H	1.5236	1.3668	4.0847	H	−3.2526	2.5494	1.8139
H	1.7469	−3.9127	1.9845	H	3.5344	2.5422	3.2557	H	−2.1279	2.9163	3.988
C	−1.7354	−0.9439	1.136	C	−0.1087	−0.3186	2.77	C	−2.6431	0.7864	−0.1185
H	−1.967	−0.1453	0.4286	H	−0.4536	−1.0698	2.0558	H	−2.0199	0.2596	−0.8435
H	−2.4147	−1.7683	0.8854	H	0.2023	−0.8686	3.6661	H	−2.9404	1.7373	−0.5779
C	−2.0257	−0.4792	2.5675	C	−1.2606	0.6235	3.1348	C	−3.8869	−0.0643	0.1721
H	−1.4148	0.3829	2.8534	H	−1.6003	1.1886	2.2601	H	−3.5888	−1.0107	0.6315
H	−3.0766	−0.1874	2.6608	H	−2.1145	0.0619	3.5275	H	−4.4362	−0.2755	−0.7517
H	−1.8385	−1.2766	3.2941	H	−0.9562	1.3516	3.8939	H	−4.5638	0.4524	0.861
C	2.5004	−1.1052	−1.6654	C	3.1882	0.5952	−0.9755	C	1.0995	−0.3448	3.1054
H	1.9219	−1.2257	−2.5883	H	2.4266	0.7938	−1.7372	H	1.9158	−0.2689	2.3787
H	3.4143	−1.6968	−1.7647	H	3.4781	−0.457	−1.0751	H	1.4905	−0.057	4.0852
N	0.0708	0.2048	−0.9082	N	0.7757	−0.562	−0.0359	N	−0.0844	−0.5438	0.5343
C	−0.5155	0.0613	−2.145	C	1.1639	−1.8775	−0.0978	C	−0.4925	−1.8501	0.6077
C	−1.1332	−1.2234	−2.5551	C	0.6523	−2.7966	−1.1479	C	0.1913	−2.9277	−0.1531
C	−1.6103	−1.5285	−3.7964	C	0.7729	−4.1539	−1.1558	C	−0.2827	−4.1865	−0.3728
C	−2.1347	−2.85	−3.7139	C	0.1819	−4.6082	−2.3702	C	0.7217	−4.8766	−1.1127
C	−1.9394	−3.2447	−2.4275	C	−0.2455	−3.4914	−3.0146	C	1.7358	−3.9881	−1.2783
O	−1.3355	−2.2735	−1.7147	O	0.0302	−2.3855	−2.2902	O	1.4334	−2.8041	−0.7036
H	−1.5849	−0.869	−4.6502	H	1.241	−4.7369	−0.3776	H	−1.2376	−4.5565	−0.0323
H	−2.5952	−3.4269	−4.5023	H	0.0901	−5.6263	−2.7187	H	0.6951	−5.8962	−1.4681
H	−2.1626	−4.1503	−1.8851	H	−0.7409	−3.3202	−3.9579	H	2.702	−4.0425	−1.7556
O	−0.5558	0.9804	−2.9558	O	1.9529	−2.3532	0.7096	O	−1.4404	−2.1919	1.3073
C	0.7099	1.5382	−0.6915	C	−0.2811	0.0455	−0.874	C	0.9579	−0.0328	−0.387
C	1.0394	1.6537	1.8554	C	−1.4	2.1062	−1.7362	C	1.6883	0.3668	−2.7553
C	−0.9376	2.6488	0.9203	C	−2.7045	0.1088	−1.5168	C	2.5013	1.8236	−1.0406
N	0.047	2.7135	1.9975	N	−2.7331	1.566	−1.5098	N	2.1579	1.7128	−2.4516
C	−0.3415	2.6452	−0.4904	C	−1.6788	−0.4999	−0.5488	C	1.3796	1.4059	−0.0721
C	1.7077	1.5874	0.4749	C	−0.3194	1.5734	−0.7775	C	0.5308	−0.1003	−1.8608
H	1.8051	1.789	2.6272	H	−1.109	1.8586	−2.7671	H	2.5344	−0.3236	−2.6284
H	−1.6281	3.4922	1.0315	H	−2.4604	−0.2189	−2.5374	H	3.3803	1.1889	−0.8586
H	−1.1273	2.5481	−1.2424	H	−1.9353	−0.2453	0.4859	H	0.5225	2.0817	−0.1679
H	2.4107	0.7509	0.4514	H	−0.5323	1.8869	0.2503	H	−0.3404	0.5467	−2.0134
H	1.2445	1.7612	−1.621	H	−0.0565	−0.1974	−1.9177	H	1.8468	−0.6591	−0.2609
H	0.5523	0.6964	2.0639	H	−1.4486	3.1983	−1.6723	H	1.3965	0.3256	−3.8101
H	−1.5307	1.7413	1.0651	H	−3.7098	−0.2633	−1.293	H	2.8046	2.8553	−0.8333
H	0.1559	3.6044	−0.6838	H	−1.7006	−1.591	−0.6278	H	1.7411	1.4996	0.9564
H	2.3032	2.4955	0.3187	H	0.6463	2.0123	−1.0461	H	0.2329	−1.1159	−2.1377
C	0.6346	4.0315	2.1585	C	−3.3976	2.1284	−0.3488	C	1.2585	2.7562	−2.9103
H	1.2809	4.0312	3.0426	H	−2.9133	1.9113	0.6198	H	0.2638	2.7624	−2.4296
H	1.2368	4.383	1.3014	H	−3.4514	3.2169	−0.4557	H	1.1061	2.6512	−3.9896
H	2.7884	−0.0508	−1.6055	H	4.0639	1.2092	−1.2036	H	0.8062	−1.3996	3.1532
H	−0.1621	4.7639	2.3263	H	−4.4221	1.7452	−0.2999	H	1.7203	3.7328	−2.7307
**M0051**	**X**	**Y**	**Z**	**M0057**	**X**	**Y**	**Z**	**M0055**	**X**	**Y**	**Z**
C	−1.1495	0.37	1.626	C	1.4894	0.4121	1.3306	C	0.5456	−1.4077	1.3013
C	−2.0746	2.1387	3.5546	C	2.8932	2.2331	2.8854	C	2.3289	−2.4755	3.1363
C	−2.1143	1.3221	1.2738	C	0.9125	0.8865	2.5154	C	0.548	−0.9152	2.6118
C	−0.678	0.2573	2.9433	C	2.7837	0.7914	0.9427	C	1.3978	−2.4506	0.9048
C	−1.1481	1.1599	3.895	C	3.4687	1.7136	1.7313	C	2.2938	−2.9672	1.8354
C	−2.56	2.2076	2.2569	C	1.6317	1.8093	3.2778	C	1.4569	−1.4683	3.5192

H	−0.7864	1.0876	4.9173	H	4.4696	2.0204	1.4394	H	2.9577	−3.7764	1.5426
H	−3.3134	2.9479	1.9981	H	1.1976	2.1818	4.2029	H	1.4705	−1.1085	4.5446
H	−2.4317	2.8352	4.3075	H	3.4392	2.9516	3.4896	H	3.0275	−2.8924	3.8559
C	−2.7163	1.3958	−0.1122	C	−0.4292	0.3986	3.016	C	−0.4216	0.1509	3.0769
H	−3.8073	1.361	−0.0087	H	−0.7223	−0.5004	2.467	H	−1.026	0.4931	2.2333
H	−2.4404	0.5057	−0.6838	H	−0.3066	0.089	4.0604	H	−1.1224	−0.3057	3.7873
C	−2.3374	2.6604	−0.8928	C	−1.5466	1.4465	2.9434	C	0.2511	1.3574	3.739
H	−2.6401	3.563	−0.3517	H	−1.7554	1.7489	1.9117	H	0.9678	1.8323	3.0605
H	−2.8304	2.6716	−1.8703	H	−2.4743	1.0502	3.3694	H	−0.4973	2.1051	4.0203
H	−1.2571	2.7266	−1.0609	H	−1.2768	2.3491	3.5019	H	0.7957	1.0755	4.6458
C	0.2599	−0.8529	3.3402	C	3.4502	0.1662	−0.2553	C	1.3176	−3.024	−0.4861
H	1.0771	−0.9803	2.6249	H	2.8	0.1521	−1.1341	H	0.2837	−3.2869	−0.7302
H	0.6891	−0.6674	4.3287	H	3.714	−0.8744	−0.0329	H	1.9293	−3.9267	−0.5666
N	−0.5969	−0.5087	0.6299	N	0.7498	−0.4677	0.4646	N	−0.3419	−0.8264	0.3283
C	−1.089	−1.7918	0.6197	C	1.0668	−1.802	0.5569	C	−1.5988	−1.3809	0.258
C	−0.3465	−2.8618	−0.0848	C	0.6321	−2.747	−0.4969	C	−2.6823	−0.6524	−0.439
C	−0.8241	−3.9895	−0.6775	C	0.3525	−4.0742	−0.3873	C	−3.7806	−1.1456	−1.0729
C	0.3149	−4.707	−1.1502	C	0.0296	−4.5262	−1.7017	C	−4.5246	−0.015	−1.5231
C	1.3965	−3.9679	−0.79	C	0.1575	−3.4417	−2.51	C	−3.8285	1.077	−1.1118
O	1.0125	−2.8473	−0.1392	O	0.5261	−2.3557	−1.795	O	−2.7107	0.7075	−0.4482
H	−1.8662	−4.2603	−0.7563	H	0.3796	−4.6447	0.5288	H	−4.0146	−2.1927	−1.1936
H	0.3273	−5.6481	−1.68	H	−0.2503	−5.5245	−2.0046	H	−5.4541	−0.0148	−2.0733
H	2.4606	−4.0985	−0.9085	H	0.036	−3.284	−3.5702	H	−3.9912	2.1399	−1.1991
O	−2.1174	−2.0948	1.2091	O	1.7293	−2.2443	1.486	O	−1.8669	−2.4511	0.7864
C	0.3216	0.1337	−0.3302	C	−0.3395	0.1736	−0.299	C	0.2984	0.1079	−0.6184
C	2.5984	1.0183	−0.8553	C	−2.7053	0.2277	−1.0734	C	1.1276	2.4083	−1.1321
C	1.0839	0.5532	−2.6593	C	−1.0737	1.4797	−2.3056	C	0.7314	0.881	−2.943
N	2.4711	0.5265	−2.2198	N	−2.272	0.6573	−2.3945	N	0.6638	2.2675	−2.5052
C	0.2133	−0.3428	−1.7799	C	0.0838	0.7146	−1.6675	C	−0.1556	−0.0096	−2.0751
C	1.7831	0.1735	0.1214	C	−1.6364	−0.632	−0.4018	C	0.2883	1.5705	−0.1701
H	2.2729	2.0784	−0.7837	H	−2.9475	1.0995	−0.4277	H	2.1949	2.1127	−1.0416
H	0.6802	1.5886	−2.6527	H	−1.2716	2.4041	−1.7212	H	1.7763	0.5033	−2.9213
H	0.5607	−1.3701	−1.8974	H	0.3882	−0.1084	−2.3172	H	−1.1879	0.319	−2.2039
H	2.188	−0.8395	0.1685	H	−1.5067	−1.5342	−1.0008	H	−0.7369	1.9456	−0.1434
H	−0.0143	1.1766	−0.3403	H	−0.5868	1.053	0.3058	H	1.3519	−0.2027	−0.6104
H	3.6568	0.991	−0.5739	H	−3.6268	−0.3545	−1.1819	H	1.0658	3.4657	−0.8521
H	1.0416	0.201	−3.6957	H	−0.7935	1.7964	−3.3162	H	0.3948	0.8297	−3.9842
H	−0.8284	−0.2936	−2.1167	H	0.9358	1.3922	−1.5426	H	−0.084	−1.0509	−2.4087
H	1.8451	0.6191	1.1207	H	−1.9734	−0.93	0.5974	H	0.7092	1.6488	0.8377
C	3.3232	1.27	−3.1263	C	−3.3333	1.3514	−3.0962	C	1.4019	3.137	−3.3993
H	3.2536	0.8438	−4.1323	H	−2.9989	1.6171	−4.1043	H	0.9975	3.0544	−4.4134
H	4.3653	1.1992	−2.7981	H	−4.2064	0.6974	−3.1885	H	1.2969	4.1774	−3.0748
H	−0.2842	−1.8038	3.38	H	4.3687	0.7005	−0.5137	H	1.6726	−2.3175	−1.2463
H	3.0549	2.3428	−3.1869	H	−3.6521	2.2805	−2.5845	H	2.4825	2.8971	−3.4385
**M0058**	**X**	**Y**	**Z**	**M0050**	**X**	**Y**	**Z**	**M0032**	**X**	**Y**	**Z**
C	0.9946	−1.6161	−0.4141	C	−0.0492	−0.9453	0.2654	C	0.6583	−1.2321	0.6275
C	1.9831	−3.5155	−2.1664	C	0.6732	−3.0334	1.9538	C	1.2461	−3.3821	2.2831
C	2.0309	−1.2578	−1.2904	C	−0.9568	−1.3875	1.2429	C	−0.3421	−1.7364	1.4696
C	0.4907	−2.9209	−0.3577	C	1.186	−1.5857	0.079	C	1.9447	−1.7989	0.595
C	0.9948	−3.8621	−1.2563	C	1.5355	−2.6194	0.9464	C	2.2255	−2.8667	1.4397
C	2.5057	−2.2267	−2.1729	C	−0.5711	−2.433	2.0839	C	−0.0231	−2.8269	2.286
H	0.6093	−4.878	−1.2323	H	2.4919	−3.1185	0.8128	H	3.2154	−3.3147	1.4197
H	3.3053	−1.9833	−2.8648	H	−1.2664	−2.7837	2.8429	H	−0.7896	−3.2451	2.9332
H	2.3638	−4.2564	−2.8634	H	0.9601	−3.8433	2.618	H	1.4729	−4.2257	2.9284
C	2.627	0.1343	−1.2235	C	−2.3455	−0.8006	1.4091	C	−1.7502	−1.1775	1.5107

H	2.7403	0.3949	−0.1651	H	−2.6158	−0.177	0.5537	H	−1.8464	−0.3305	0.8275
H	1.9183	0.8648	−1.6384	H	−3.0588	−1.6335	1.4137	H	−2.4421	−1.944	1.139
C	3.9716	0.3102	−1.926	C	−2.5306	0.0134	2.6956	C	−2.1887	−0.7332	2.9105
H	4.7111	−0.4036	−1.5487	H	−1.911	0.9149	2.6925	H	−1.5112	0.0288	3.3104
H	4.3557	1.3195	−1.7497	H	−3.5744	0.3256	2.8004	H	−3.1976	−0.3098	2.8775
H	3.8891	0.1754	−3.0097	H	−2.2626	−0.5751	3.5794	H	−2.2	−1.5675	3.6188
C	−0.5111	−3.3249	0.6924	C	2.1085	−1.2428	−1.0634	C	2.9809	−1.2975	−0.3761
H	−1.3129	−2.5906	0.8077	H	1.5815	−0.7096	−1.8554	H	2.5638	−1.2397	−1.3865
H	−0.9586	−4.2938	0.454	H	2.5217	−2.161	−1.492	H	3.8463	−1.9655	−0.3972
N	0.4166	−0.6212	0.4506	N	−0.4032	0.18	−0.5619	N	0.3939	−0.1079	−0.2283
C	0.8803	−0.6123	1.7443	C	−1.3538	0.0199	−1.5488	C	−0.4909	−0.2001	−1.2671
C	0.1367	0.1169	2.7965	C	−1.5638	−1.3103	−2.1671	C	−0.8327	−1.5447	−1.7916
C	0.6143	0.7044	3.9271	C	−2.6295	−1.7051	−2.9201	C	−1.94	−1.9056	−2.499
C	−0.5201	1.2252	4.618	C	−2.3654	−3.048	−3.3164	C	−1.7837	−3.2876	−2.8101
C	−1.5998	0.8965	3.8616	C	−1.1558	−3.3603	−2.7797	C	−0.5843	−3.652	−2.2833
O	−1.2186	0.2198	2.7555	O	−0.6581	−2.3204	−2.0816	O	0.0021	−2.6098	−1.6609
H	1.6535	0.75	4.2159	H	−3.4859	−1.0917	−3.1554	H	−2.755	−1.2475	−2.76
H	−0.5309	1.7654	5.5533	H	−2.9885	−3.6927	−3.9184	H	−2.4646	−3.9214	−3.3589
H	−2.6613	1.0542	3.9706	H	−0.539	−4.2452	−2.8027	H	−0.0298	−4.5774	−2.2673
O	1.8929	−1.2179	2.0707	O	−2.0576	0.9436	−1.9407	O	−1.0281	0.7661	−1.795
C	−0.465	0.3553	−0.2186	C	−0.3349	1.5607	0.0042	C	0.952	1.1861	0.2438
C	−2.7222	0.9514	−1.1143	C	1.7812	2.4469	−1.1143	C	−0.8182	3.0569	0.3051
C	−1.1512	2.7069	−0.6551	C	1.9793	1.6559	1.1376	C	0.7858	3.127	−1.4904
N	−2.5518	2.3123	−0.6253	N	2.4806	2.611	0.157	N	0.0695	3.9062	−0.484
C	−0.311	1.8047	0.2464	C	0.454	1.667	1.319	C	1.6417	2.0381	−0.8397
C	−1.9402	−0.0499	−0.2665	C	0.2566	2.5542	−1.0124	C	0.0014	2.0442	1.0968
H	−2.3986	0.8673	−2.1741	H	2.1632	3.1934	−1.8199	H	−1.358	3.6986	1.0122
H	−0.7485	2.6818	−1.6906	H	2.4616	1.8586	2.1002	H	1.4553	3.8105	−2.0278
H	−0.6576	1.9398	1.2718	H	0.1714	0.884	2.0266	H	2.0633	1.385	−1.6111
H	−2.3519	−0.0846	0.7441	H	−0.2074	2.4123	−1.99	H	−0.6508	1.3993	1.6932
H	−0.1203	0.3404	−1.2601	H	−1.3727	1.8574	0.1986	H	1.7406	0.8516	0.9218
H	−3.7891	0.7041	−1.0872	H	2.0488	1.4644	−1.5179	H	−1.5623	2.5457	−0.3255
H	−1.075	3.7431	−0.3081	H	2.2986	0.6544	0.8347	H	0.0968	2.6853	−2.2234
H	0.7427	2.1017	0.1991	H	0.1512	2.6191	1.7725	H	2.4831	2.5358	−0.3423
H	−2.0312	−1.0465	−0.7129	H	−0.022	3.5629	−0.6819	H	0.6275	2.6075	1.7993
C	−3.3763	3.2435	−1.3692	C	2.4866	3.9801	0.638	C	−0.6619	4.9921	−1.1046
H	−3.2648	4.2508	−0.9545	H	2.9243	4.6302	−0.1271	H	−1.1629	5.5899	−0.3352
H	−4.429	2.9553	−1.284	H	1.4948	4.3925	0.8974	H	0.0337	5.6452	−1.6427
H	−0.0137	−3.4119	1.6657	H	2.9523	−0.6226	−0.7404	H	3.3425	−0.2955	−0.1179
H	−3.1174	3.284	−2.4454	H	3.1167	4.0432	1.5316	H	−1.4289	4.6432	−1.8226

**M0052**	**X**	**Y**	**Z**
C	1.1394	0.2182	1.3019
C	2.3293	1.8647	3.1941
C	0.585	0.2959	2.5857
C	2.302	0.9248	0.9623
C	2.8804	1.7524	1.9217
C	1.1972	1.1335	3.5221
H	3.7808	2.3067	1.6703
H	0.7807	1.2008	4.5244
H	2.7944	2.5105	3.933
C	−0.634	−0.5029	2.9952
H	−0.8884	−1.229	2.2191
H	−0.3784	−1.0827	3.8904
C	−1.8624	0.3628	3.2977
H	−2.2003	0.9002	2.4056

H	−2.6933	−0.258	3.6487
H	−1.6466	1.1069	4.0716
C	2.9214	0.7874	−0.4034
H	3.0002	−0.2659	−0.6872
H	3.9241	1.2236	−0.4176
N	0.4902	−0.6077	0.3189
C	0.9072	−1.9174	0.281
C	0.0705	−2.9479	−0.3824
C	0.4544	−4.1711	−0.8412
C	−0.725	−4.8062	−1.3354
C	−1.736	−3.9216	−1.1324
O	−1.2739	−2.7936	−0.5494
H	1.4616	−4.5583	−0.814
H	−0.8074	−5.7875	−1.7788
H	−2.7968	−3.9465	−1.328
O	1.9496	−2.2867	0.8052
C	−0.5502	−0.0334	−0.5662
C	−1.3362	0.3517	−2.9272
C	−1.8509	1.9901	−1.2664
N	−1.5846	1.7658	−2.6805
C	−0.7561	1.4652	−0.3226
C	−0.2272	−0.2519	−2.0527
H	−2.2721	−0.1925	−2.736
H	−2.8009	1.4943	−1.0212
H	0.1779	2.012	−0.4926
H	0.7374	0.2158	−2.2802
H	−1.504	−0.5253	−0.3427
H	−1.0988	0.215	−3.9876
H	−1.9989	3.0627	−1.1023
H	−1.0444	1.6584	0.7144
H	−0.125	−1.3157	−2.2862
C	−0.5546	2.6354	−3.2185
H	0.4504	2.5001	−2.7789
H	−0.4699	2.4681	−4.2974
H	2.3284	1.2993	−1.1714
H	−0.8463	3.6794	−3.0629

Table S4. Energy and Boltzmann distribution used for a*R*-35′ UV and ECD calculations.

	M0001	M0002	M0003	M0005	M0006
SCF Energy (au)	−1037.564335	−1037.564211	−1037.563493	−1037.563847	−1037.563429
rel G (kJ/mol)	0.00	0.95	3.86	−0.35	−0.06
Boltzmann Distribution	19.7%	13.5%	4.1%	22.7%	20.3%
	M0010	M0011	M0012	M0014	M0026
SCF Energy (au)	−1037.562704	−1037.562771	−1037.562928	−1037.562636	−1037.562634
rel G (kJ/mol)	5.35	5.87	6.13	5.65	5.39
Boltzmann Distribution	2.3%	1.8%	1.7%	2.0%	2.2%
	M0008	M0004	M0007	M0017	
SCF Energy (au)	−1037.563082	−1037.562591	−1037.562615	−1037.561684	
rel G (kJ/mol)	4.02	5.00	4.84	9.69	
Boltzmann Distribution	3.9%	2.6%	2.8%	0.4%	

Table S5. Wavelength, oscillator strength, and rotatory strength used for a*R*-35′ UV and ECD calculations.

M0001			M0002			M0003		
Wavelength (nm)	Oscillator strength	Rotatory strength	Wavelength (nm)	Oscillator strength	Rotatory strength	Wavelength (nm)	Oscillator strength	Rotatory strength
167.8	1.89.E−02	76.74	168.3	8.61.E−02	113.56	168.6	5.28.E−02	−75.79
169.5	7.29.E−02	31.13	169.0	6.65.E−03	−41.75	171.3	3.92.E−02	11.31
178.3	1.58.E−02	15.21	179.0	7.00.E−03	0.66	178.1	1.83.E−01	58.85
180.2	3.08.E−02	25.60	180.5	2.84.E−03	−1.04	178.6	1.94.E−01	−32.87
181.6	2.32.E−01	12.83	182.3	1.65.E−01	−24.88	179.4	9.84.E−02	−20.62
183.5	7.97.E−02	−19.90	182.9	4.74.E−02	−5.60	180.4	1.12.E−01	37.90
184.2	3.02.E−01	41.86	183.7	4.02.E−01	−58.42	180.8	2.71.E−02	3.37
185.1	3.65.E−01	36.84	184.8	3.94.E−01	−13.95	182.7	2.21.E−01	27.54
188.1	4.00.E−02	12.05	187.7	3.94.E−02	−14.54	188.4	5.74.E−02	15.89
190.8	3.66.E−02	−11.28	190.6	2.94.E−02	18.18	191.6	1.06.E−01	75.01
192.0	3.91.E−02	−47.32	192.7	3.40.E−02	41.54	192.4	6.62.E−02	−74.13
198.2	5.86.E−03	−0.93	196.6	4.81.E−03	7.20	192.8	7.02.E−02	−94.70
198.4	6.82.E−03	2.24	197.7	1.48.E−02	18.18	198.1	3.20.E−02	15.89
199.7	1.99.E−02	−22.05	199.2	2.45.E−02	5.46	201.9	5.85.E−02	69.32
203.1	9.56.E−03	17.14	202.3	5.05.E−03	−9.90	205.1	7.19.E−03	5.61
209.8	4.28.E−02	−7.09	208.8	3.31.E−02	−8.83	210.0	1.49.E−02	−11.71
212.3	4.13.E−02	17.97	213.1	5.11.E−02	−16.45	219.2	1.37.E−01	3.36
230.7	3.12.E−01	44.17	229.2	3.34.E−01	−26.58	232.8	1.10.E−01	71.39
233.4	1.52.E−02	−38.26	233.4	3.24.E−03	−1.99	237.4	1.56.E−01	−55.81
249.2	2.93.E−02	−0.89	248.7	3.78.E−02	−0.89	252.8	2.24.E−02	−0.32
M0005			M0006			M0010		
Wavelength (nm)	Oscillator strength	Rotatory strength	Wavelength (nm)	Oscillator strength	Rotatory strength	Wavelength (nm)	Oscillator strength	Rotatory strength
168.4	4.39.E−02	−20.90	169.0	1.08.E−01	−107.11	170.2	3.95.E−02	53.04
169.5	5.09.E−02	−41.93	169.6	2.01.E−02	57.82	170.4	8.41.E−02	−5.87
178.4	2.17.E−02	10.60	178.7	2.66.E−03	−6.81	181.1	1.13.E−01	59.82

180.0	2.96.E−02	−3.78	179.2	6.21.E−02	53.91	182.7	3.91.E−01	60.05
181.6	1.91.E−01	−11.76	181.2	3.28.E−01	−16.24	184.3	8.79.E−02	−54.88
182.7	1.20.E−01	−14.31	182.5	2.62.E−01	−2.11	185.7	2.47.E−01	−65.46
183.0	2.21.E−01	4.09	183.0	3.31.E−02	4.35	187.2	7.23.E−02	61.01
184.0	3.93.E−01	−34.00	185.1	2.61.E−01	40.28	187.5	3.26.E−03	−1.54
187.9	5.74.E−02	−16.38	187.8	4.78.E−02	18.49	189.7	5.07.E−02	−7.43
191.0	4.10.E−02	5.45	190.8	5.29.E−02	1.26	191.4	2.49.E−02	−13.15
192.4	4.24.E−02	65.13	192.3	6.68.E−02	−90.26	193.7	3.87.E−02	−5.48
196.6	1.98.E−02	35.10	196.5	1.39.E−02	−14.07	194.3	2.87.E−02	−22.84
198.5	3.18.E−03	0.71	197.9	1.70.E−03	0.34	196.4	2.34.E−03	−0.56
199.4	2.73.E−02	−10.28	198.2	3.75.E−02	−19.36	198.8	2.30.E−02	−21.09
202.1	1.01.E−02	−18.48	202.7	2.26.E−02	33.54	206.2	4.32.E−03	−2.18
208.8	3.06.E−02	−12.82	209.3	4.42.E−02	4.20	209.2	3.49.E−02	2.39
214.6	7.21.E−02	−18.58	214.1	5.79.E−02	5.50	214.7	2.51.E−02	26.32
230.5	3.10.E−01	−41.36	230.0	2.82.E−01	56.90	230.5	3.12.E−01	106.76
233.7	4.80.E−03	14.11	233.9	1.51.E−02	−34.90	234.3	5.19.E−02	−57.18
249.2	3.15.E−02	−2.62	249.9	5.11.E−02	2.12	249.3	1.64.E−02	6.94
M0011			M0012			M0014		
Wavelength (nm)	Oscillator strength	Rotatory strength	Wavelength (nm)	Oscillator strength	Rotatory strength	Wavelength (nm)	Oscillator strength	Rotatory strength
170.7	1.87.E−02	46.54	169.1	1.20.E−02	−14.30	170.2	4.80.E−02	−49.51
171.2	8.90.E−02	8.18	170.5	7.26.E−02	−1.01	171.3	8.22.E−02	−9.06
180.5	7.85.E−03	−4.12	182.6	4.21.E−02	30.28	180.3	1.30.E−02	−7.65
181.9	1.70.E−01	54.34	183.7	3.57.E−01	−23.69	182.0	2.08.E−01	−52.62
183.2	3.66.E−01	−110.91	184.6	1.09.E−01	0.02	183.3	5.04.E−01	89.94
183.5	3.86.E−01	14.29	185.2	3.77.E−01	−13.00	183.6	1.96.E−01	31.35
186.7	3.49.E−03	−0.06	186.9	7.23.E−03	3.65	186.5	3.56.E−03	−4.34
187.0	1.91.E−02	48.74	187.5	3.22.E−02	−47.78	187.1	2.59.E−02	−55.20
190.1	8.70.E−03	−8.39	189.3	1.56.E−02	5.36	190.1	9.99.E−03	9.70
191.4	2.69.E−02	−4.64	190.9	2.13.E−02	18.77	191.4	2.64.E−02	4.99
192.4	1.74.E−02	−17.83	192.1	4.44.E−02	20.46	192.8	1.99.E−02	13.32
193.9	1.95.E−02	11.24	194.1	4.10.E−02	1.31	194.0	2.00.E−02	−7.83
196.7	2.05.E−02	−3.74	195.8	3.56.E−03	2.76	196.8	5.06.E−02	6.88
196.8	2.97.E−02	−2.32	199.8	1.11.E−02	9.17	197.1	2.54.E−03	1.42
205.7	6.40.E−03	−11.81	206.1	1.80.E−02	11.55	205.8	1.16.E−02	12.07
207.7	1.17.E−02	4.41	208.7	1.25.E−02	−6.51	207.9	8.27.E−03	−0.65
216.5	7.99.E−02	51.89	214.6	3.15.E−02	−26.43	216.4	7.76.E−02	−40.04
231.2	2.70.E−01	100.91	230.2	3.67.E−01	−75.47	231.3	2.47.E−01	−114.25
233.5	7.96.E−02	−81.94	233.3	2.69.E−02	48.17	233.9	1.03.E−01	88.06
249.3	1.21.E−02	7.63	248.6	6.50.E−03	−7.04	249.4	9.73.E−03	−9.13
M0026			M0008			M0004		
Wavelength (nm)	Oscillator strength	Rotatory strength	Wavelength (nm)	Oscillator strength	Rotatory strength	Wavelength (nm)	Oscillator strength	Rotatory strength
170.2	4.80.E−02	−49.49	170.0	8.11.E−02	−44.54	168.4	6.70.E−02	1.71
171.3	8.20.E−02	−9.03	171.4	2.47.E−02	6.12	169.8	4.57.E−02	61.02
180.3	1.32.E−02	−7.82	177.8	3.09.E−01	28.99	178.1	2.33.E−01	−32.68

182.0	2.09.E−01	−52.71	178.5	2.13.E−01	−38.68	178.5	2.12.E−01	−75.46
183.3	5.03.E−01	90.73	179.3	1.69.E−02	44.36	180.1	1.06.E−01	74.81
183.6	1.96.E−01	30.78	180.3	7.57.E−02	14.89	180.6	5.78.E−02	−72.26
186.5	3.53.E−03	−4.36	180.6	3.84.E−02	−24.67	181.1	1.92.E−02	2.30
187.1	2.59.E−02	−55.13	183.2	1.28.E−01	33.00	181.6	1.00.E−01	−7.96
190.1	1.01.E−02	9.74	187.9	7.71.E−02	17.74	188.2	6.85.E−02	−25.75
191.4	2.64.E−02	4.99	191.4	1.31.E−01	20.07	191.1	1.59.E−01	47.16
192.8	1.97.E−02	13.37	191.8	6.37.E−02	86.69	192.1	2.72.E−02	−33.34
194.0	2.02.E−02	−7.80	192.5	6.01.E−02	−230.02	192.7	7.96.E−02	133.65
196.8	5.11.E−02	6.92	197.4	3.64.E−02	9.73	199.4	3.55.E−02	−31.59
197.1	2.55.E−03	1.43	201.1	7.85.E−02	71.92	201.2	9.44.E−02	−48.11
205.8	1.15.E−02	12.01	204.0	8.41.E−03	5.77	204.8	1.85.E−03	−2.87
207.9	8.32.E−03	−0.64	209.7	1.35.E−02	−4.70	210.0	2.44.E−02	15.52
216.4	7.79.E−02	−40.07	218.9	1.45.E−01	−2.07	221.1	1.61.E−01	−3.95
231.3	2.47.E−01	−114.21	232.6	1.30.E−01	69.88	234.8	1.16.E−01	−76.40
233.9	1.03.E−01	87.84	237.0	1.21.E−01	−46.92	238.1	1.41.E−01	30.97
249.4	9.95.E−03	−9.14	252.7	3.69.E−02	0.75	253.1	2.83.E−02	−3.00

M0007			M0017		
Wavelength (nm)	Oscillator strength	Rotatory strength	Wavelength (nm)	Oscillator strength	Rotatory strength
168.5	5.48.E−02	23.87	169.7	3.61.E−03	2.15
169.5	1.39.E−02	9.90	172.4	4.36.E−02	7.36
177.1	2.66.E−01	20.18	177.5	3.07.E−01	97.67
177.7	2.22.E−01	−45.33	178.4	3.35.E−01	27.80
179.6	5.64.E−02	60.61	181.9	9.70.E−04	−6.95
179.8	5.36.E−02	−47.43	182.9	4.94.E−02	23.27
180.5	3.84.E−03	−20.20	184.4	4.75.E−03	−6.96
180.6	6.67.E−02	−11.05	185.7	1.06.E−01	−44.36
188.2	6.23.E−02	−21.28	186.7	5.49.E−02	−25.84
190.1	1.79.E−01	56.09	190.0	2.99.E−02	−15.81
191.7	3.05.E−02	−63.10	191.7	5.96.E−02	6.51
192.8	9.32.E−02	127.72	192.7	2.27.E−02	−60.77
199.1	7.17.E−02	−69.41	199.0	9.11.E−02	−18.84
201.8	7.71.E−02	−21.57	202.1	8.06.E−02	60.36
205.6	3.74.E−03	−2.14	204.2	1.15.E−02	4.42
210.5	1.97.E−02	19.60	210.1	2.27.E−02	−12.29
222.1	1.86.E−01	−5.57	217.7	1.36.E−01	23.73
236.1	4.15.E−02	−44.29	233.2	9.53.E−02	69.41
239.3	1.93.E−01	8.50	240.6	1.56.E−01	−9.93
253.0	1.91.E−02	−2.67	253.1	2.86.E−02	15.73

8. Docking study and MD simulation

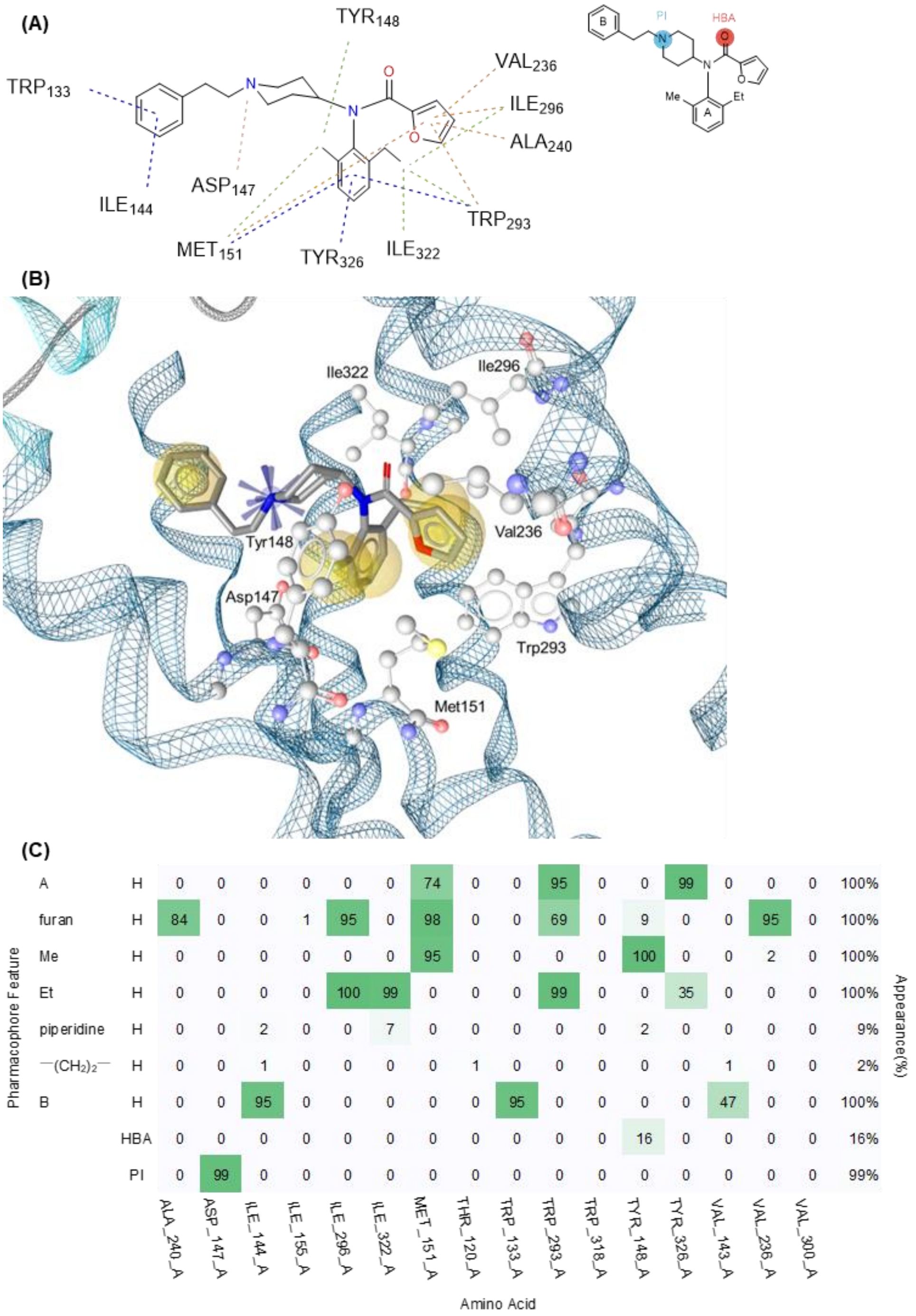

Figure S18. Interactions of ligands with peripheral amino acid residues in a*R*-**31** complex structures predicted via docking studies and MD simulations for the MOR (PDB: 5C1M). (A) 2D conformations, (B) 3D pharmacophore conformations, and (C) MD pharmacophore plots of a*R*-**31**. Interactions of the ligands with a*R*-**31** were assessed via LigandScout–assisted MD pharmacophore analyses. Subsequently, pharmacophore models of the cluster with the highest frequency of occurrence (401st frame) were adopted from the cluster analysis results based on MD trajectories. For the MD pharmacophore plot, all trajectories were used.

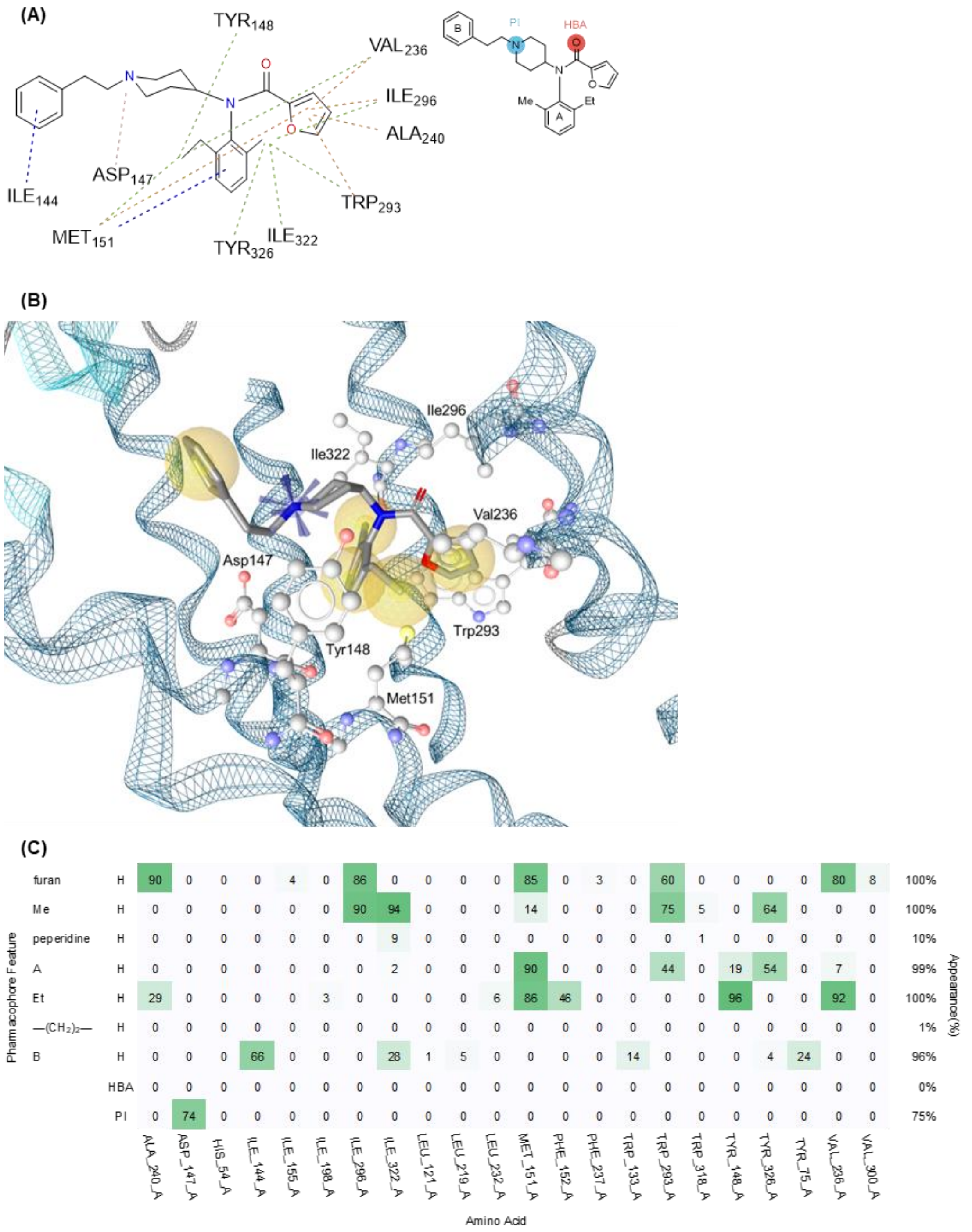

Figure S19. Interactions of ligands with peripheral amino acid residues in a*S*-**31** complex structures predicted via docking studies and MD simulations for the MOR (PDB: 5C1M). (A) 2D conformations, (B) 3D pharmacophore conformations, and (C) MD pharmacophore plots of a*S*-**31**. Interactions of the ligands with a*S*-**31** were assessed via LigandScout−assisted MD pharmacophore analyses. Subsequently, pharmacophore models of the cluster with the highest frequency of occurrence (399th frame) were adopted from the cluster analysis results based on MD trajectories. For the MD pharmacophore plot, all trajectories were used.

Table S6. Screening results for rigid (ligand conformation) docking sorted by binding energy.

#	Name	Binding energy [kcal/mol]	Ligand efficiency [kcal/(mol*Atom)]	Dissociation constant [pM]
1	conf325	9.988	0.3222	47719.8359
2	conf466	9.630	0.3106	87320.5391
3	conf69	9.375	0.3024	134286.9688
4	conf478	9.223	0.2975	173560.6875
5	conf183	9.138	0.2948	200335.1719
6	conf77	8.924	0.2879	287489.0312
7	conf132	8.810	0.2842	348485.2188
8	conf64	8.728	0.2815	400212.9688
9	conf72	8.713	0.2811	410474.625
10	conf160	8.694	0.2805	423851.2812
11	conf423	8.632	0.2785	470608.875
12	conf424	8.605	0.2776	492551.1875
13	conf185	8.597	0.2773	499246.9688
14	conf92	8.573	0.2765	519885.4688
15	conf397	8.569	0.2764	523407.2188
16	conf469	8.555	0.2760	535922.3125
17	conf83	8.375	0.2702	726178.875
18	conf53	8.372	0.2701	729865.1875
19	conf25	8.351	0.2694	756198.5625
20	conf441	8.336	0.2689	775587.8125
21	conf104	8.304	0.2679	818629.3125
22	conf116	8.301	0.2678	822784.9375
23	conf294	8.267	0.2667	871382.125
24	conf61	7.967	0.257	1445811.125
25	conf130	7.790	0.2513	1949190.5
26	conf16	7.555	0.2437	2898088
27	conf110	7.528	0.2428	3033212.5
28	conf42	7.395	0.2385	3796585
29	conf4	7.329	0.2364	4243965
30	conf169	7.286	0.235	4563428
31	conf163	7.165	0.2311	5597387.5
32	conf354	7.078	0.2283	6482719
33	conf278	7.061	0.2278	6671421.5
34	conf455	7.030	0.2268	7029779
35	conf216	7.010	0.2261	7271129.5
36	conf103	6.993	0.2256	7482781
37	conf332	6.978	0.2251	7674643
38	conf375	6.868	0.2215	9240368
39	conf146	6.847	0.2209	9573758

40	conf229	6.837	0.2205	9736717
41	conf221	6.815	0.2198	10105057
42	conf184	6.757	0.218	11144314
43	conf417	6.757	0.218	11144314
44	conf67	6.745	0.2176	11372330
45	conf350	6.740	0.2174	11468708
46	conf300	6.735	0.2173	11565903
47	conf341	6.727	0.217	11723131
48	conf262	6.655	0.2147	13237937
49	conf297	6.636	0.2141	13669339
50	conf219	6.600	0.2129	14525659

Table S7. Binding energies calculated via 10−ns MD simulations of each conformer, sorted by average binding energy obtained from MD trajectories of the last 5 ns

#	Name	Average (total)	Average (second half)	Max Binding Energy (kJ/mol)	at Time (ps)
		Binding Energy (kJ/mol)	Binding Energy (kJ/mol)		
1	conf424 (a*S*)	1492.315	1527.20155	1718.600	5000
2	conf423 (a*S*)	1340.569	1351.14725	1525.160	8250
3	conf132 (a*R*)	1282.697	1305.80235	1596.765	6500
4	conf25 (a*R*)	1297.472	1279.00015	1459.537	1500
5	conf116 (a*S*)	1211.793	1251.59475	1405.889	9750
6	conf183 (a*S*)	1110.013	1249.95185	1419.082	7500
7	conf294 (a*R*)	1176.669	1215.26530	1341.103	9000
8	conf185 (a*S*)	1266.052	1213.39835	1532.951	3750
9	conf478 (a*S*)	1236.133	1212.00600	1483.836	750
10	conf92 (a*R*)	1199.256	1208.82945	1439.531	0
11	conf466 (a*S*)	1230.570	1194.06710	1550.336	1750
12	conf397 (a*S*)	1185.099	1151.44655	1393.477	0
13	conf160 (a*S*)	1132.325	1144.41215	1362.115	0
14	conf104 (a*R*)	1074.786	1122.11475	1385.968	7500
15	conf441 (a*R*)	1087.570	1103.00340	1432.747	0
16	conf325 (a*R*)	1090.479	1081.25000	1377.285	3500
17	conf469 (a*S*)	1115.818	1078.88910	1436.735	750
18	conf53 (a*R*)	1081.934	1076.15990	1338.571	750
19	conf83 (a*R*)	1069.768	1046.87930	1343.475	2500
20	conf72 (a*S*)	1066.975	1013.62570	1239.848	250
21	conf69 (a*S*)	964.173	988.57705	1345.062	0
22	conf77 (a*S*)	841.582	800.36345	1401.467	0
23	conf64 (a*R*)	859.220	778.32210	1160.749	0

9. ^{1}H-, ^{13}C-, 2D-NMR and HRMS Spectra

^{1}H NMR spectrum of compound **1** ($CDCl_3$)

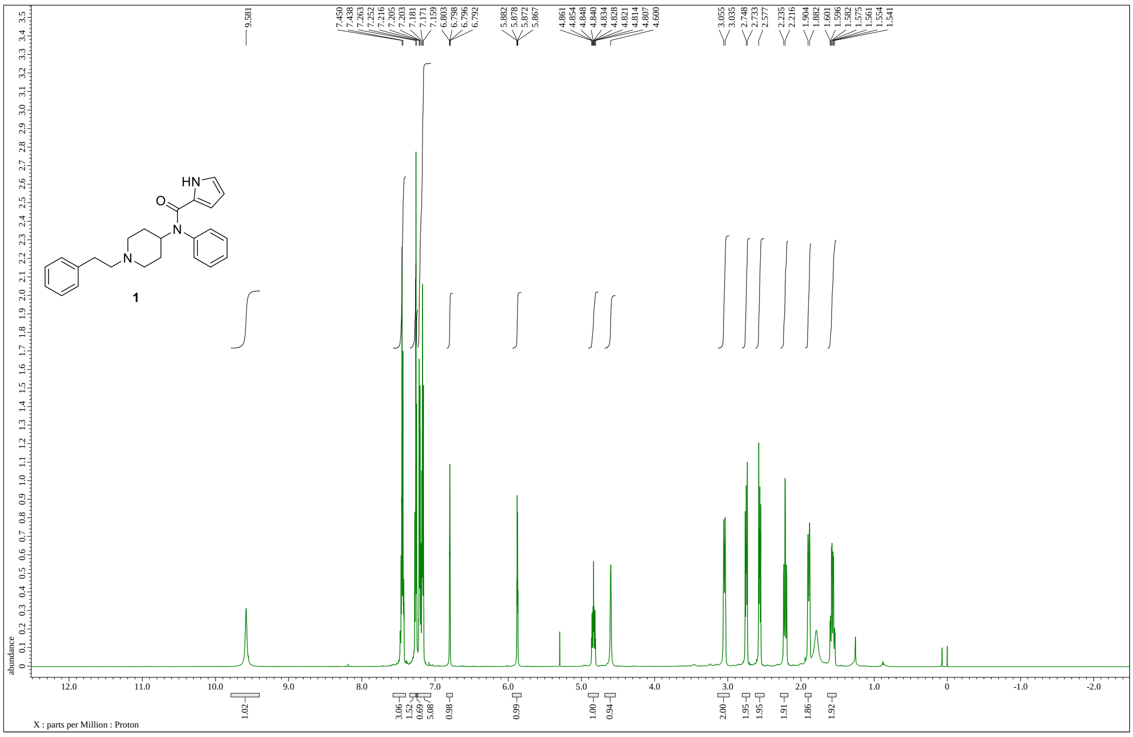

^{13}C NMR spectrum of compound **1** ($CDCl_3$)

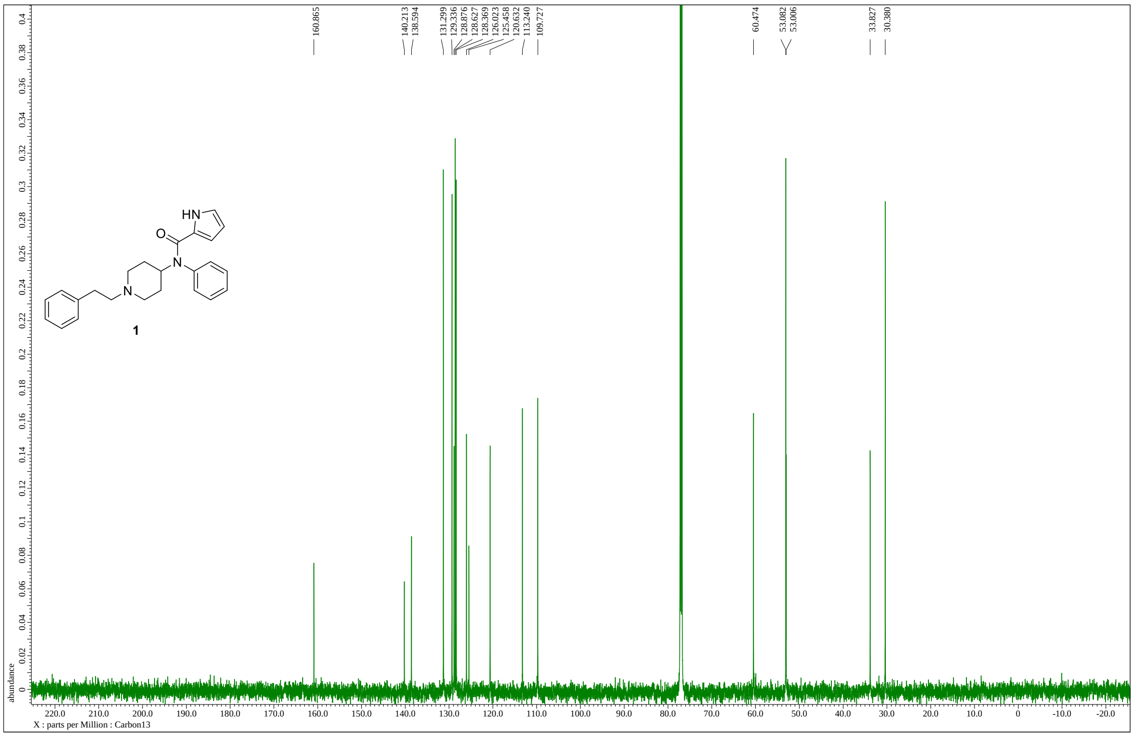

HRMS of compound **1**

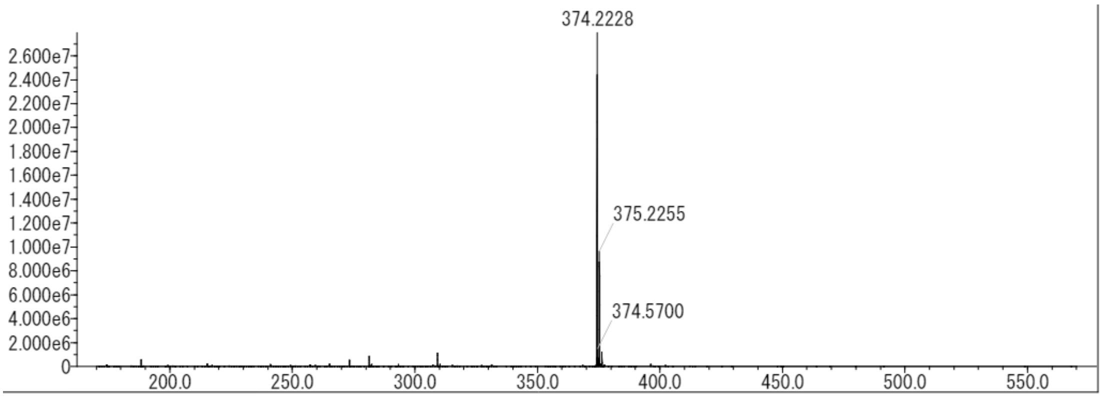

Formula $[M+H]^+$	Theoretical *m/z*	Found *m/z*
$C_{24}H_{28}N_3O$	374.2227	374.2228

^{1}H NMR spectrum of compound **2** ($CDCl_3$)

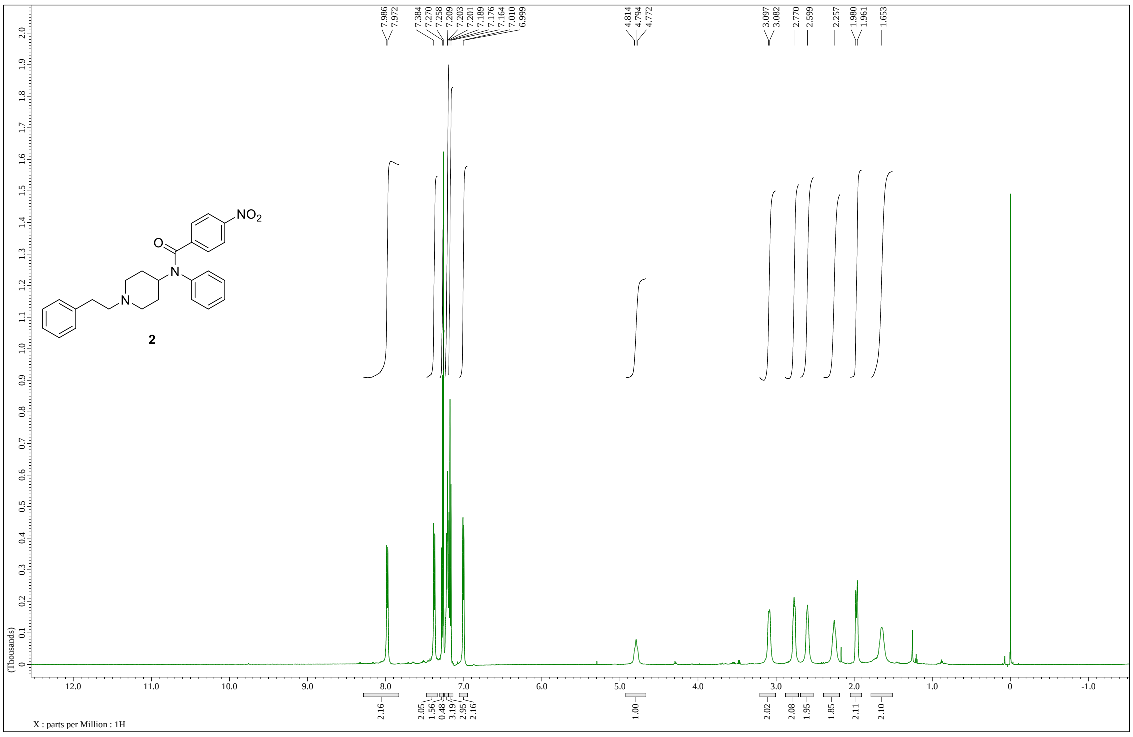

^{13}C NMR spectrum of compound **2** ($CDCl_3$)

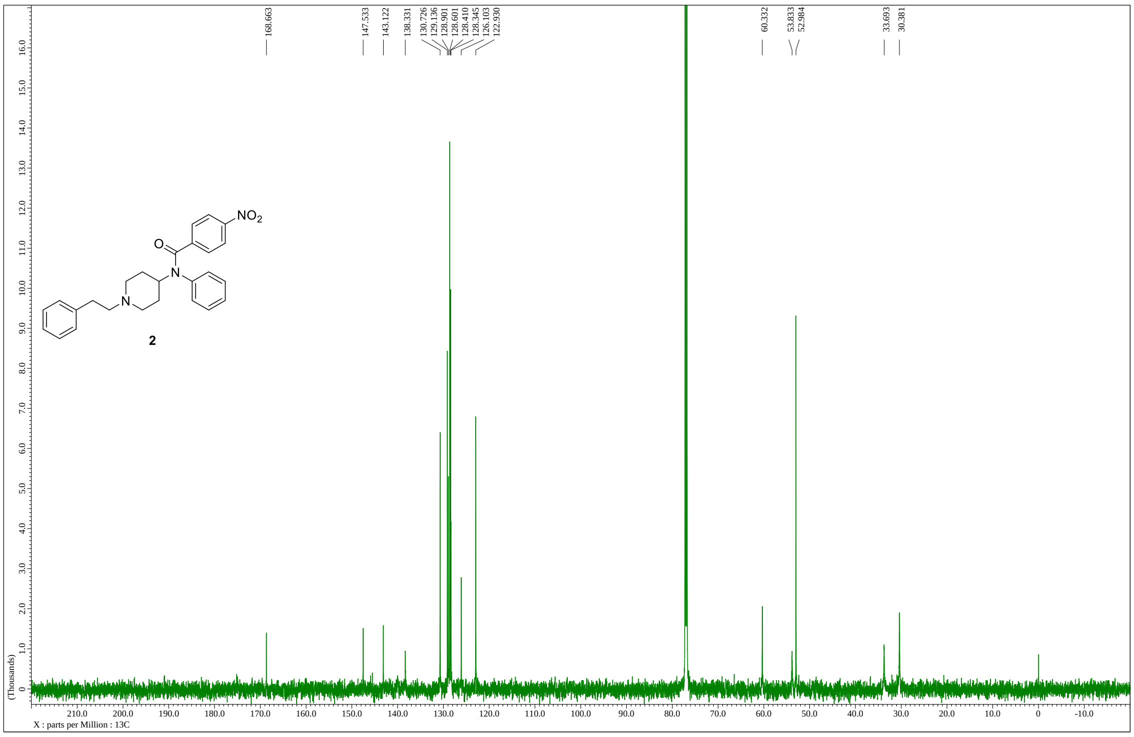

HRMS of compound **2**

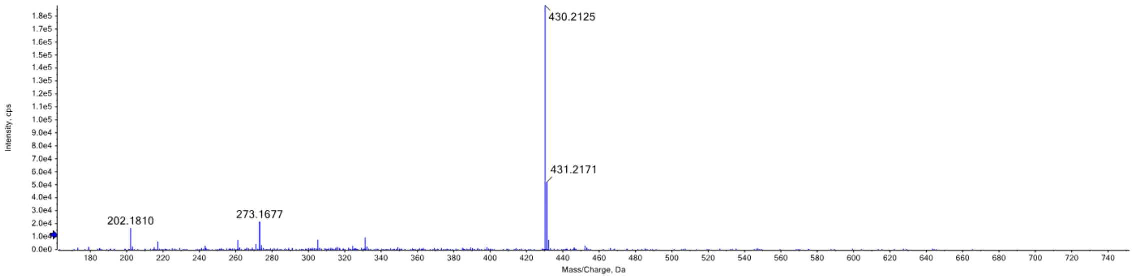

Formula $[M+H]^+$	Theoretical *m/z*	Found *m/z*
$C_{26}H_{28}N_3O_3$	430.2125	430.2125

^{1}H NMR spectrum of compound **3** ($CDCl_3$)

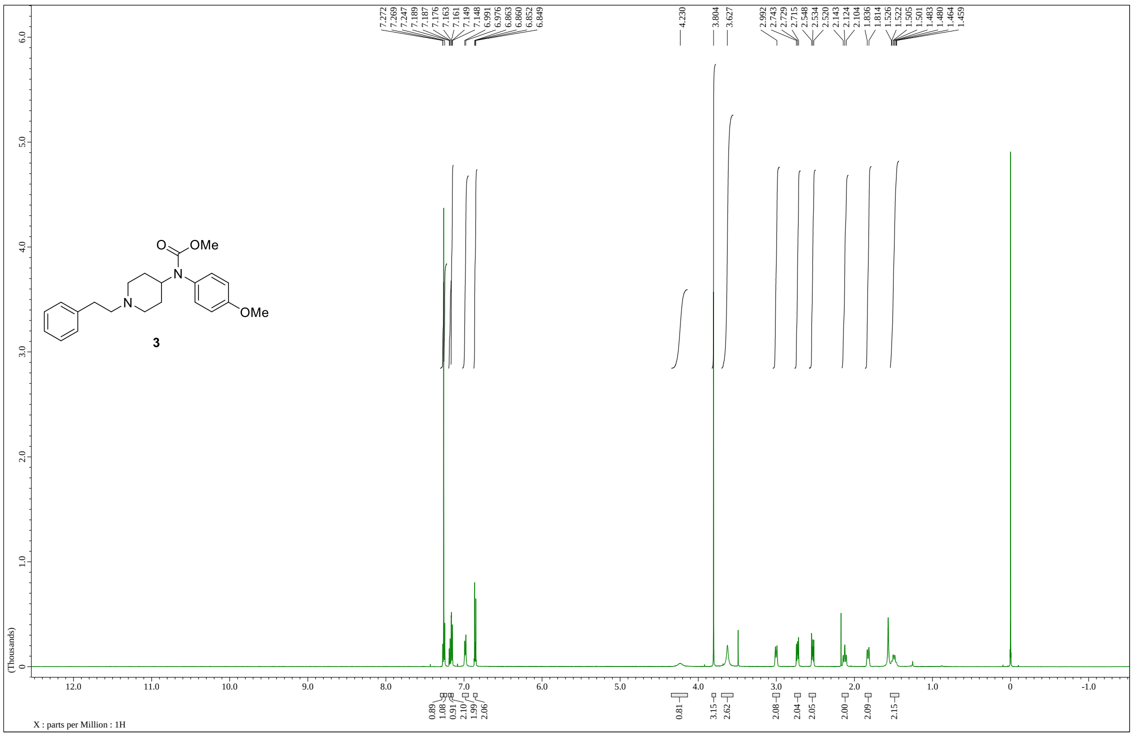

^{13}C NMR spectrum of compound **3** ($CDCl_3$)

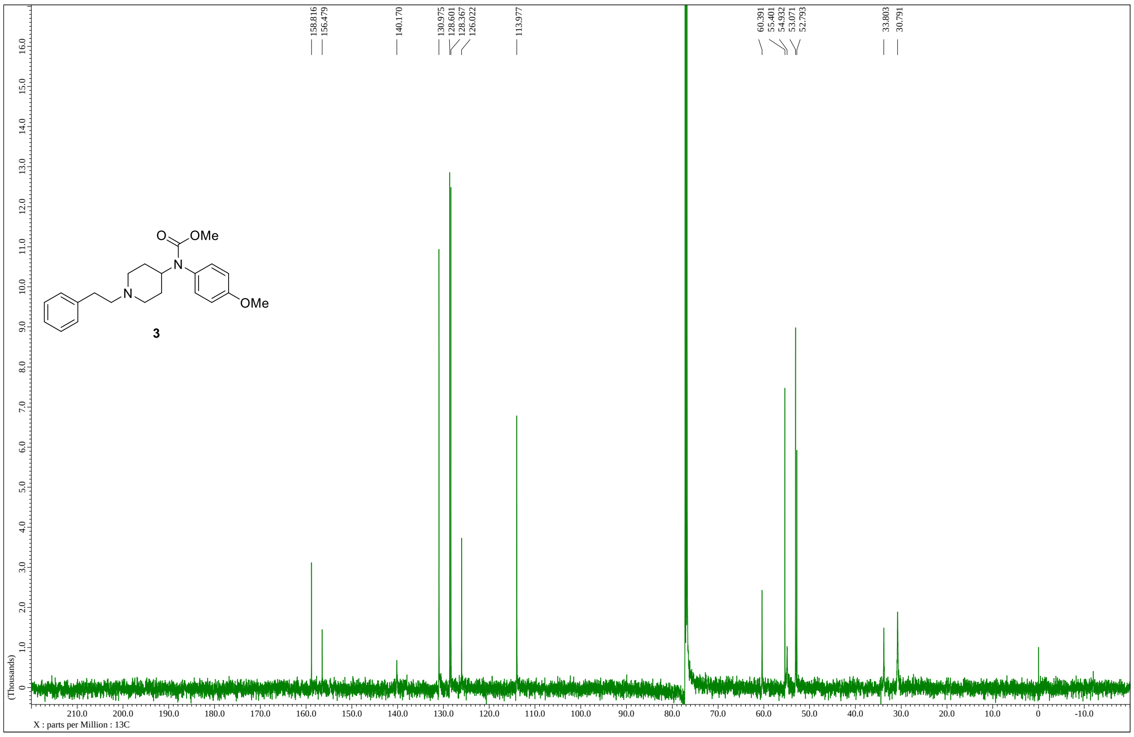

HRMS of compound **3**

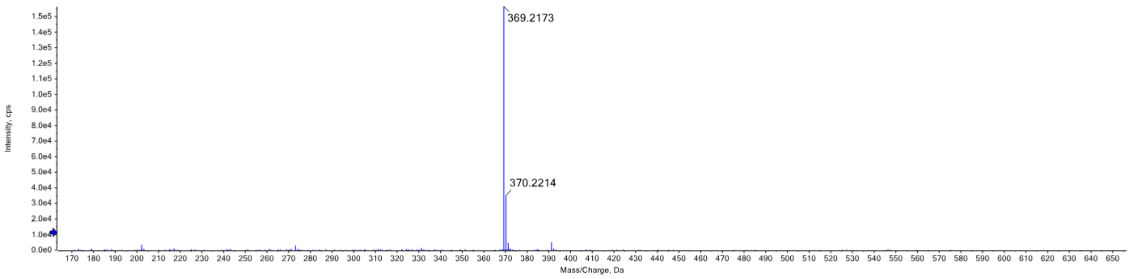

Formula [M+H]⁺	Theoretical *m/z*	Found *m/z*
$C_{22}H_{29}N_2O_3$	369.2173	369.2173

^{1}H NMR spectrum of compound **4** ($CDCl_3$)

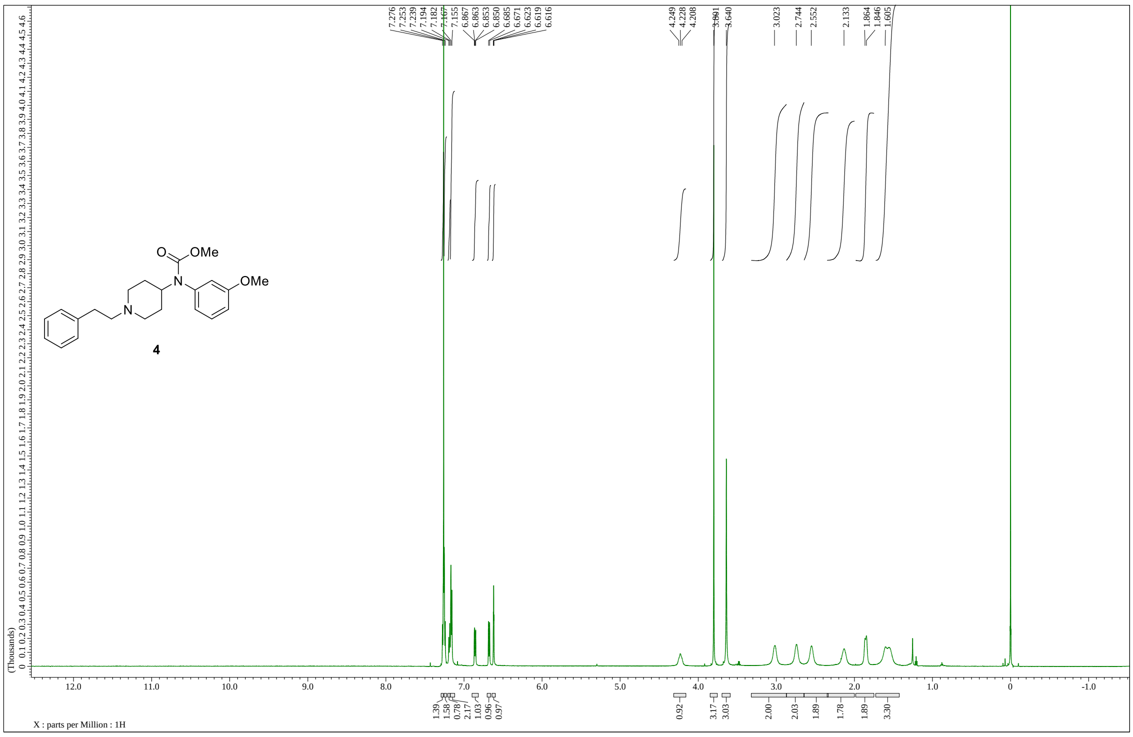

^{13}C NMR spectrum of compound **4** ($CDCl_3$)

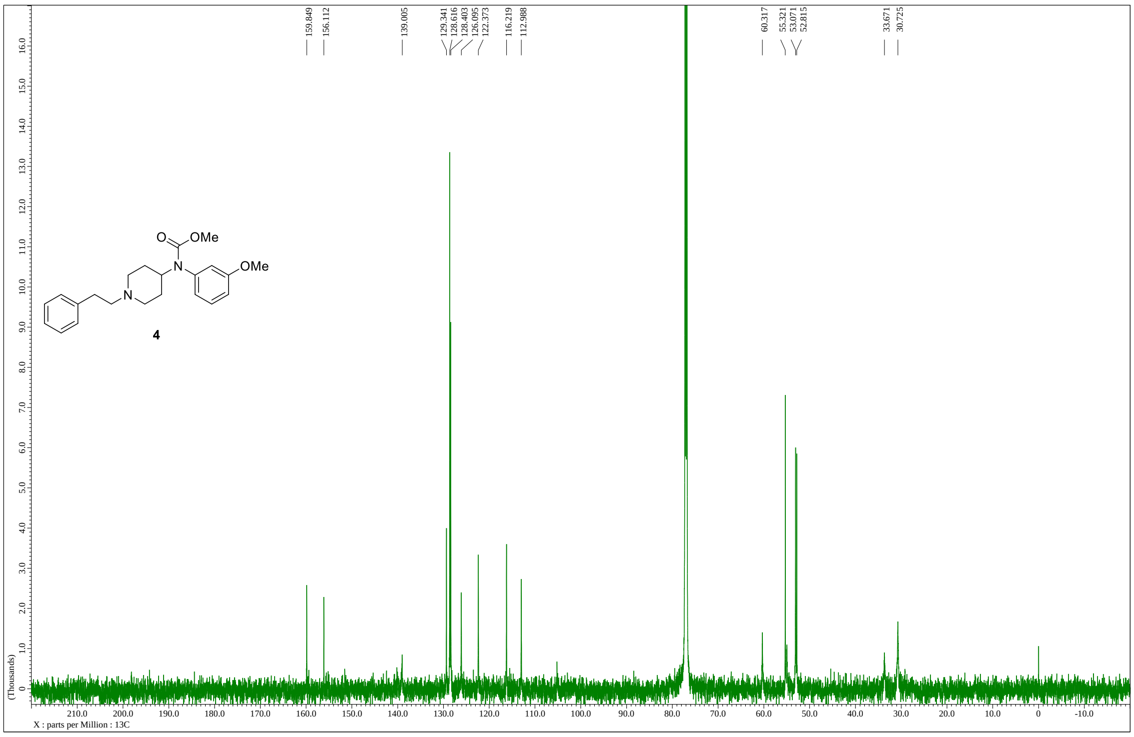

HRMS of compound **4**

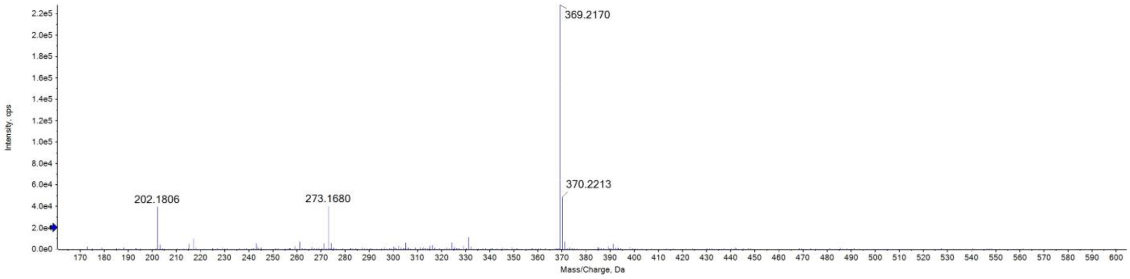

Formula [M+H]$^+$	Theoretical *m/z*	Found *m/z*
$C_{22}H_{29}N_2O_3$	369.2173	369.2170

^{1}H NMR spectrum of compound **5** ($CDCl_3$)

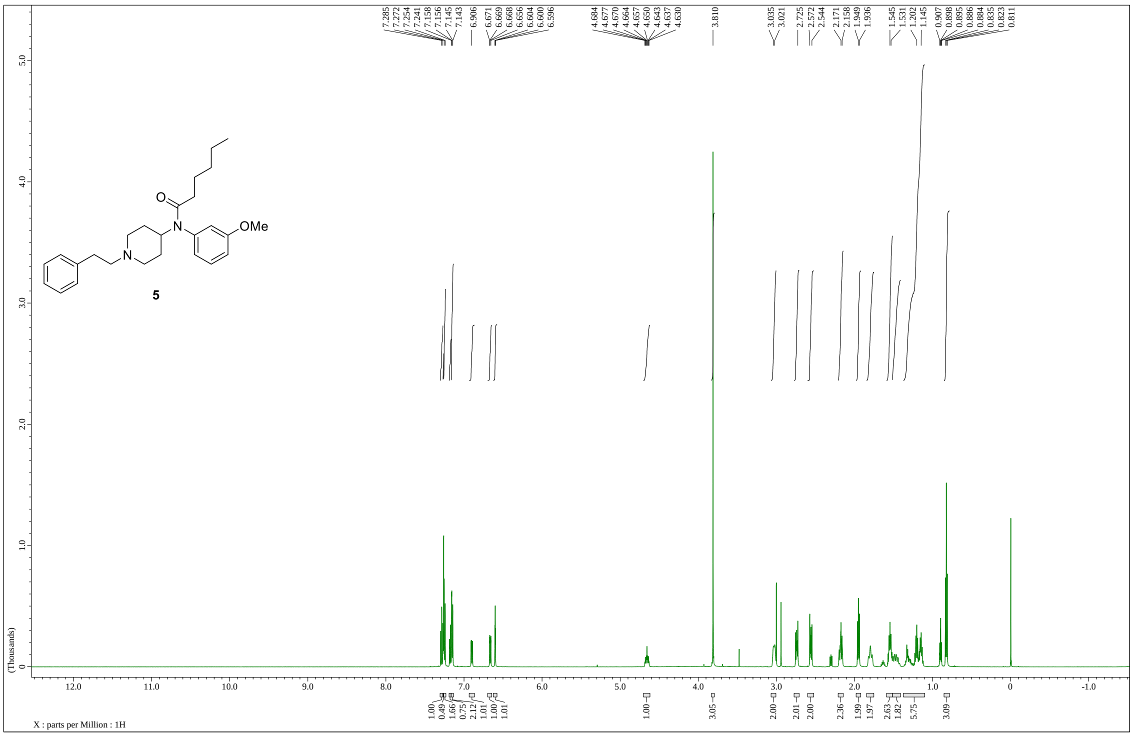

^{13}C NMR spectrum of compound **5** ($CDCl_3$)

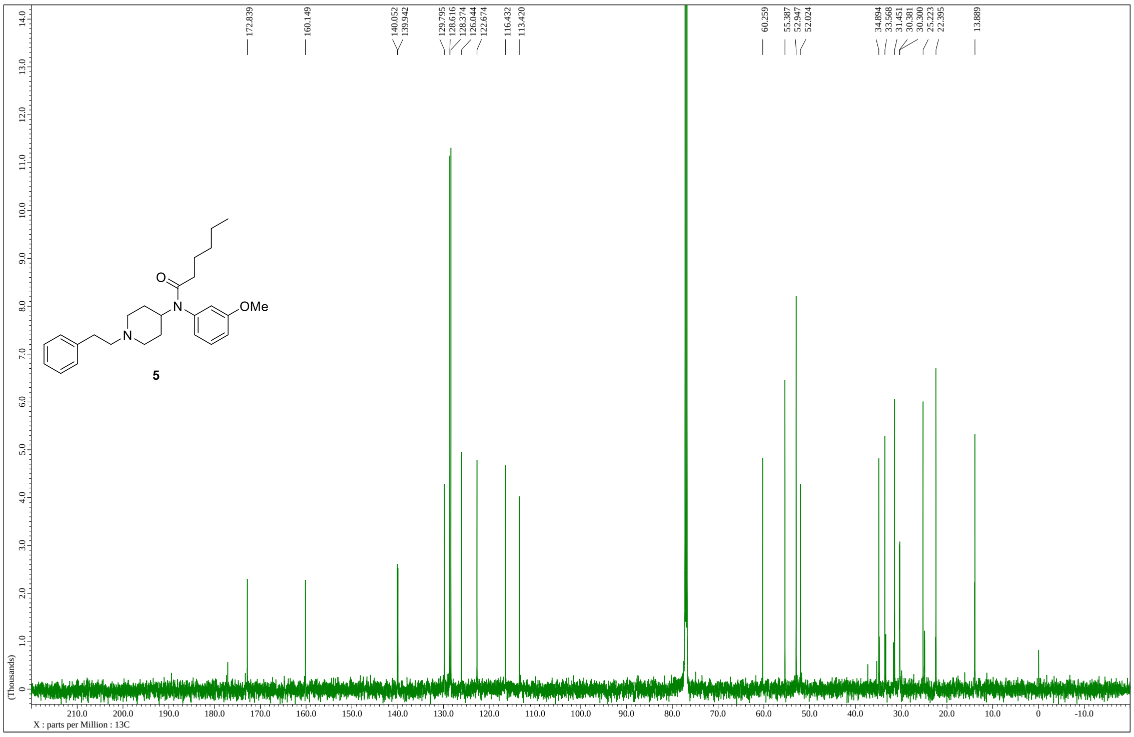

HRMS of compound **5**

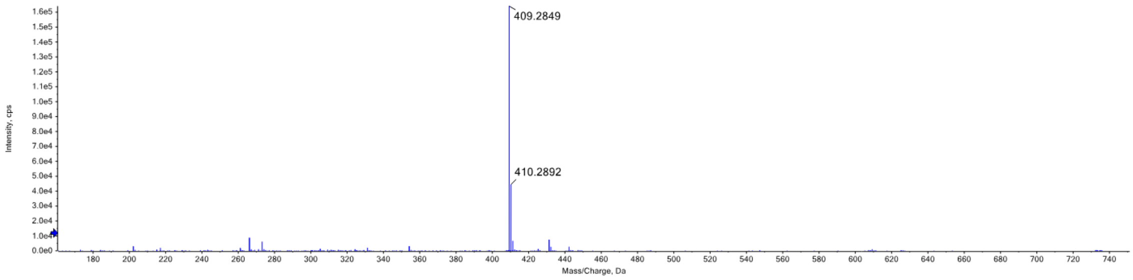

Formula [M+H]+	Theoretical *m/z*	Found *m/z*
$C_{26}H_{37}N_2O_2$	409.2850	409.2849

^{1}H NMR spectrum of compound **6** ($CDCl_3$)

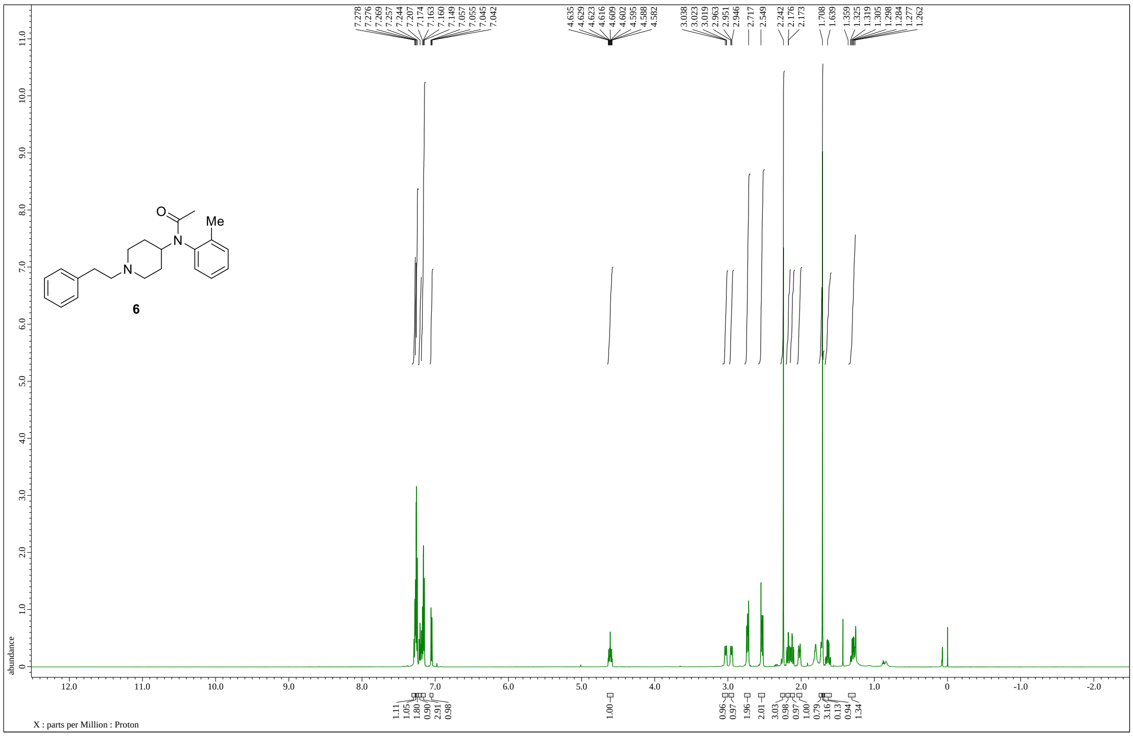

^{13}C NMR spectrum of compound **6** ($CDCl_3$)

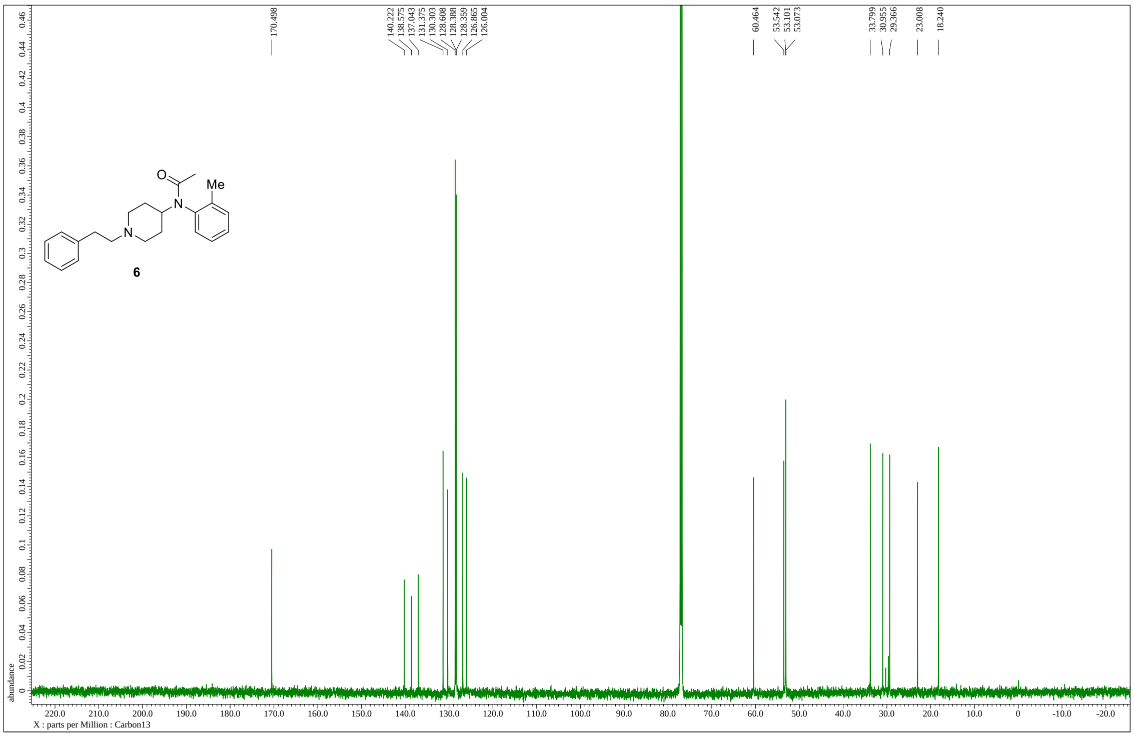

HRMS of compound **6**

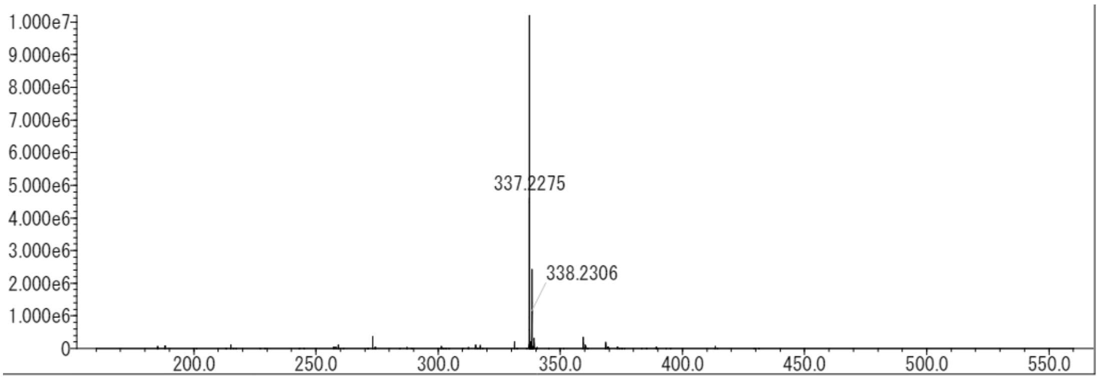

Formula $[M+H]^+$	Theoretical m/z	Found m/z
$C_{22}H_{29}N_2O$	337.2274	337.2275

^{1}H NMR spectrum of compound **7** ($CDCl_3$)

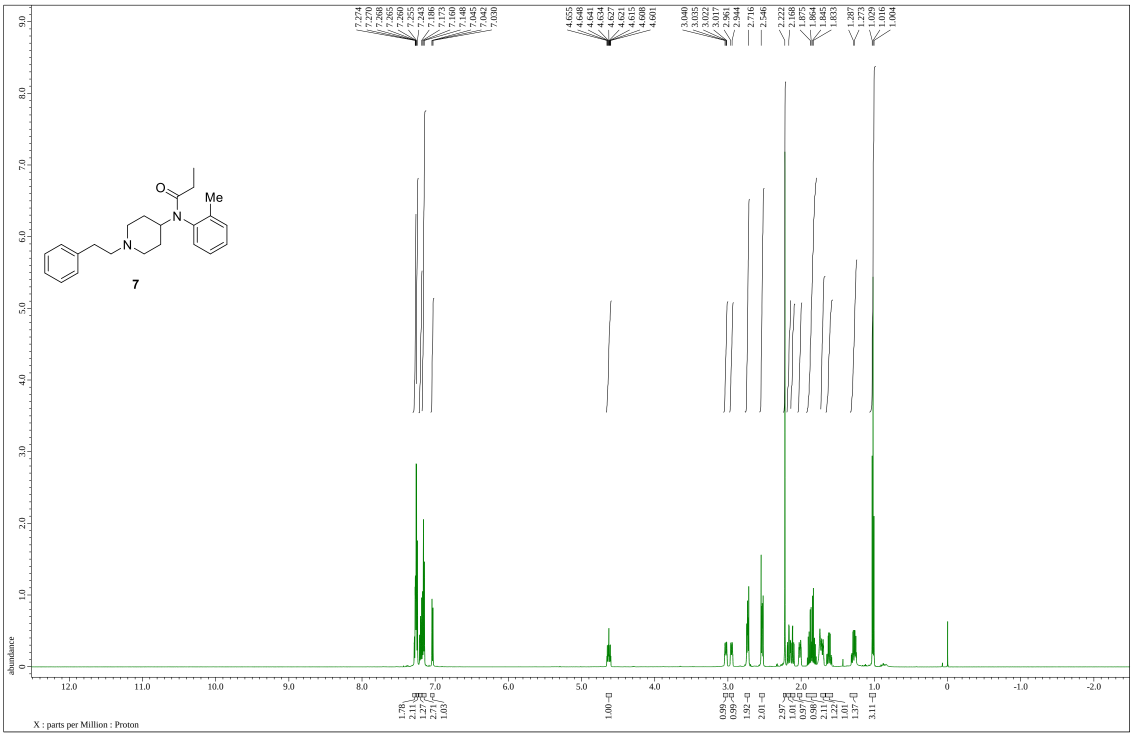

^{13}C NMR spectrum of compound **7** ($CDCl_3$)

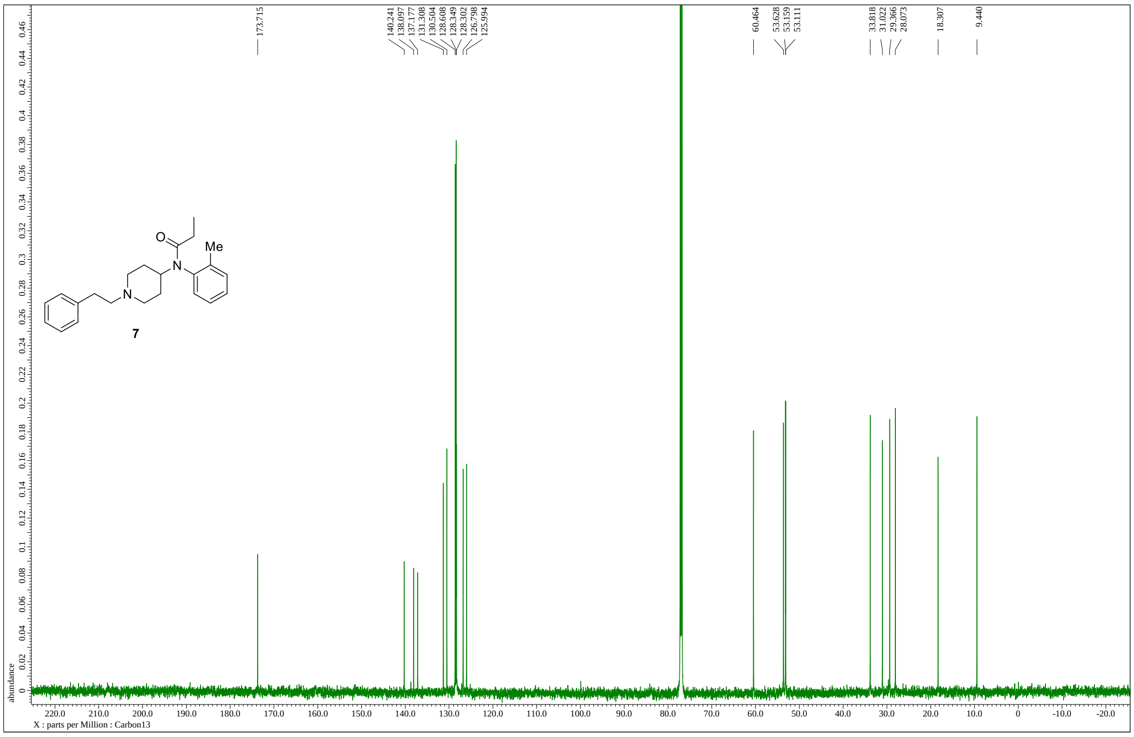

HRMS of compound **7**

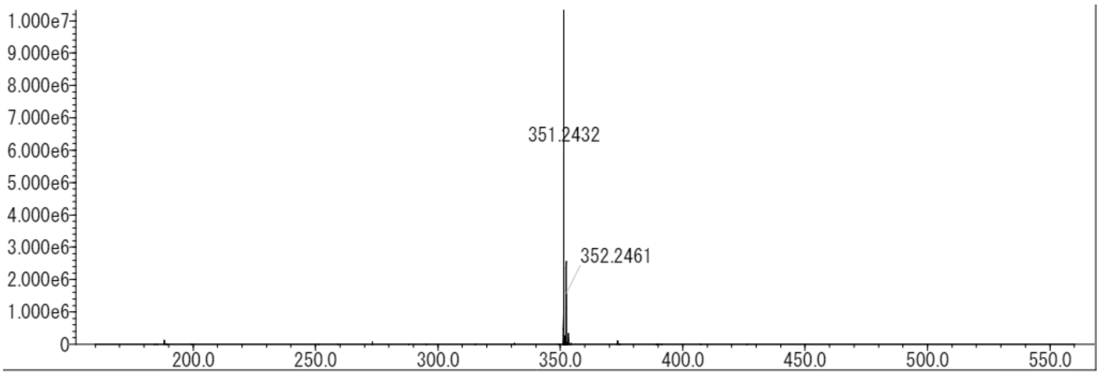

Formula [M+H]+	Theoretical *m/z*	Found *m/z*
$C_{23}H_{31}N_2O$	351.2431	351.2432

^{1}H NMR spectrum of compound **8** ($CDCl_3$)

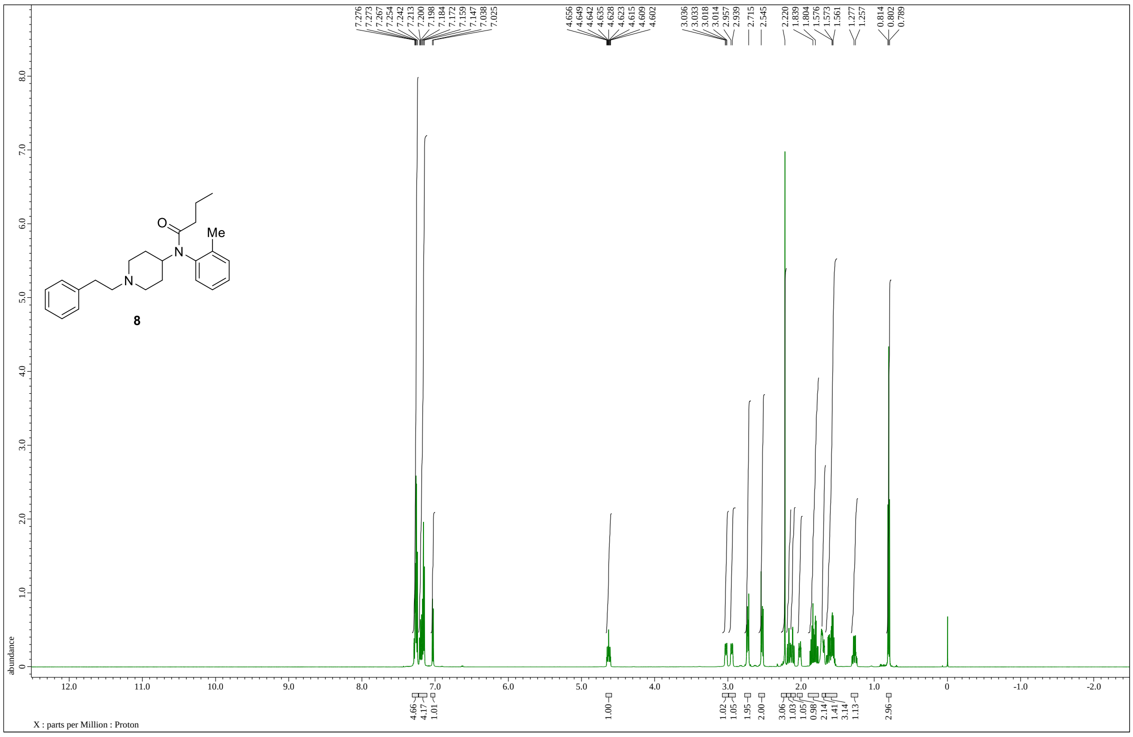

^{13}C NMR spectrum of compound **8** ($CDCl_3$)

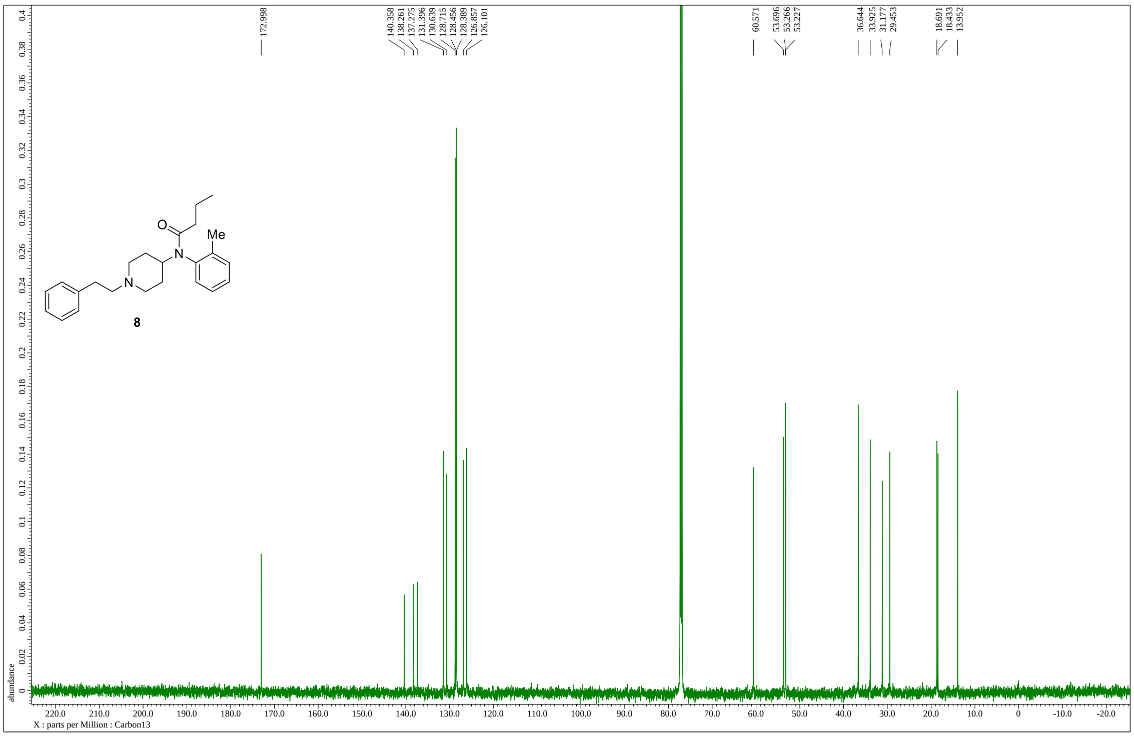

HRMS of compound **8**

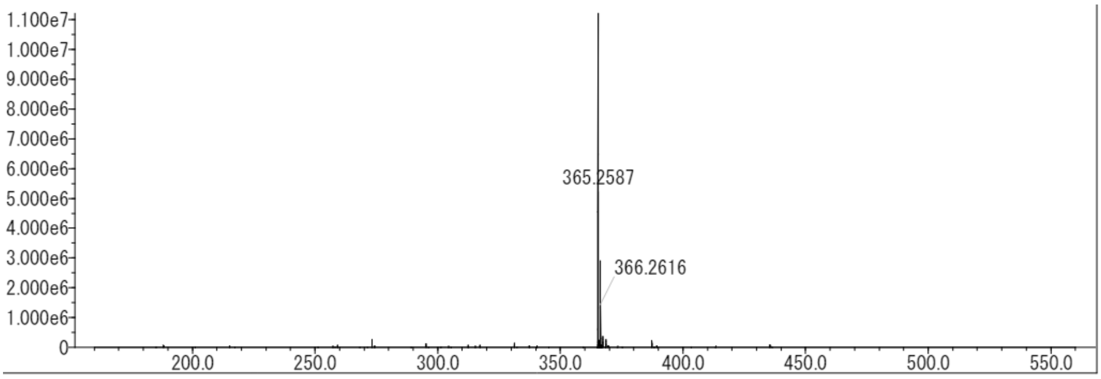

Formula $[M+H]^+$	Theoretical *m/z*	Found *m/z*
$C_{24}H_{33}N_2O$	365.2587	365.2587

^{1}H NMR spectrum of compound **9** ($CDCl_3$)

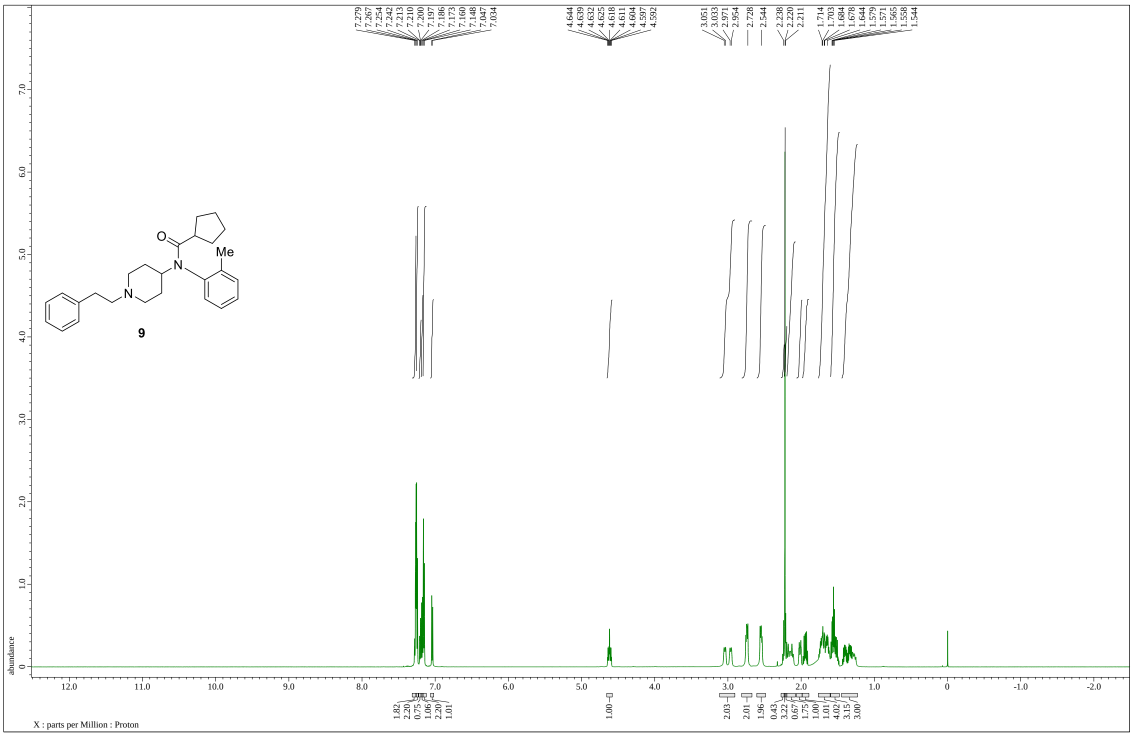

^{13}C NMR spectrum of compound **9** ($CDCl_3$)

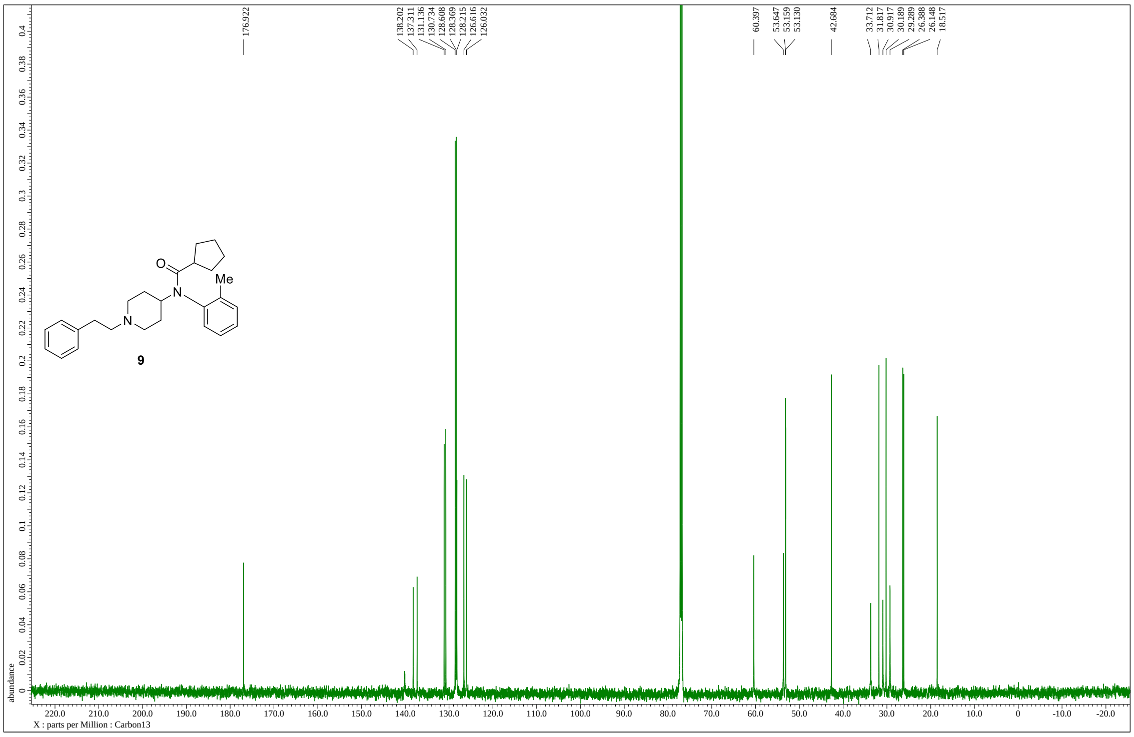

HRMS of compound **9**

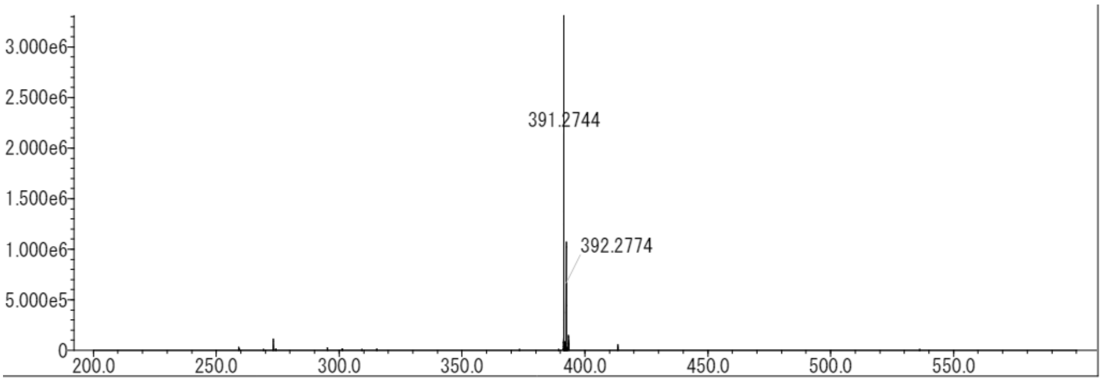

Formula [M+H]+	Theoretical *m/z*	Found *m/z*
$C_{26}H_{35}N_2O$	391.2744	391.2744

^{1}H NMR spectrum of compound **10** ($CDCl_3$)

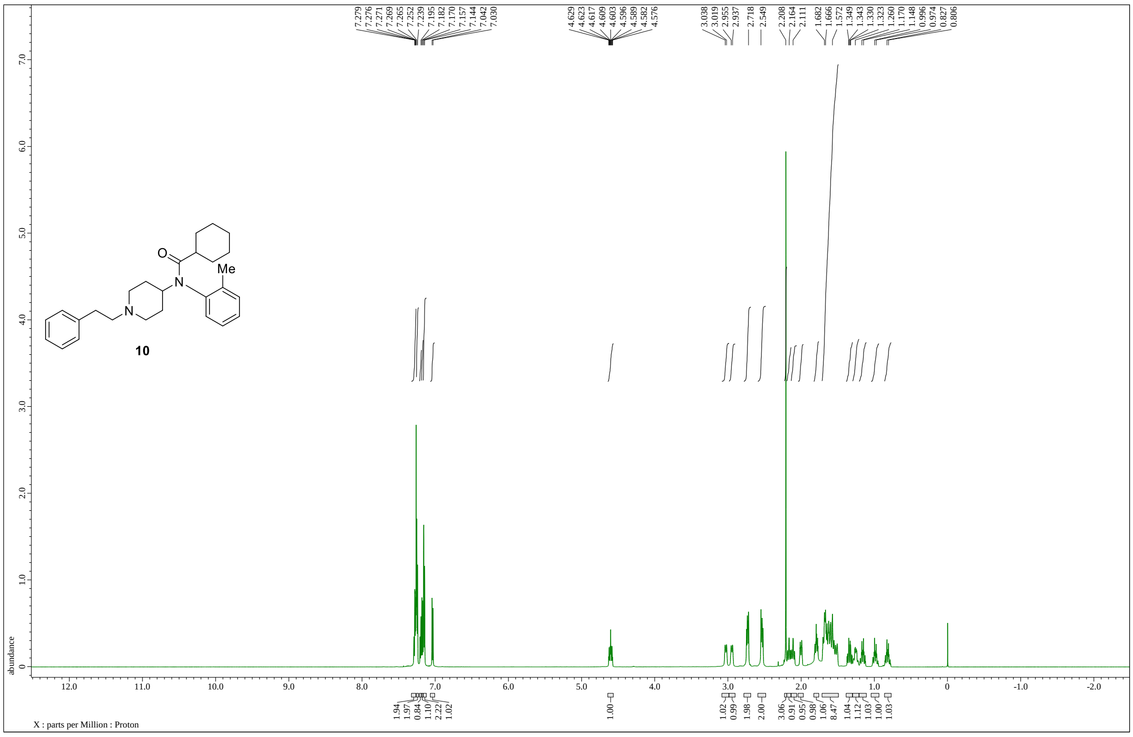

^{13}C NMR spectrum of compound **10** ($CDCl_3$)

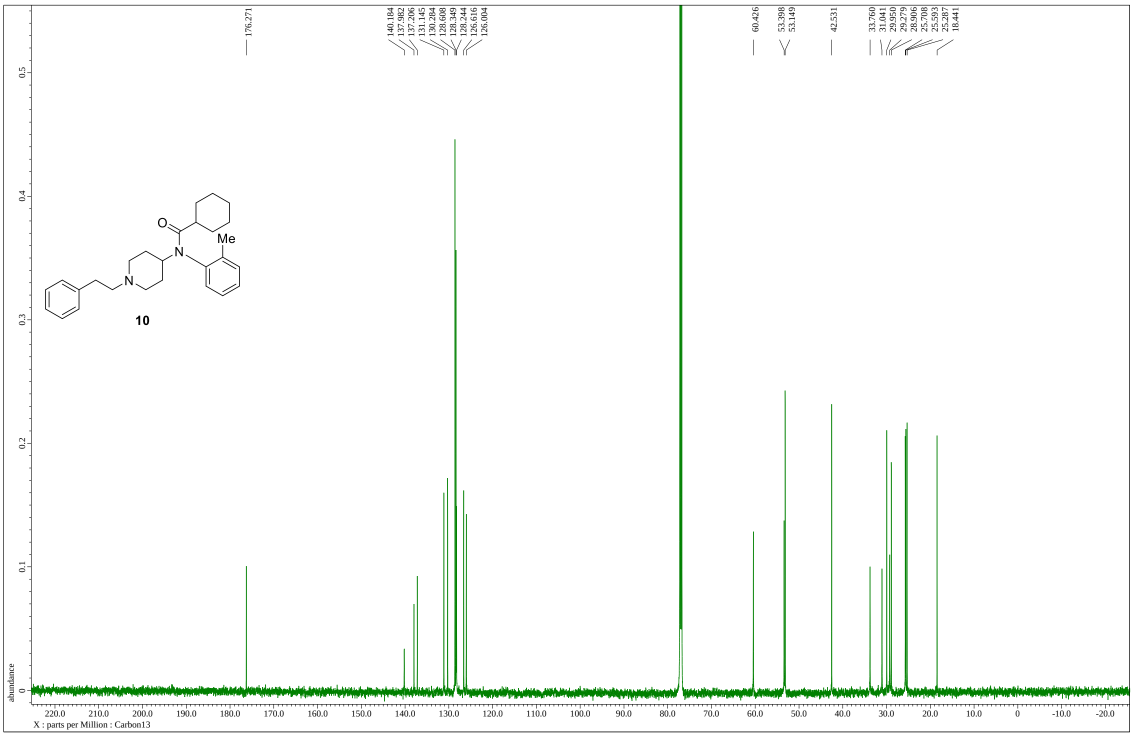

HRMS of compound **10**

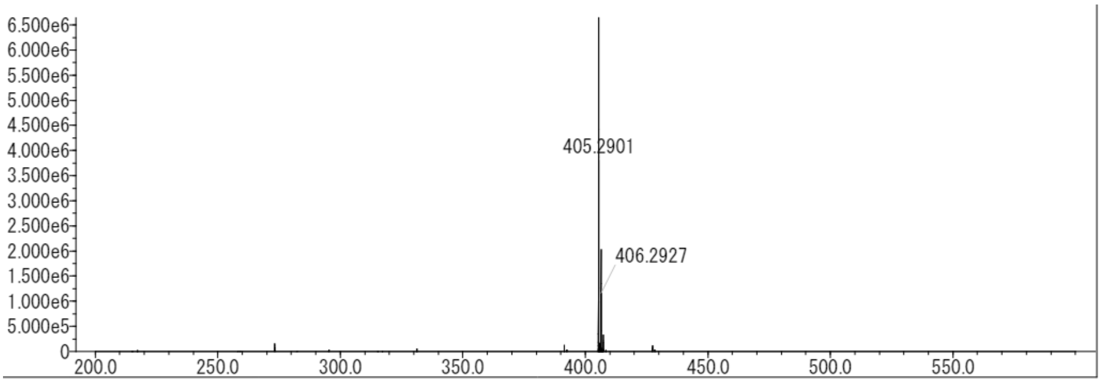

Formula [M+H]⁺	Theoretical *m/z*	Found *m/z*
$C_{27}H_{37}N_2O$	405.2900	405.2901

^{1}H NMR spectrum of compound **11** ($CDCl_3$)

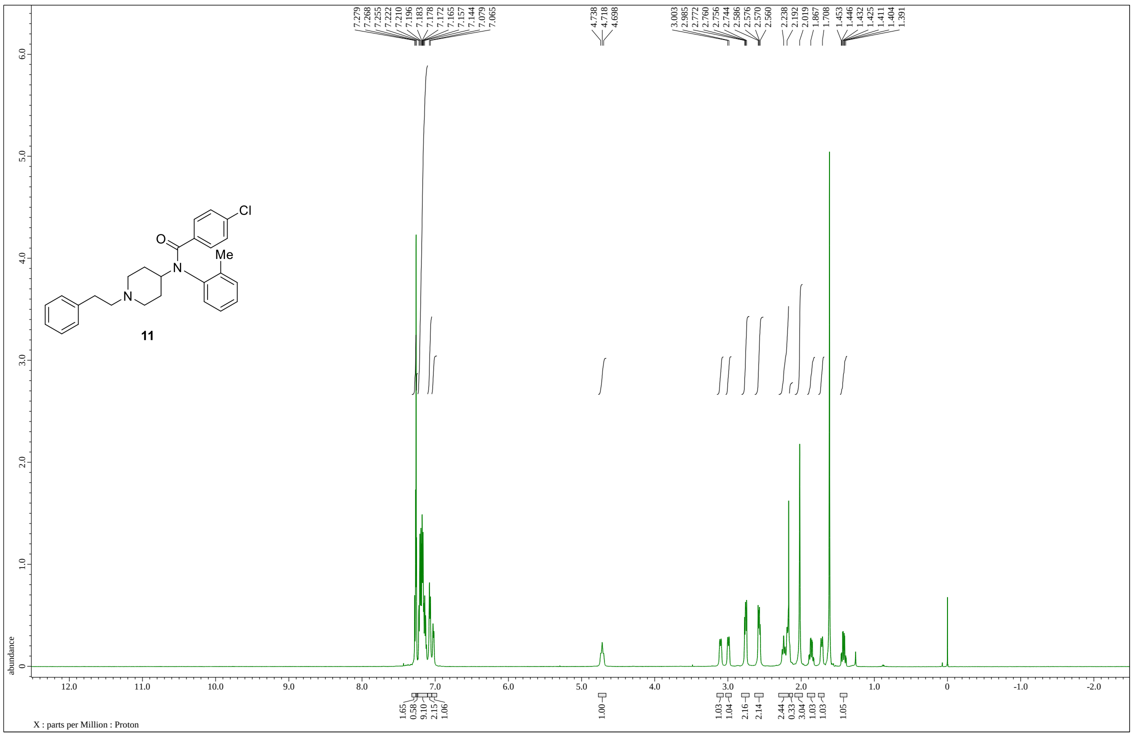

^{13}C NMR spectrum of compound **11** ($CDCl_3$)

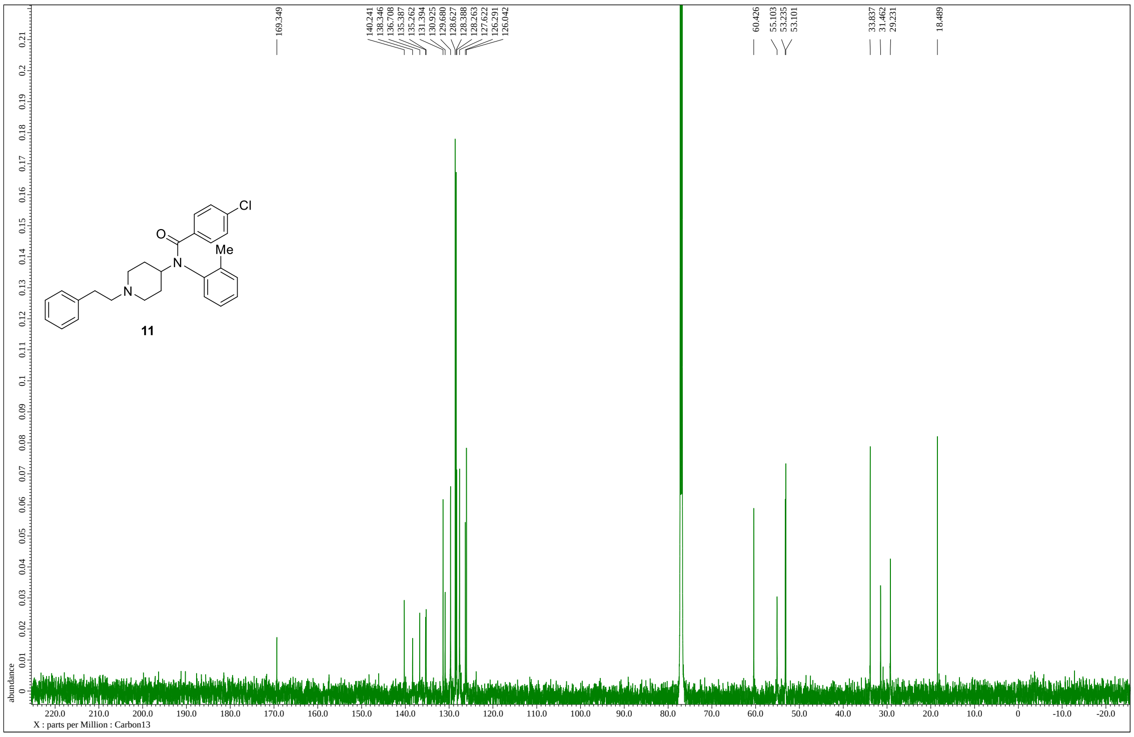

HRMS of compound **11**

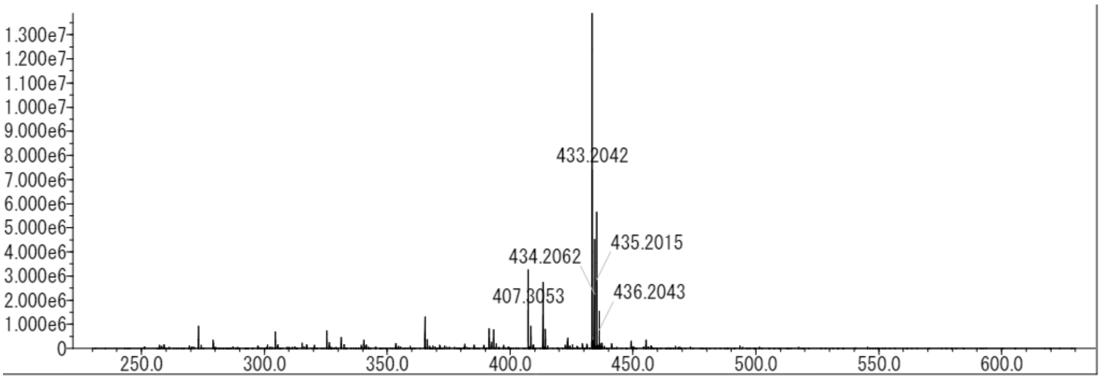

Formula [M+H]+	Theoretical *m/z*	Found *m/z*
$C_{27}H_{30}ClN_2O$	433.2041	433.2042

^{1}H NMR spectrum of compound **12** ($CDCl_3$)

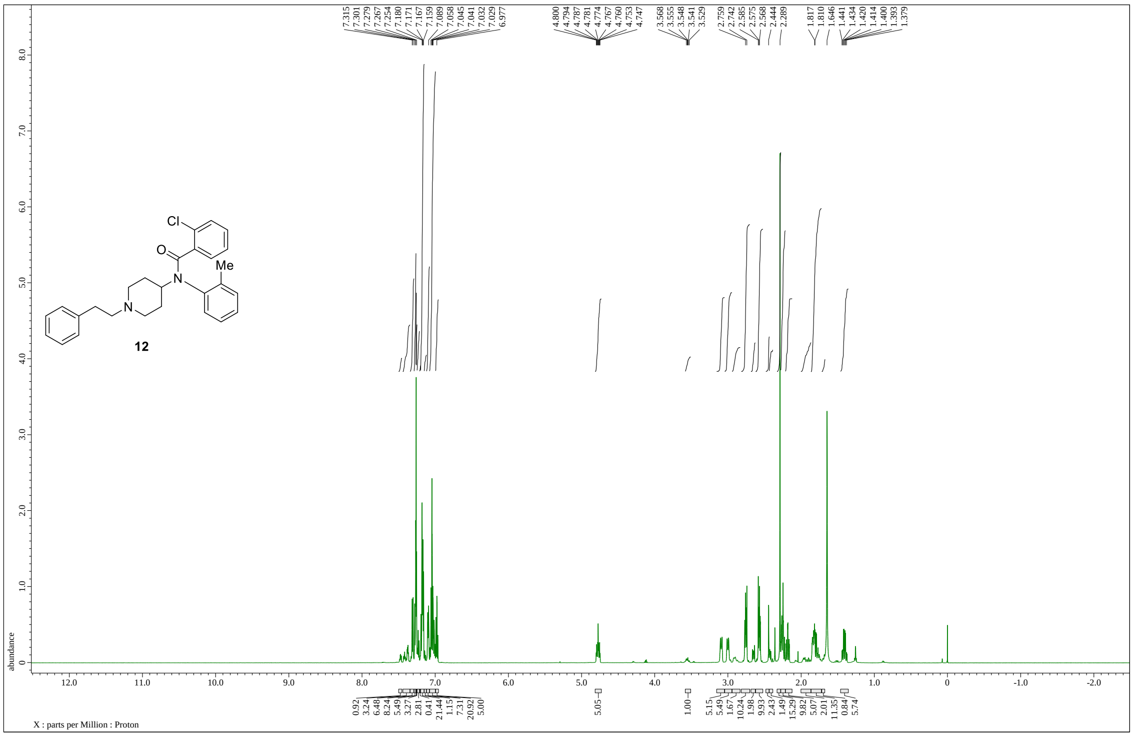

^{13}C NMR spectrum of compound **12** ($CDCl_3$)

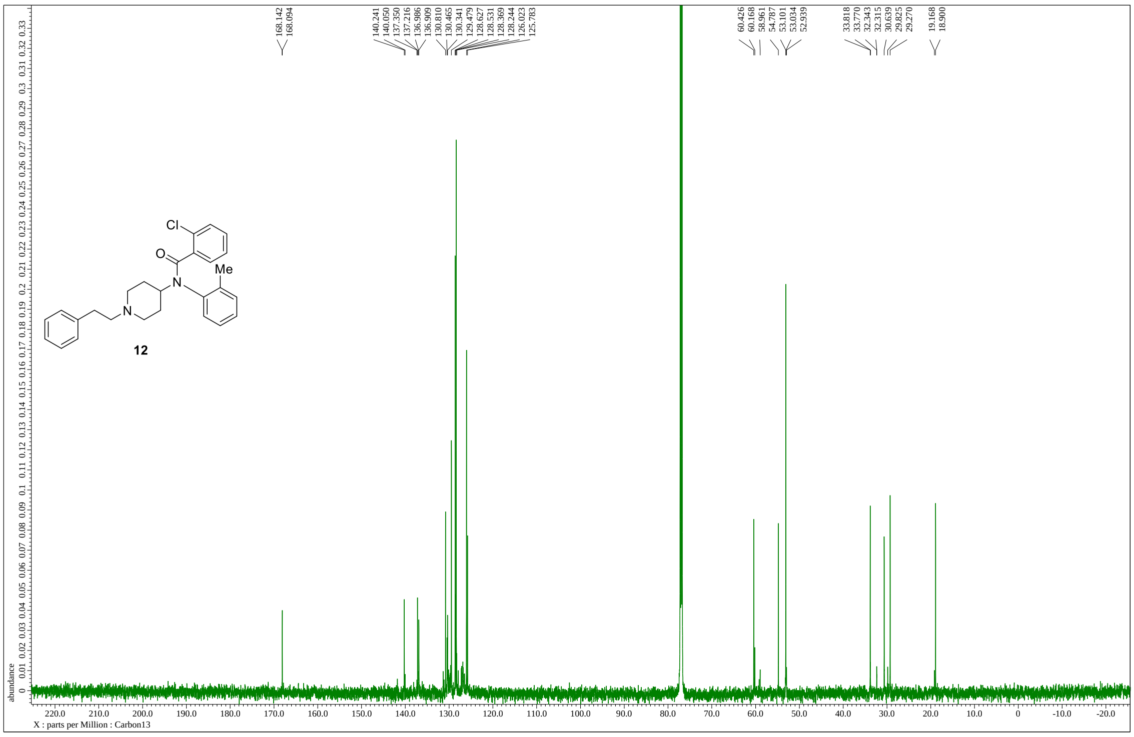

HRMS of compound **12**

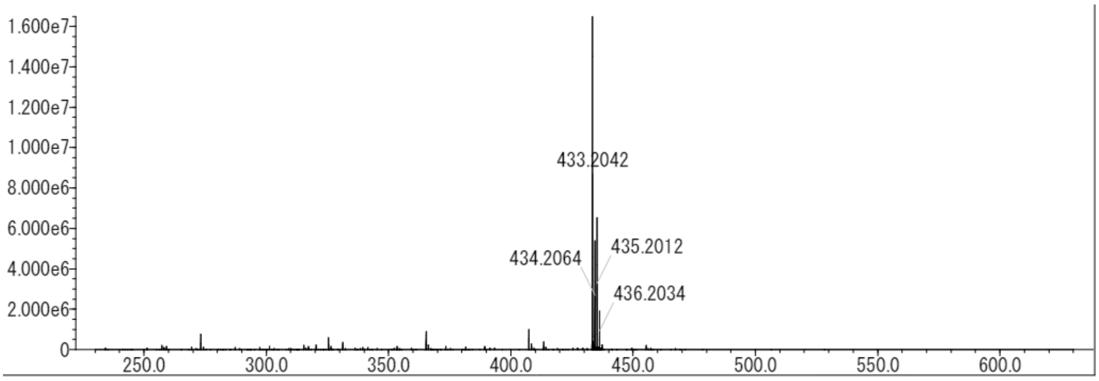

Formula $[M+H]^+$	Theoretical *m/z*	Found *m/z*
$C_{27}H_{30}ClN_2O$	433.2041	433.2042

^{1}H NMR spectrum of compound **13** ($CDCl_3$)

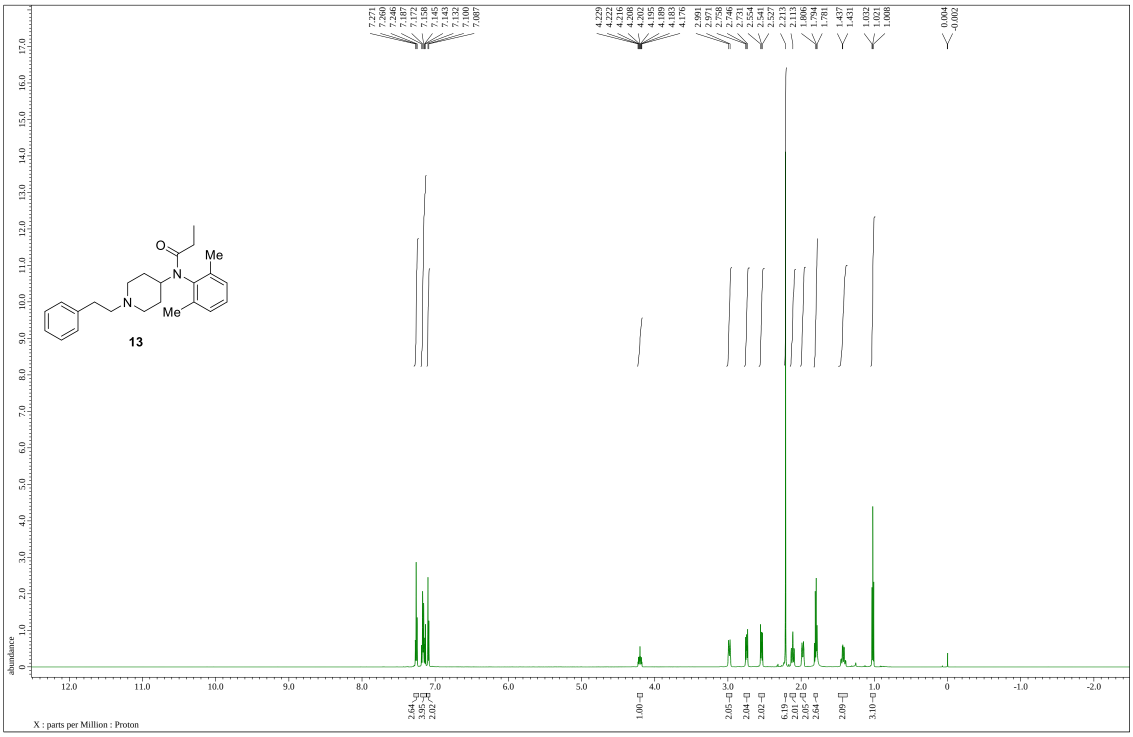

^{13}C NMR spectrum of compound **13** ($CDCl_3$)

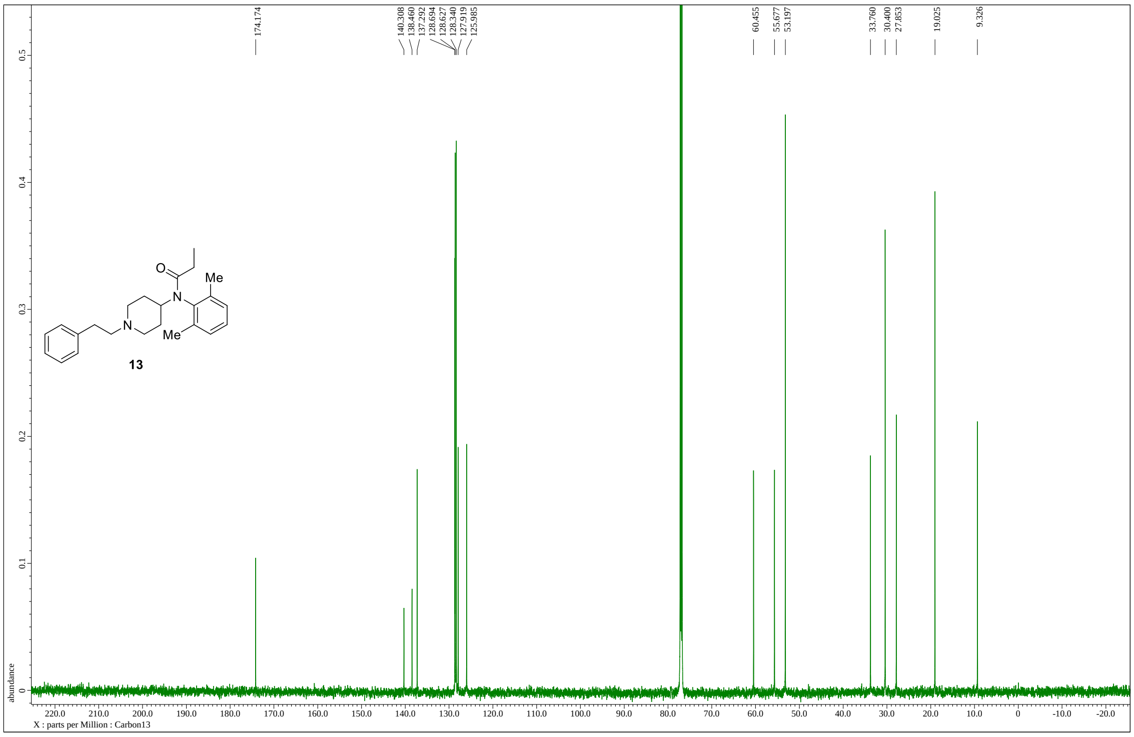

HRMS of compound **13**

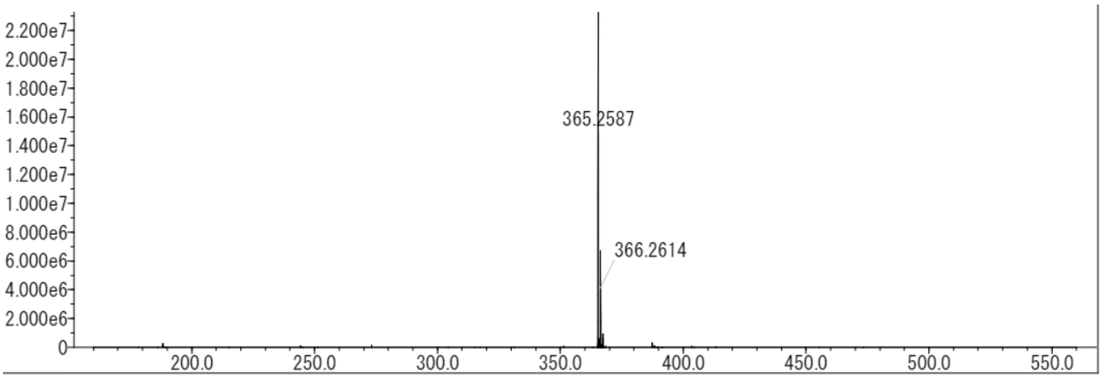

Formula [M+H]⁺	Theoretical *m/z*	Found *m/z*
$C_{24}H_{33}N_2O$	365.2587	365.2587

^{1}H NMR spectrum of compound **14** ($CDCl_3$)

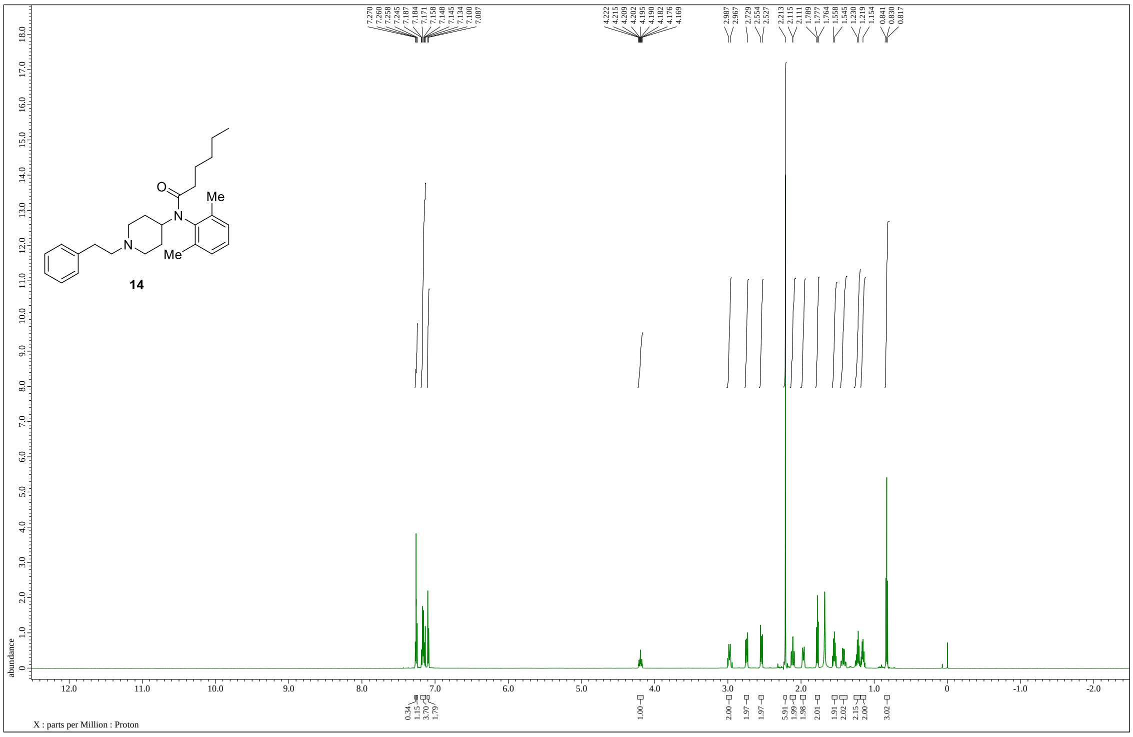

^{13}C NMR spectrum of compound **14** ($CDCl_3$)

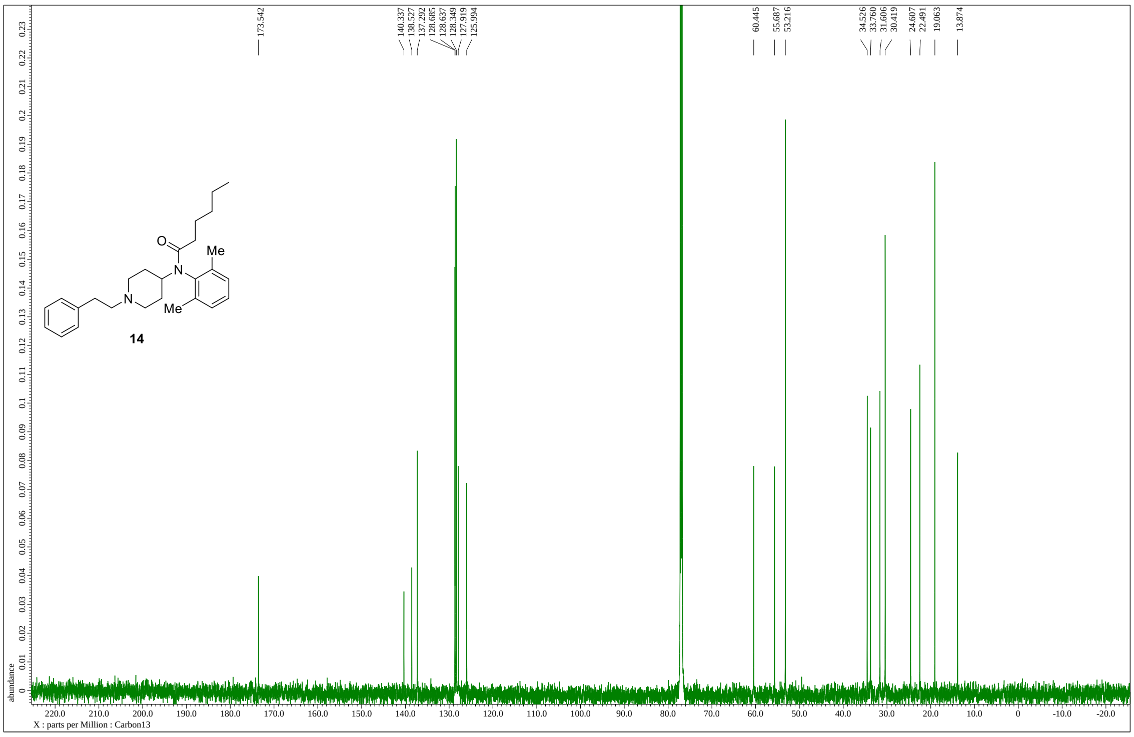

HRMS of compound **14**

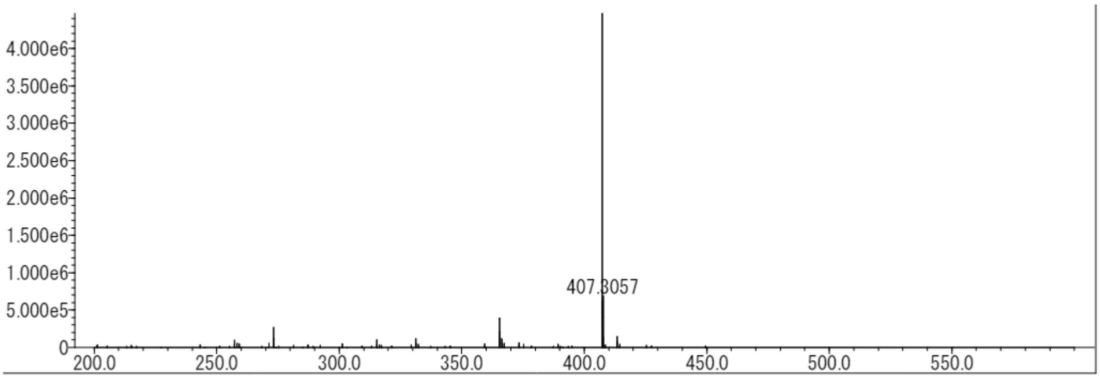

Formula [M+H]$^+$	Theoretical *m/z*	Found *m/z*
$C_{27}H_{39}N_2O$	407.3057	407.3057

^{1}H NMR spectrum of compound **15** ($CDCl_3$)

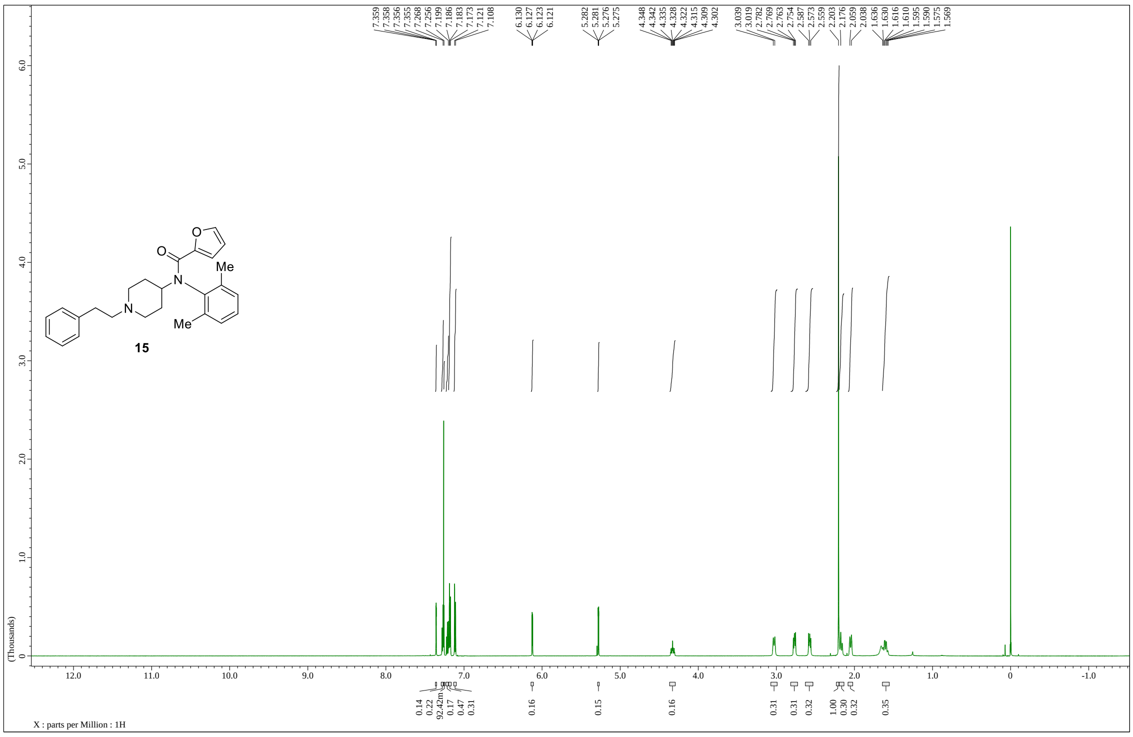

^{13}C NMR spectrum of compound **15** ($CDCl_3$)

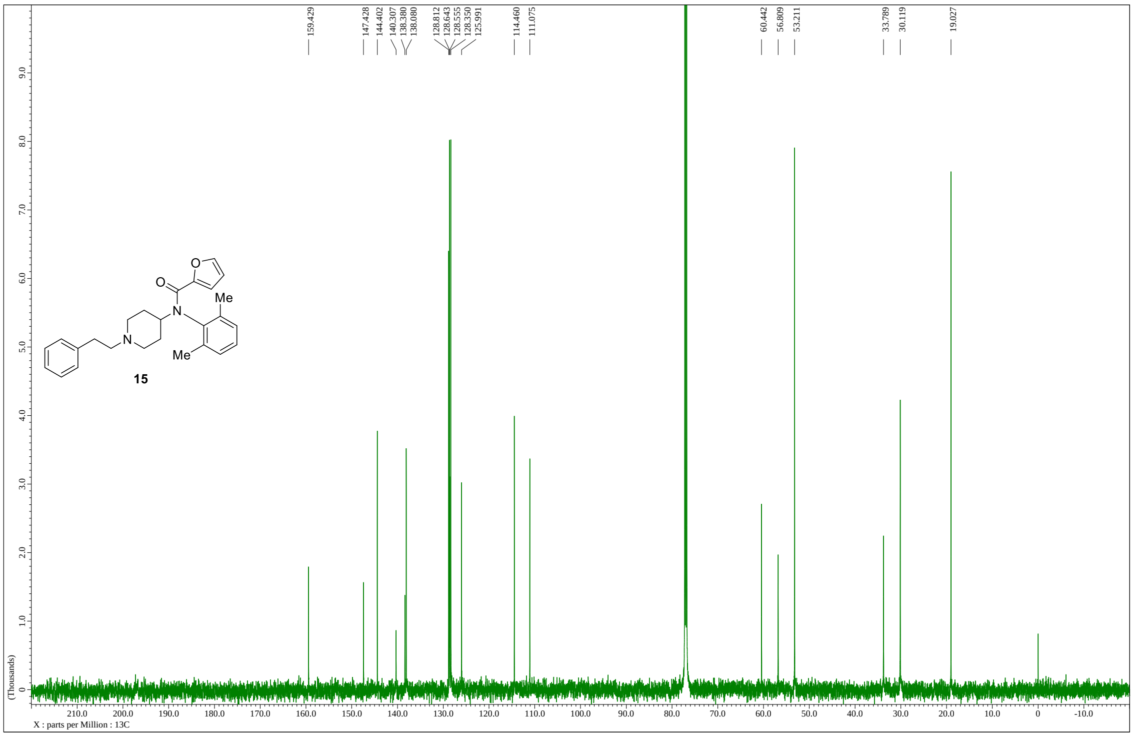

HRMS of compound **15**

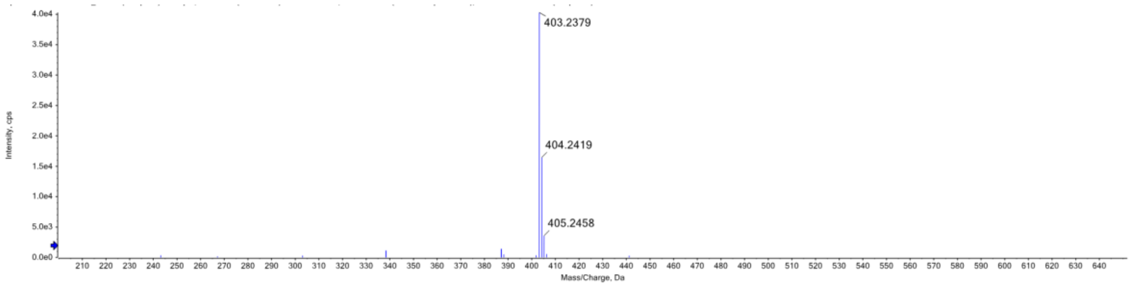

Formula $[M+H]^+$	Theoretical *m/z*	Found *m/z*
$C_{26}H_{31}N_2O_2$	403.2380	403.2379

^1H NMR spectrum of compound **16** ($CDCl_3$)

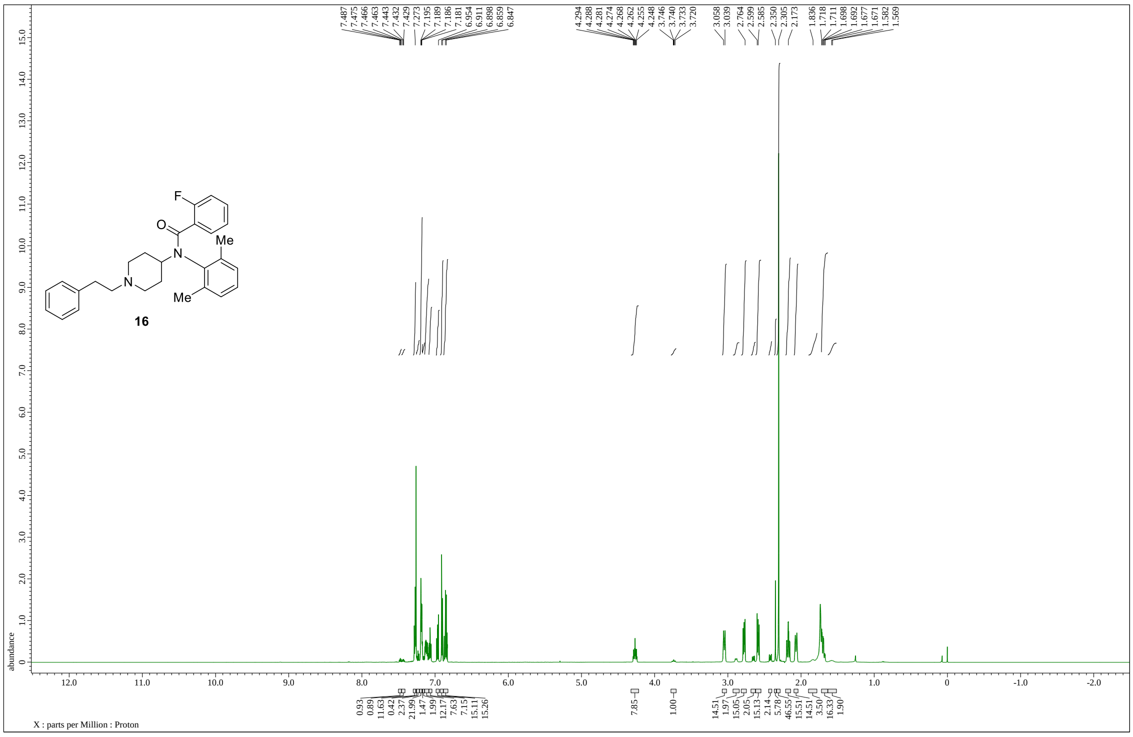

^{13}C NMR spectrum of compound **16** ($CDCl_3$)

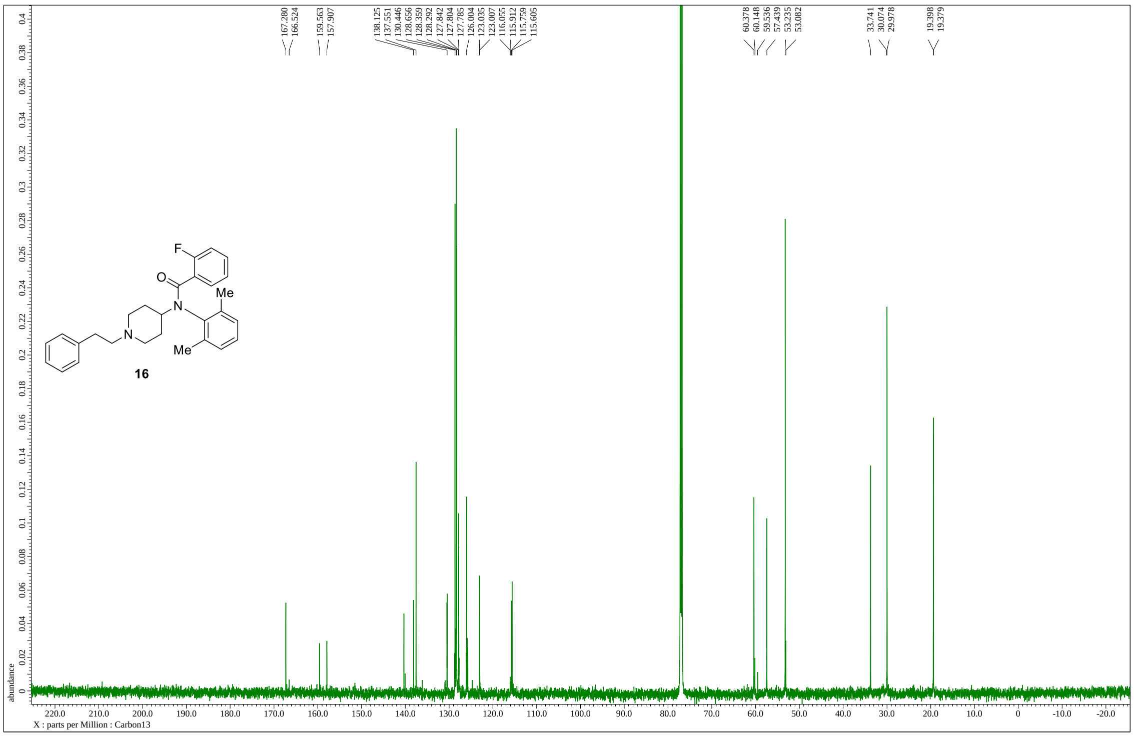

HRMS of compound **16**

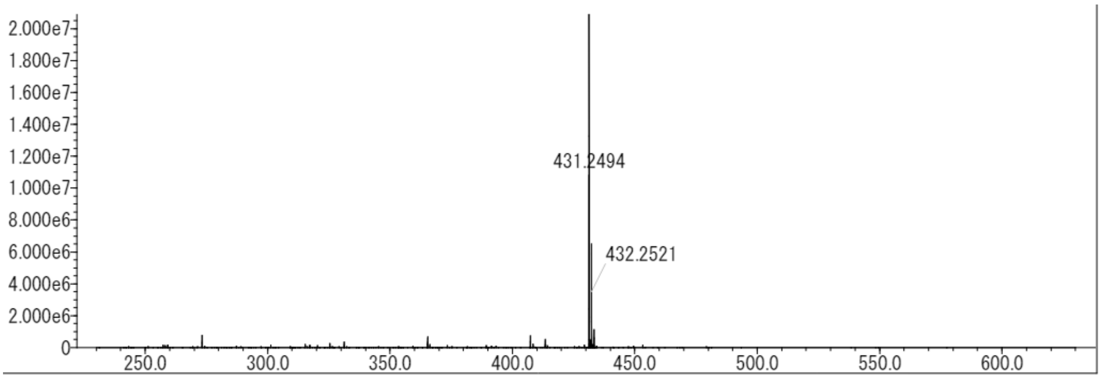

Formula $[M+H]^+$	Theoretical *m/z*	Found *m/z*
$C_{28}H_{32}FN_2O$	431.2493	431.2494

^{1}H NMR spectrum of compound **17** ($CDCl_3$)

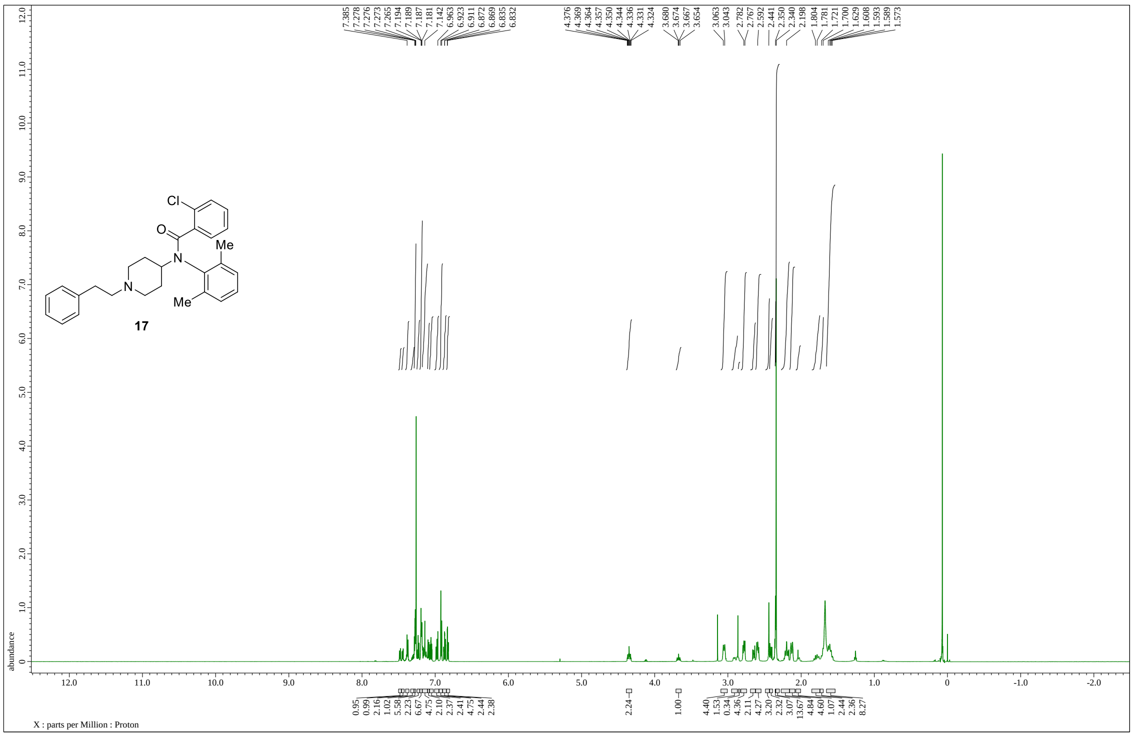

^{13}C NMR spectrum of compound **17** ($CDCl_3$)

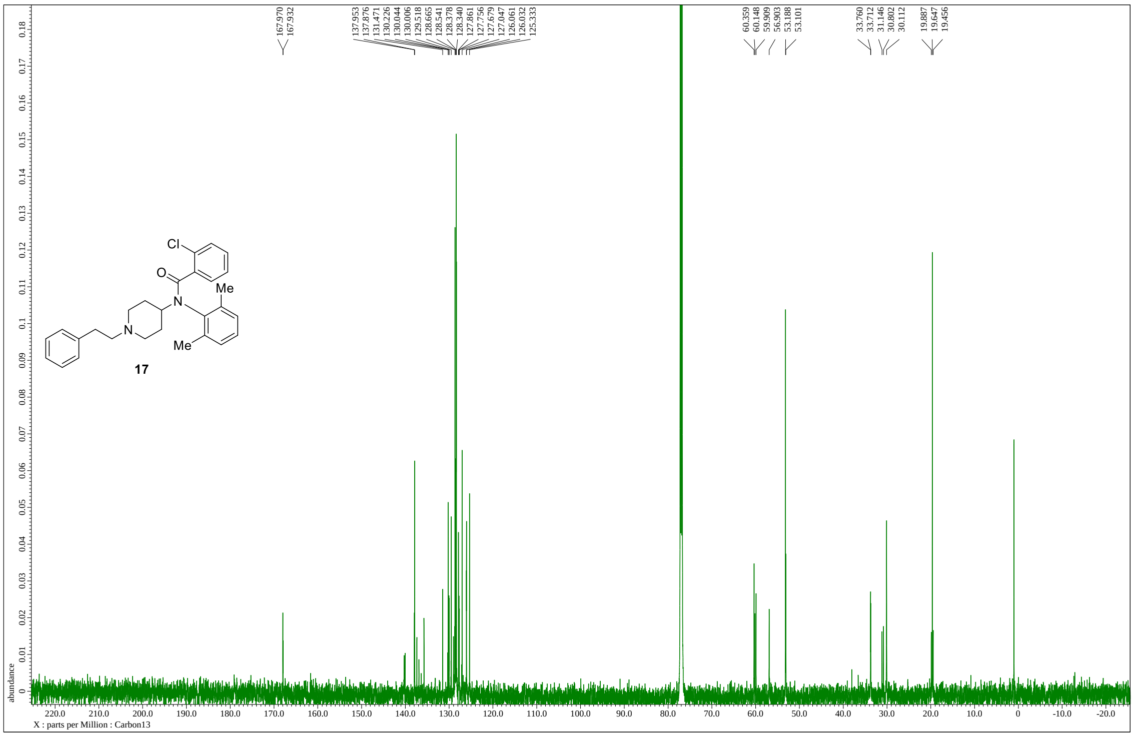

NOESY spectrum of compound **17** ($CDCl_3$)

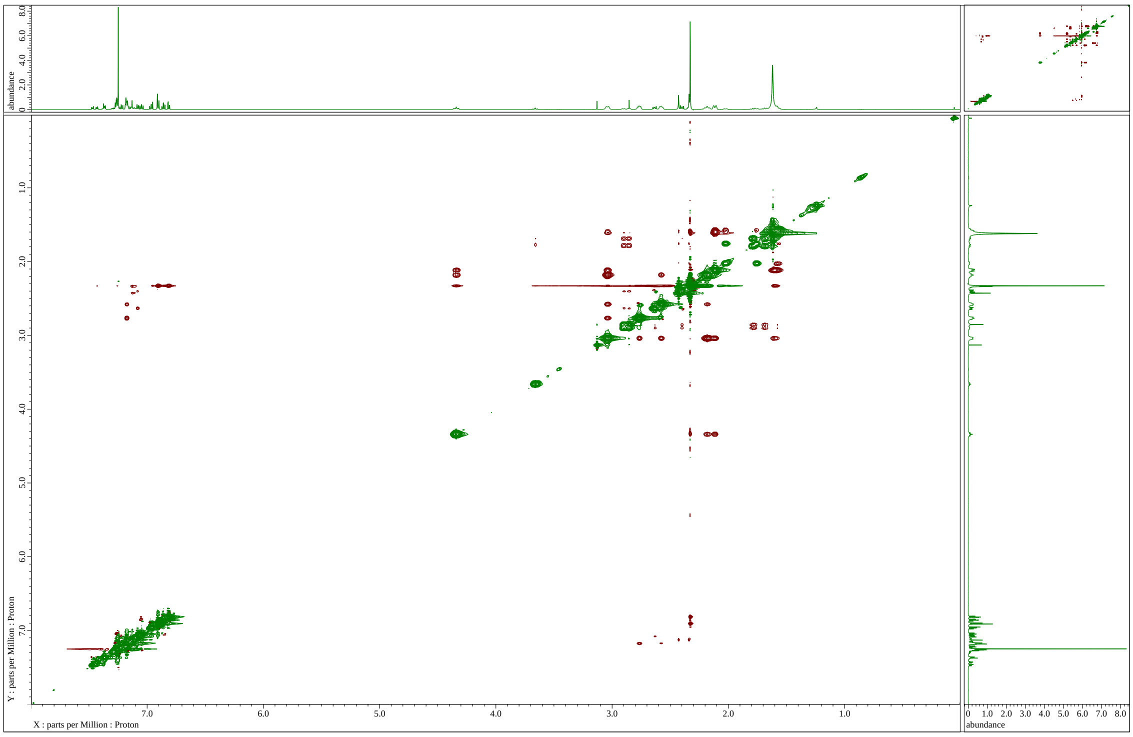

HRMS of compound **17**

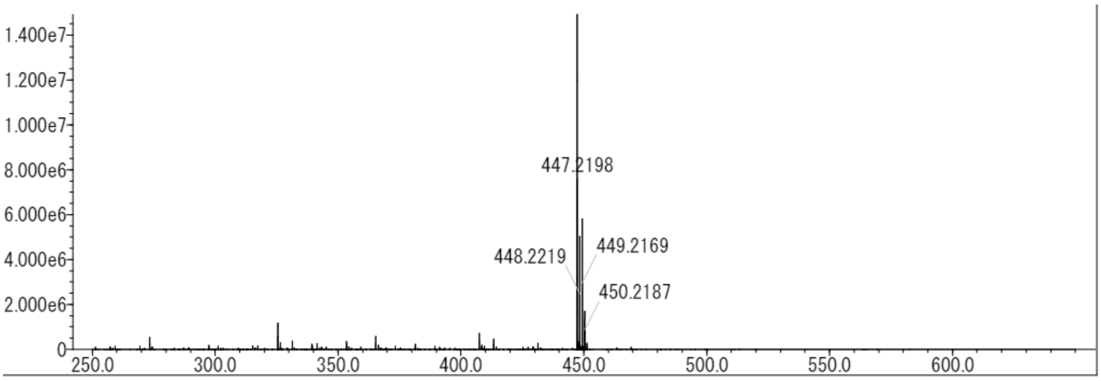

Formula $[M+H]^+$	Theoretical *m/z*	Found *m/z*
$C_{28}H_{32}ClN_2O$	447.2198	447.2198

^{1}H NMR spectrum of compound **18** ($CDCl_3$)

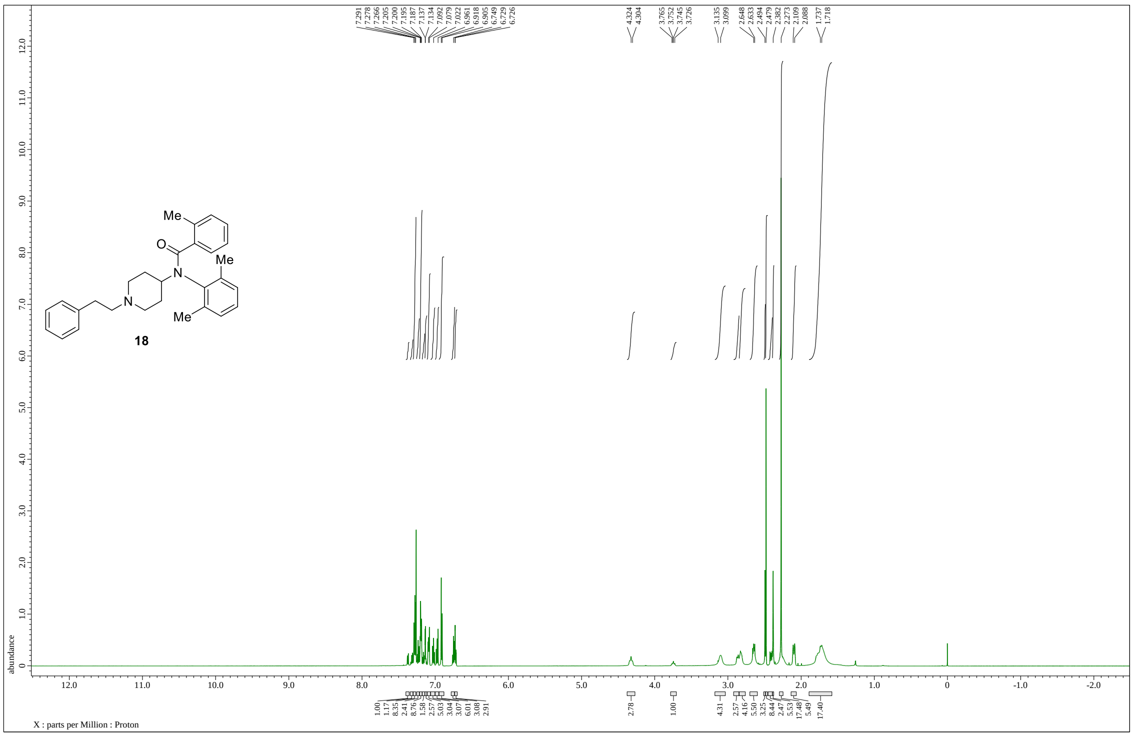

^{13}C NMR spectrum of compound **18** ($CDCl_3$)

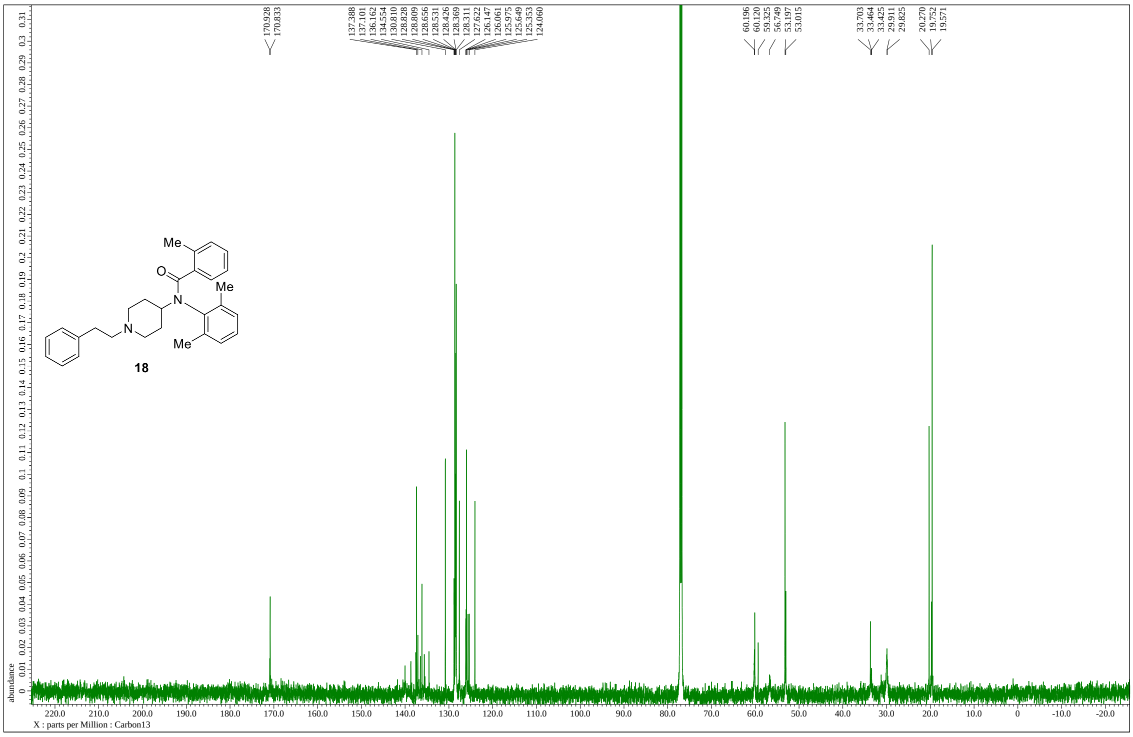

HRMS of compound **18**

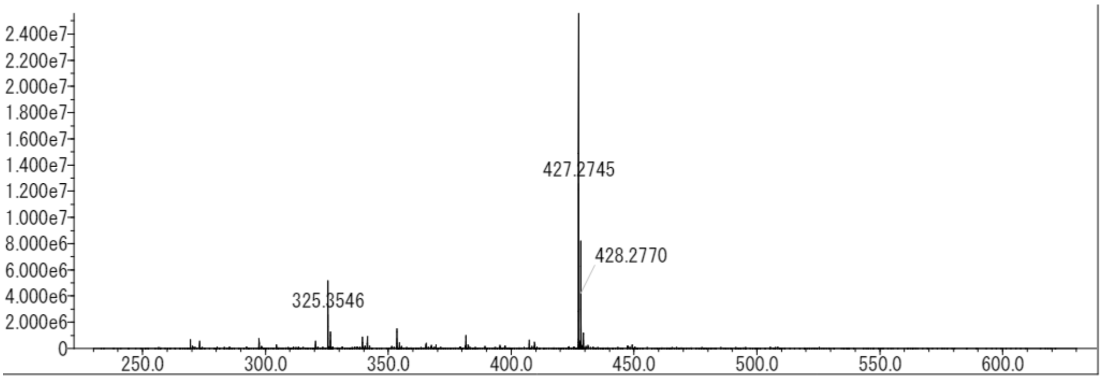

Formula [M+H]⁺	Theoretical *m/z*	Found *m/z*
$C_{29}H_{35}N_2O$	427.2744	427.2745

^{1}H NMR spectrum of compound **19** ($CDCl_3$)

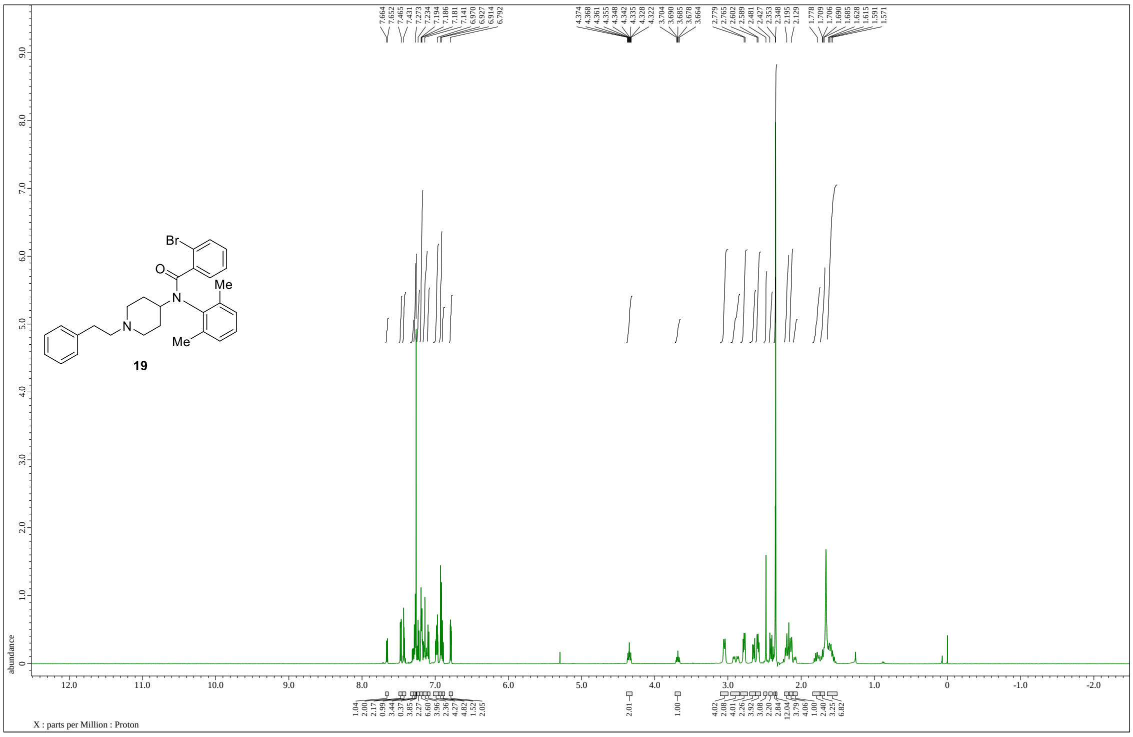

^{13}C NMR spectrum of compound **19** ($CDCl_3$)

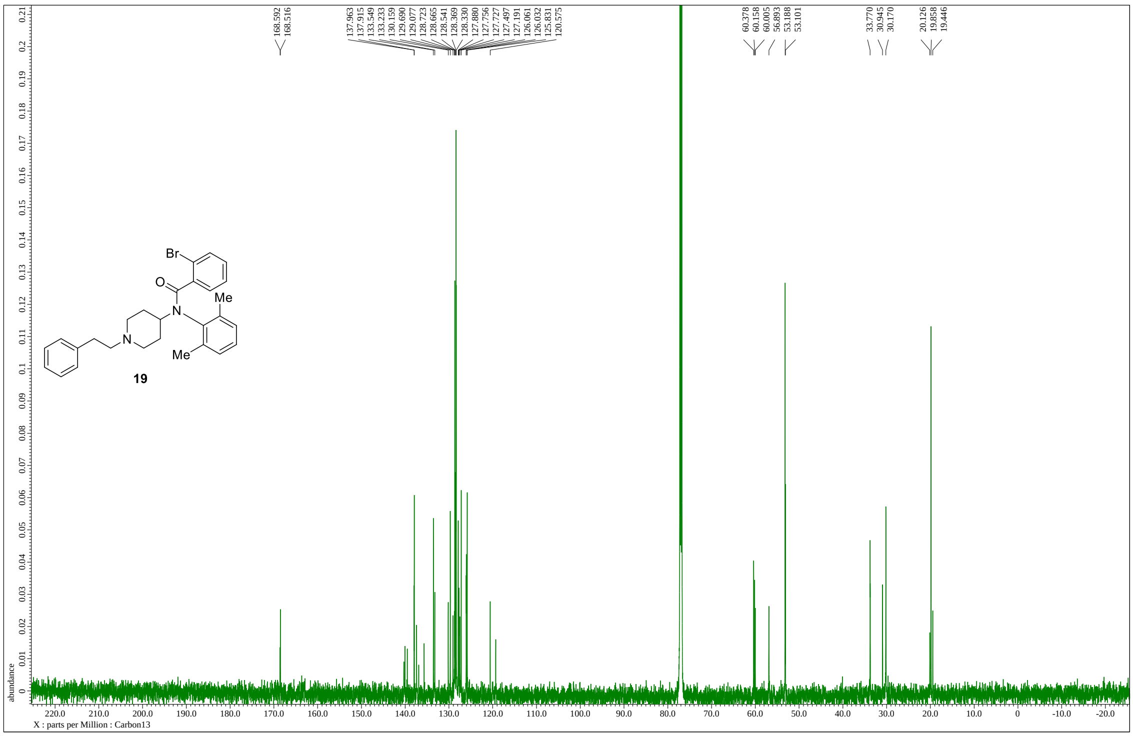

HRMS of compound **19**

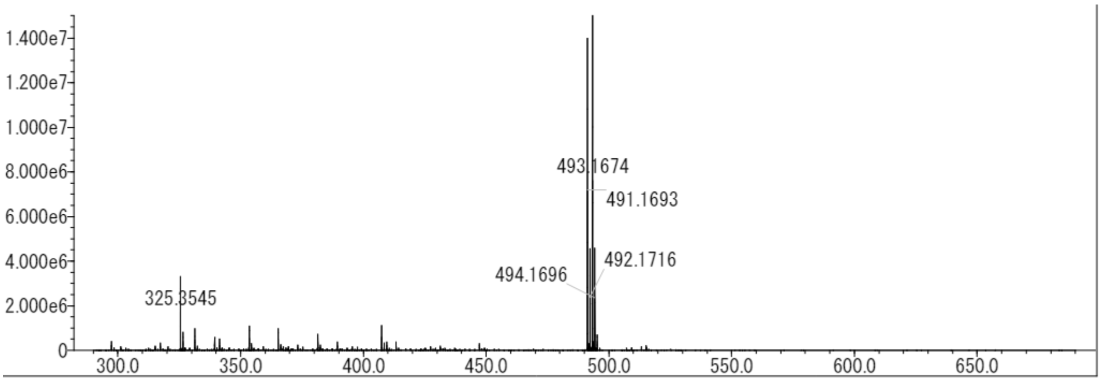

Formula [M+H]+	Theoretical *m/z*	Found *m/z*
$C_{28}H_{32}BrN_2O$	491.1693	491.1693

^{1}H NMR spectrum of compound **20** ($CDCl_3$)

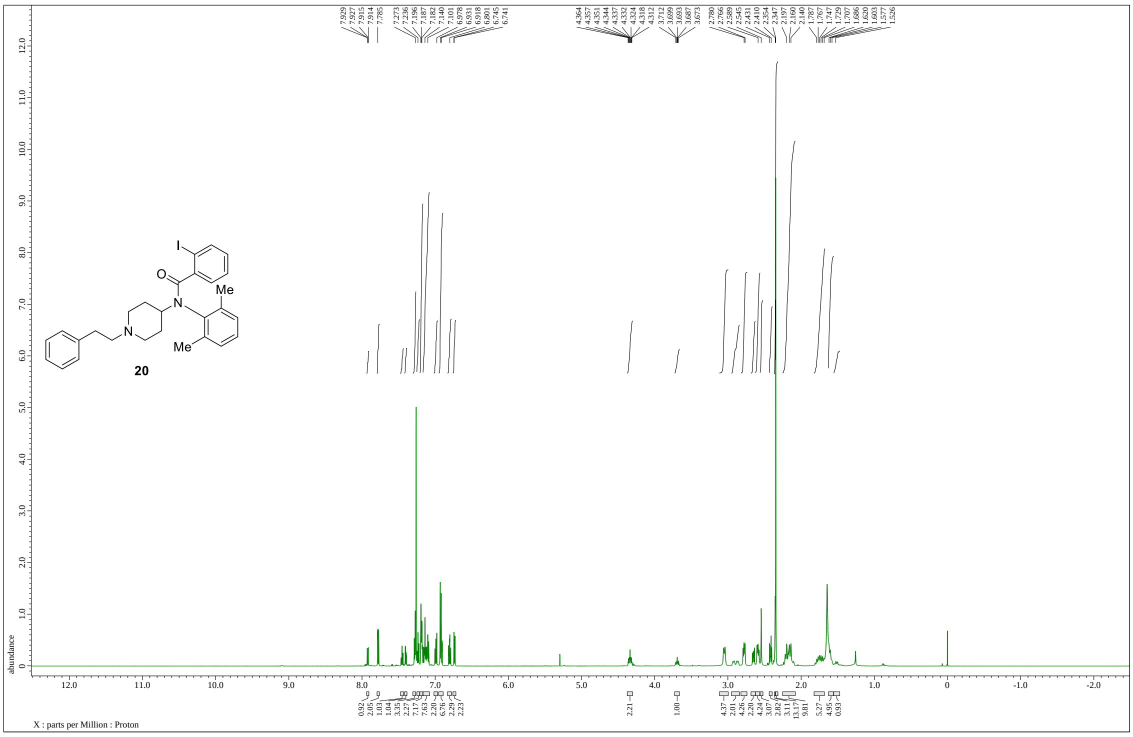

^{13}C NMR spectrum of compound **20** ($CDCl_3$)

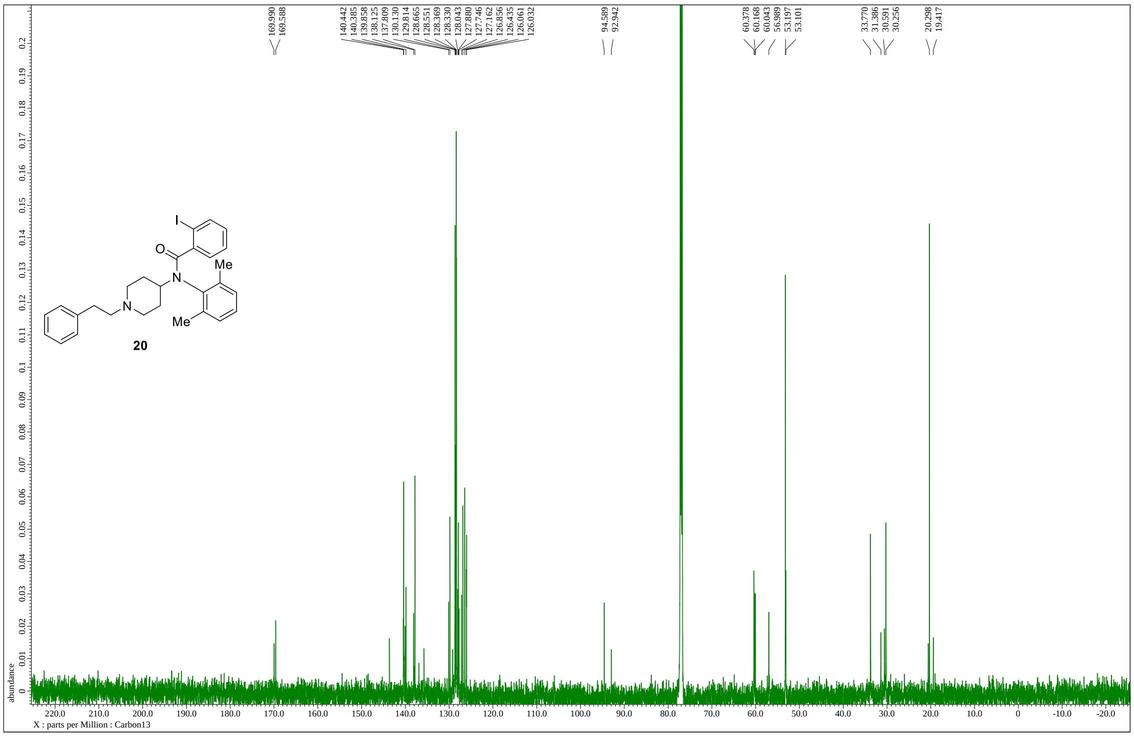

HRMS of compound **20**

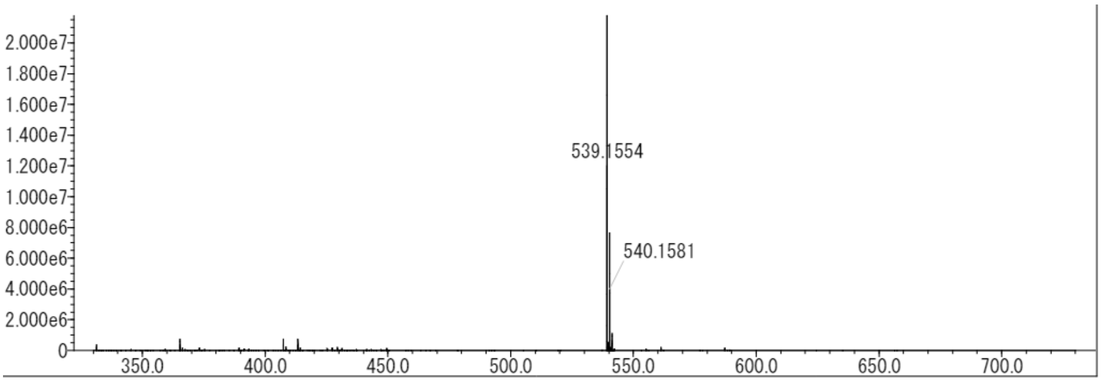

Formula [M+H]+	Theoretical *m/z*	Found *m/z*
$C_{28}H_{32}IN_2O$	539.1554	539.1554

^{1}H NMR spectrum of compound **21** ($CDCl_3$)

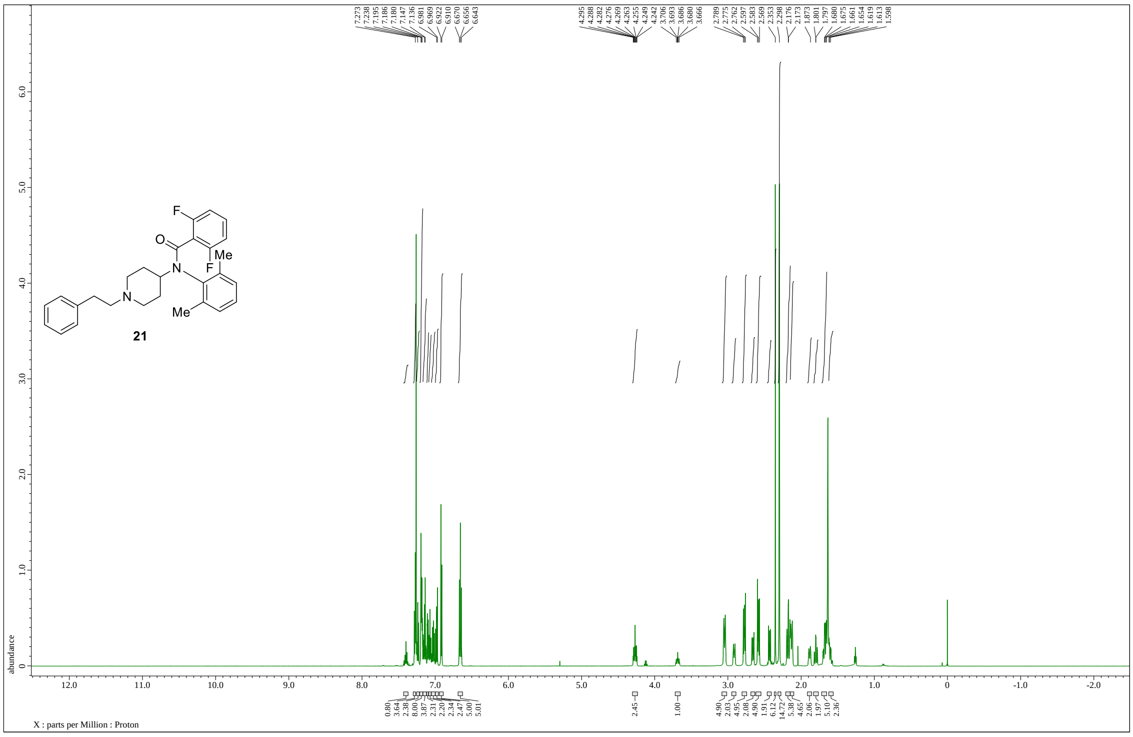

^{13}C NMR spectrum of compound **21** ($CDCl_3$)

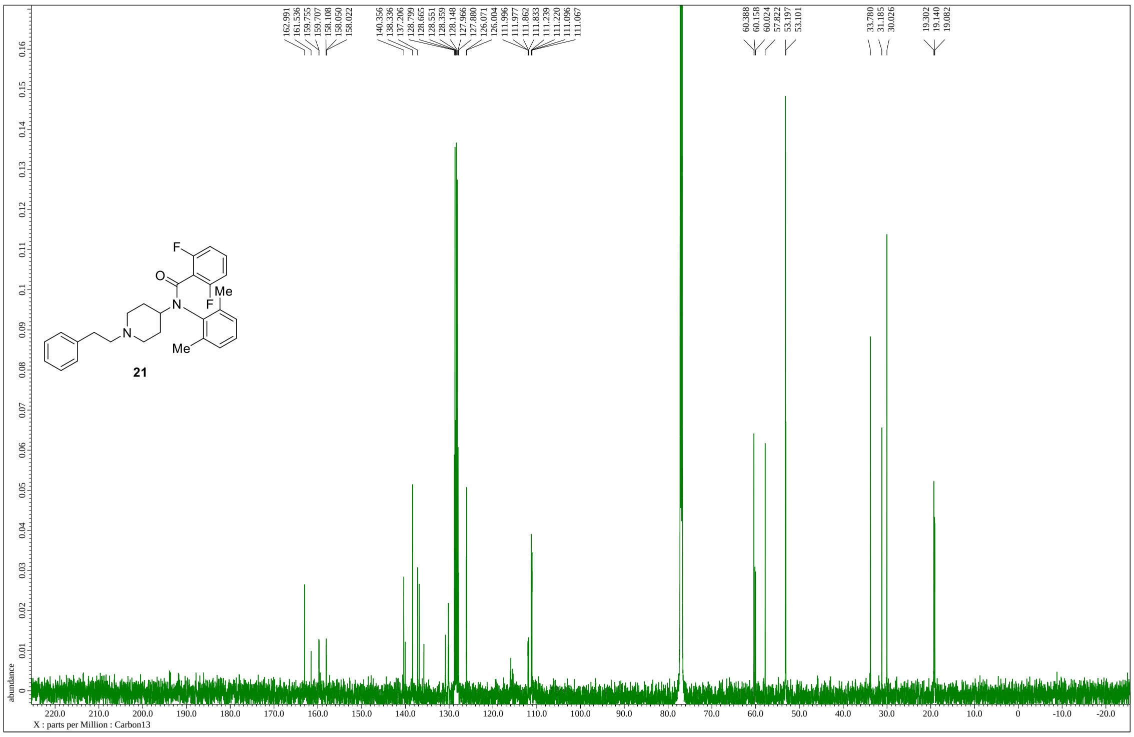

HRMS of compound **21**

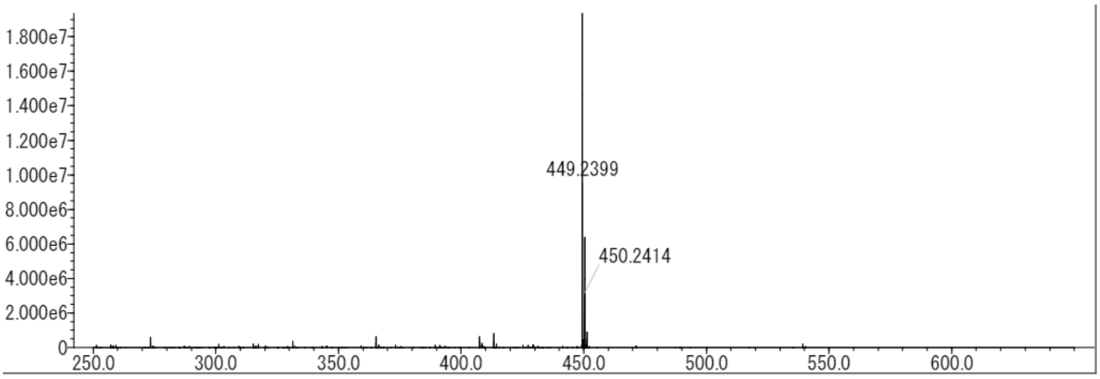

Formula [M+H]+	Theoretical *m/z*	Found *m/z*
$C_{28}H_{31}F_2N_2O$	449.2399	449.2399

^{1}H NMR spectrum of compound **22** ($CDCl_3$)

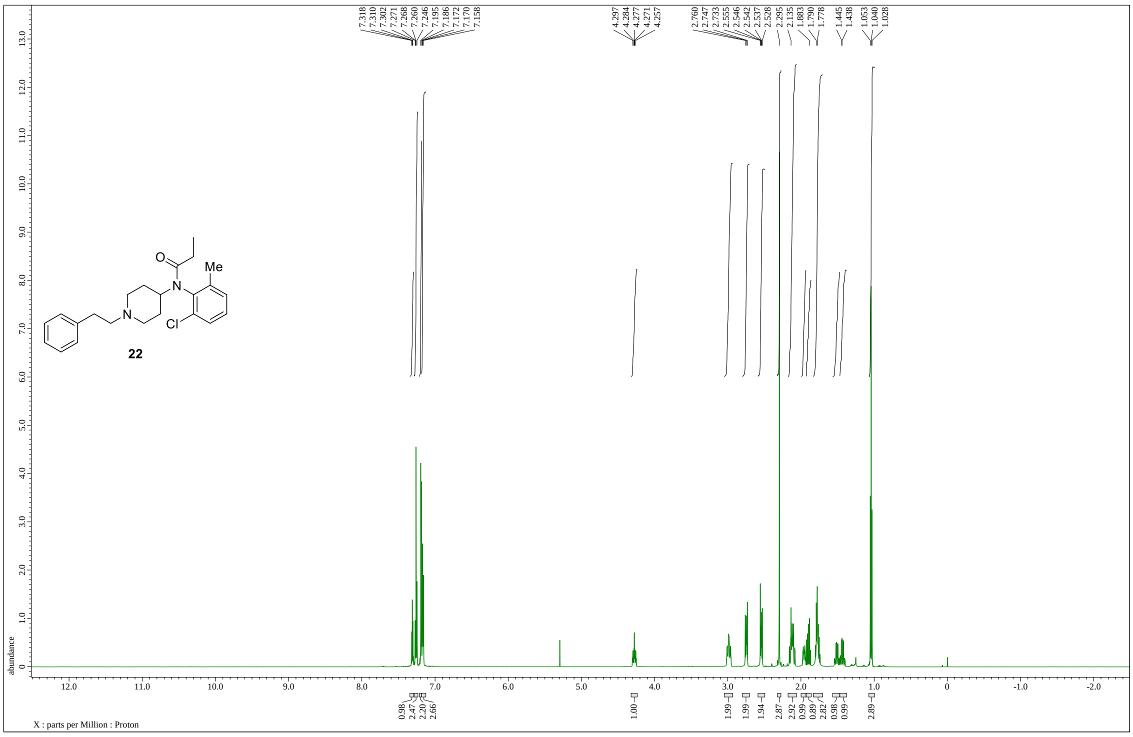

^{13}C NMR spectrum of compound **22**($CDCl_3$)

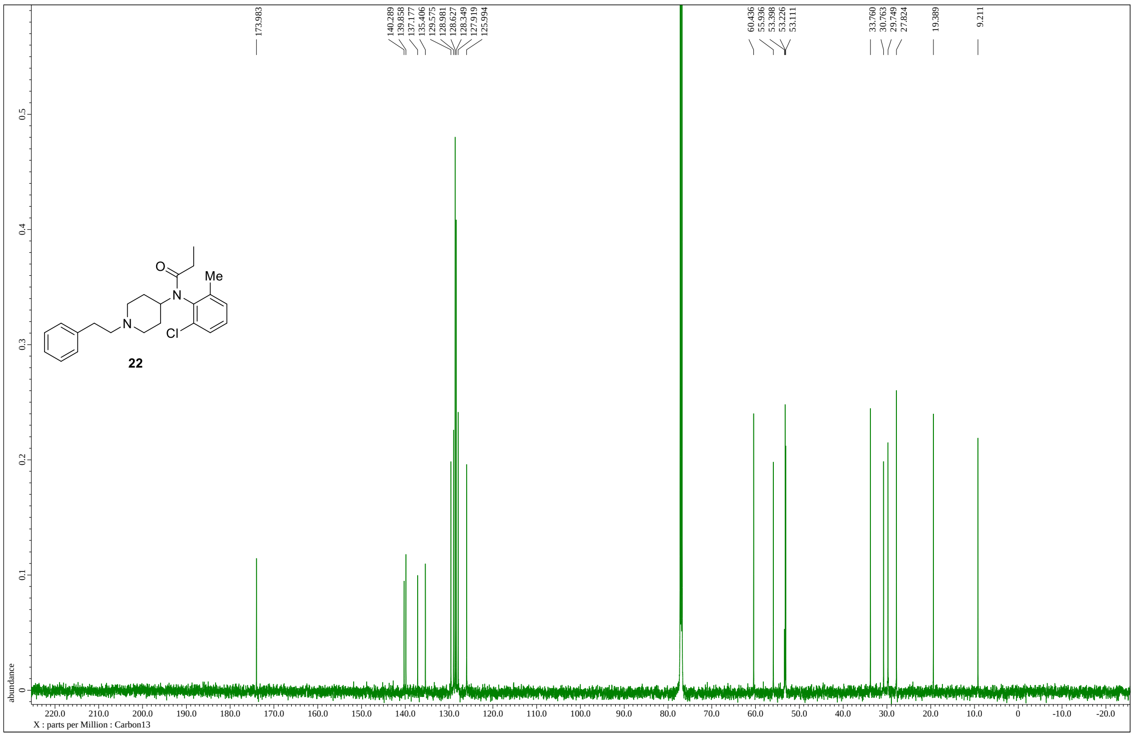

HRMS of compound **22**

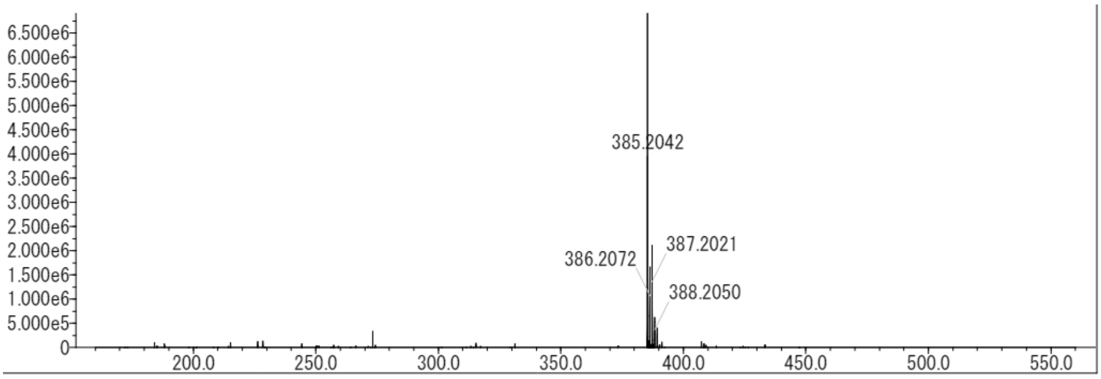

Formula $[M+H]^+$	Theoretical *m/z*	Found *m/z*
$C_{23}H_{30}ClN_2O$	385.2041	385.2042

^1H NMR spectrum of compound **23** ($CDCl_3$)

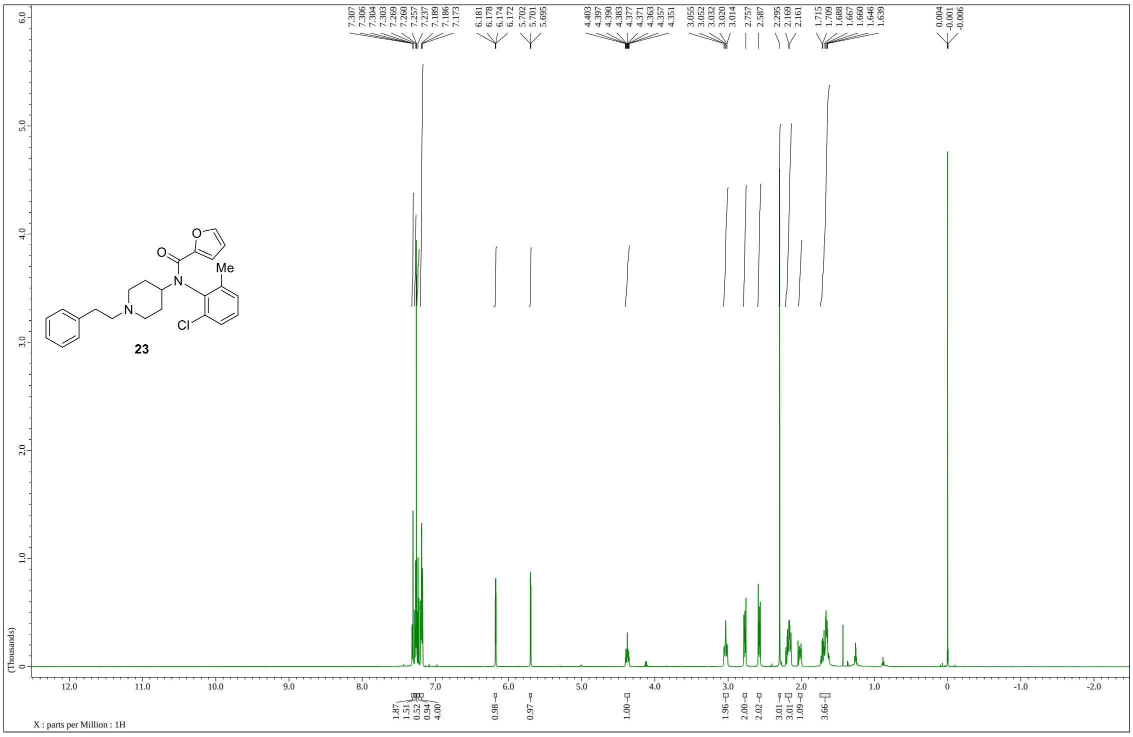

^{13}C NMR spectrum of compound **23**($CDCl_3$)

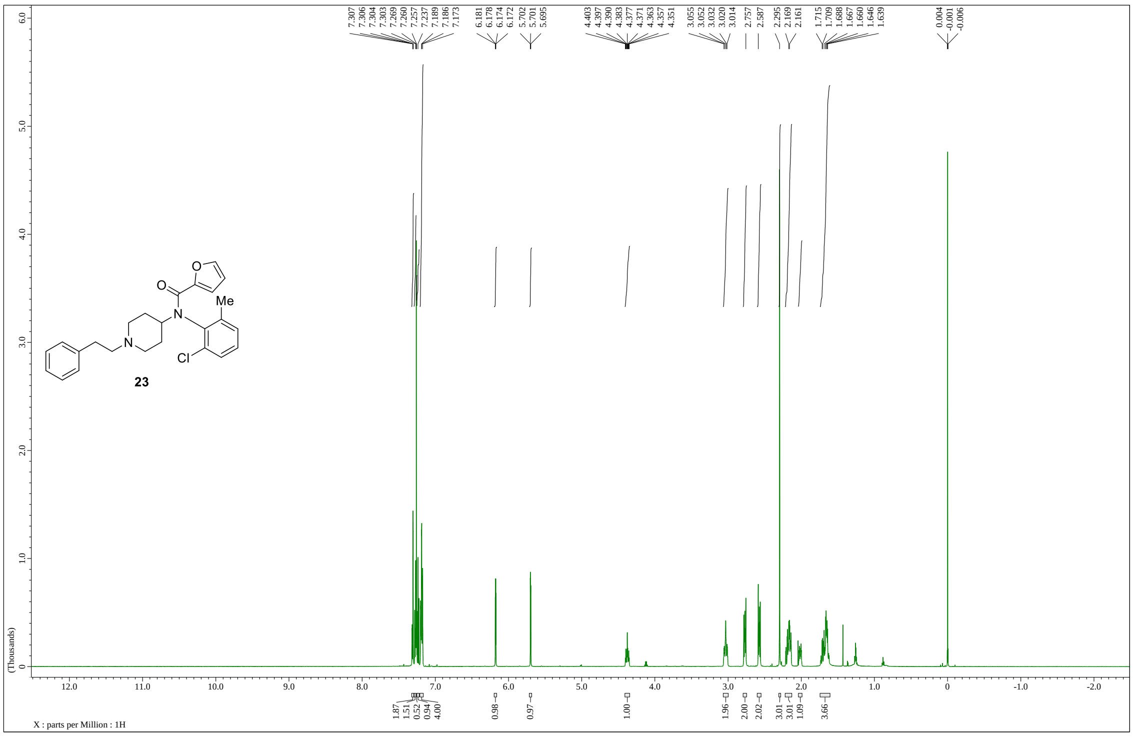

HRMS of compound **23**

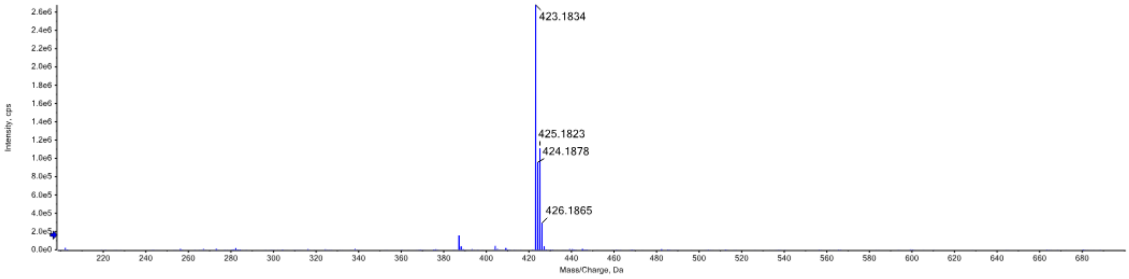

Formula $[M+H]^+$	Theoretical *m/z*	Found *m/z*
$C_{25}H_{28}ClN_2O_2$	423.1834	423.1834

^{1}H NMR spectrum of compound **24** ($CDCl_3$)

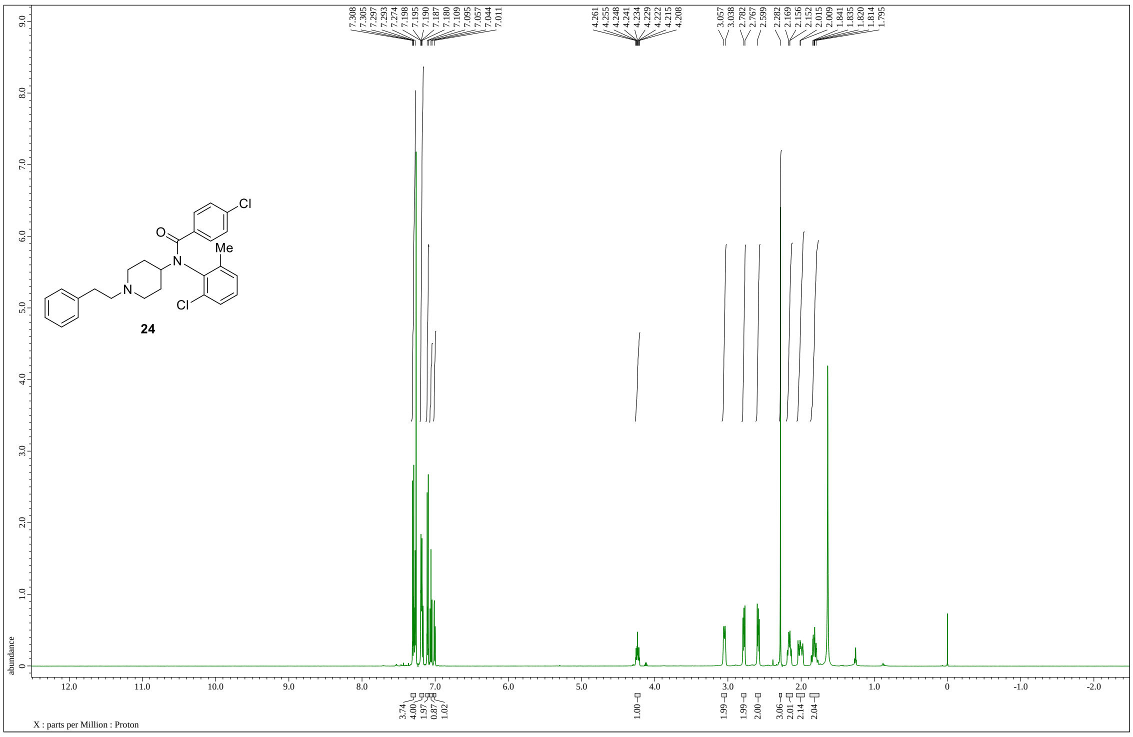

^{13}C NMR spectrum of compound **24** ($CDCl_3$)

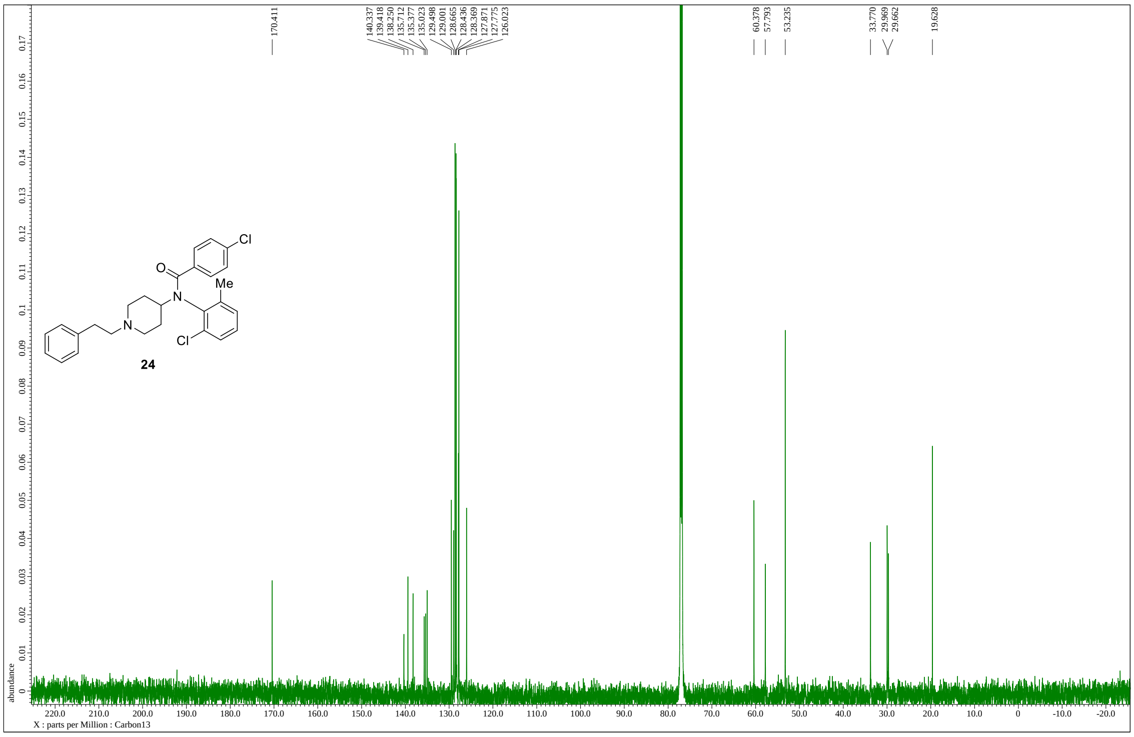

HRMS of compound **24**

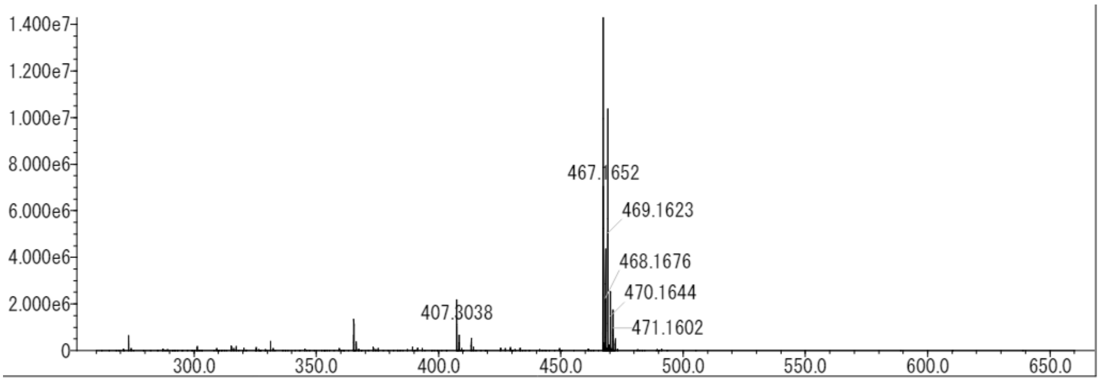

Formula [M+H]+	Theoretical *m/z*	Found *m/z*
$C_{27}H_{29}Cl_2N_2O$	467.1651	467.1652

^{1}H NMR spectrum of compound **25** ($CDCl_3$)

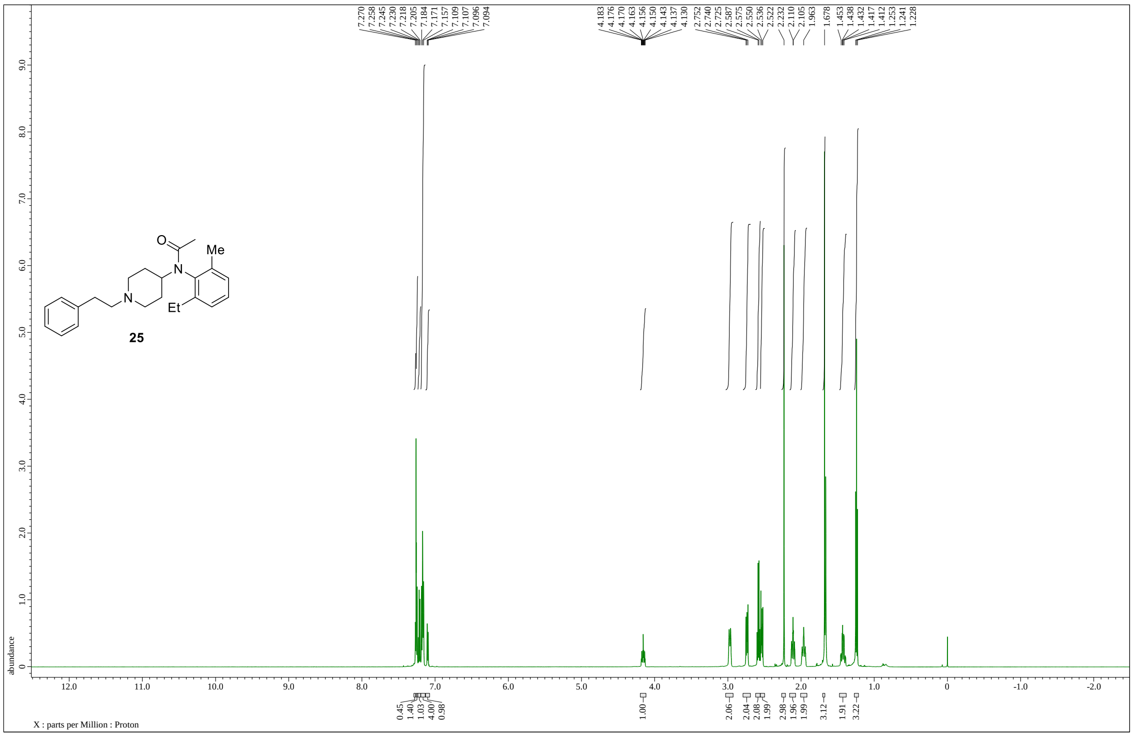

^{13}C NMR spectrum of compound **25** ($CDCl_3$)

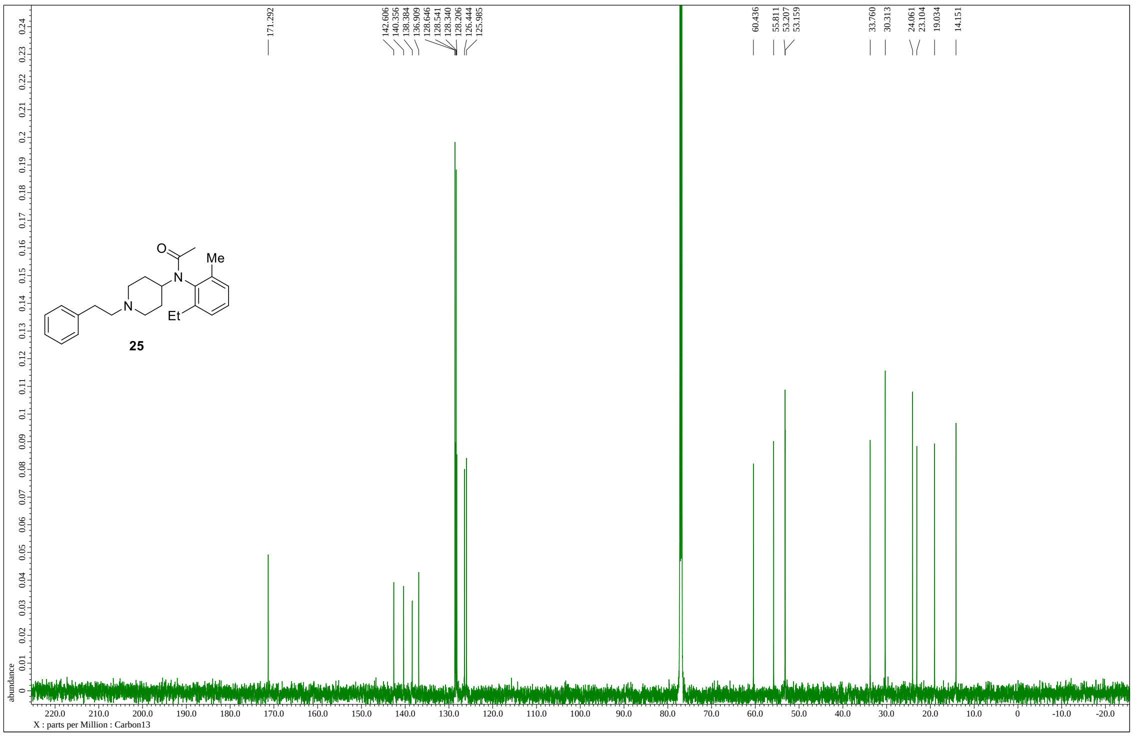

HRMS of compound **25**

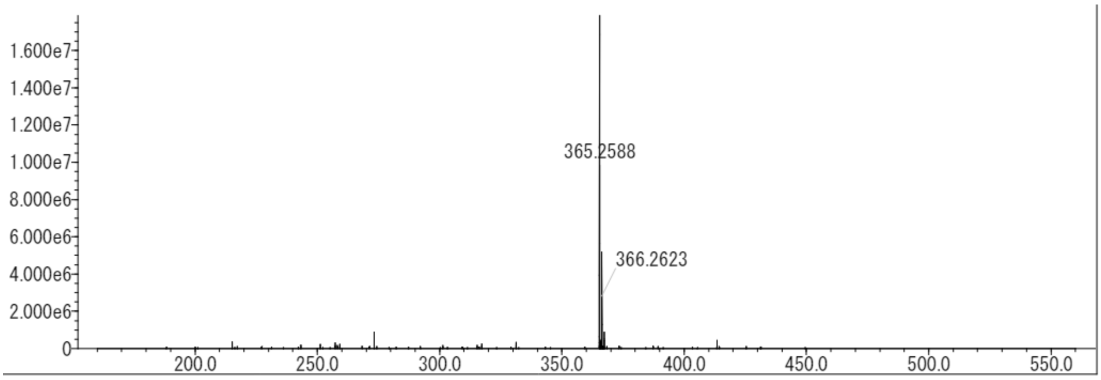

Formula [M+H]+	Theoretical *m/z*	Found *m/z*
$C_{24}H_{33}N_2O$	365.2587	365.2588

^{1}H NMR spectrum of compound **26** ($CDCl_3$)

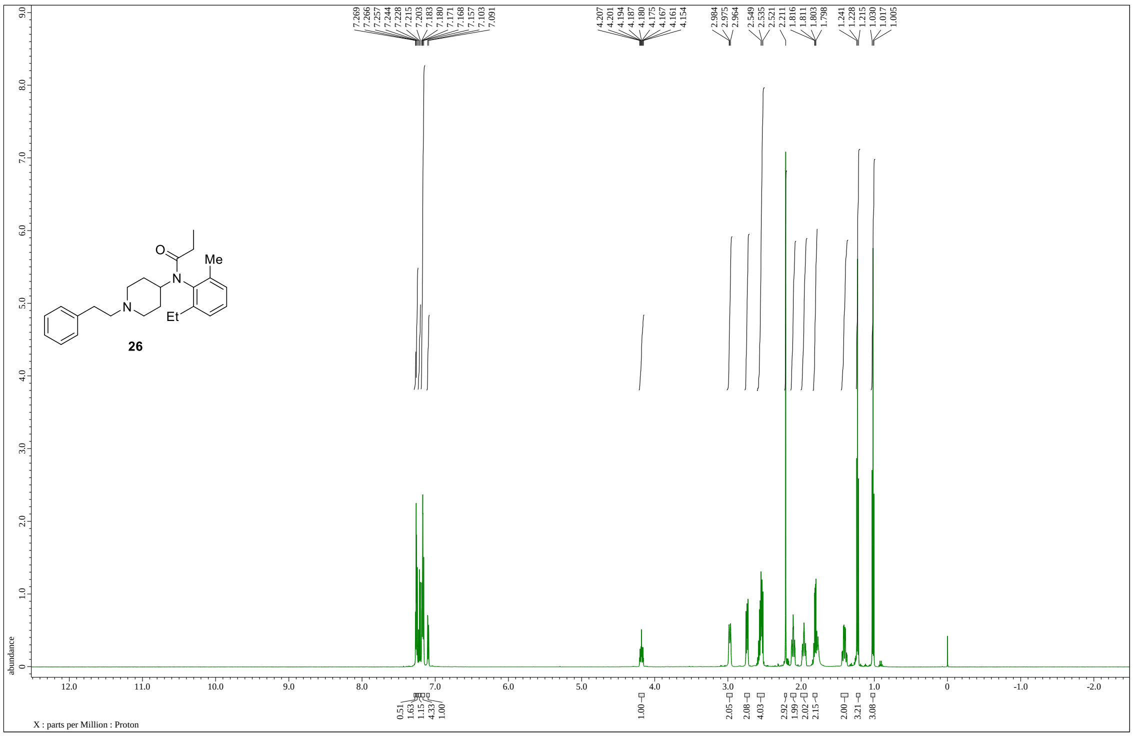

^{13}C NMR spectrum of compound **26** ($CDCl_3$)

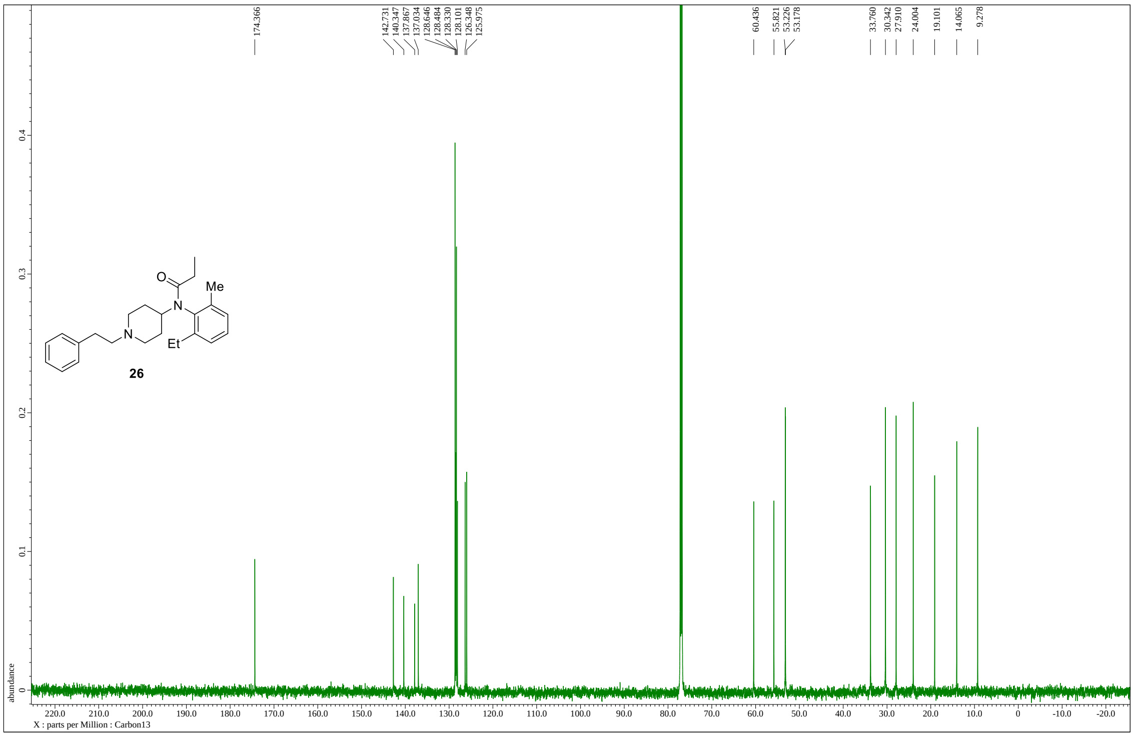

HRMS of compound **26**

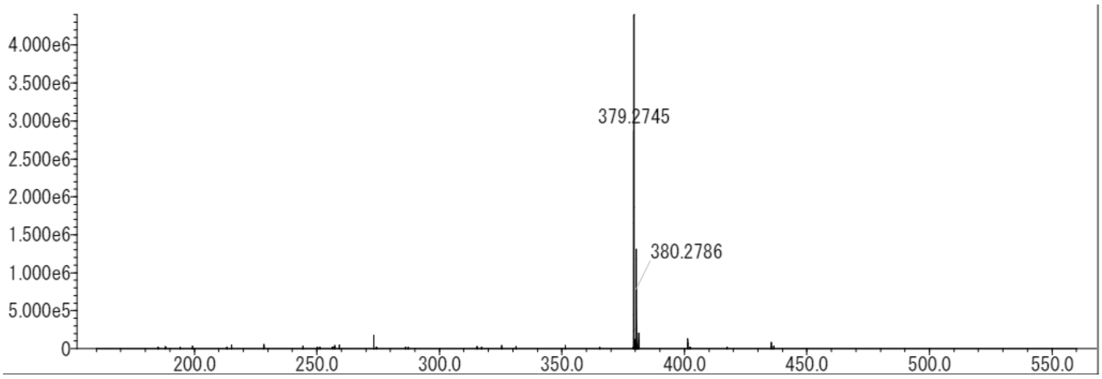

Formula [M+H]⁺	Theoretical *m/z*	Found *m/z*
$C_{25}H_{35}N_2O$	379.2744	379.2745

^{1}H NMR spectrum of compound **27** ($CDCl_3$)

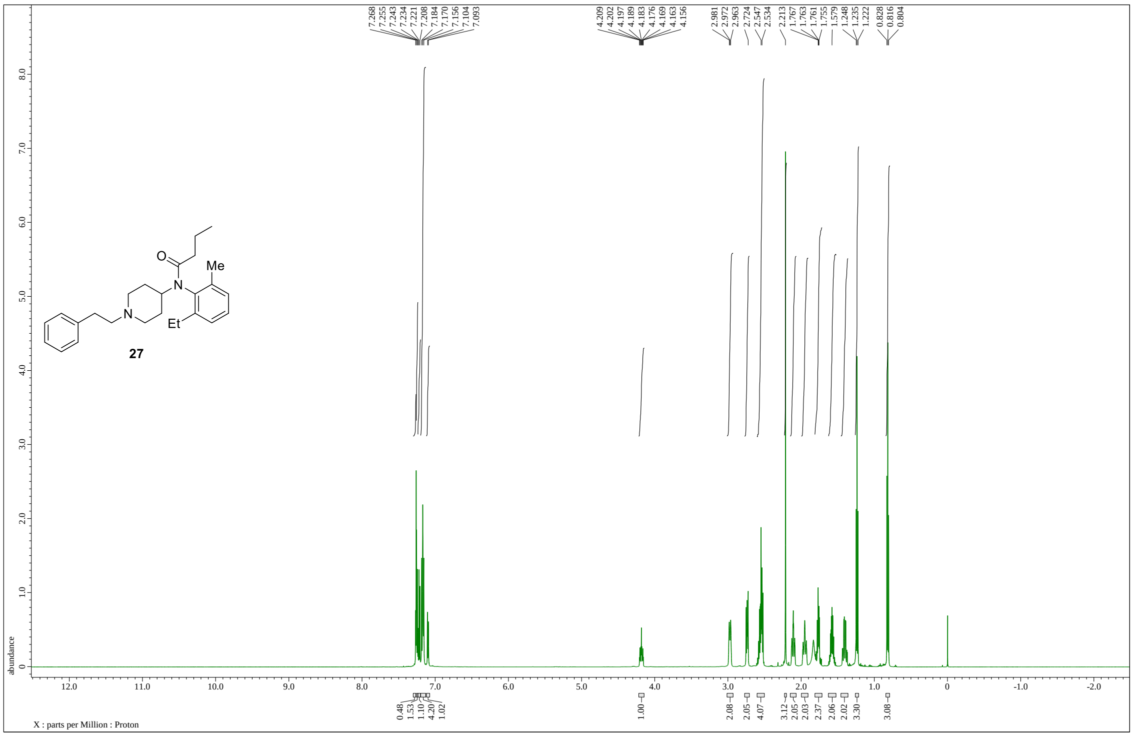

^{13}C NMR spectrum of compound **27** ($CDCl_3$)

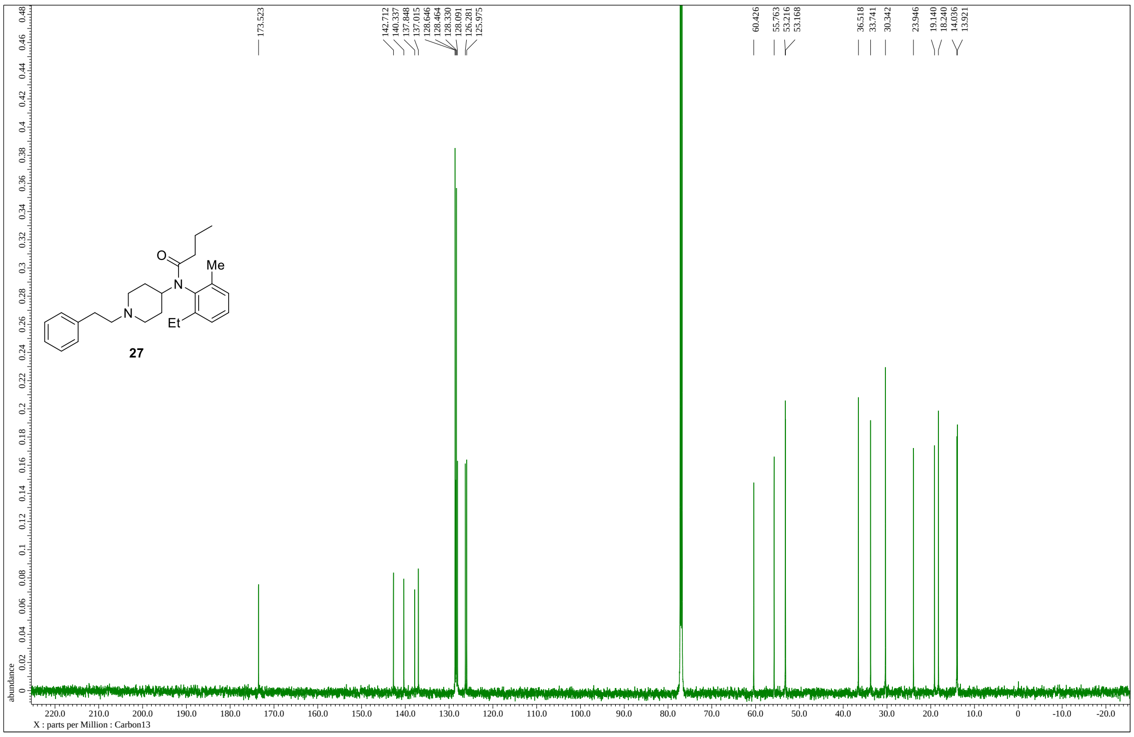

HRMS of compound **27**

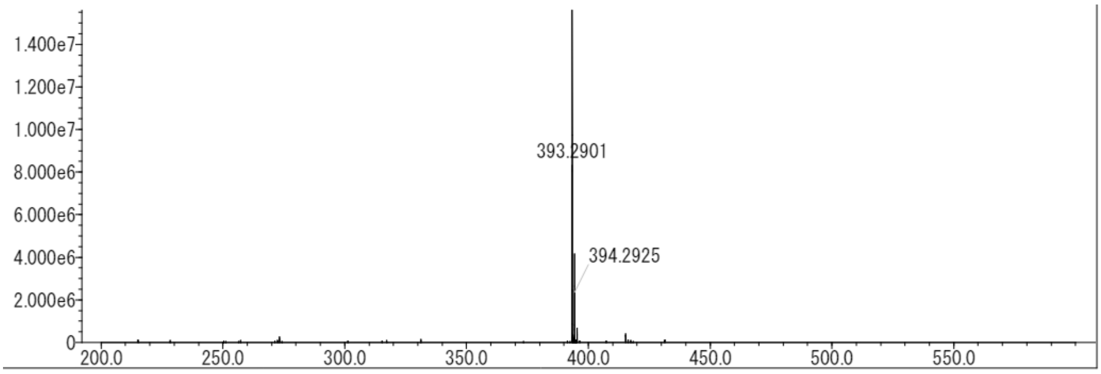

Formula [M+H]+	Theoretical *m/z*	Found *m/z*
$C_{26}H_{37}N_2O$	393.2900	393.2901

^{1}H NMR spectrum of compound **28** ($CDCl_3$)

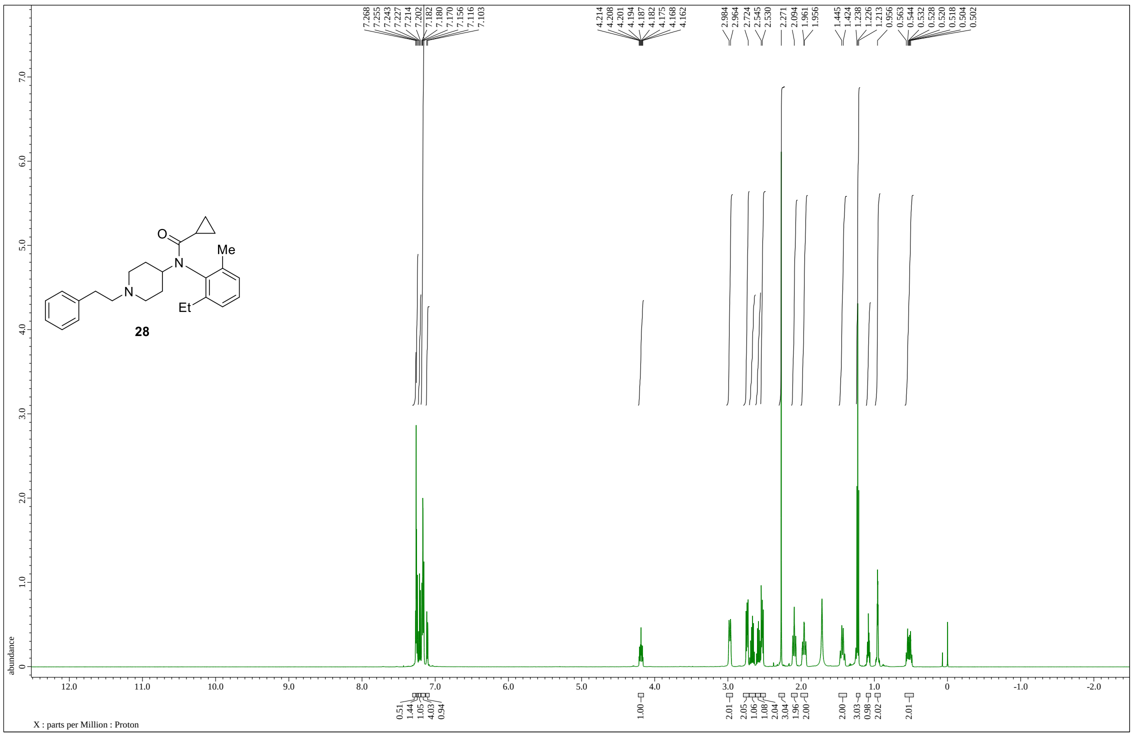

^{13}C NMR spectrum of compound **28** ($CDCl_3$)

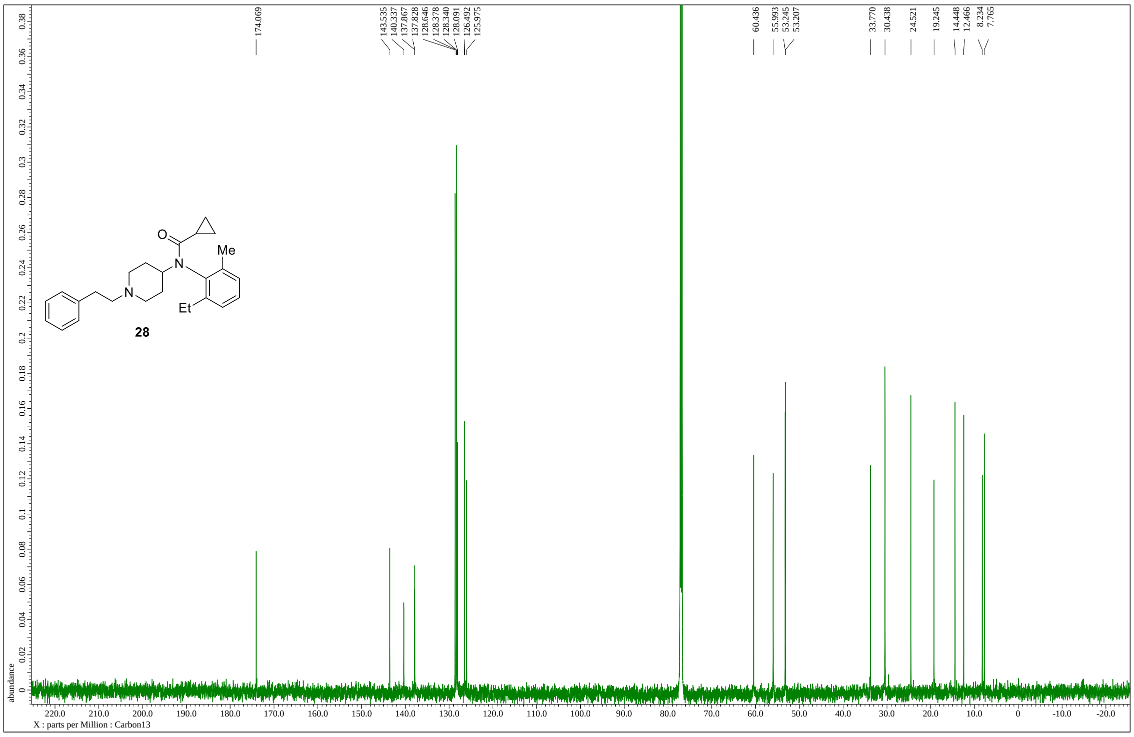

HRMS of compound **28**

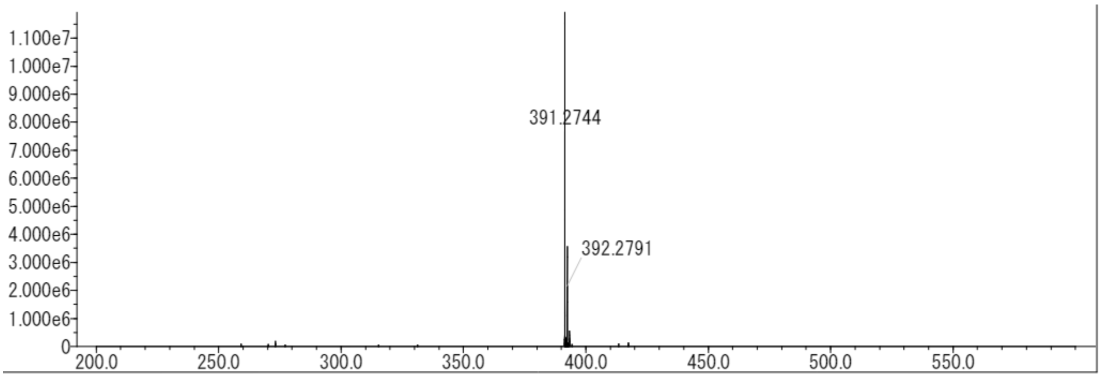

Formula [M+H]+	Theoretical *m/z*	Found *m/z*
$C_{26}H_{35}N_2O$	391.2744	391.2744

^{1}H NMR spectrum of compound **29** ($CDCl_3$)

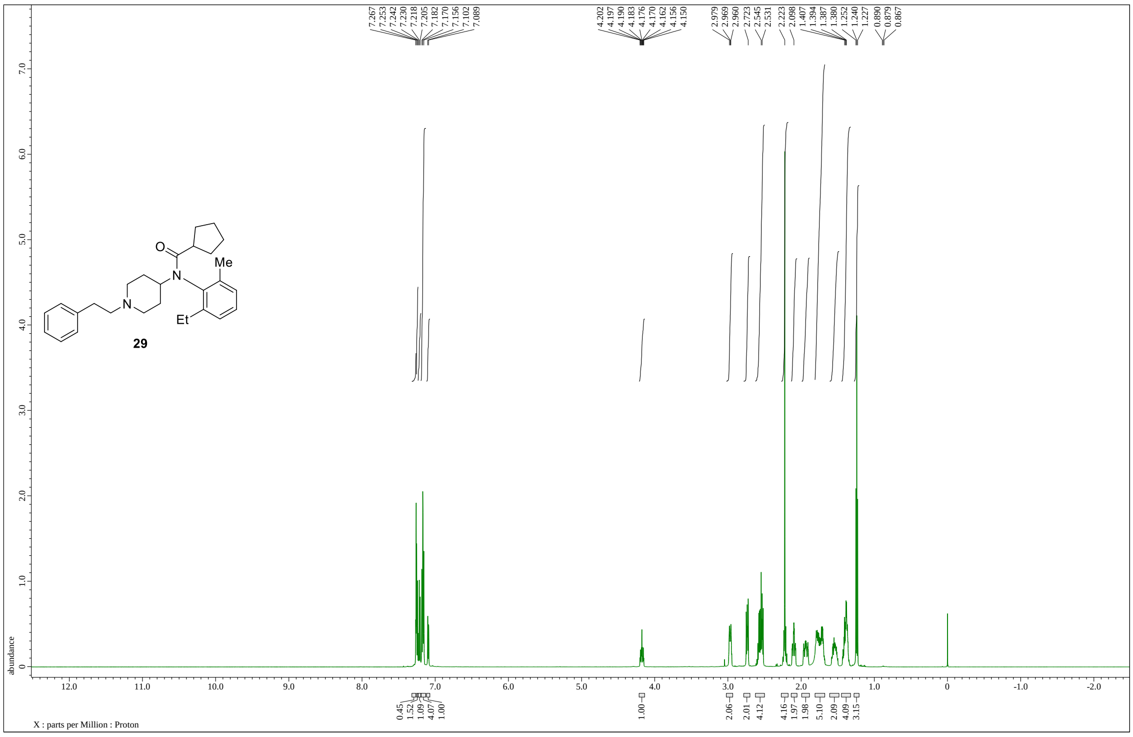

^{13}C NMR spectrum of compound **29**($CDCl_3$)

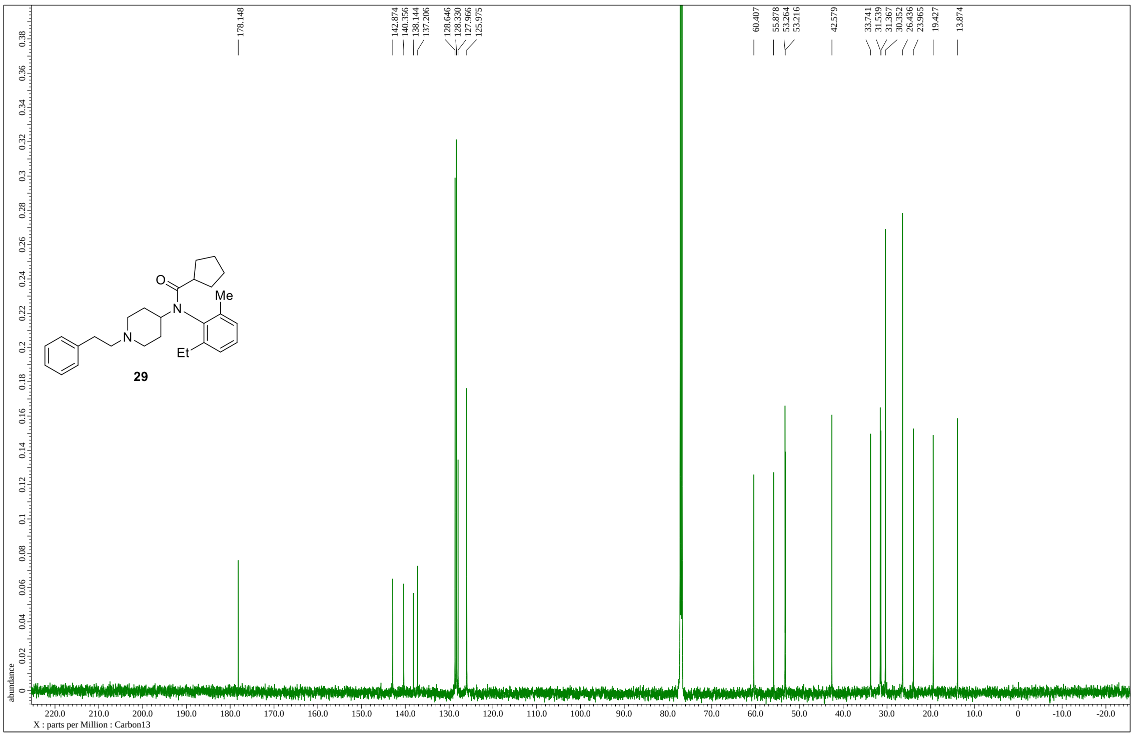

HRMS of compound **29**

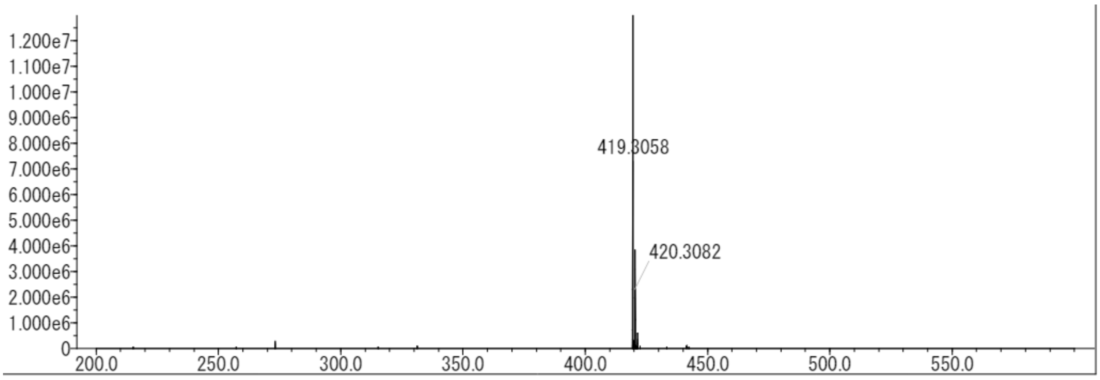

Formula $[M+H]^+$	Theoretical *m/z*	Found *m/z*
$C_{28}H_{39}N_2O$	419.3057	419.3058

^{1}H NMR spectrum of compound **30** ($CDCl_3$)

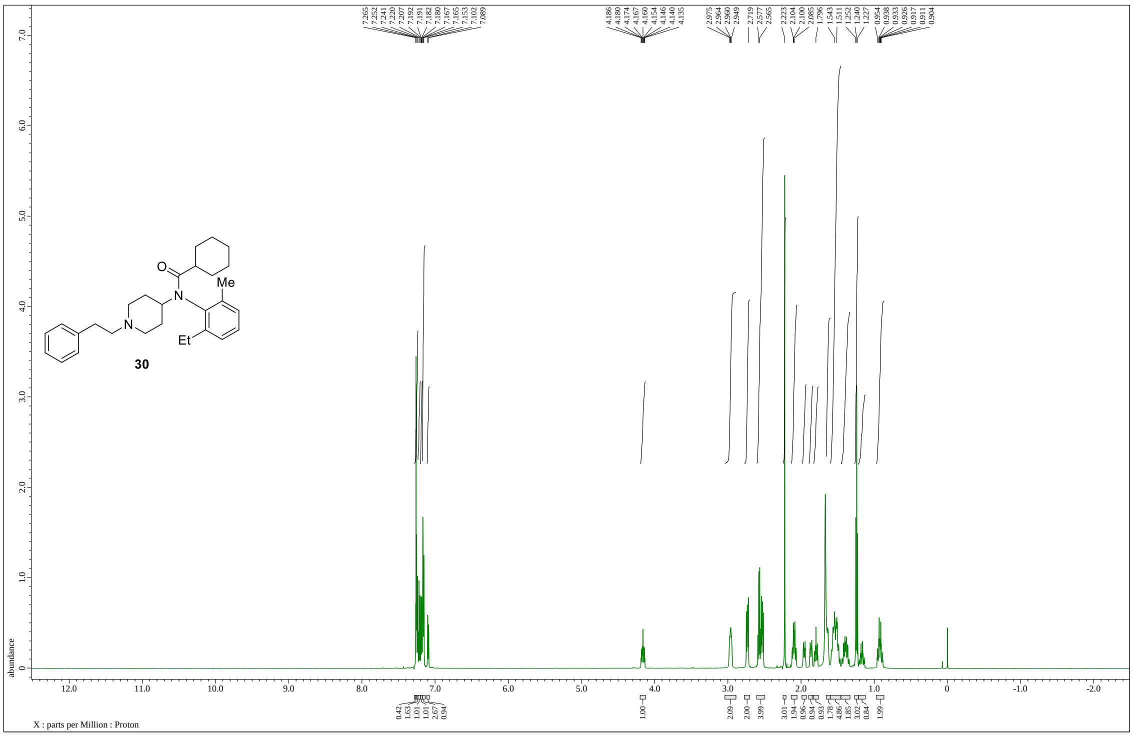

^{13}C NMR spectrum of compound **30** ($CDCl_3$)

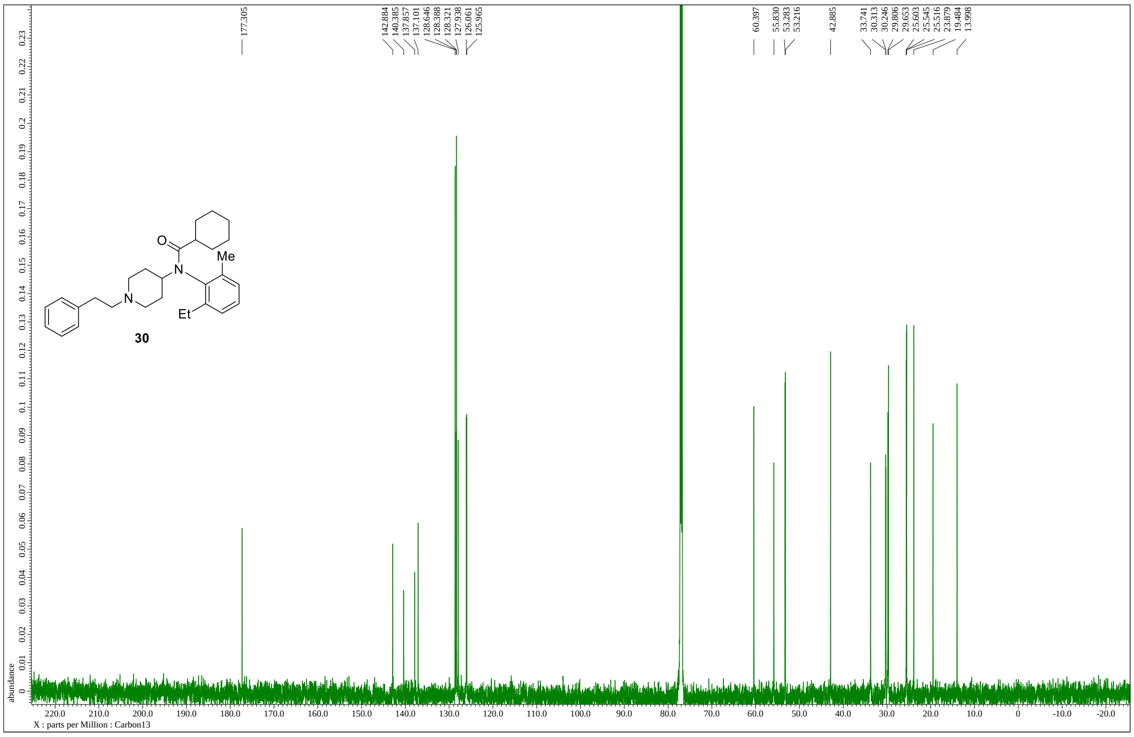

HRMS of compound **30**

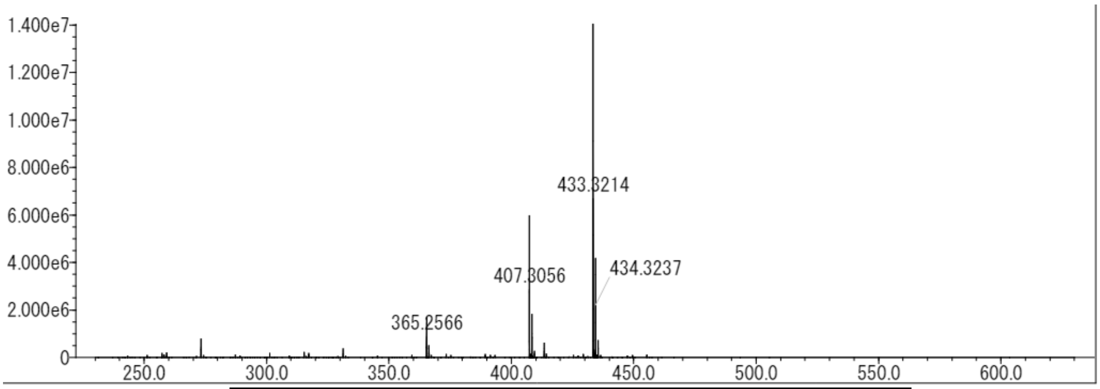

Formula [M+H]+	Theoretical *m/z*	Found *m/z*
$C_{29}H_{41}N_2O$	433.3213	433.3214

^{1}H NMR spectrum of compound **31** ($CDCl_3$)

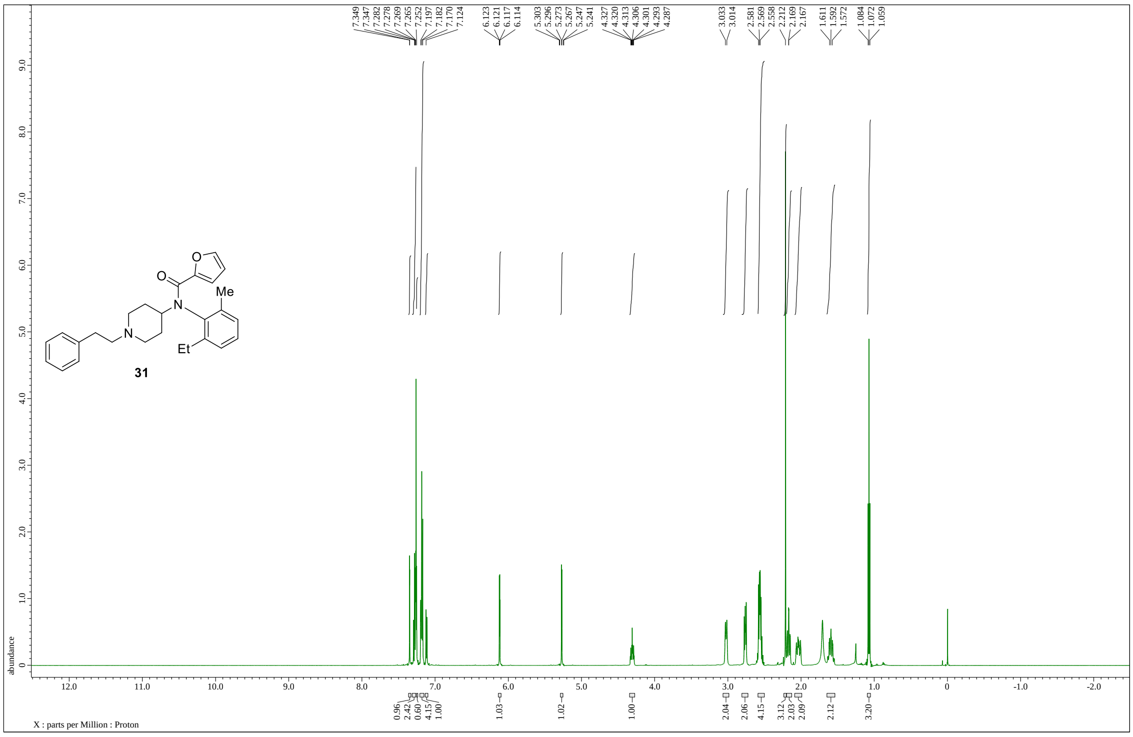

^{13}C NMR spectrum of compound **31** ($CDCl_3$)

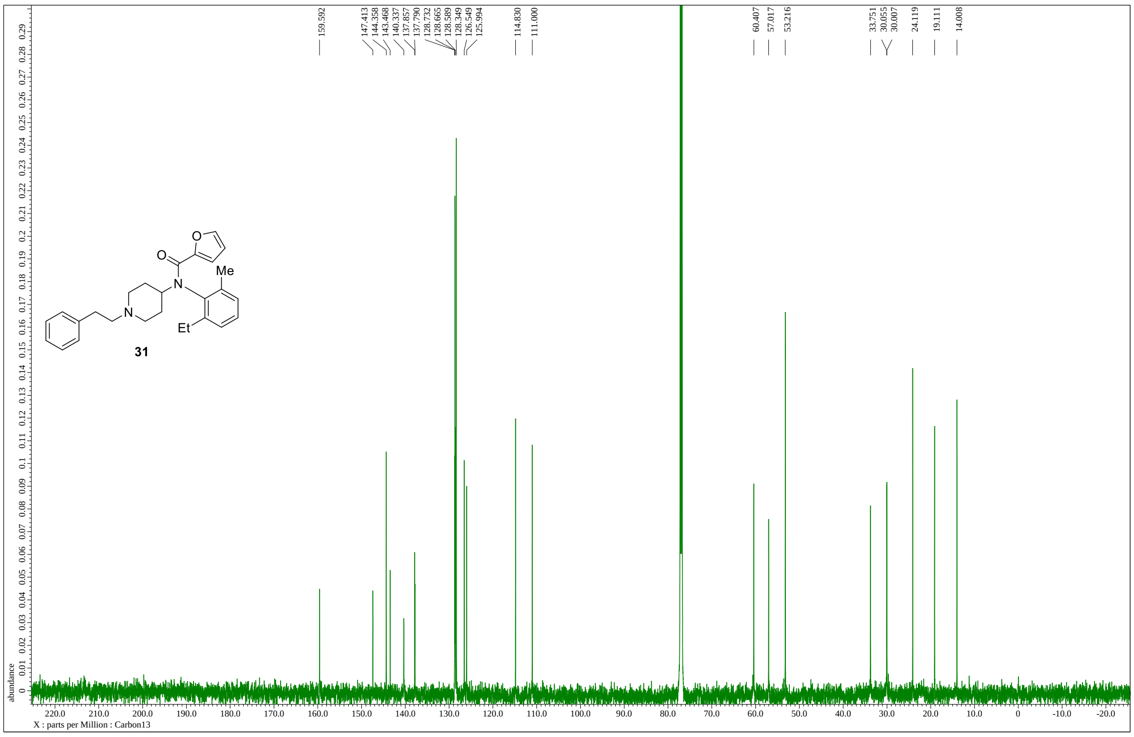

HRMS of compound **31**

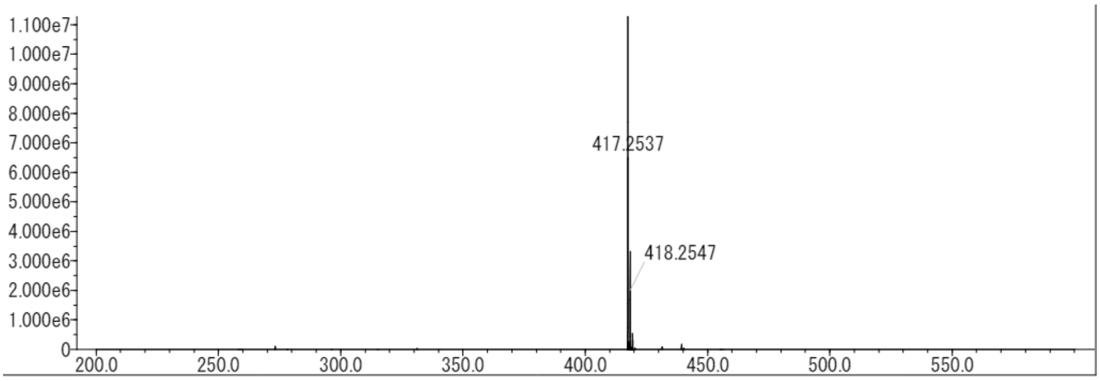

Formula [M+H]+	Theoretical *m/z*	Found *m/z*
$C_{27}H_{33}N_2O_2$	417.2537	417.2537

^1H NMR spectrum of compound **32** ($CDCl_3$)

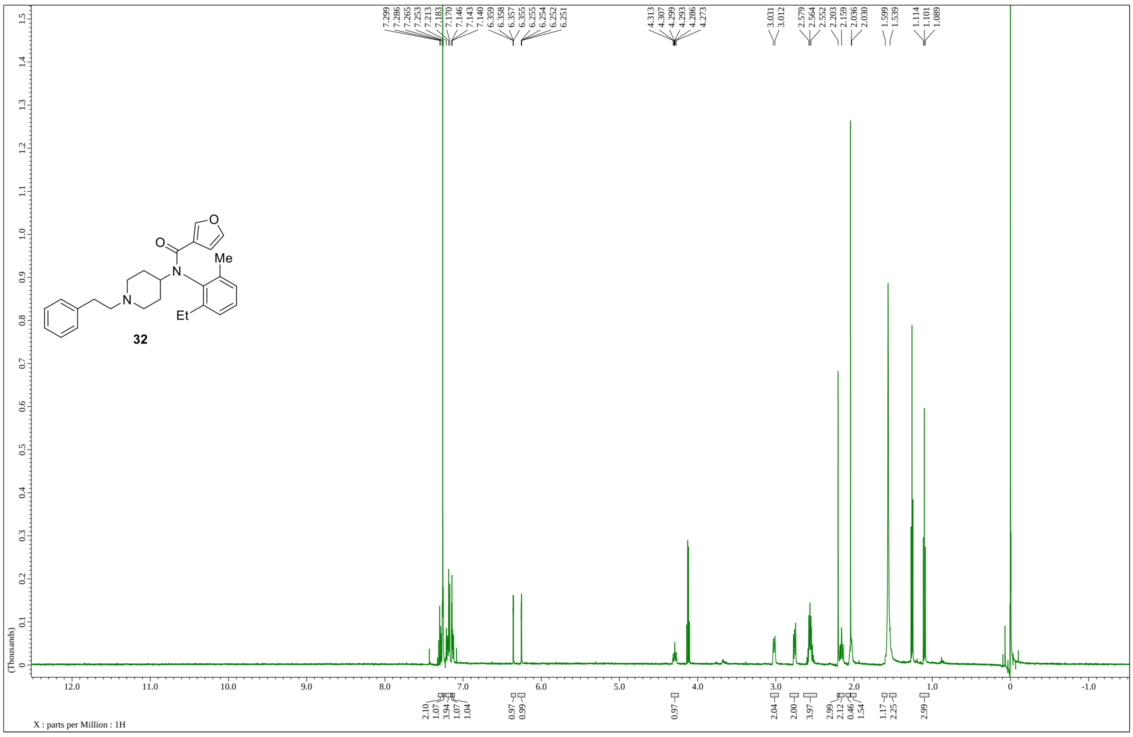

^{13}C NMR spectrum of compound **32** ($CDCl_3$)

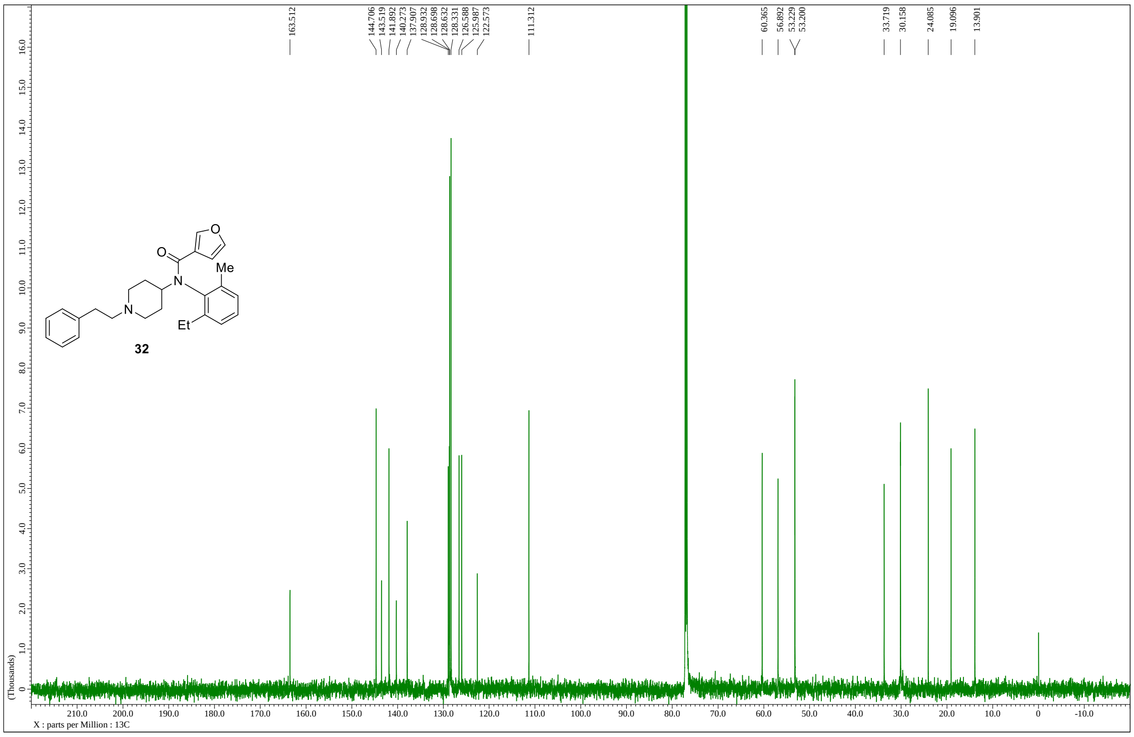

HRMS of compound **32**

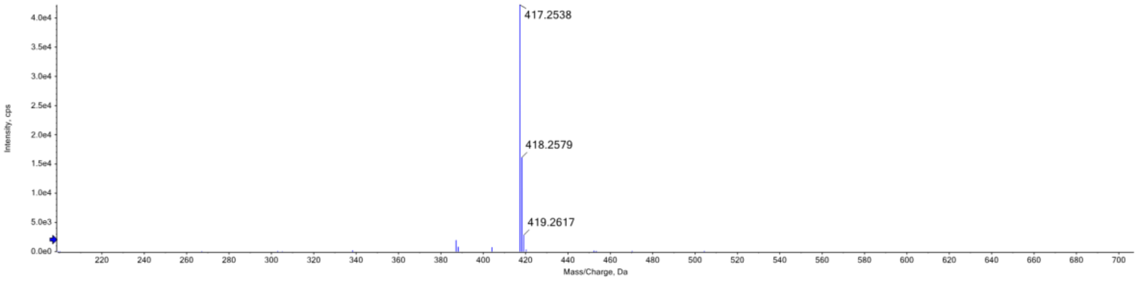

Formula $[M+H]^+$	Theoretical *m/z*	Found *m/z*
$C_{27}H_{33}N_2O_2$	417.2537	417.2538

^{1}H NMR spectrum of compound **33** ($CDCl_3$)

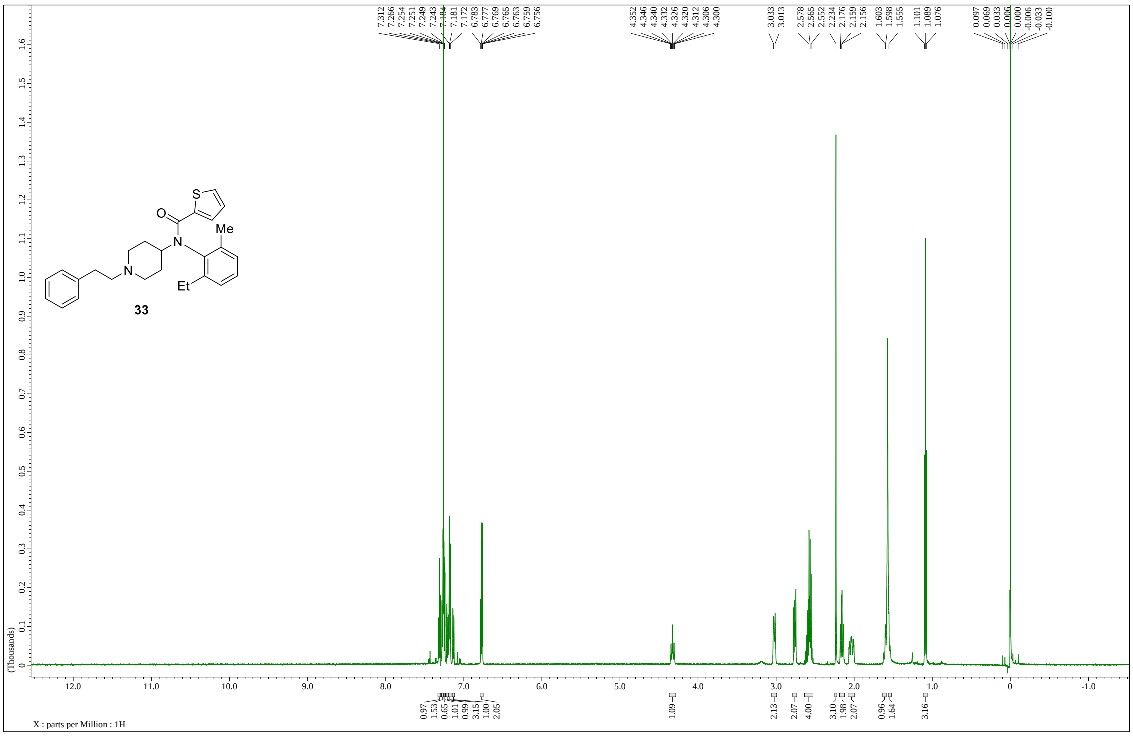

^{13}C NMR spectrum of compound **33** ($CDCl_3$)

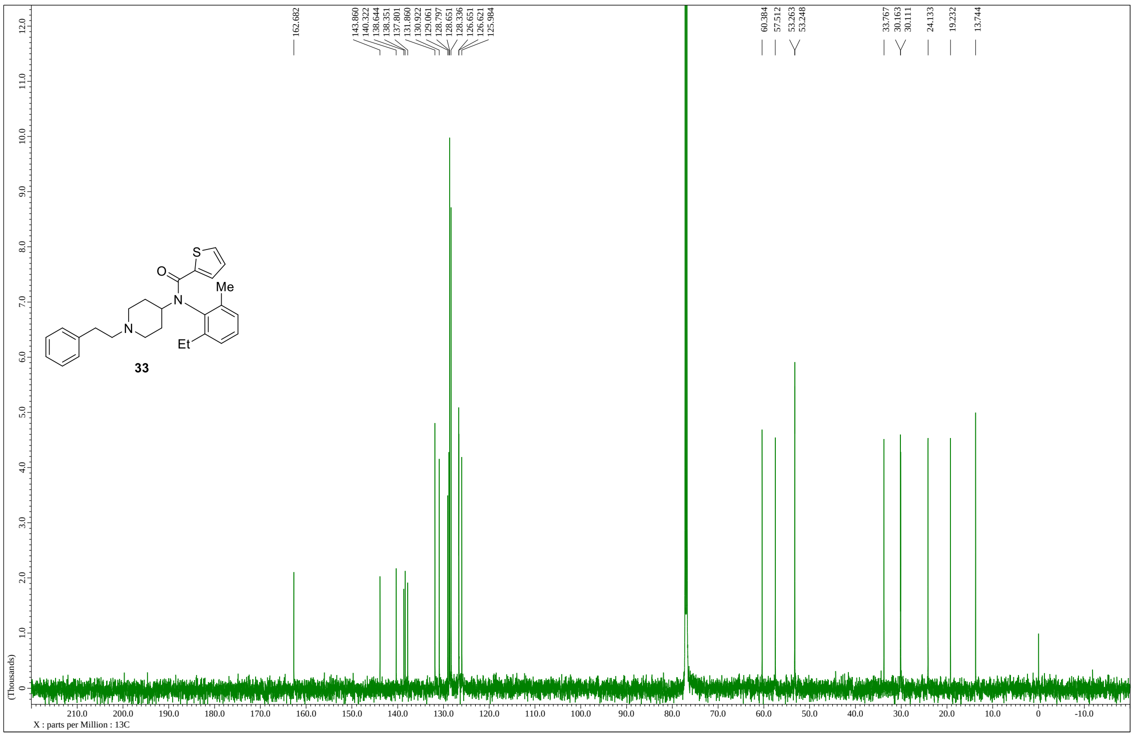

HRMS of compound **33**

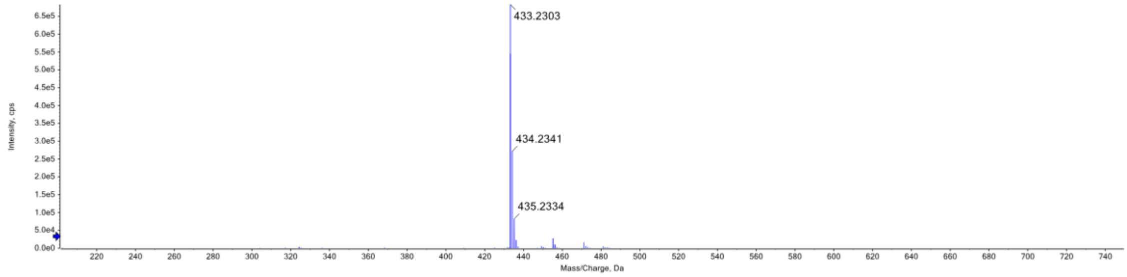

Formula [M+H]$^+$	Theoretical *m/z*	Found *m/z*
$C_{27}H_{33}N_2OS$	433.2308	433.2303

^{1}H NMR spectrum of compound **34** ($CDCl_3$)

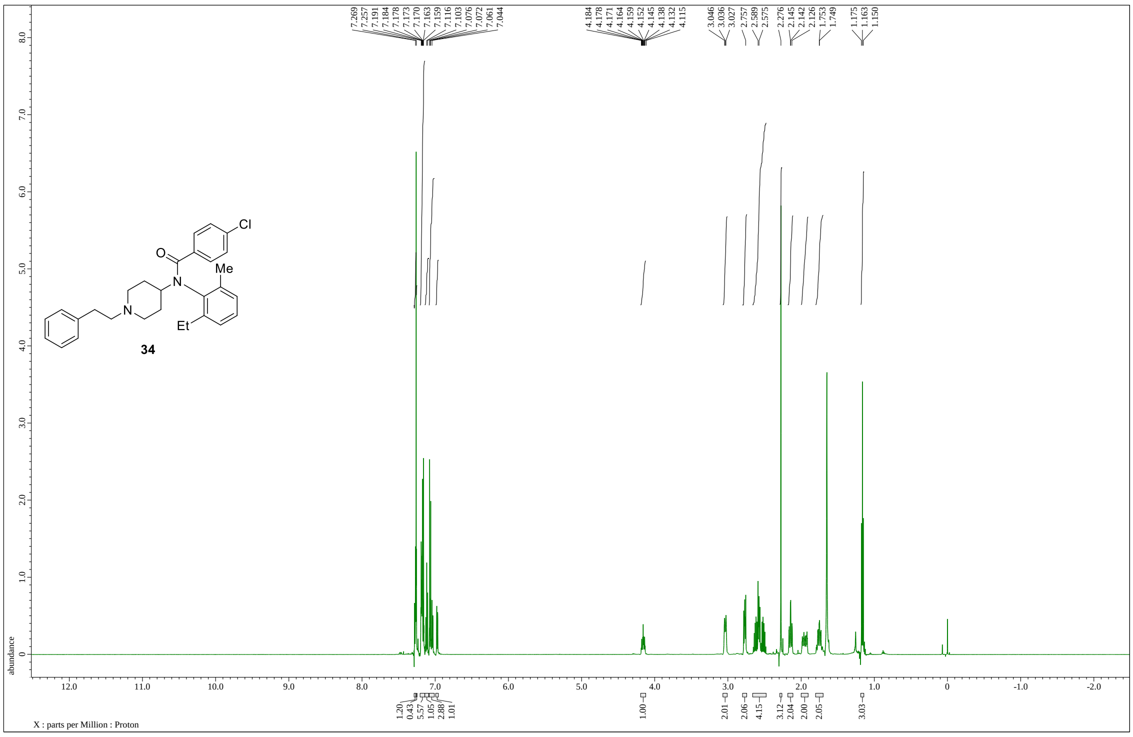

^{13}C NMR spectrum of compound **34** ($CDCl_3$)

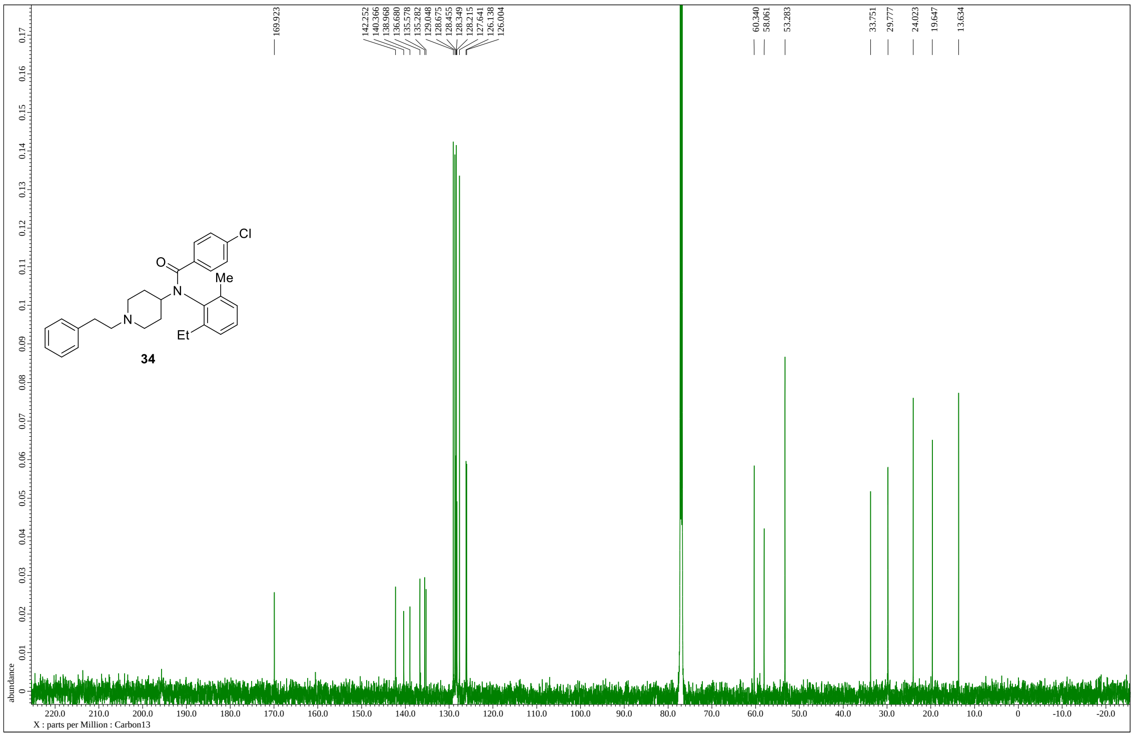

HRMS of compound **34**

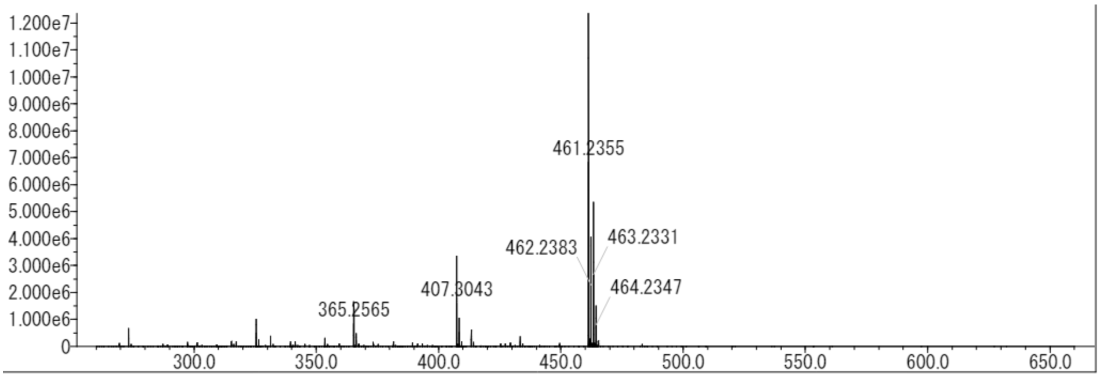

Formula $[M+H]^+$	Theoretical *m/z*	Found *m/z*
$C_{29}H_{34}ClN_2O$	461.2354	461.2355

^1H NMR spectrum of compound **35** ($CDCl_3$)

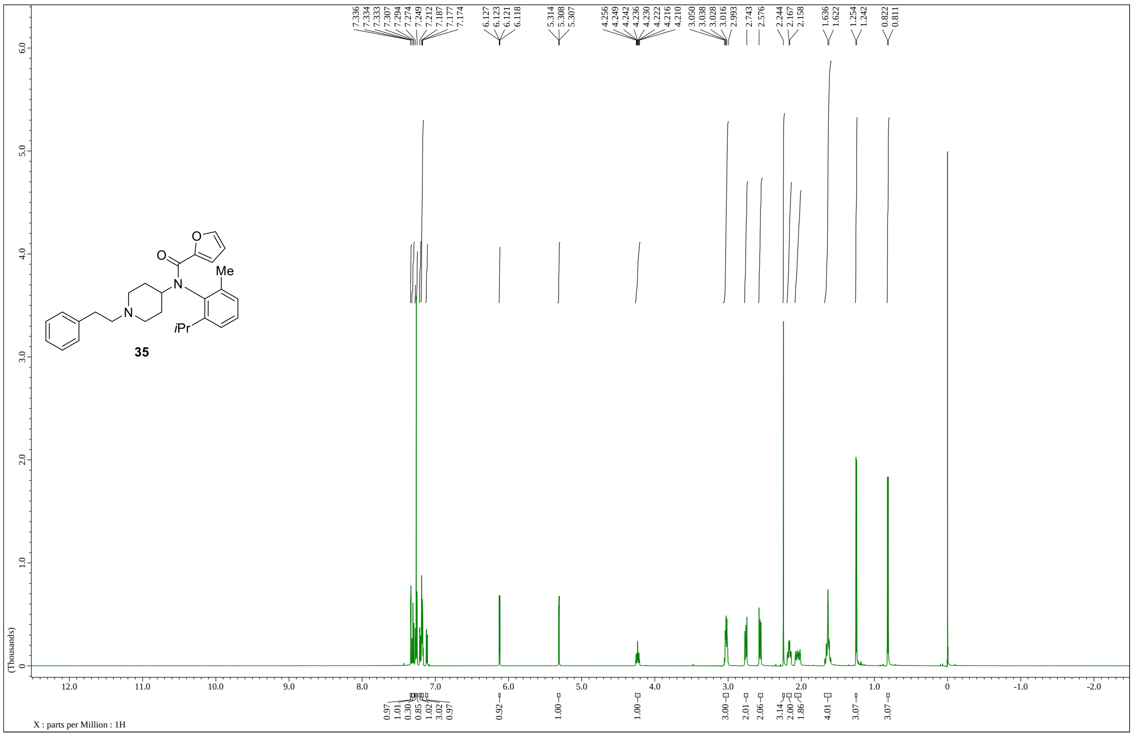

^{13}C NMR spectrum of compound **35** ($CDCl_3$)

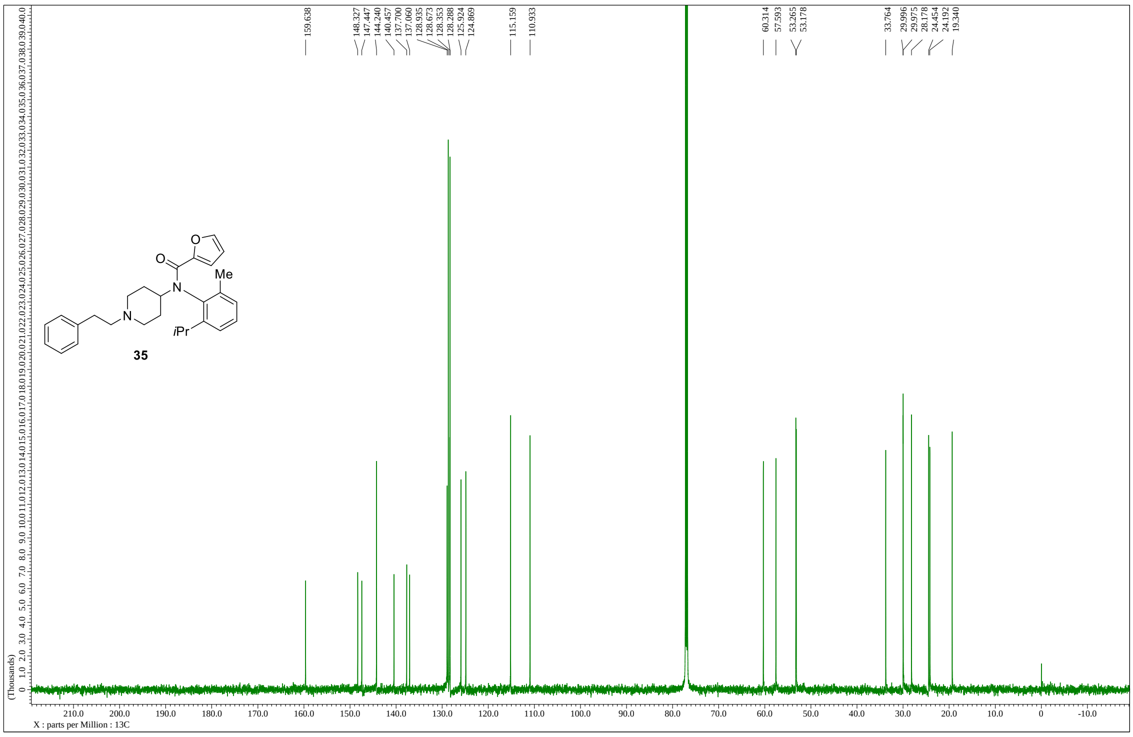

HRMS of compound **35**

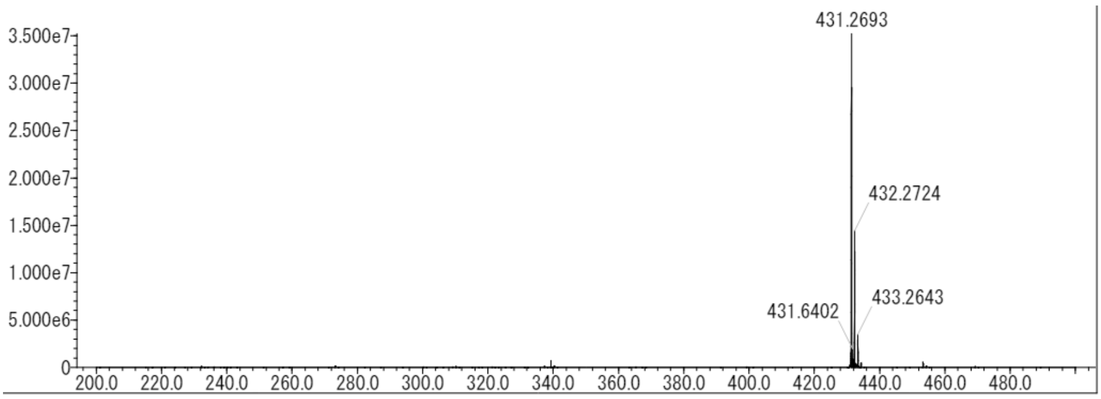

Formula [M+H]⁺	Theoretical *m/z*	Found *m/z*
$C_{28}H_{35}N_2O_2$	431.2693	431.2693

^{1}H NMR spectrum of compound **36** ($CDCl_3$)

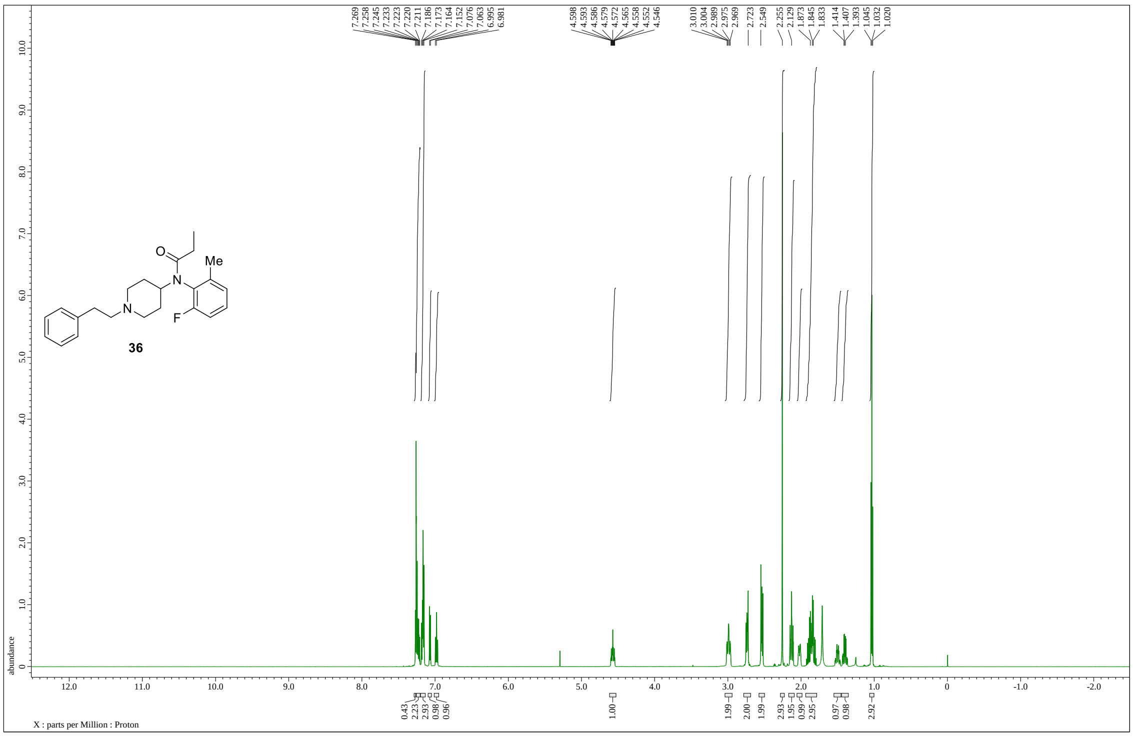

^{13}C NMR spectrum of compound **36** ($CDCl_3$)

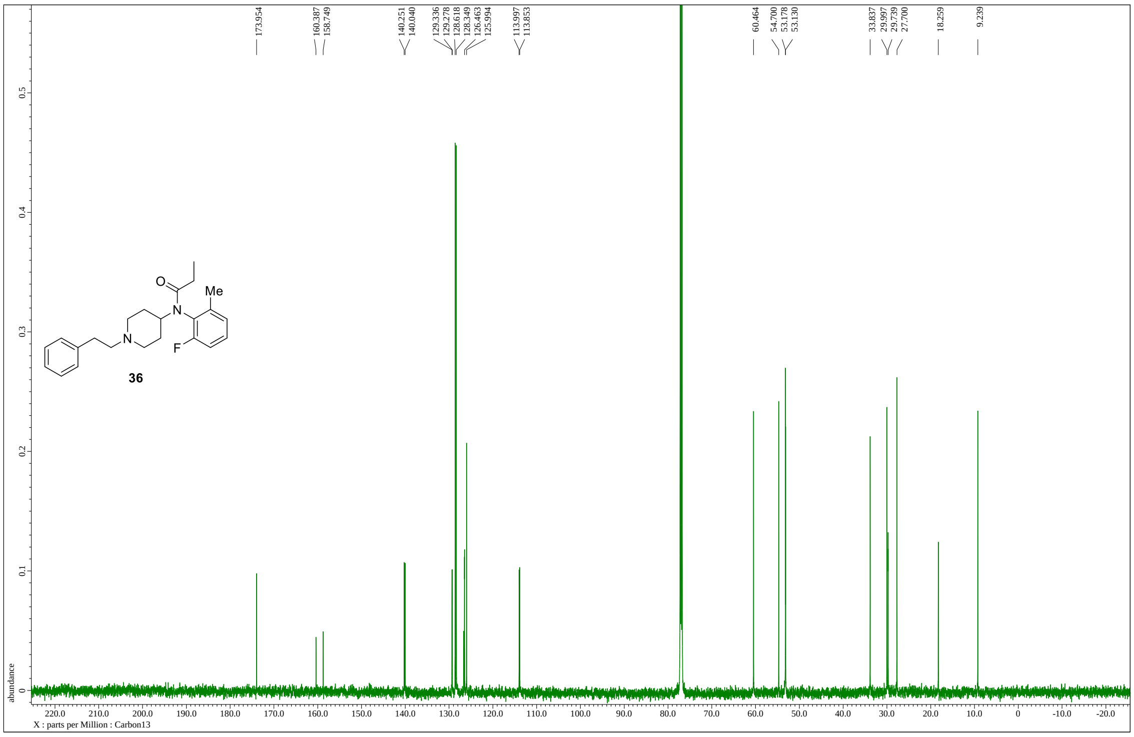

HRMS of compound **36**

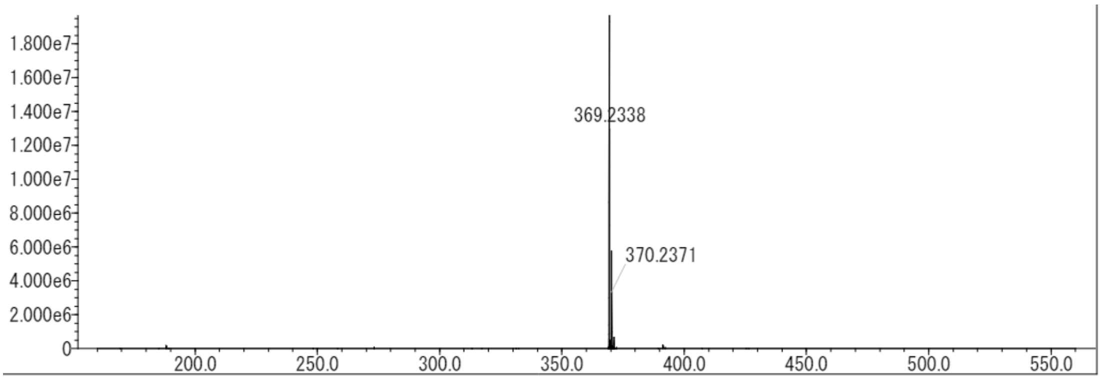

Formula [M+H]+	Theoretical *m/z*	Found *m/z*
$C_{23}H_{30}FN_2O$	369.2337	369.2338

^{1}H NMR spectrum of compound **37** ($CDCl_3$)

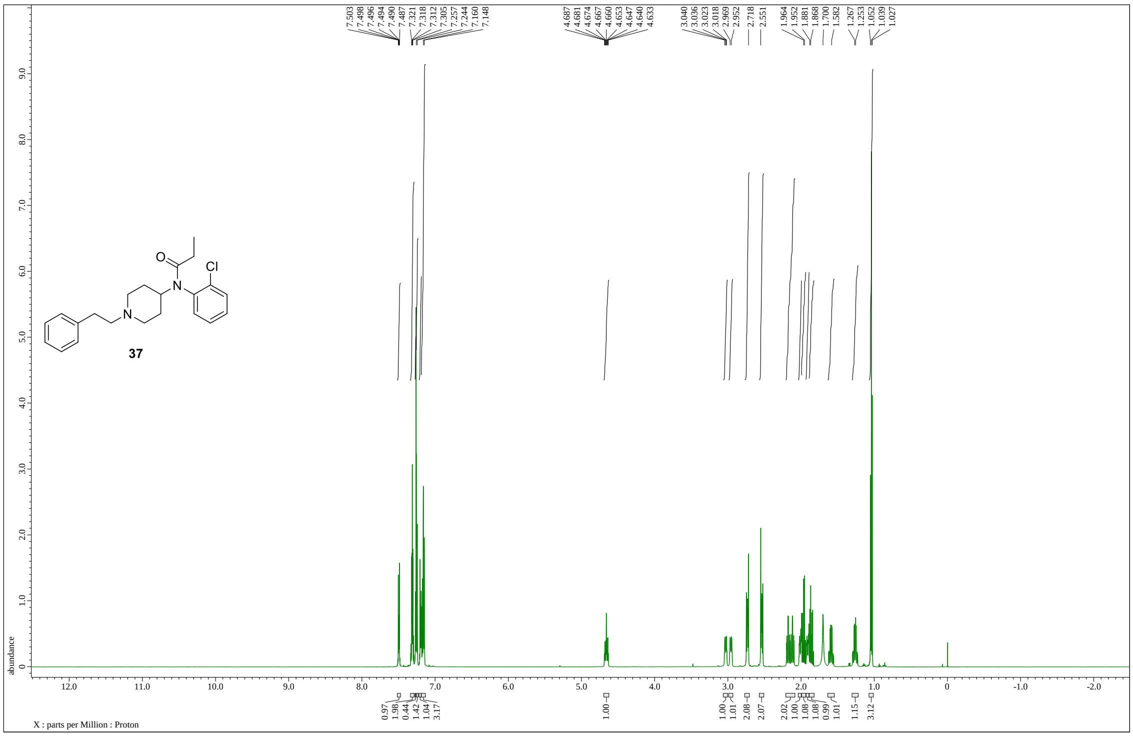

^{13}C NMR spectrum of compound **37** ($CDCl_3$)

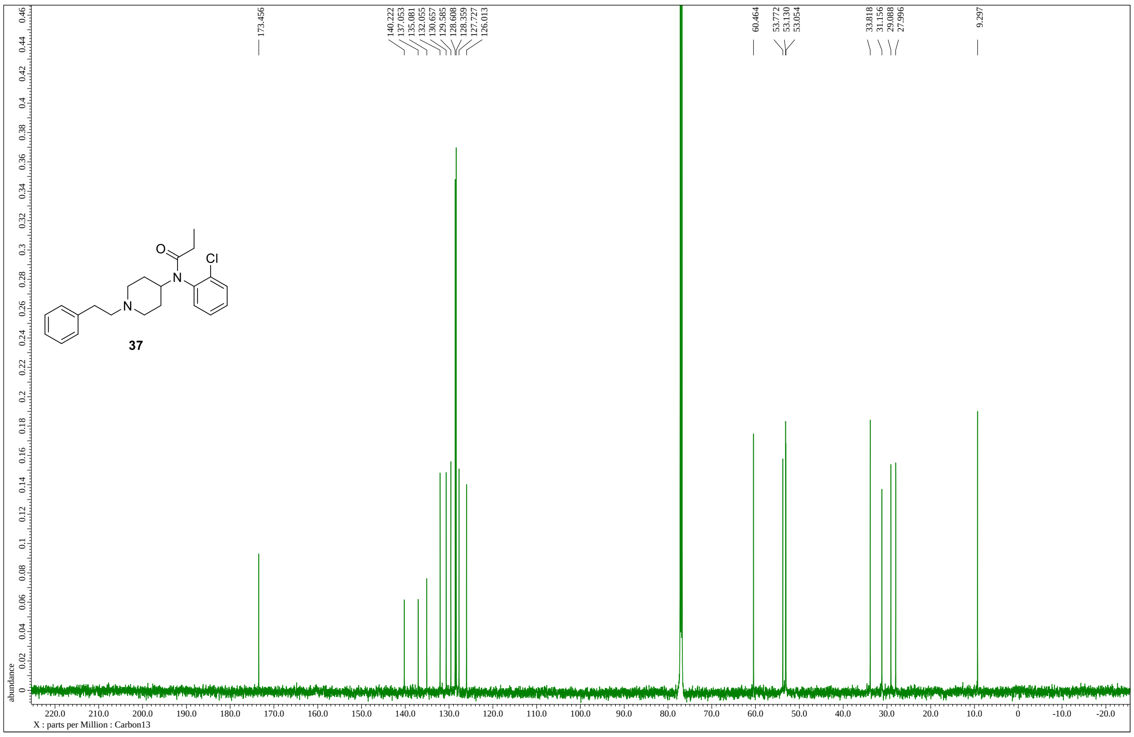

HRMS of compound **37**

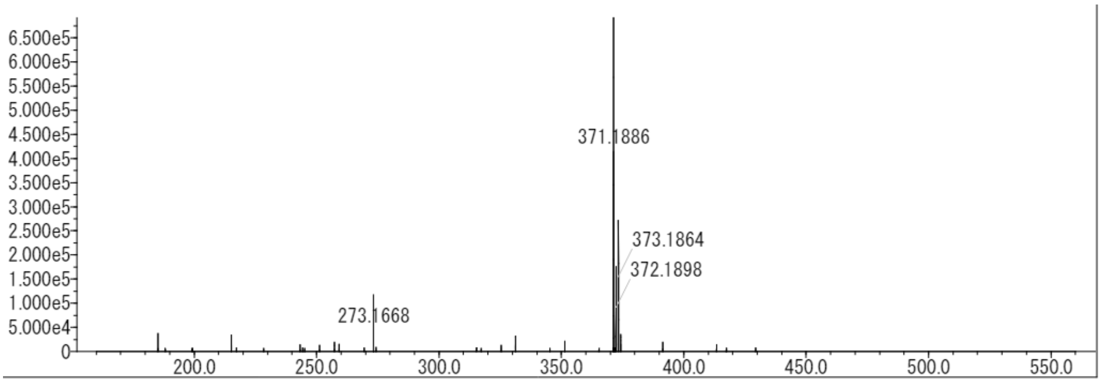

Formula $[M+H]^+$	Theoretical *m/z*	Found *m/z*
$C_{22}H_{28}ClN_2O$	371.1885	371.1886

^{1}H NMR spectrum of compound **38** ($CDCl_3$)

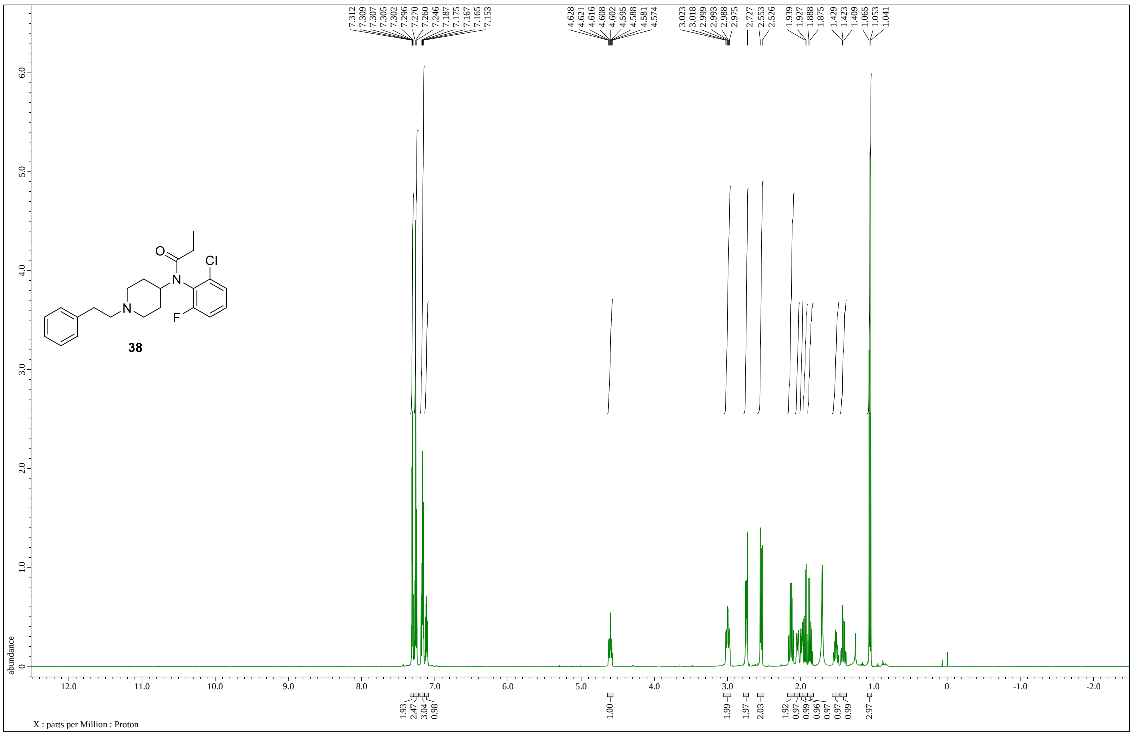

^{13}C NMR spectrum of compound **38** ($CDCl_3$)

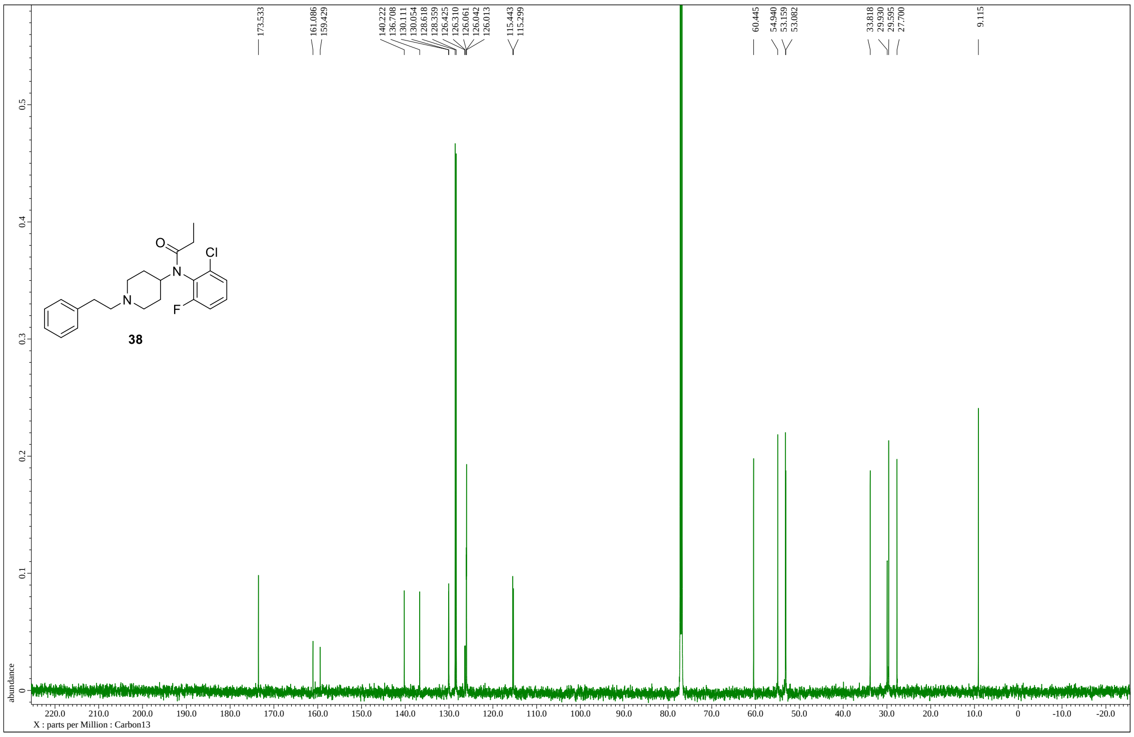

HRMS of compound **38**

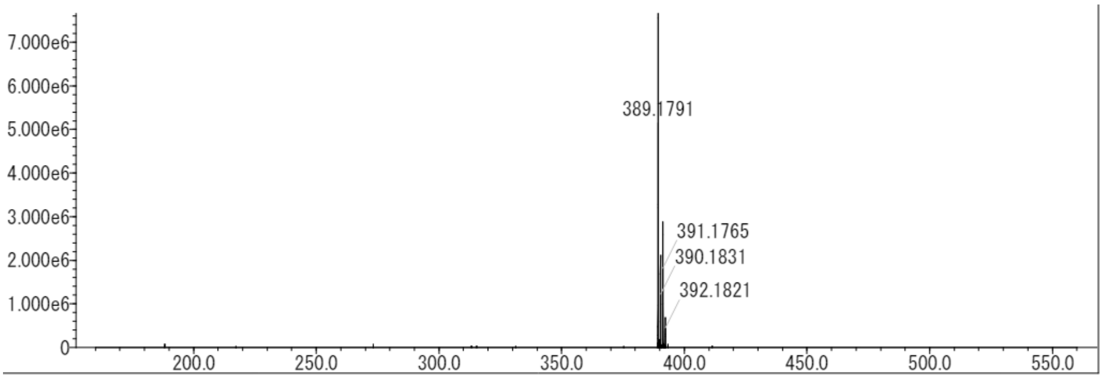

Formula $[M+H]^+$	Theoretical *m/z*	Found *m/z*
$C_{22}H_{27}ClFN_2O$	389.1790	389.1791

^{1}H NMR spectrum of compound **41** ($CDCl_3$)

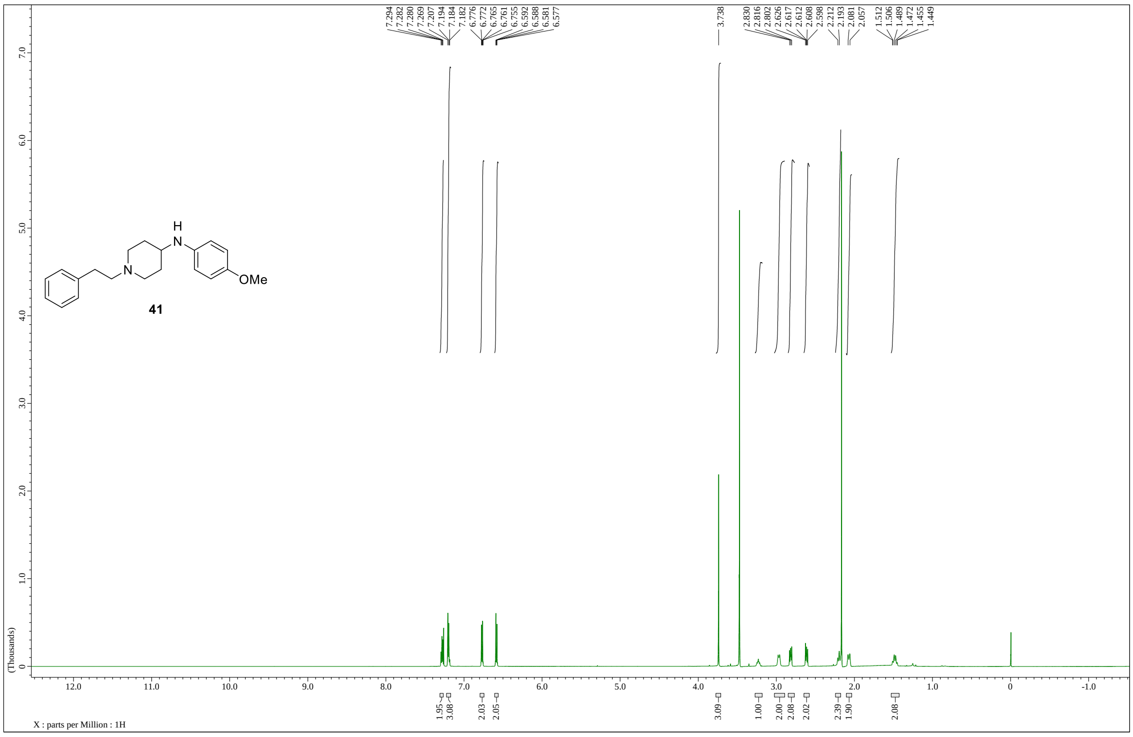

^{13}C NMR spectrum of compound **41** ($CDCl_3$)

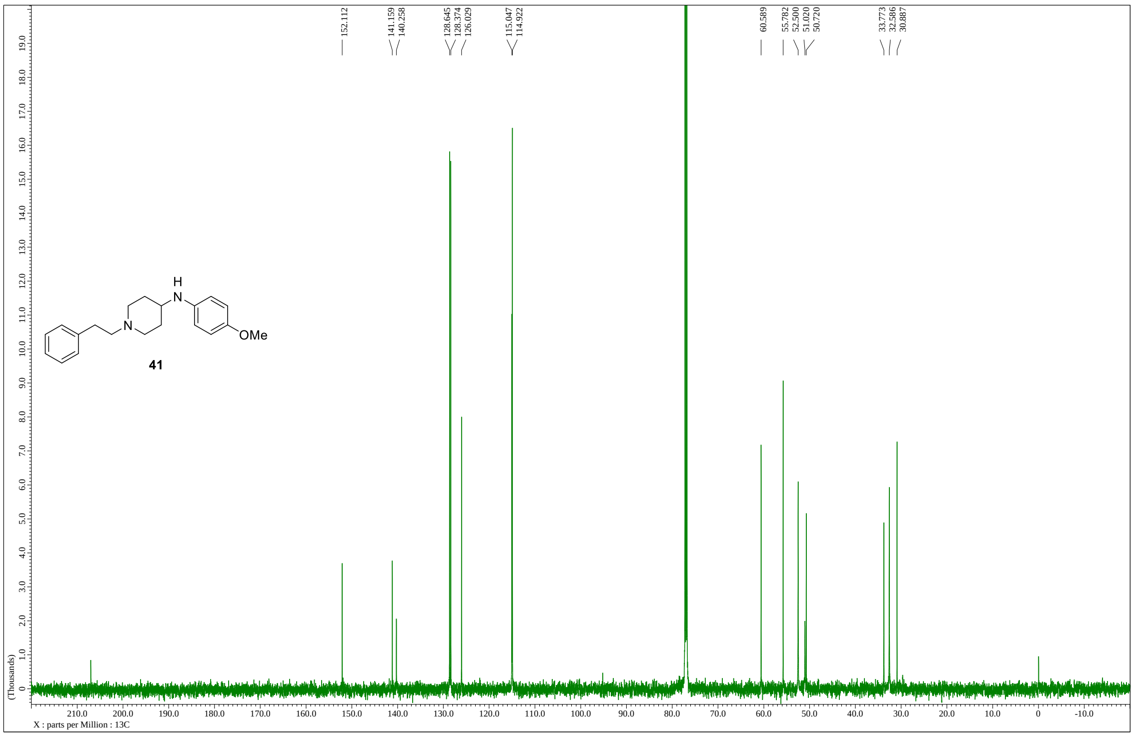

HRMS of compound **41**

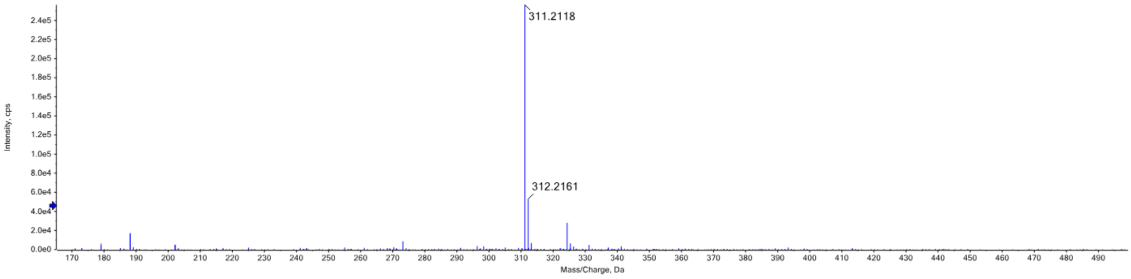

Formula [M+H]$^{+}$	Theoretical *m/z*	Found *m/z*
$C_{20}H_{27}N_2O$	311.2118	311.2118

^{1}H NMR spectrum of compound **42** ($CDCl_3$)

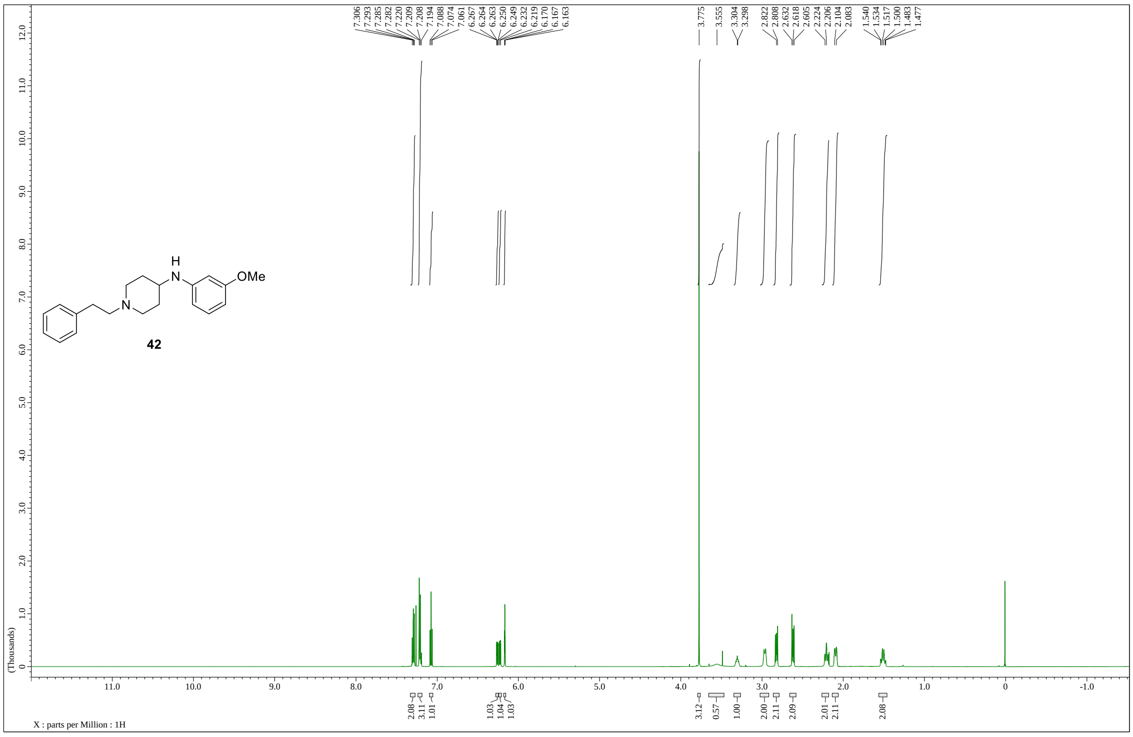

^{13}C NMR spectrum of compound **42** ($CDCl_3$)

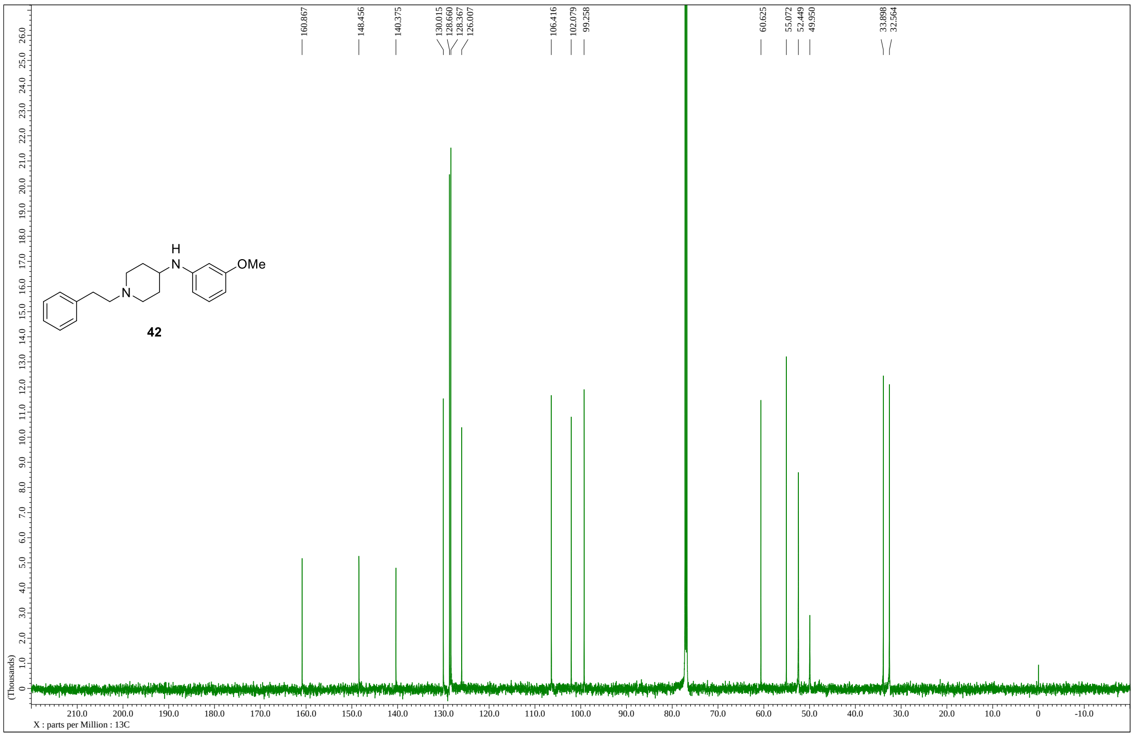

HRMS of compound **42**

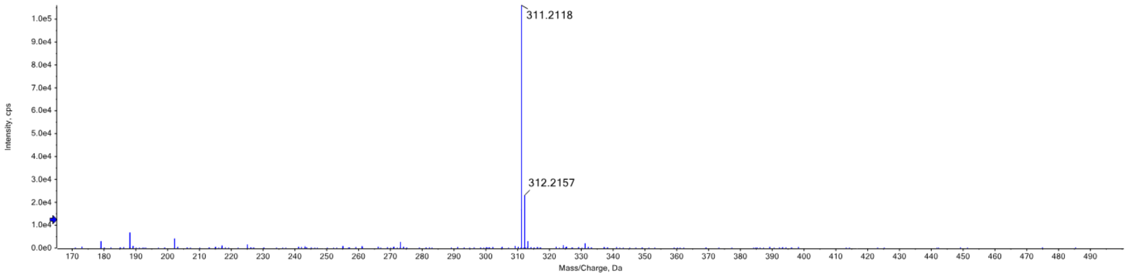

Formula $[M+H]^+$	Theoretical *m/z*	Found *m/z*
$C_{20}H_{27}N_2O$	311.2118	311.2118

^{1}H NMR spectrum of compound **43** ($CDCl_3$)

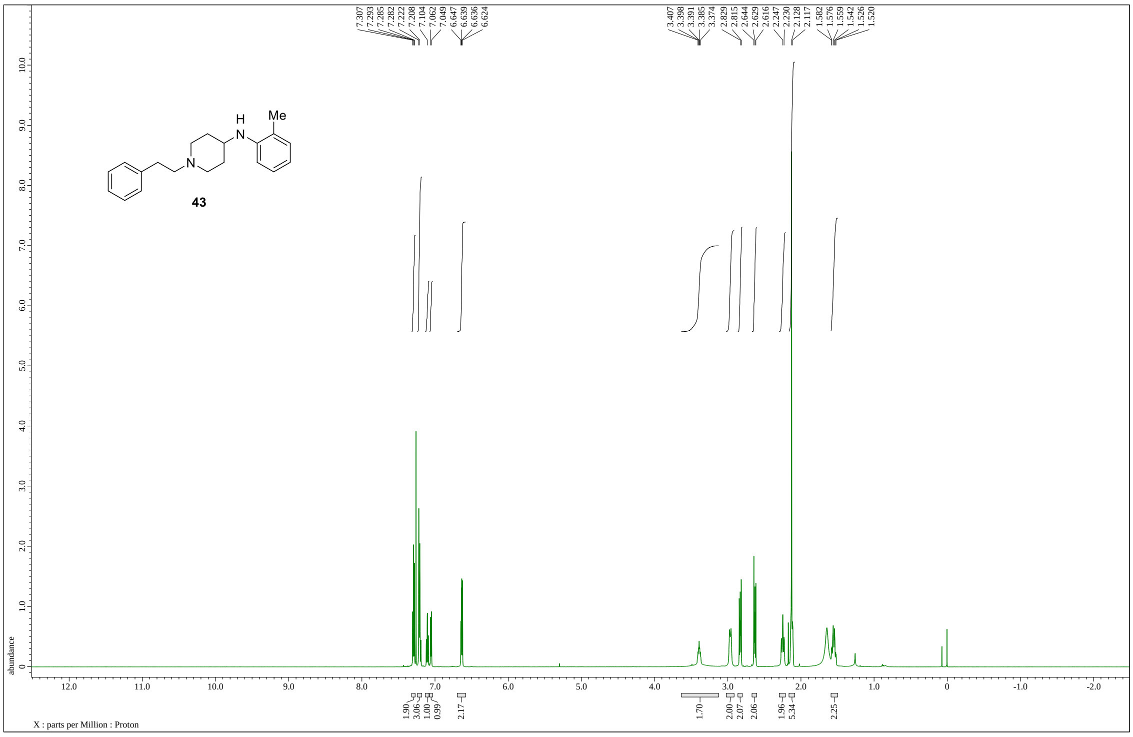

^{13}C NMR spectrum of compound **43** ($CDCl_3$)

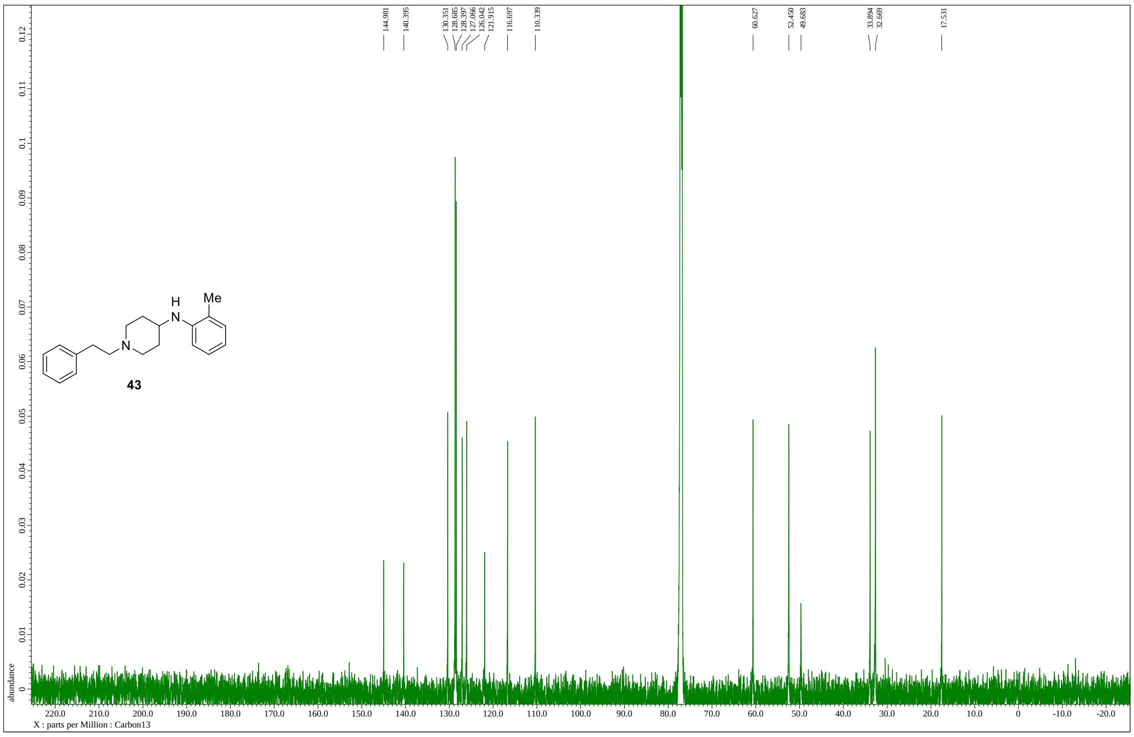

HRMS of compound **43**

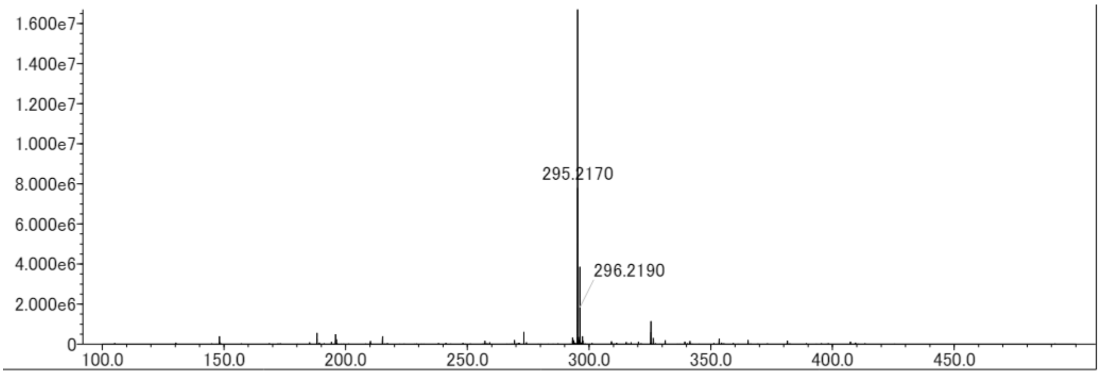

Formula $[M+H]^+$	Theoretical *m/z*	Found *m/z*
$C_{20}H_{27}N_2$	295.2169	295.2170

^{1}H NMR spectrum of compound **44** ($CDCl_3$)

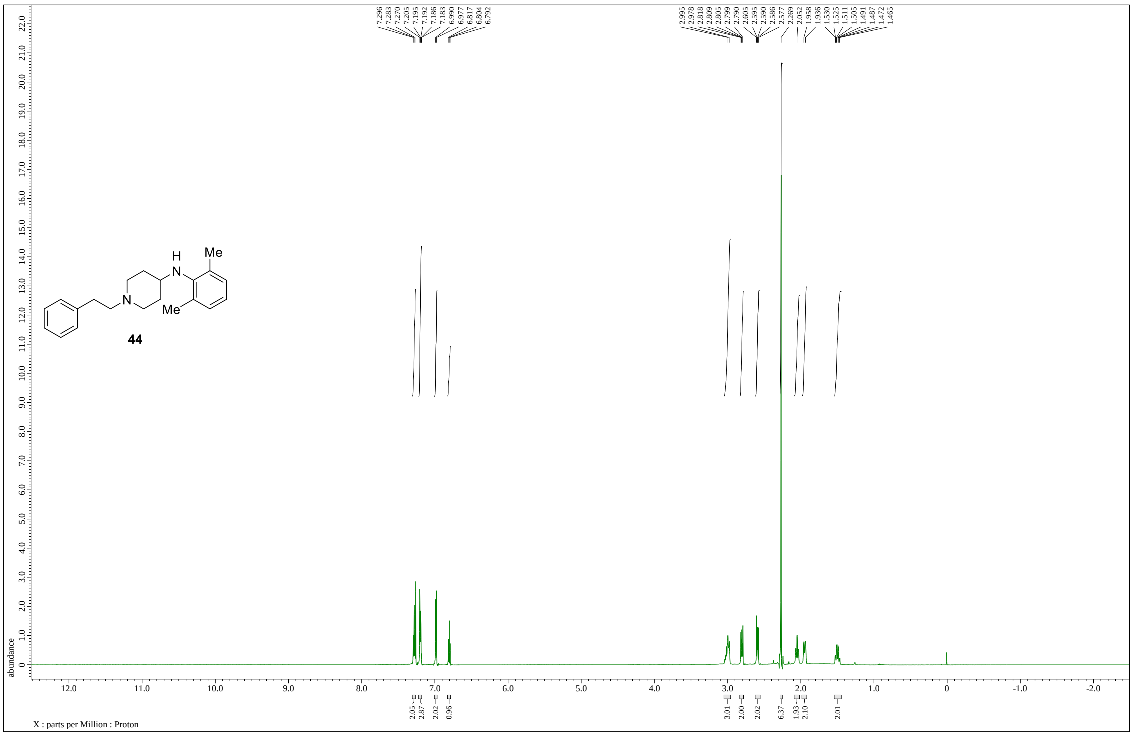

^{13}C NMR spectrum of compound **44** ($CDCl_3$)

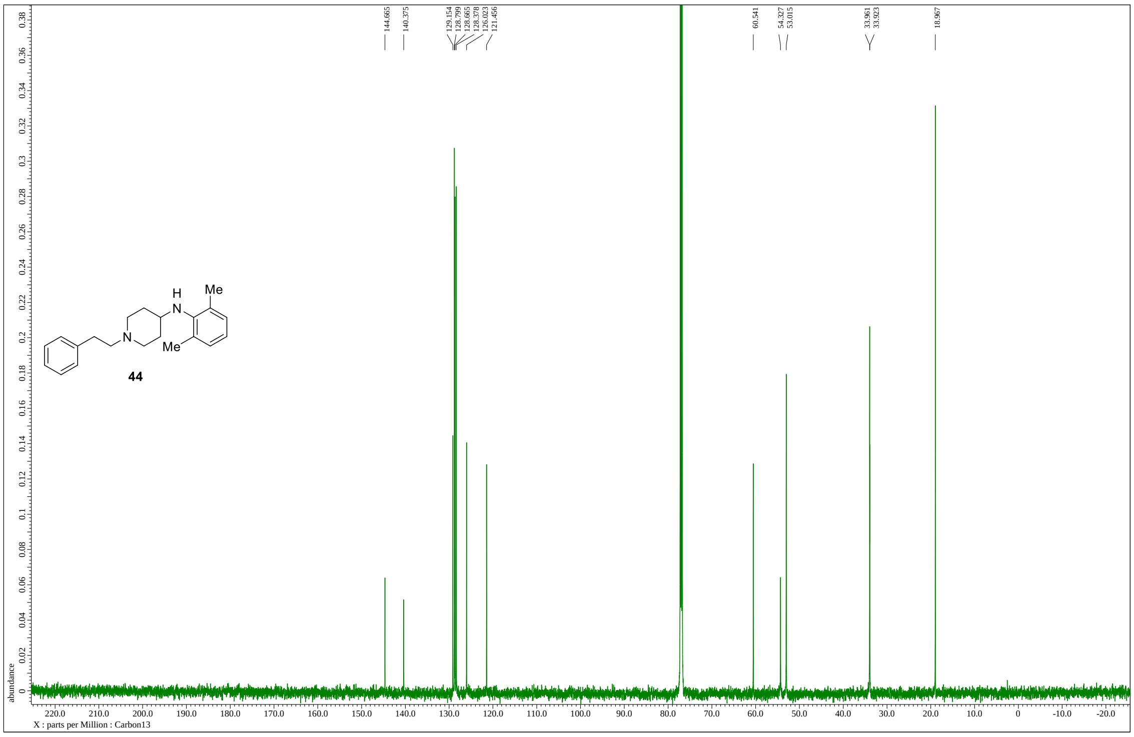

HRMS of compound **44**

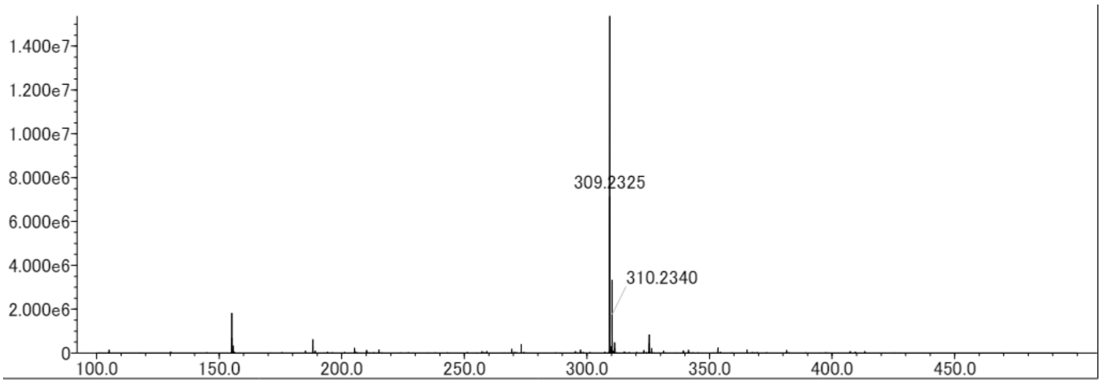

Formula [M+H]+	Theoretical *m/z*	Found *m/z*
$C_{21}H_{29}N_2$	309.2325	309.2325

^{1}H NMR spectrum of compound **45** ($CDCl_3$)

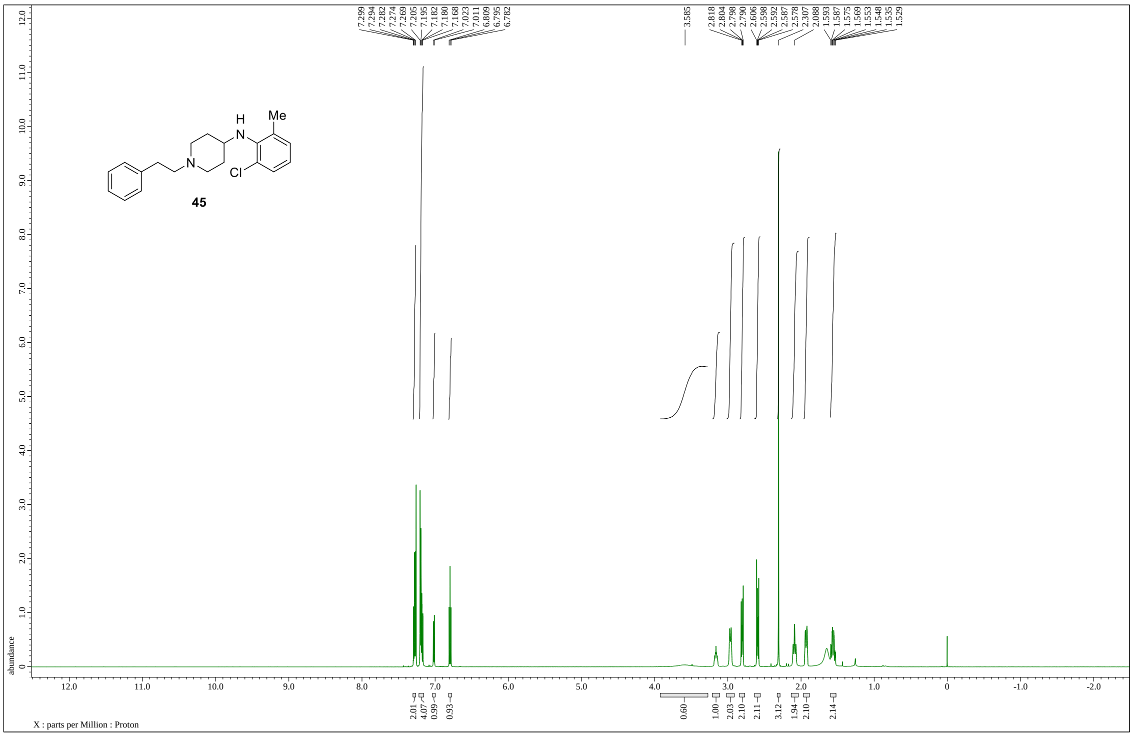

^{13}C NMR spectrum of compound **45** ($CDCl_3$)

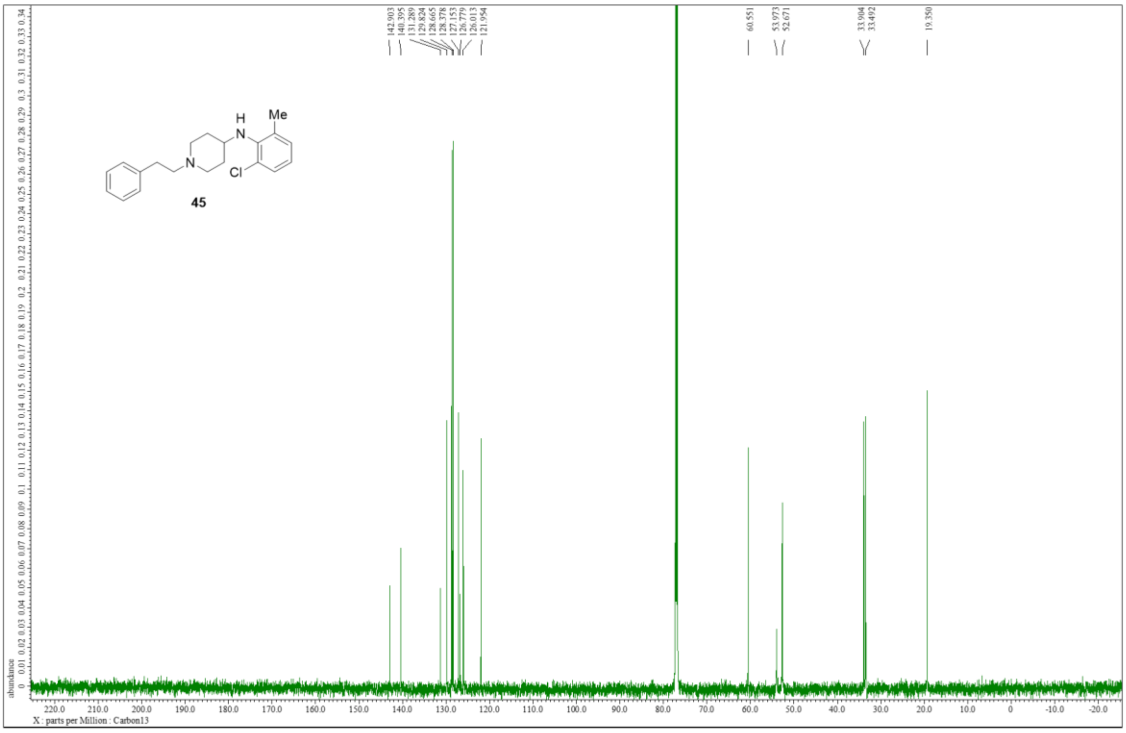

HRMS of compound **45**

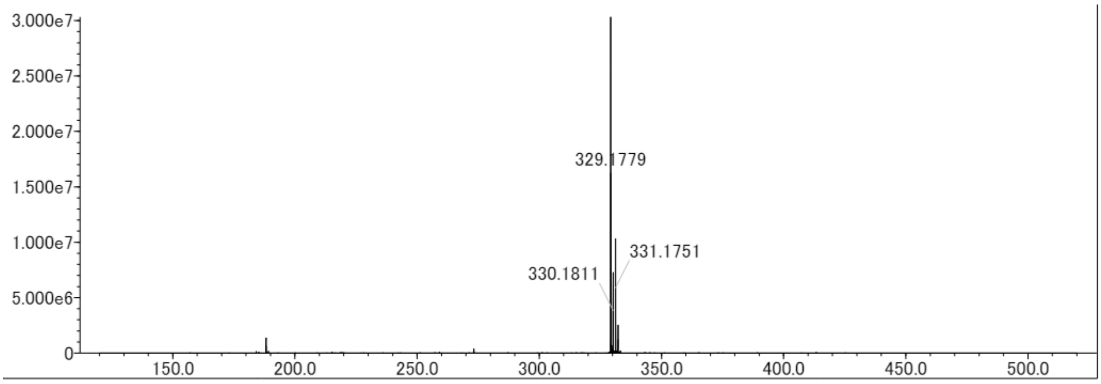

Formula $[M+H]^+$	Theoretical *m/z*	Found *m/z*
$C_{20}H_{26}ClN_2$	329.1779	329.1779

^{1}H NMR spectrum of compound **46** ($CDCl_3$)

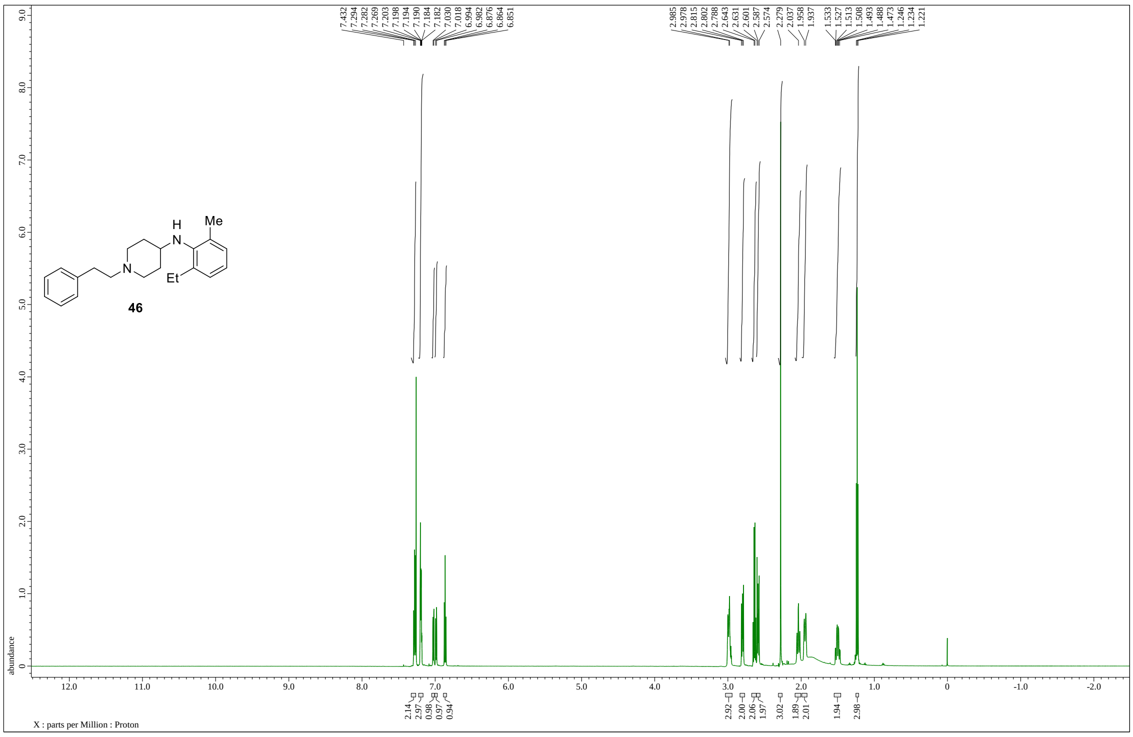

^{13}C NMR spectrum of compound **46** ($CDCl_3$)

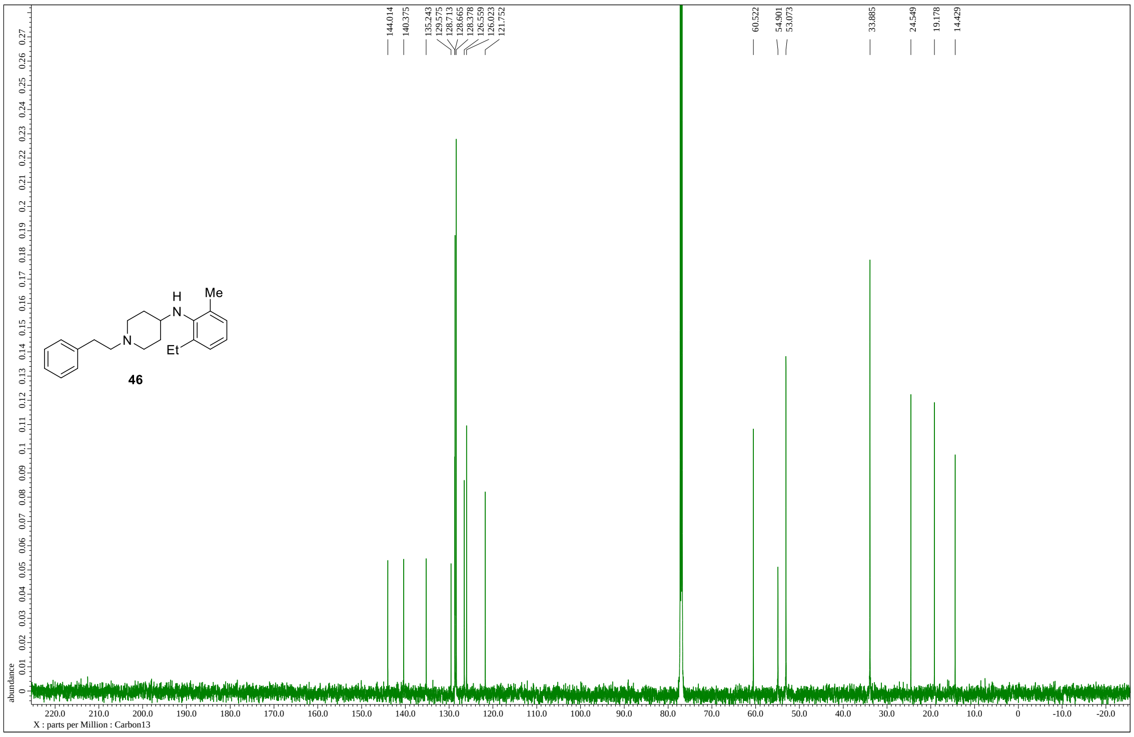

HRMS of compound **46**

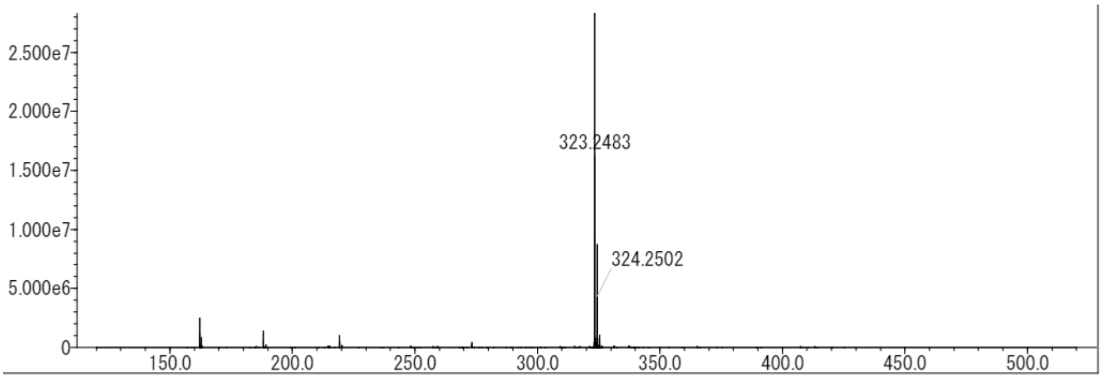

Formula [M+H]+	Theoretical *m/z*	Found *m/z*
$C_{22}H_{31}N_2$	323.2482	323.2483

^{1}H NMR spectrum of compound **47** ($CDCl_3$)

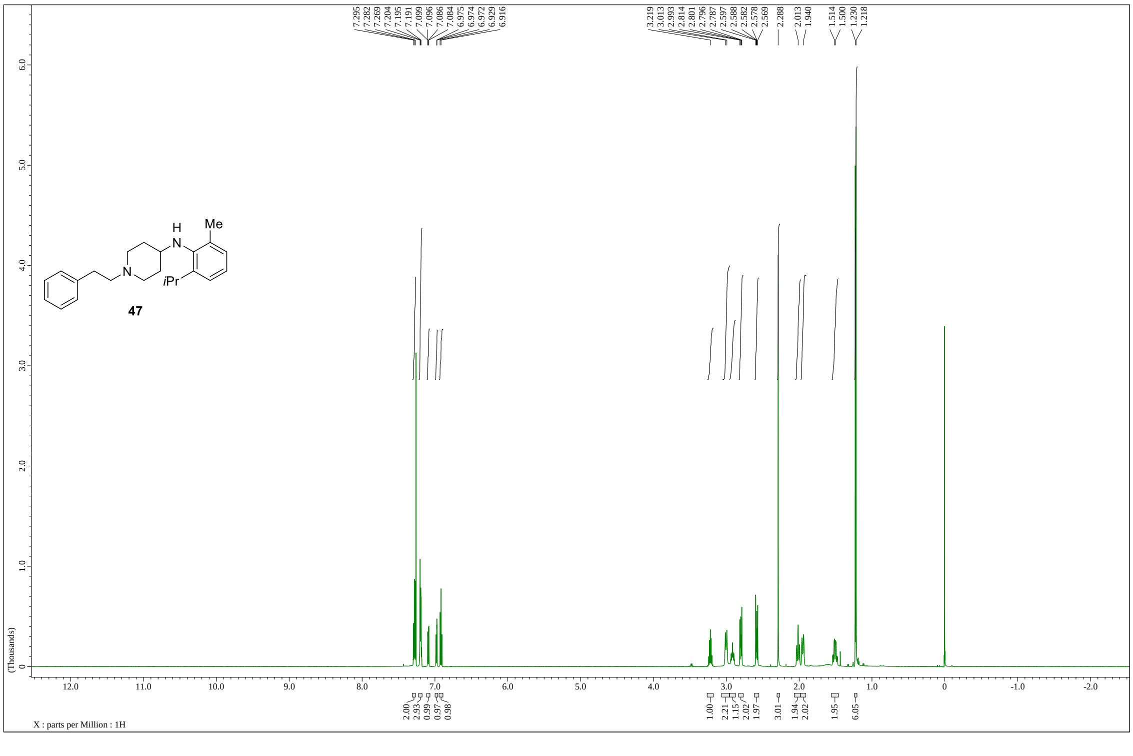

^{13}C NMR spectrum of compound **47** ($CDCl_3$)

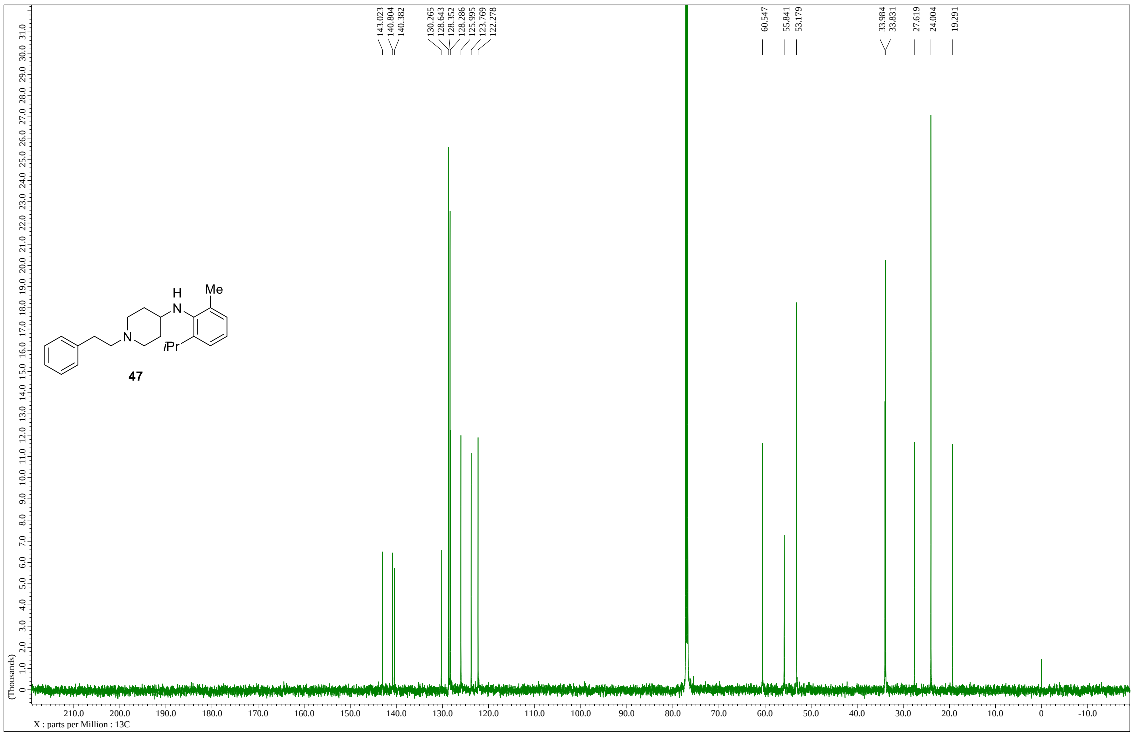

HRMS of compound **47**

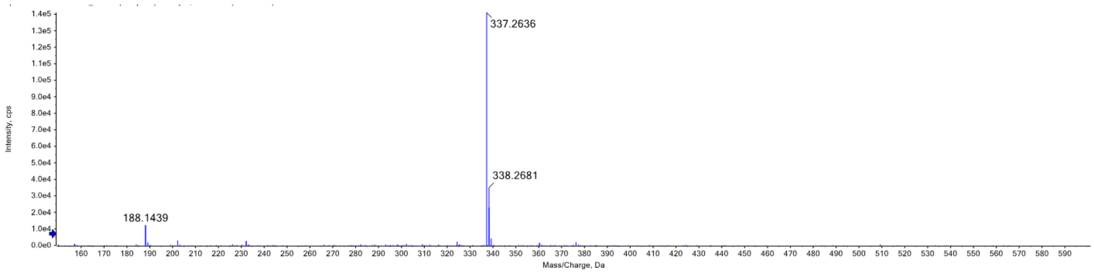

Formula [M+H]+	Theoretical *m/z*	Found *m/z*
$C_{23}H_{33}N_2$	337.2638	337.2636

^1H NMR spectrum of compound **48** ($CDCl_3$)

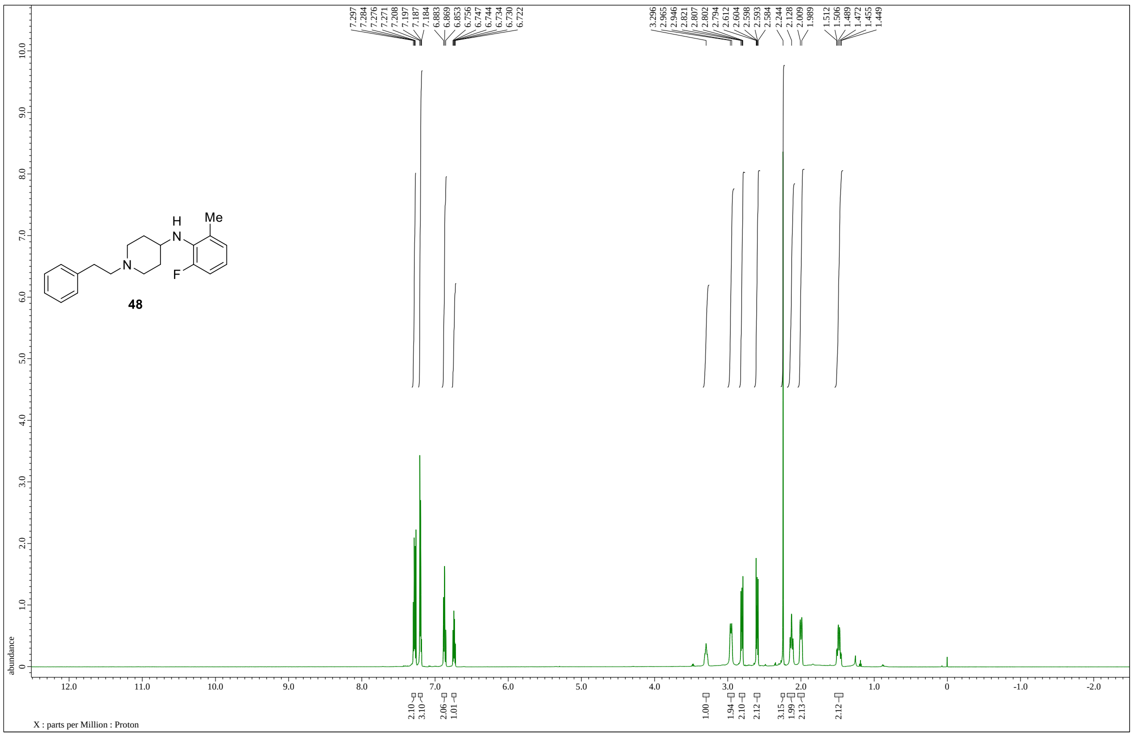

^{13}C NMR spectrum of compound **48** ($CDCl_3$)

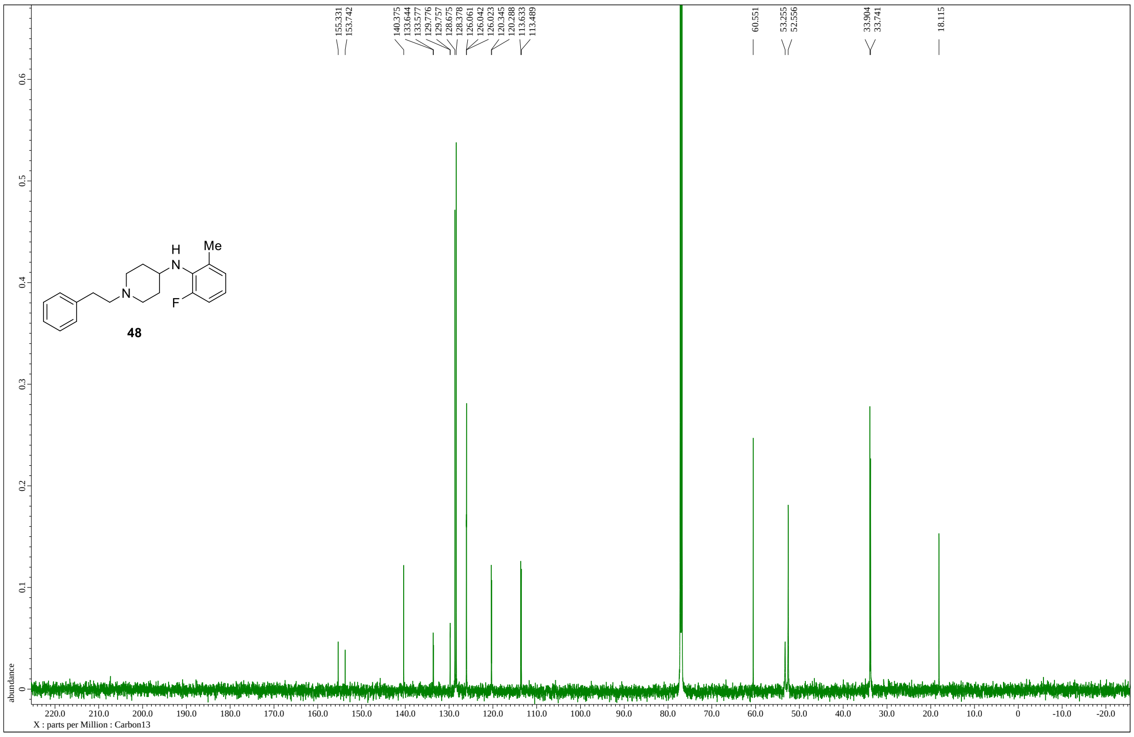

HRMS of compound **48**

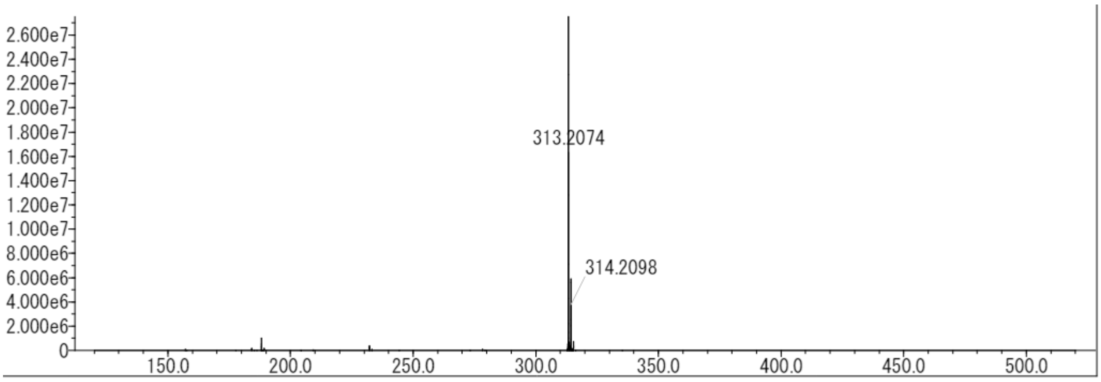

Formula [M+H]+	Theoretical *m/z*	Found *m/z*
$C_{20}H_{26}FN_2$	313.2075	313.2074

^{1}H NMR spectrum of compound **49** ($CDCl_3$)

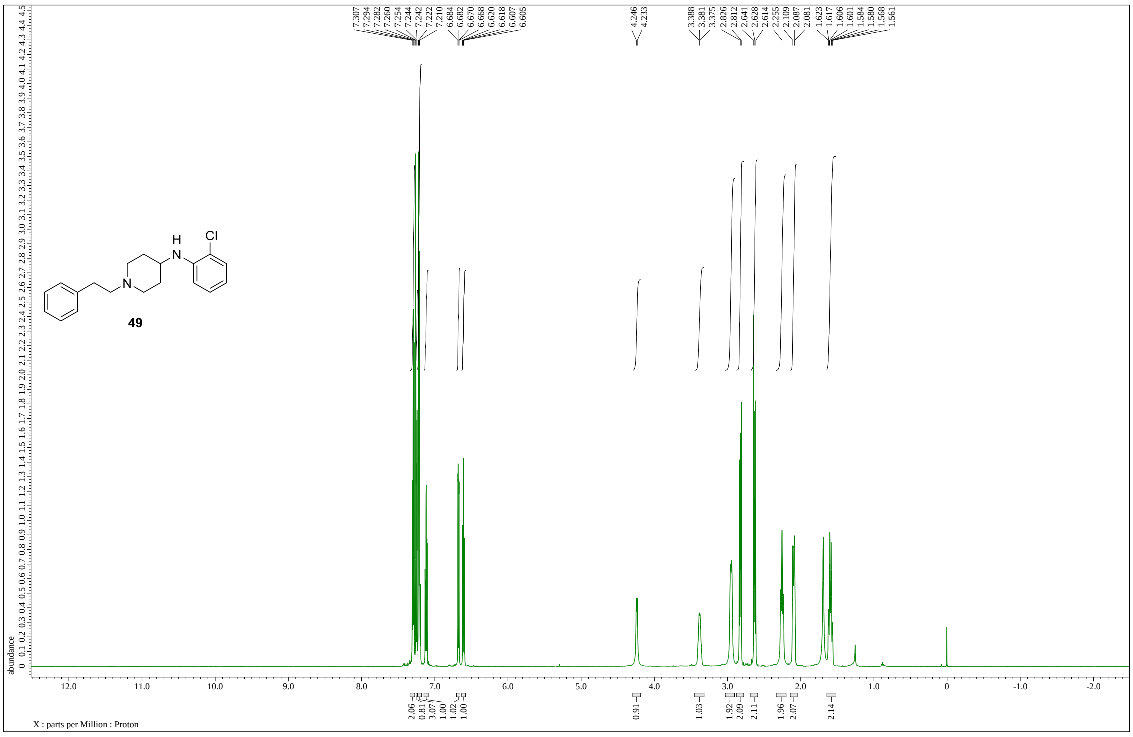

^{13}C NMR spectrum of compound **49** ($CDCl_3$)

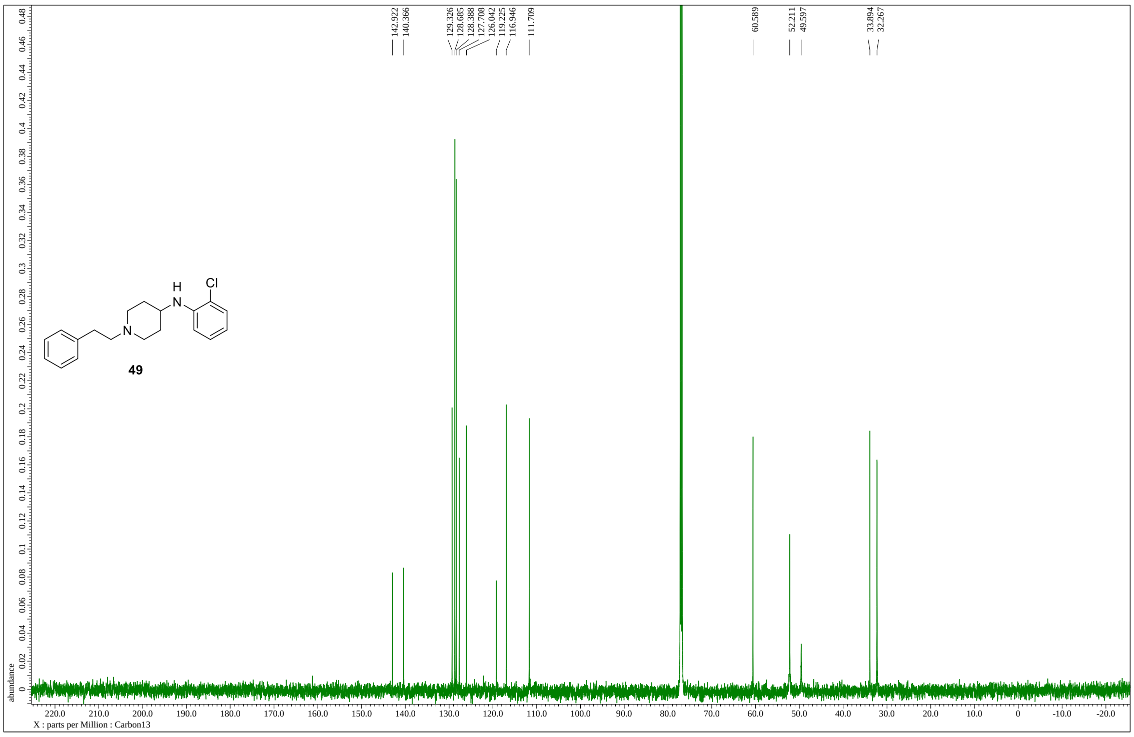

HRMS of compound **49**

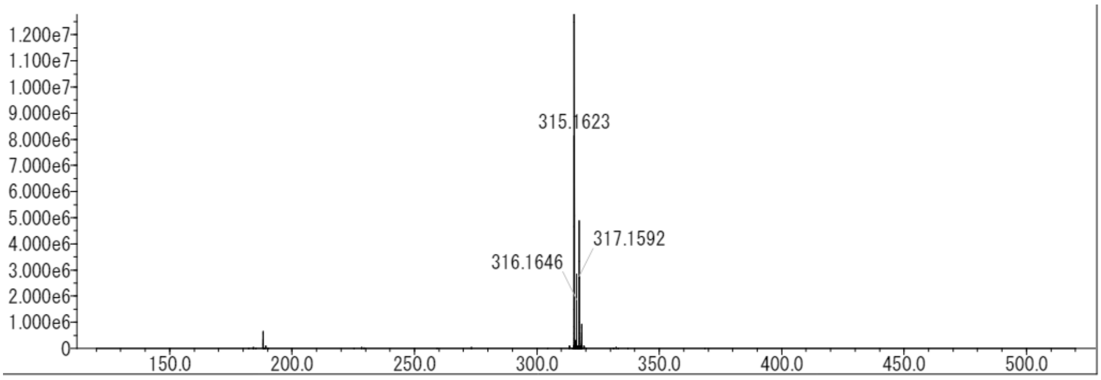

Formula $[M+H]^+$	Theoretical *m/z*	Found *m/z*
$C_{19}H_{24}ClN_2$	315.1623	315.1623

^{1}H NMR spectrum of compound **50** ($CDCl_3$)

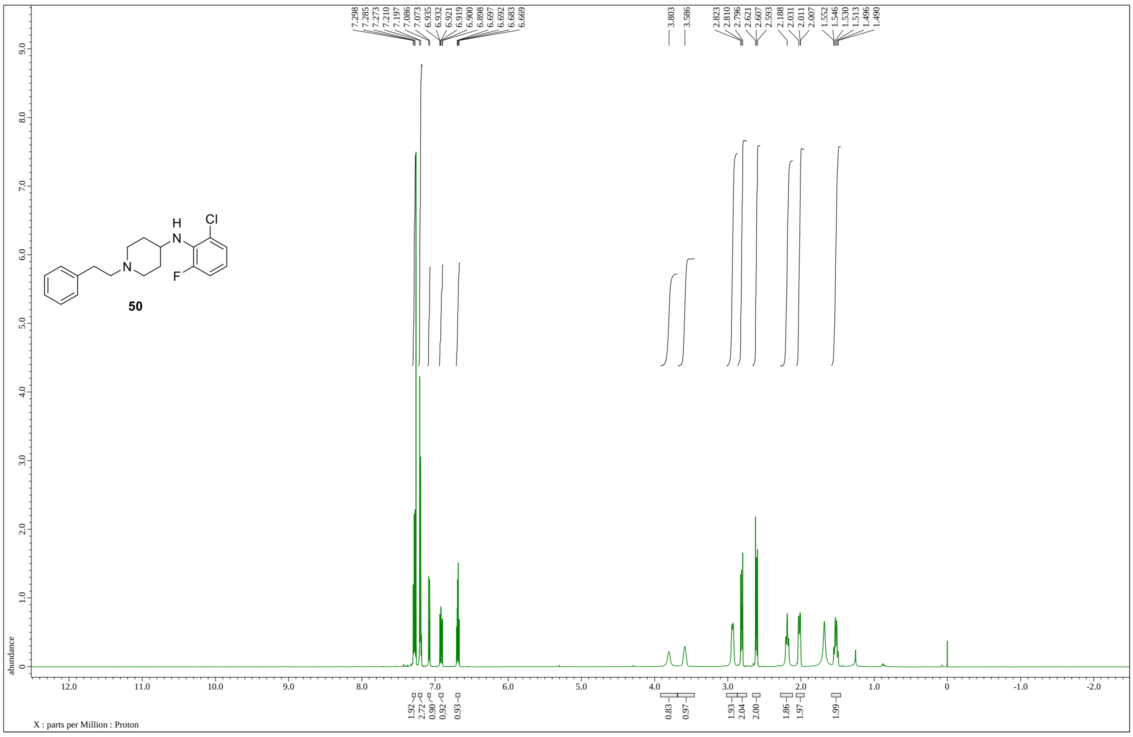

^{13}C NMR spectrum of compound **50** ($CDCl_3$)

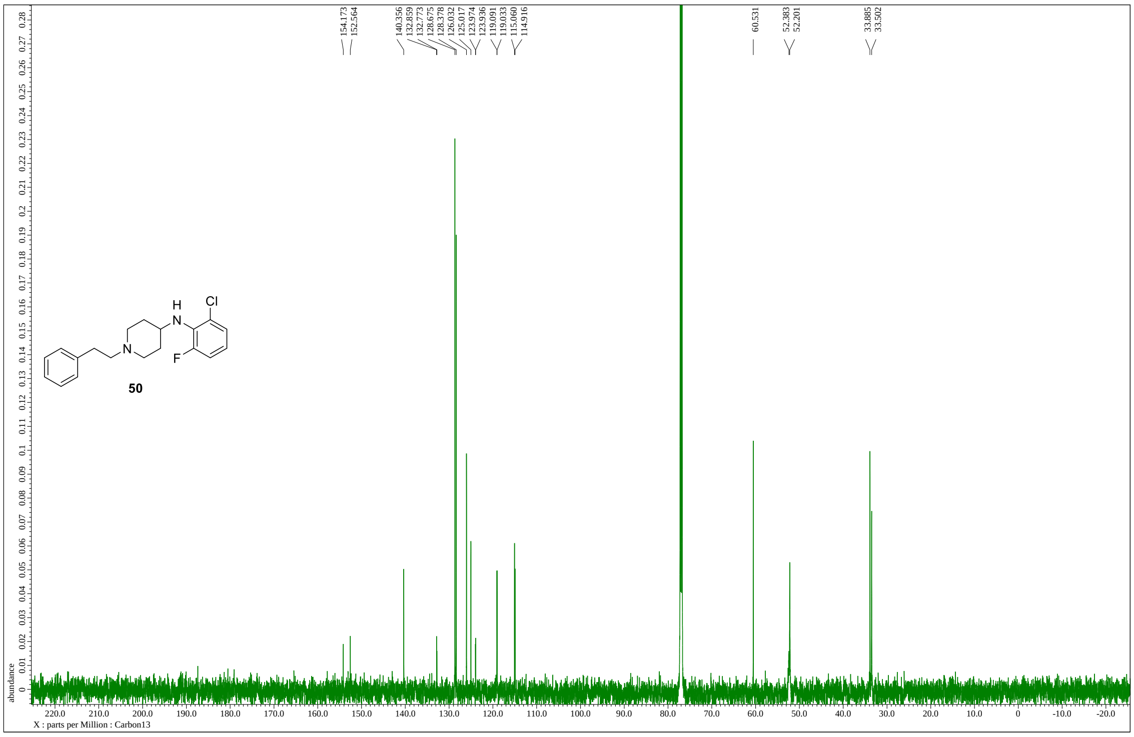

HRMS of compound **50**

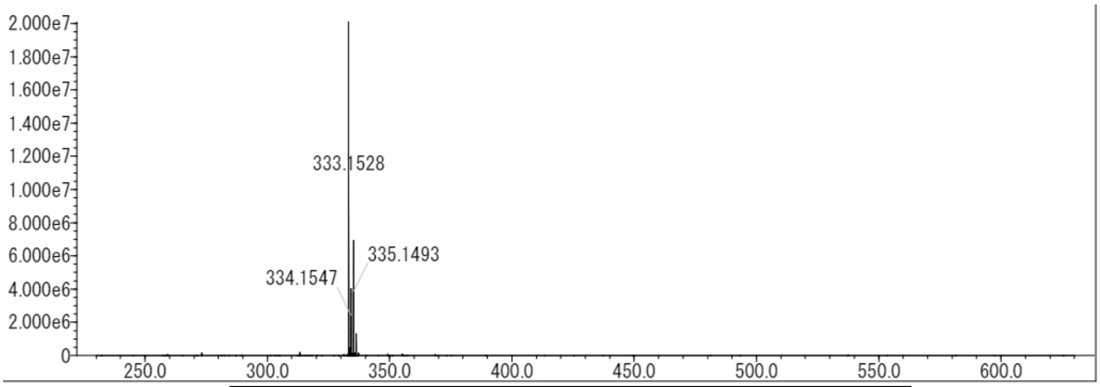

Formula $[M+H]^+$	Theoretical *m/z*	Found *m/z*
$C_{19}H_{23}ClFN_2$	333.1528	333.1528

10. HPLC chromatograms for target compounds

HPLC for compound **1**

The purity of **1** was 99.44%.

HPLC conditions:

Column: CHIRALPAK® IG, Eluent: 30% EtOH in Hex, Flow rate: 0.5 mL/min, Temp: 25 °C,

Wavelength: 270 nm

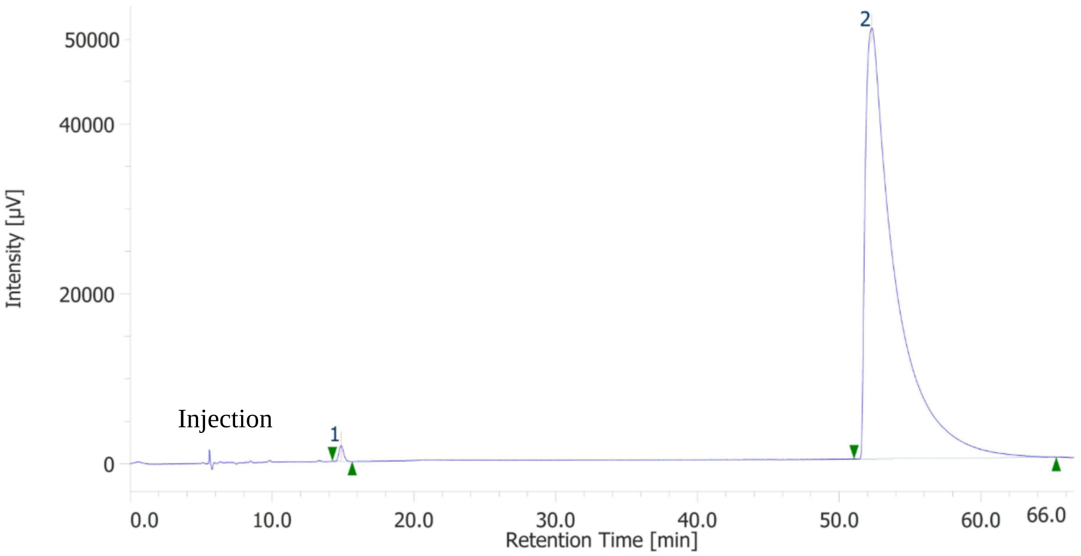

Peak No.	Retention Time (min)	Area (µV·sec)	Height (µV)	Area %
1	14.873	41695	1832	0.559
2	52.285	7423375	50712	99.441
Total				100.000

HPLC for compound **2**

The purity of **2** was 95.87%.

HPLC conditions:

Column: CHIRALPAK® IG, Eluent: 30% EtOH in Hex, Flow rate: 0.5 mL/min, Temp: 25 ˚C,

Wavelength: 210 nm

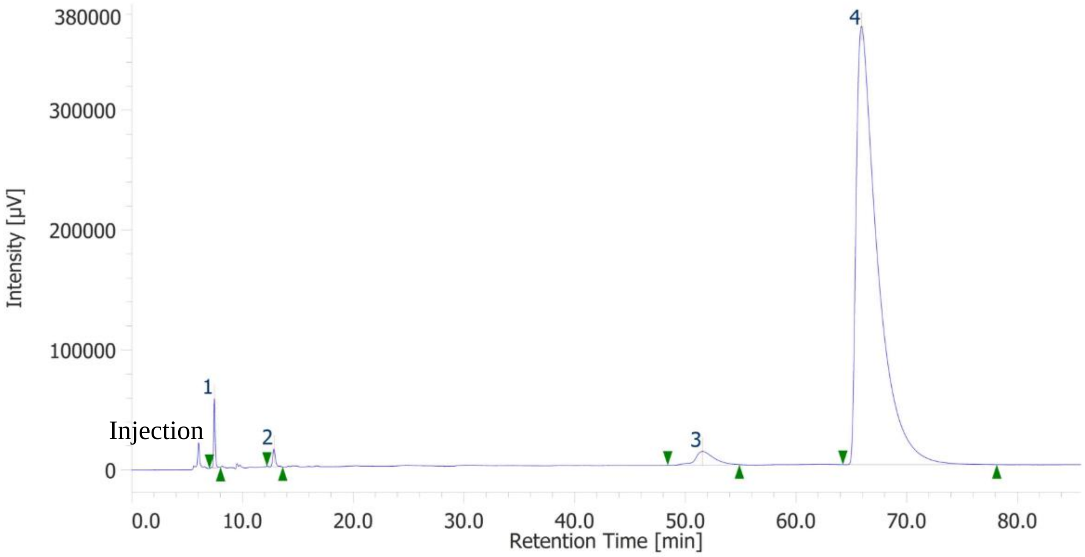

Peak No.	Retention Time (min)	Area (µV·sec)	Height (µV)	Area %
1	7.458	548706	58046	1.072
2	12.837	243976	14613	0.477
3	51.537	1318741	11297	2.577
4	65.905	49060143	365267	95.874
Total				100.000

HPLC for compound **3**

The purity of **3** was 96.27%.

HPLC conditions:

Column: CHIRALPAK® IG, Eluent: 30% EtOH in Hex, Flow rate: 0.5 mL/min, Temp: 25 ˚C,

Wavelength: 210 nm

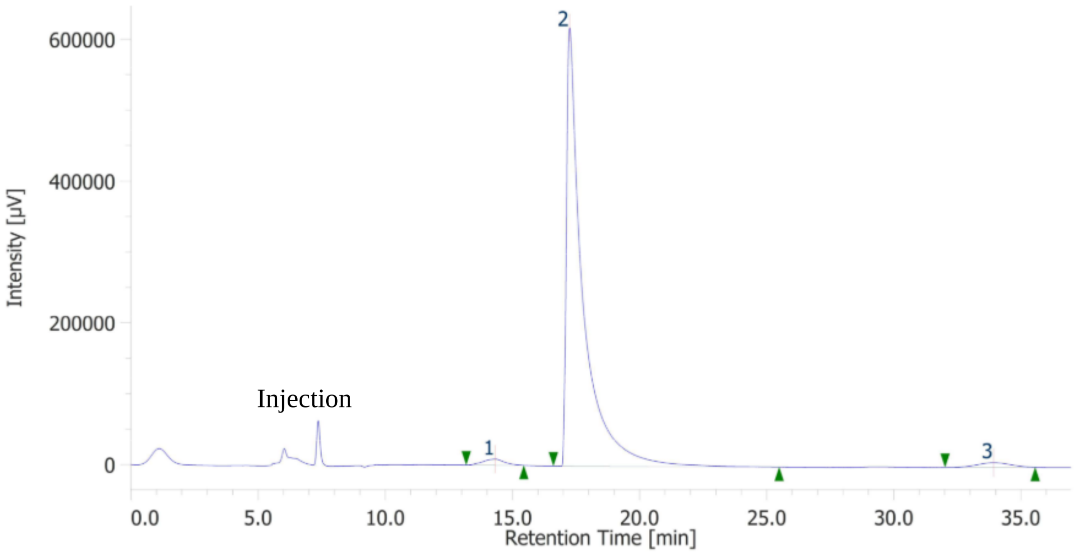

Peak No.	Retention Time (min)	Area (µV·sec)	Height (µV)	Area%
1	14.328	496280	8486	1.717
2	17.247	27833659	618173	96.270
3	33.920	582059	6470	2.013
Total				100.000

HPLC for compound **4**

The purity of **4** was 98.15%.

HPLC conditions:

Column: CHIRALPAK® IG, Eluent: 30% EtOH in Hex, Flow rate: 0.5 mL/min, Temp: 25 ˚C,

Wavelength: 210 nm

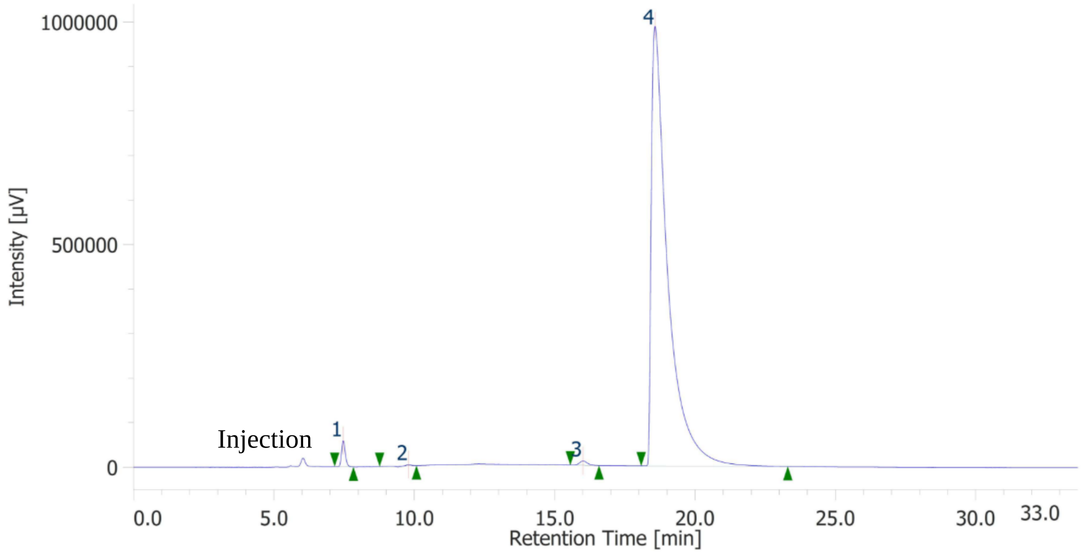

Peak No.	Retention Time (min)	Area (µV·sec)	Height (µV)	Area %
1	7.468	539771	58401	1.320
2	9.797	29418	2636	0.072
3	16.003	186252	9665	0.455
4	18.572	40141532	987359	98.153
Total				100.000

HPLC for compound **5**

The purity of **5** was 98.50%.

HPLC conditions:

Column: CHIRALPAK® IG, Eluent: 30% EtOH in Hex, Flow rate: 0.5 mL/min, Temp: 25 ˚C,

Wavelength: 270 nm

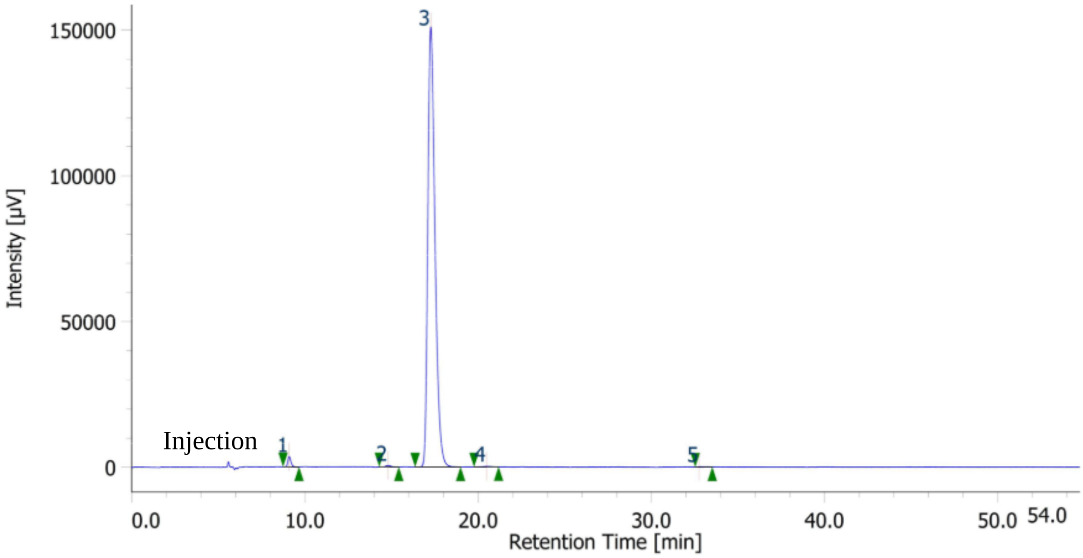

Peak No.	Retention Time (min)	Area (μV·sec)	Height (μV)	Area %
1	9.092	42061	3483	0.955
2	14.803	10292	494	0.234
3	17.260	4339361	150916	98.503
4	20.483	9056	238	0.206
5	32.755	4523	167	0.103
Total				100.000

HPLC for compound **6**

The purity of **6** was 99.82%. (Peaks 2 and 3 are enantiomers of compound **6**.)

HPLC conditions:

Column: CHIRALPAK® IE, Eluent: 20% EtOH in Hex, Flow rate: 0.5 mL/min, Temp: 25 °C,

Wavelength: 210 nm

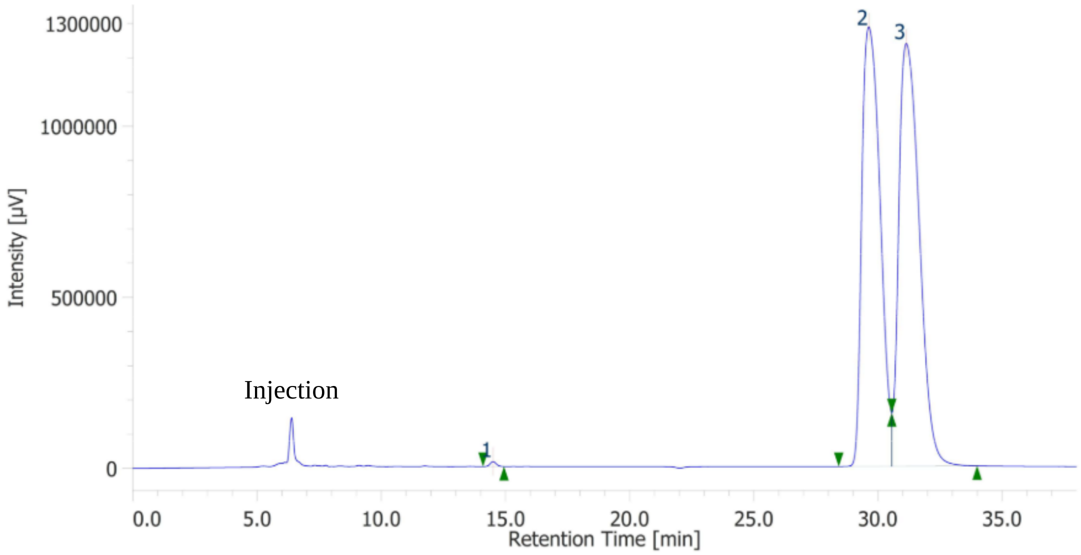

Peak No.	Retention Time (min)	Area (µV·sec)	Height (µV)	Area %
1	14.495	242406	14141	0.177
2	29.628	65671517	1284122	47.954
3	31.135	71034247	1235298	51.869
Total				100.000

HPLC for compound **7**

The purity of **7** was 95.00%. (Peak 3 is enantiomers of compound **7**.)

HPLC conditions:

Column: CHIRALPAK® IG, Eluent: 30% EtOH in Hex, Flow rate: 0.5 mL/min, Temp: 25 °C,

Wavelength: 210 nm

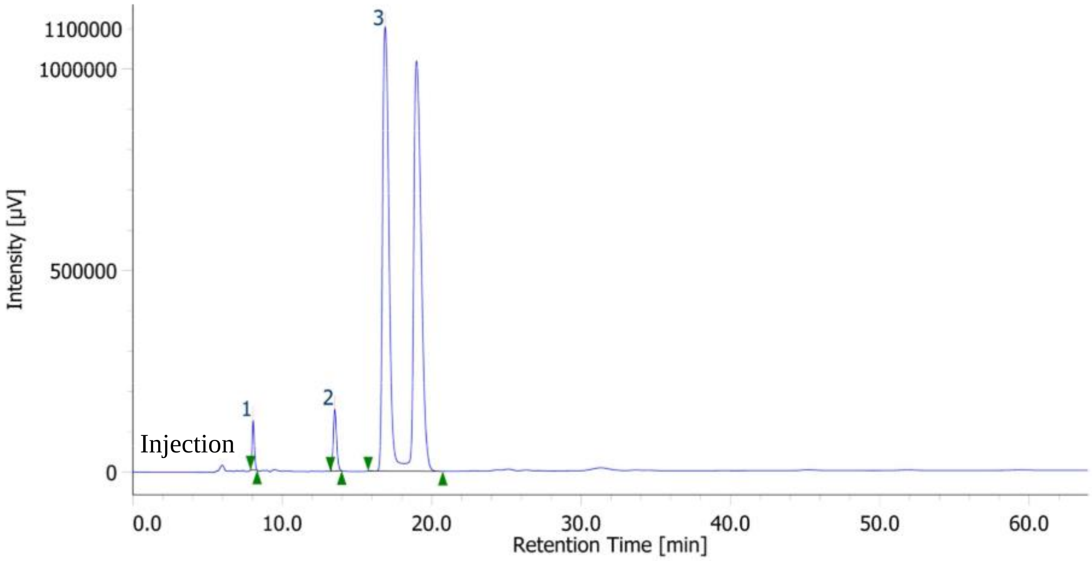

Peak No.	Retention Time (min)	Area (µV·sec)	Height (µV)	Area %
1	8.047	1245892	122883	1.734
2	13.510	2345500	152613	3.264
3	16.887	68265942	1101965	95.002
Total				100.000

HPLC for compound **8**

The purity of **8** was 97.97%. (Peaks 2 and 3 are enantiomers of compound **8**.)

HPLC conditions:

Column: CHIRALPAK® IG, Eluent: 20% IPA in Hex, Flow rate: 0.5 mL/min, Temp: 25 ˚C,

Wavelength: 210 nm

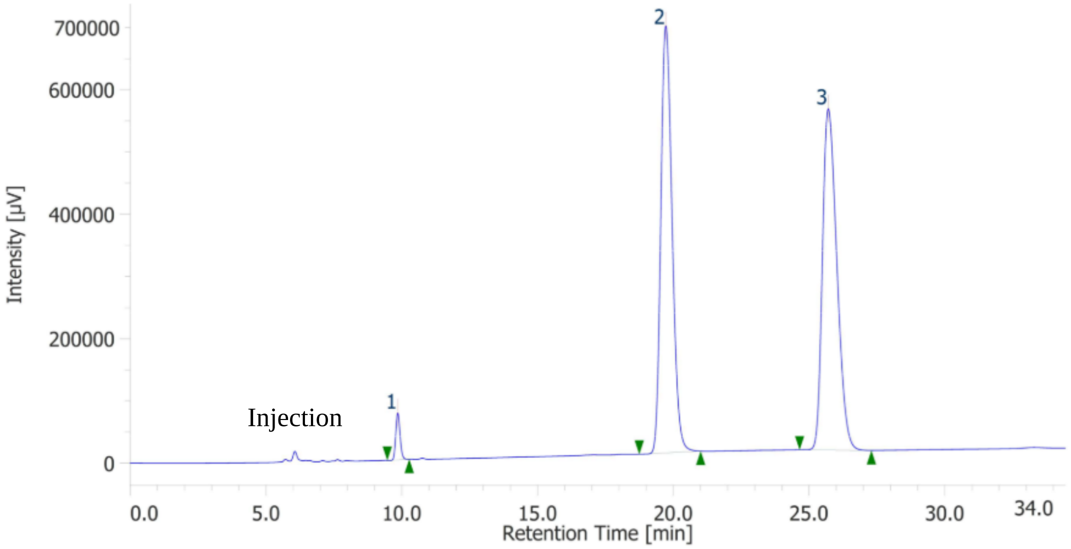

Peak No.	Retention Time (min)	Area (µV·sec)	Height (µV)	Area %
1	9.853	828604	75383	2.031
2	19.722	19772855	686208	48.470
3	25.707	20192217	548566	49.498
Total				100.000

HPLC for compound (+)-**8**

The purity of (+)-**8** was 95.11%.

HPLC conditions:

Column: CHIRALPAK® IG, Eluent: 20% IPA in Hex, Flow rate: 0.5 mL/min, Temp: 25 ˚C,

Wavelength: 210 nm

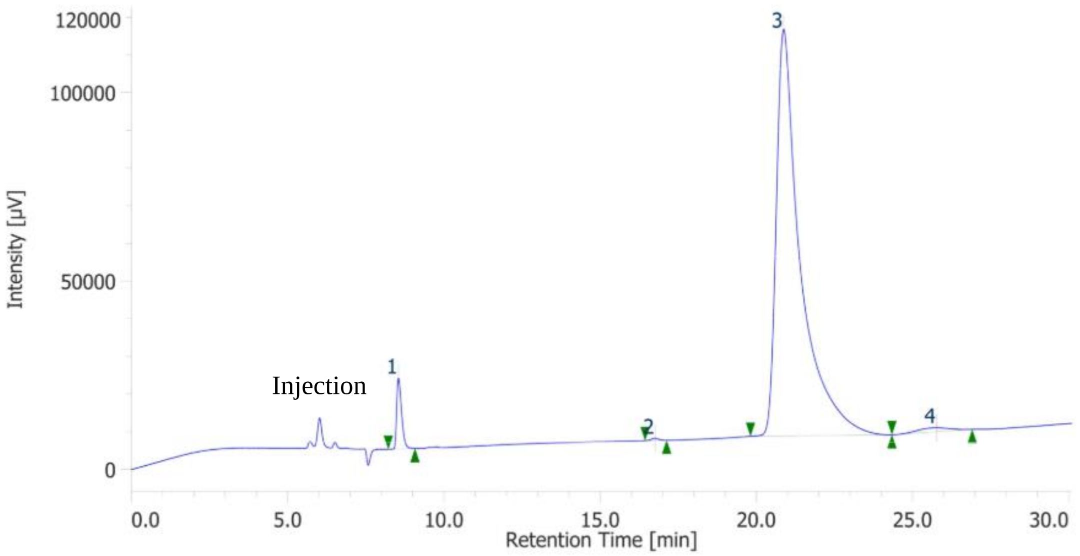

Peak No.	Retention Time (min)	Area (µV·sec)	Height (µV)	Area %
1	8.545	206096	18877	1.776
2	16.755	10218	600	0.168
3	20.870	5775633	107879	95.152
4	25.762	77966	1090	1.284
Total				100.000

HPLC for compound (−)-**8**

The purity of (−)-**8** was 95.11%.

HPLC conditions:

Column: CHIRALPAK® IA, Eluent: 10% IPA in Hex, Flow rate: 0.5 mL/min, Temp: 25 ˚C,

Wavelength: 210 nm

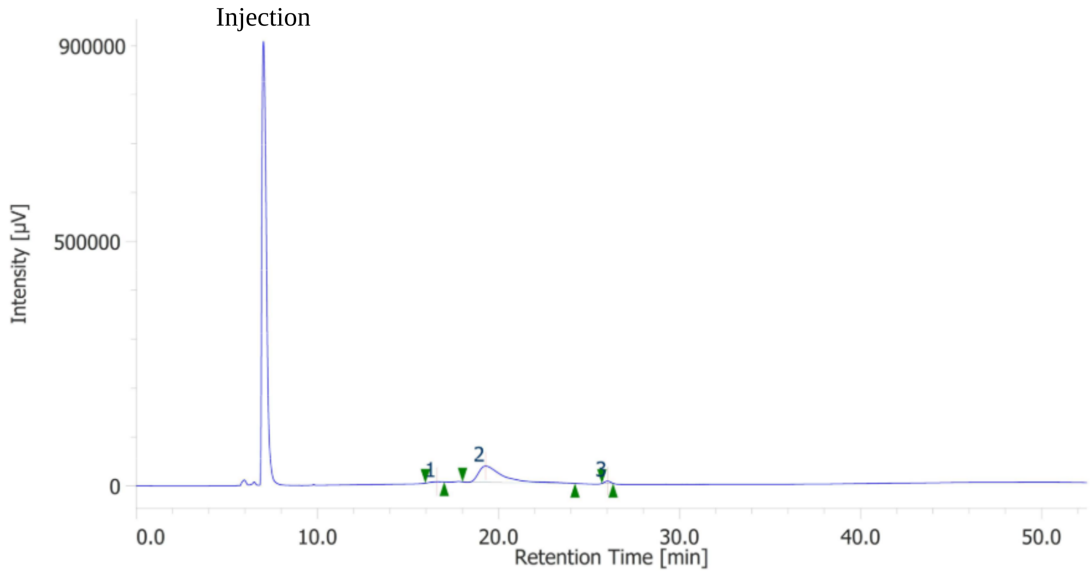

Peak No.	Retention Time (min)	Area (µV·sec)	Height (µV)	Area %
1	16.595	56190	1451	1.776
2	19.285	3008902	32711	95.114
3	26.023	98379	4602	3.110
Total				100.000

HPLC for compound **9**

The purity of **9** was 99.74%. (Peaks 4 and 5 are enantiomers of compound **9**.)

HPLC conditions:

Column: CHIRALPAK® IF, Eluent: 5% EtOH in Hex, Flow rate: 0.5 mL/min, Temp: 25 ˚C,

Wavelength: 210 nm

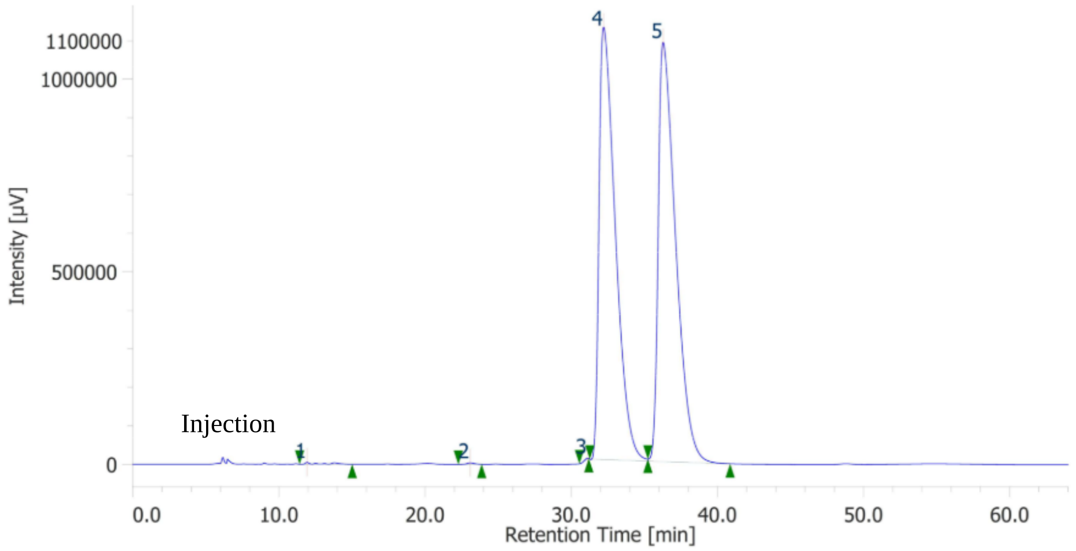

Peak No.	Retention Time (min)	Area (µV·sec)	Height (µV)	Area %
1	11.928	284049	4695	0.160
2	23.085	141182	3961	0.079
3	31.118	42324	2260	0.024
4	32.210	87191339	1122286	49.093
5	36.287	89945852	1088264	50.644
Total				100.000

HPLC for compound **10**

The purity of **10** was 99.64%. (Peaks 1 and 2 are enantiomers of compound **10**.)

HPLC conditions:

Column: CHIRALPAK® IG, Eluent: 30% EtOH in Hex, Flow rate: 0.5 mL/min, Temp: 25 °C,

Wavelength: 270 nm

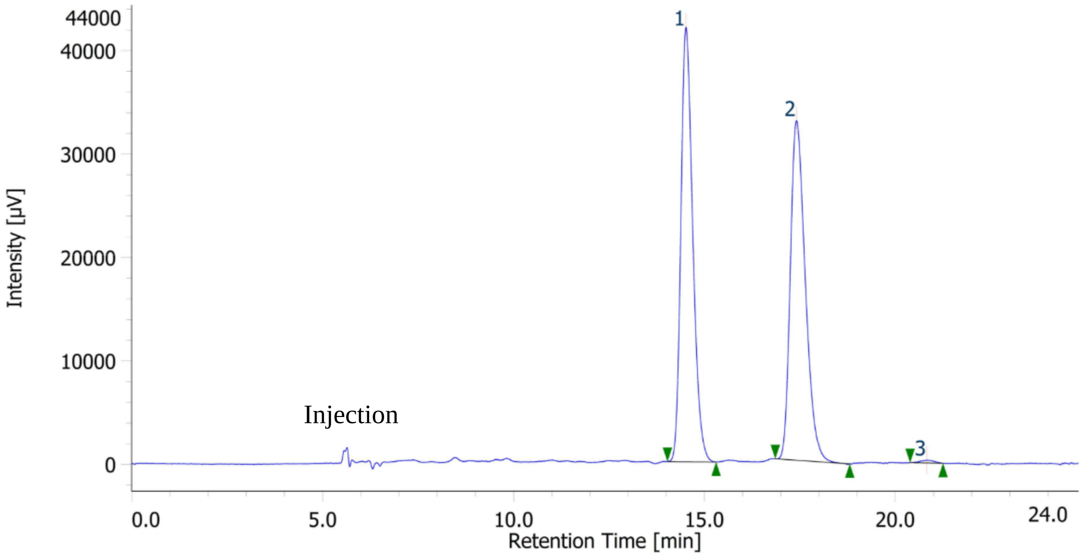

Peak No.	Retention Time (min)	Area (µV·sec)	Height (µV)	Area %
1	14.515	906869	42022	50.165
2	17.407	894301	32849	49.470
3	20.822	6598	284	0.365
Total				100.000

HPLC for compound **13**

The purity of **13** was 96.66%.

HPLC conditions:

Column: CHIRALPAK® IG, Eluent: 30% EtOH in Hex, Flow rate: 0.5 mL/min, Temp: 25 °C,

Wavelength: 210 nm

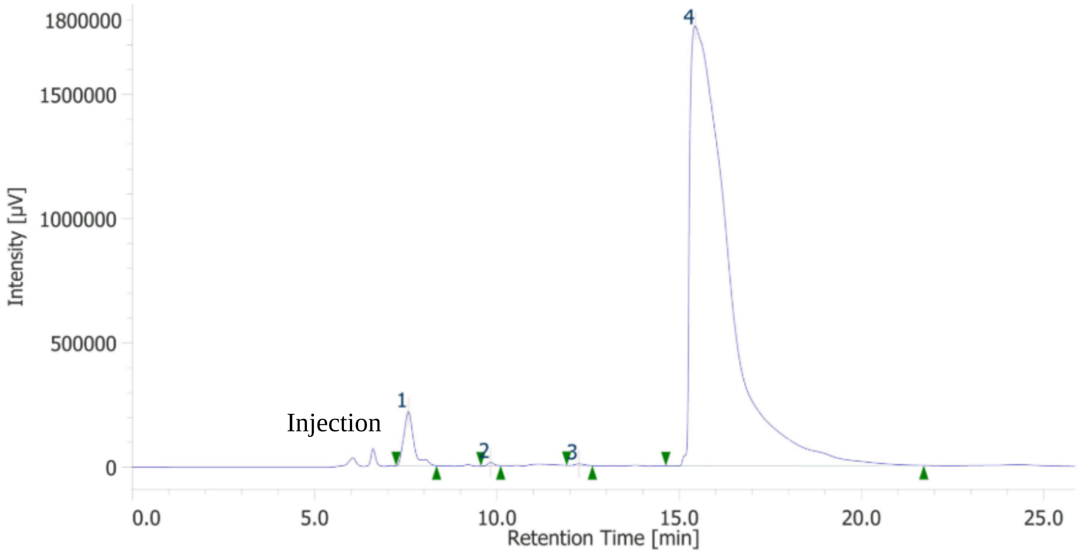

Peak No.	Retention Time (min)	Area (µV·sec)	Height (µV)	Area %
1	7.572	4183854	217592	3.129
2	9.823	159249	13361	0.119
3	12.240	119757	6916	0.090
4	15.442	129229155	1770132	96.662
Total				100.000

HPLC for compound **15**

The purity of **15** was 99.86%.

HPLC conditions:

Column: CHIRALPAK® IG, Eluent: 30% EtOH in Hex, Flow rate: 0.5 mL/min, Temp: 25 °C,

Wavelength: 270 nm

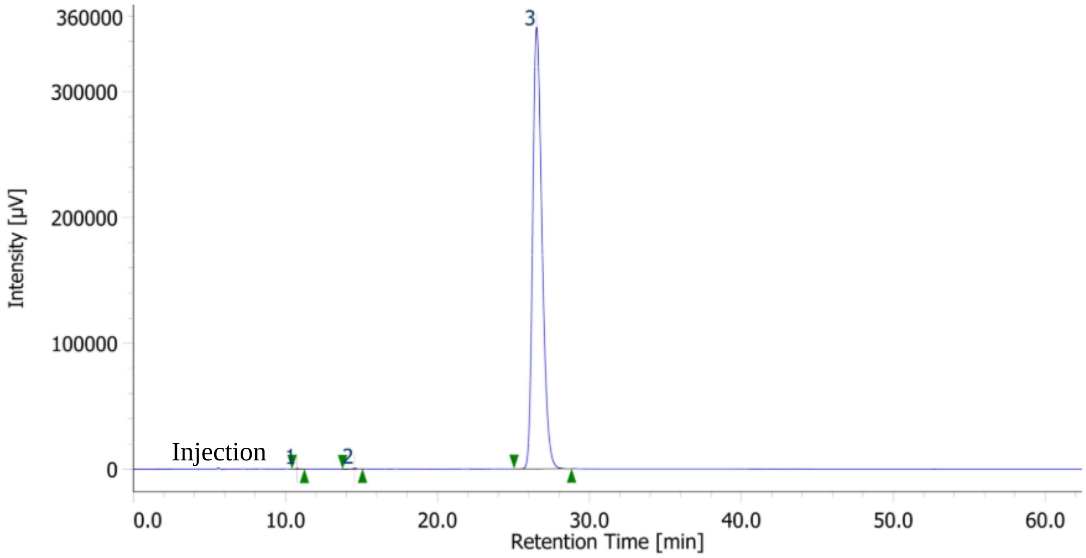

Peak No.	Retention Time (min)	Area (µV·sec)	Height (µV)	Area %
1	10.760	5711	427	0.038
2	14.542	14828	552	0.099
3	26.518	14958447	350990	99.863
Total				100.000

HPLC for compound **16**

The purity of **16** was 98.43%.

HPLC conditions:

Column: CHIRALPAK® IG, Eluent: 30% EtOH in Hex, Flow rate: 0.5 mL/min, Temp: 25 °C,

Wavelength: 210 nm

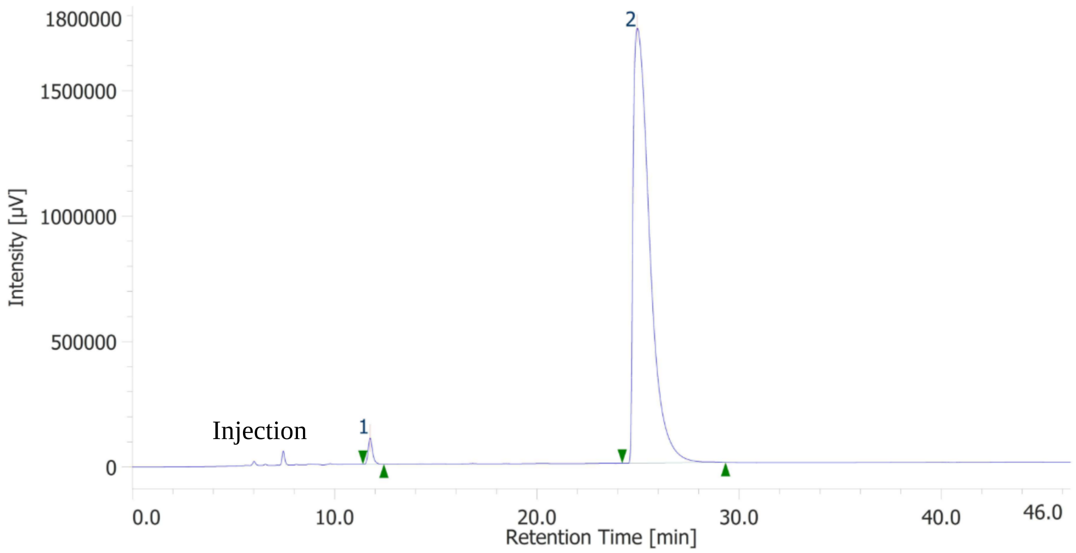

Peak No.	Retention Time (min)	Area (µV·sec)	Height (µV)	Area %
1	11.755	1575412	104218	1.573
2	24.952	98608011	1733830	98.427
Total				100.000

HPLC for compound **21**

The purity of **21** was 99.42% (Peaks 3 and 4 are diastereomers of compound **21**.)

HPLC conditions:

Column: CHIRALPAK® IG, Eluent: 30% EtOH in Hex, Flow rate: 0.5 mL/min, Temp: 25 °C,

Wavelength: 210 nm

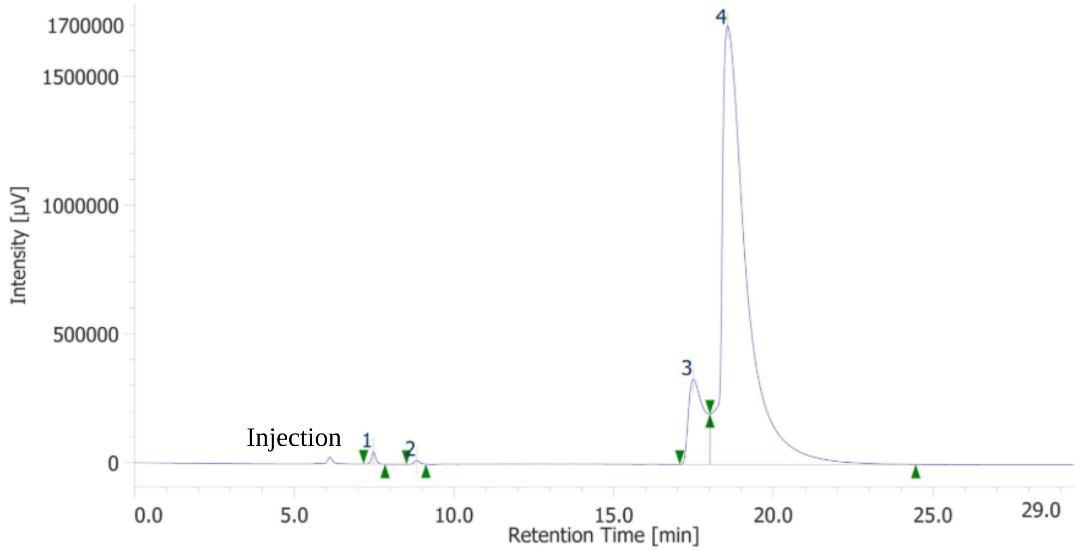

Peak No.	Retention Time (min)	Area (µV·sec)	Height (µV)	Area %
1	7.480	431132	46867	0.419
2	8.837	161916	14019	0.157
3	17.493	11276148	330424	10.946
4	18.567	91148365	1703243	88.478
Total				100.000

HPLC for compound **22**

The purity of **22** was 99.96%. (Peaks 2 and 3 are diastereomers of compound **22**.)

HPLC conditions:

Column: CHIRALPAK® IF, Eluent: 10% EtOH in Hex, Flow rate: 0.3 mL/min, Temp: 25 ˚C,

Wavelength: 210 nm

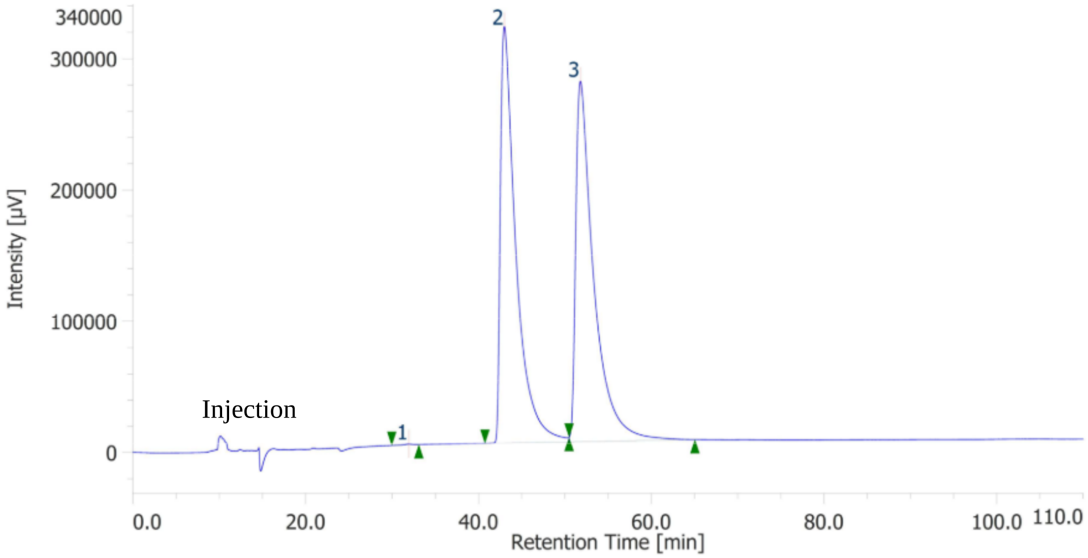

Peak No.	Retention Time (min)	Area (μV·sec)	Height (μV)	Area %
1	31.943	36050	761	0.045
2	42.988	39652949	317157	49.841
3	51.793	39869673	274331	50.114
Total				100.000

HPLC for compound **23**

The purity of **23** was 98.40%. (Peaks 6 and 7 are enantiomers of compound **23**.)

HPLC conditions

Column: CHIRALPAK® IG, Eluent: 30% EtOH in Hex, Flow rate: 0.5 mL/min, Temp: 25 °C,

Wavelength: 210 nm

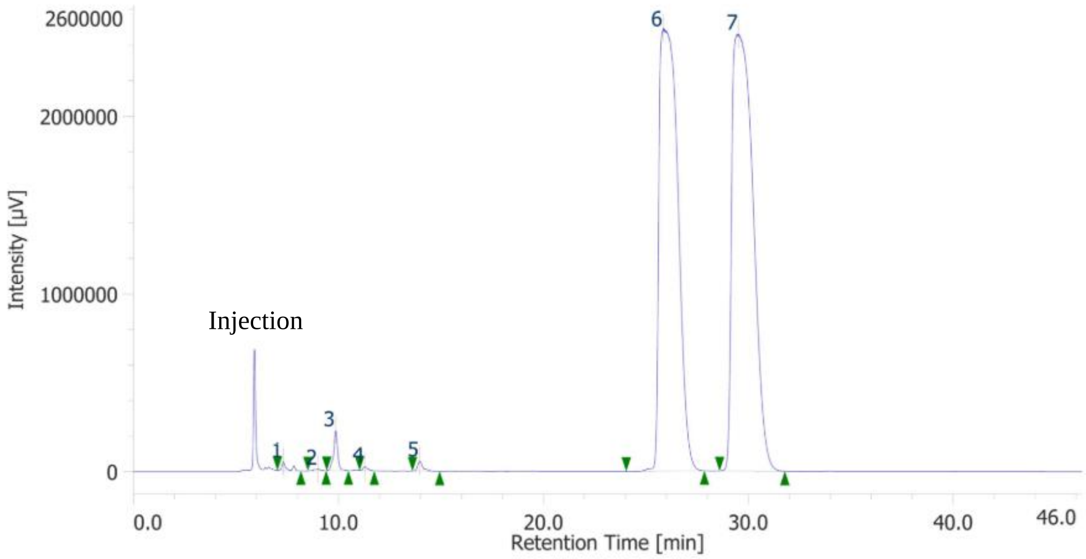

Peak No.	Retention Time (min)	Area (µV·sec)	Height (µV)	Area %
1	7.308	819275	49545	0.241
2	9.003	210369	8749	0.062
3	9.863	3126384	224574	0.919
4	11.280	332776	20784	0.098
5	13.962	966290	56047	0.284
6	25.842	158814194	2492991	46.692
7	29.518	175865104	2457736	51.705
Total				100.000

HPLC for compound (+)-**23**

The purity of (+)-**23** was 99.23%.

HPLC conditions:

Column: CHIRALPAK® IG, Eluent: 30% EtOH in Hex, Flow rate: 0.5 mL/min, Temp: 25 °C,

Wavelength: 210 nm

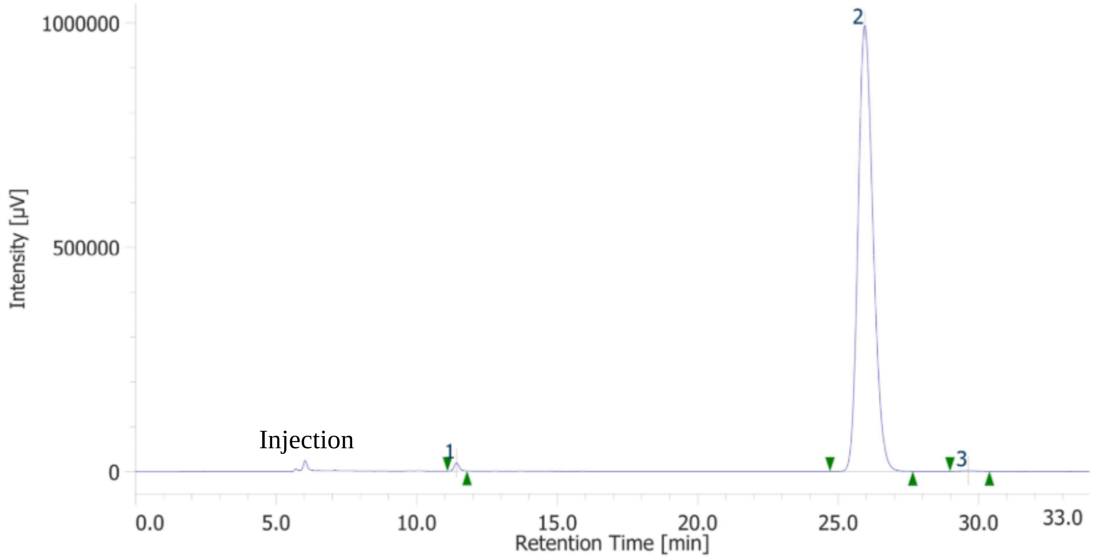

Peak No.	Retention Time (min)	Area (µV·sec)	Height (µV)	Area %
1	11.410	231946	17947	0.622
2	25.938	37004327	992539	99.227
3	29.630	56323	1449	0.151
Total				100.000

HPLC for compound (–)-**23**

The purity of (–)-**23** was 98.99%.

HPLC conditions:

Column: CHIRALPAK® IG, Eluent: 30% EtOH in Hex, Flow rate: 0.5 mL/min, Temp: 25 °C,

Wavelength: 210 nm

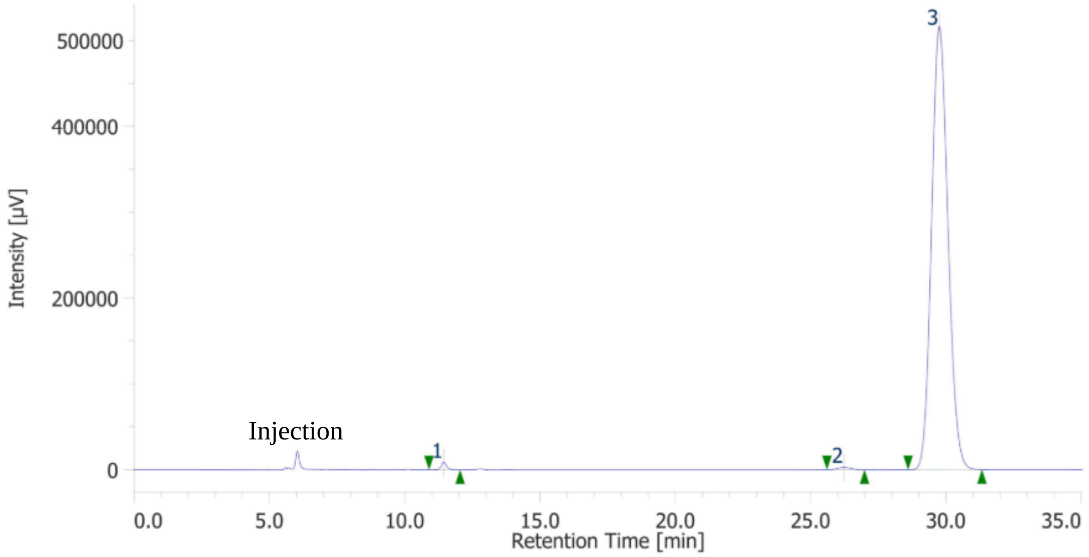

Peak No.	Retention Time (min)	Area (µV·sec)	Height (µV)	Area %
1	11.438	123053	8578	0.560
2	26.233	98099	2778	0.446
3	29.750	21761125	516119	98.994
Total				100.000

HPLC for compound **25**

The purity of **25** was 99.70%. (Peaks 3 and 4 are enantiomers of compound **25**.)

HPLC conditions:

Column: CHIRALPAK® IA, Eluent: 5% EtOH in Hex, Flow rate: 0.5 mL/min, Temp: 25 °C,

Wavelength: 210 nm

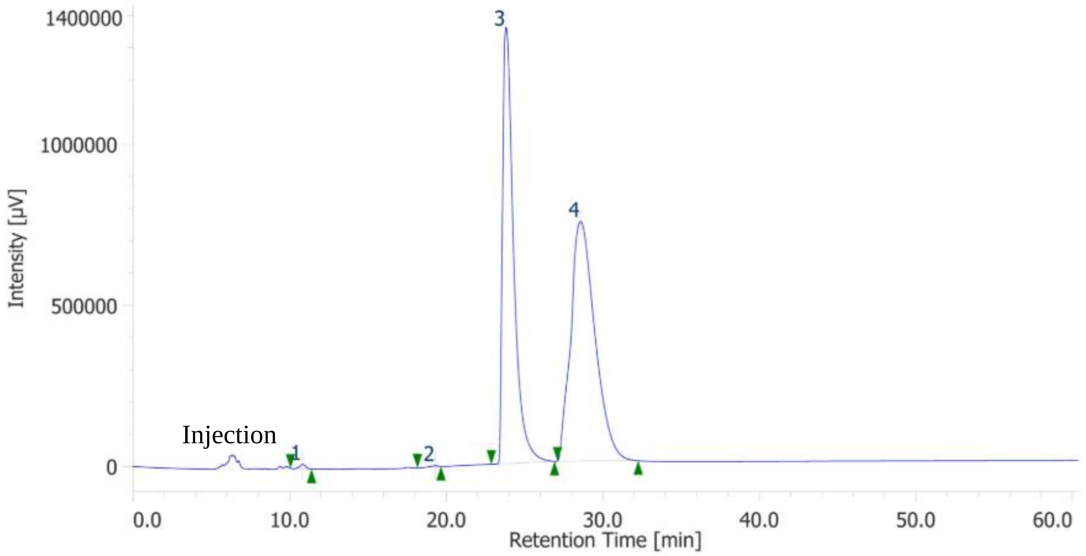

Peak No.	Retention Time (min)	Area (µV·sec)	Height (µV)	Area %
1	10.815	330901	13047	0.226
2	19.305	106006	3298	0.072
3	23.815	66469617	1353951	45.324
4	28.570	79748301	743082	54.378
Total				100.000

HPLC for compound **26**

The purity of **26** was 96.95%. (Peaks 1 and 2 are enantiomers of compound **26**.)

HPLC conditions:

Column: CHIRALPAK® IG, Eluent: 20% EtOH in Hex, Flow rate: 0.5 mL/min, Temp: 25 °C,

Wavelength: 210 nm

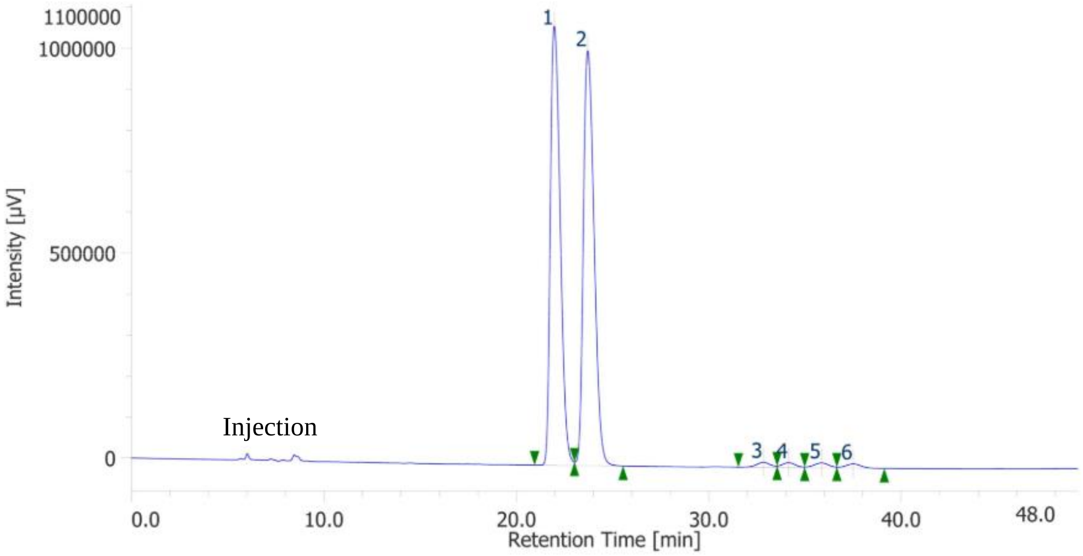

Peak No.	Retention Time (min)	Area (µV·sec)	Height (µV)	Area %
1	21.965	38233802	1071280	47.854
2	23.703	39223456	1012464	49.092
3	32.827	620505	12193	0.777
4	34.142	586348	11913	0.734
5	35.850	662282	12016	0.829
6	37.500	571058	10202	0.715
Total				100.000

HPLC for compound **27**

The purity of **27** was 99.88%. (Peaks 2 and 3 are enantiomers of compound **27**.)

HPLC conditions:

Column: CHIRALPAK® IG, Eluent: 50% IPA in Hex, Flow rate: 0.5 mL/min, Temp: 25 ˚C,

Wavelength: 210 nm

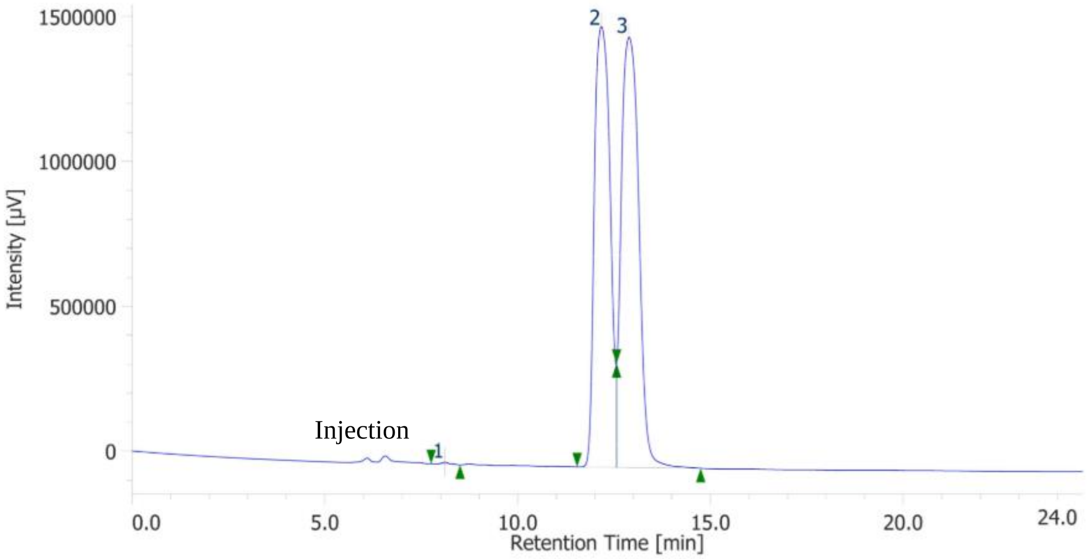

Peak No.	Retention Time (min)	Area (µV·sec)	Height (µV)	Area %
1	8.107	105736	7008	0.117
2	12.167	42821170	1519270	47.554
3	12.878	47121080	1483918	52.329
Total				100.000

HPLC for compound **28**

The purity of **28** was 99.99%. (Peaks 2 and 3 are enantiomers of compound **28**.)

HPLC conditions:

Column: CHIRALPAK® ID, Eluent: 10% IPA in Hex, Flow rate: 0.3 mL/min, Temp: 25 °C,

Wavelength: 210 nm

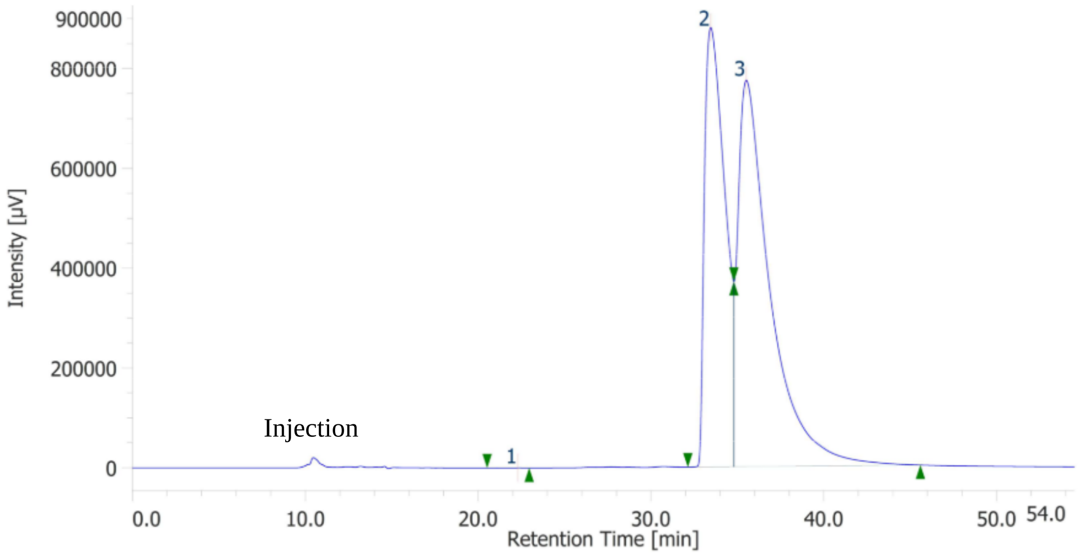

Peak No.	Retention Time (min)	Area (µV·sec)	Height (µV)	Area %
1	22.287	11184	246	0.006
2	33.457	71909381	880339	41.547
3	35.513	101158098	773736	58.446
Total				100.000

HPLC for compound **29**

The purity of **29** was 99.34%. (Peaks 3 and 4 are enantiomers of compound **29**.)

HPLC conditions:

Column: CHIRALPAK® IF, Eluent: 5% EtOH in Hex, Flow rate: 0.5 mL/min, Temp: 25 ˚C,

Wavelength: 210 nm

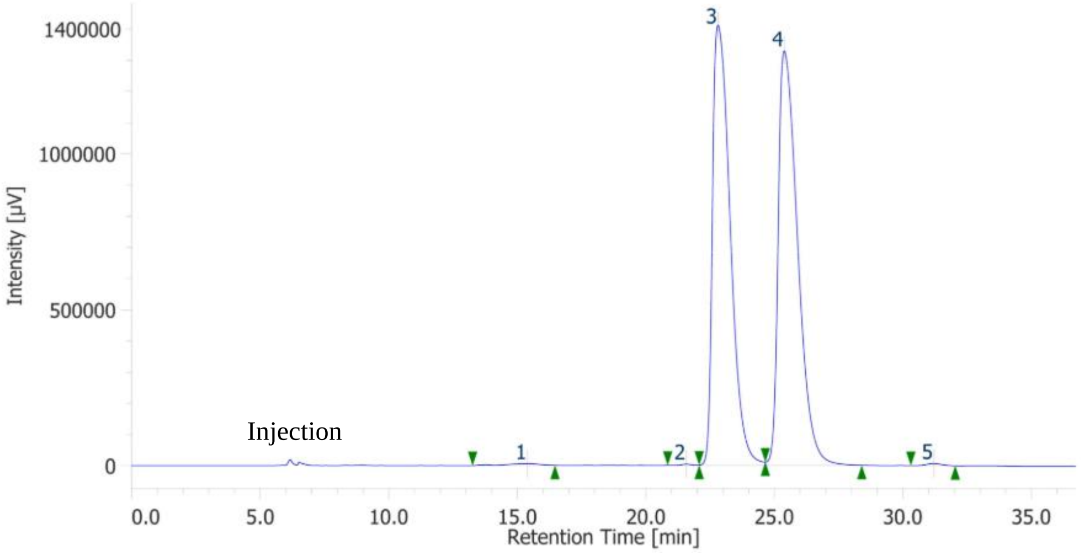

Peak No.	Retention Time (min)	Area (µV·sec)	Height µV)	Area %
1	15.392	520787	5598	0.377
2	21.572	104127	3908	0.075
3	22.802	67558917	1410793	48.931
4	25.378	69600113	1328598	50.410
5	31.195	284685	6899	0.206
Total				100.000

HPLC for compound **30**

The purity of **30** was 99.95%. (Peaks 2 and 3 are enantiomers of compound **30**.)

HPLC conditions:

Column: CHIRALPAK® IF, Eluent: 10% EtOH in Hex, Flow rate: 0.5 mL/min, Temp: 25 ˚C,

Wavelength: 210 nm

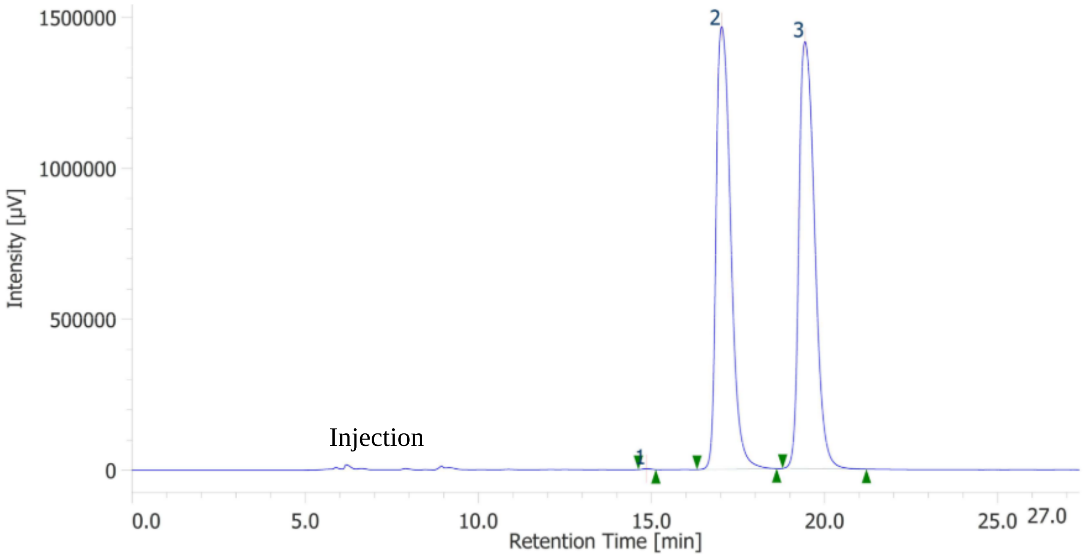

Peak No.	Retention Time (min)	Area (µV·sec)	Height (µV)	Area %
1	14.860	45377	2918	0.052
2	17.025	42870508	1465380	49.020
3	19.437	44538829	1414147	50.928
Total				100.000

HPLC for compound **31**

The purity of **31** was 99.49%. (Peaks 3 and 4 are enantiomers of compound **31**.)

HPLC conditions:

Column: CHIRALPAK® IG, Eluent: 50% EtOH in Hex, Flow rate: 0.5 mL/min, Temp: 25 °C,

Wavelength: 210 nm

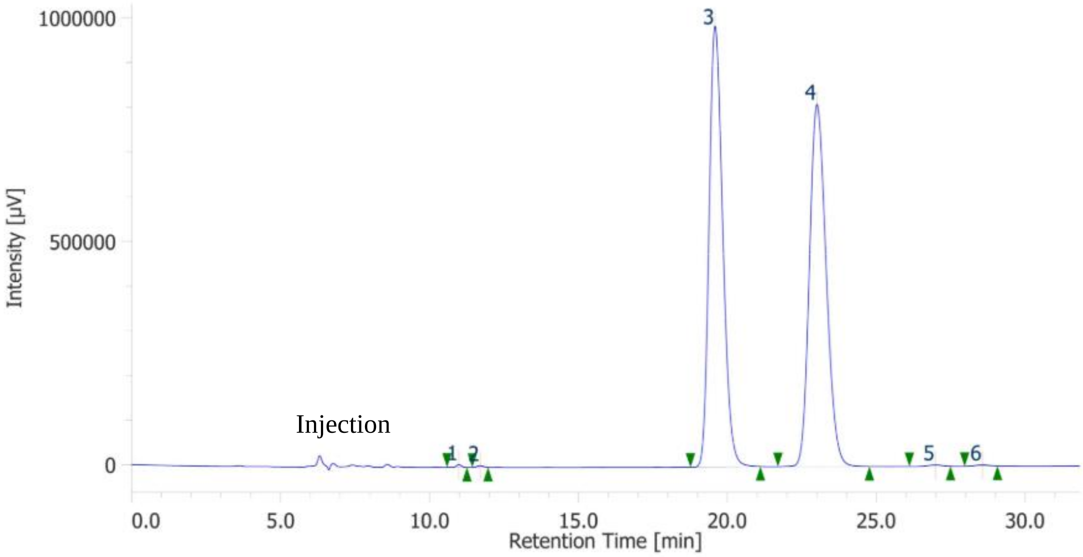

Peak No.	Retention Time (min)	Area (µV·sec)	Height (µV)	Area %
1	10.977	69876	5967	0.110
2	11.695	57424	3777	0.091
3	19.585	31328612	986813	49.475
4	23.007	31672568	810747	50.018
5	26.983	101295	3049	0.160
6	28.563	92146	2665	0.146
Total				100.000

HPLC for compound (+)-**31**

The purity of (+)-**31** was 98.59%.

HPLC conditions:

Column: CHIRALPAK® IA, Eluent: 10% EtOH in Hex, Flow rate: 0.5 mL/min, Temp: 25 °C,

Wavelength: 210 nm

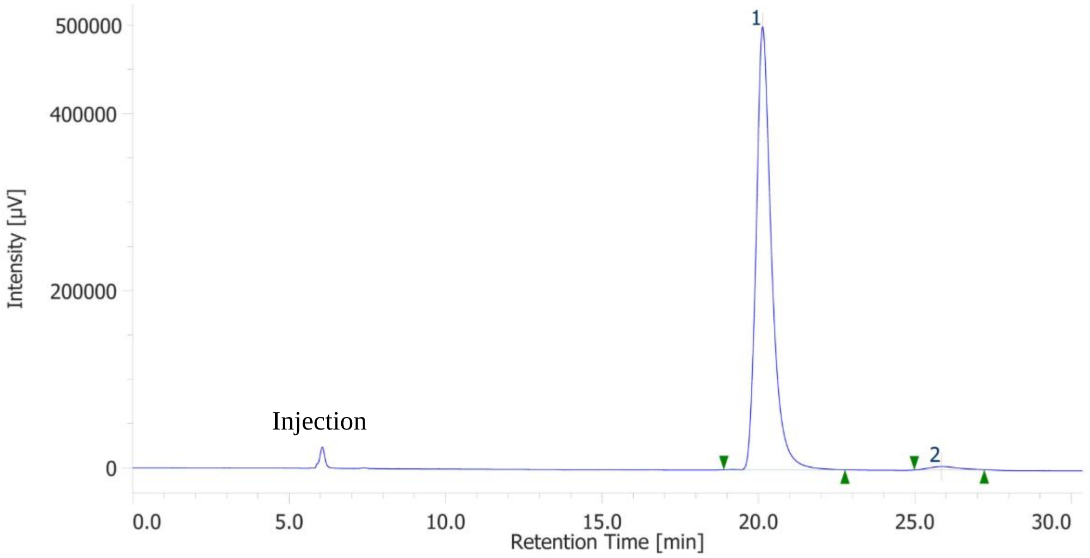

Peak No.	Retention Time (min)	Area (µV·sec)	Height (µV)	Area %
1	20.128	17110966	500412	98.586
2	25.847	245383	3797	1.414
Total				100.000

HPLC for compound (−)-**31**

The purity of (−)-**31** was 95.92%.

HPLC conditions:

Column: CHIRALPAK® IA, Eluent: 10% EtOH in Hex, Flow rate: 0.5 mL/min, Temp: 25 °C,

Wavelength: 210 nm

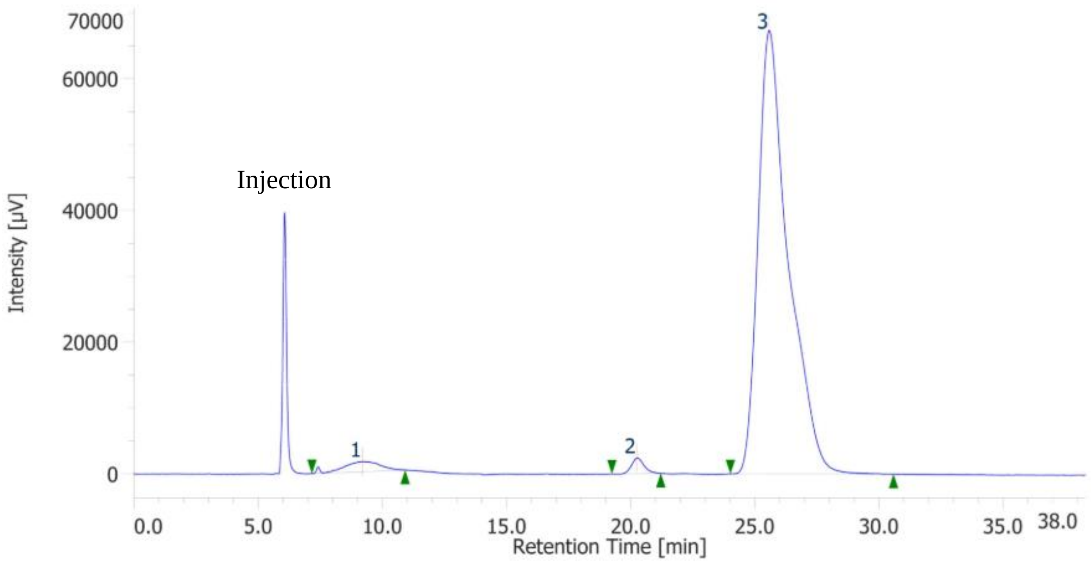

Peak No.	Retention Time (min)	Area (µV·sec)	Height (µV)	Area %
1	9.197	160282	1548	2.691
2	20.238	82823	2402	1.390
3	25.573	5714182	67338	95.919
Total				100.000

HPLC for compound **32**

The purity of **32** was 98.42%. (Peaks 4 and 6 are enantiomers of compound **32**.)

HPLC conditions:

Column: CHIRALPAK® IG, Eluent: 20% EtOH in Hex, Flow rate: 0.5 mL/min, Temp: 25 °C,

Wavelength: 210 nm

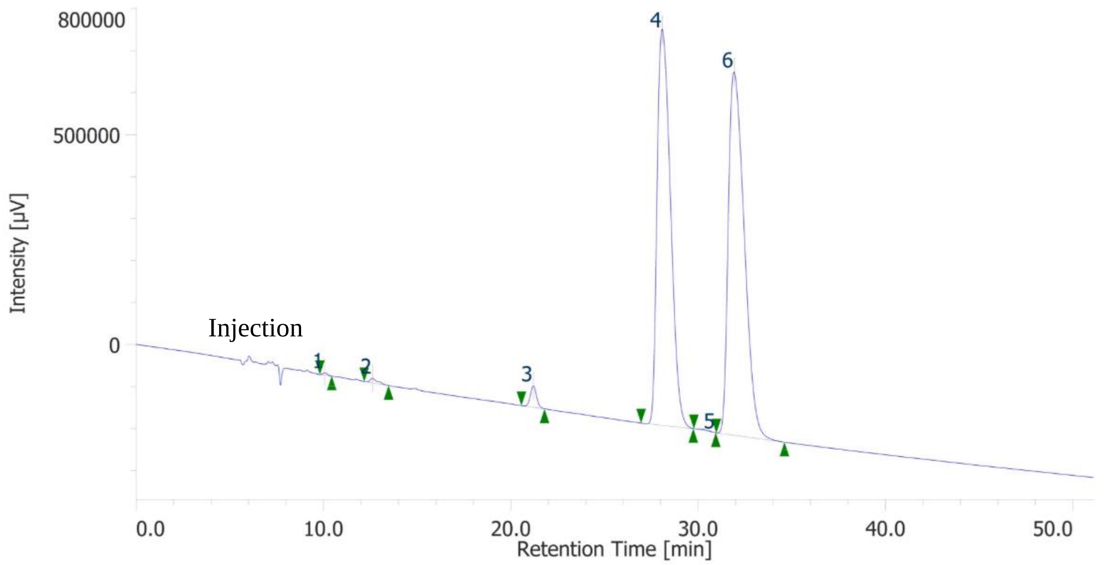

Peak No.	Retention Time (min)	Area (µV·sec)	Height (µV)	Area %
1	10.055	94183	5912	0.094
2	12.617	318486	11061	0.319
3	21.200	1165178	51180	1.168
4	28.075	46953197	943859	47.073
5	30.930	202	38	0.000
6	31.913	51213382	865945	51.345
Total				100.000

HPLC for compound (+)-**32**

The purity of (+)-**32** was 95.52%.

HPLC conditions:

Column: CHIRALPAK® IG, Eluent: 20% EtOH in Hex, Flow rate: 0.5 mL/min, Temp: 25 °C,

Wavelength: 210 nm

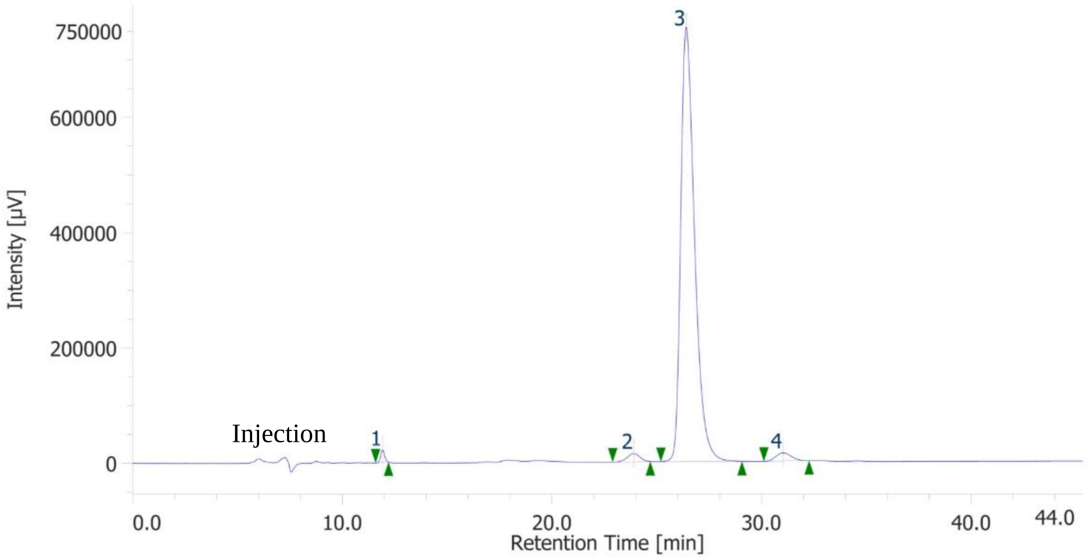

Peak No.	Retention Time (min)	Area (µV·sec)	Height (µV)	Area %
1	11.920	286772	21481	0.795
2	23.898	602474	14496	1.671
3	26.398	34450344	753281	95.522
4	31.015	725871	14279	2.013
Total				100.000

HPLC for compound (−)-**32**

The purity of (−)-**32** was 97.31%.

HPLC conditions:

Column: CHIRALPAK® IG, Eluent: 20% EtOH in Hex, Flow rate: 0.5 mL/min, Temp: 25 °C,

Wavelength: 210 nm

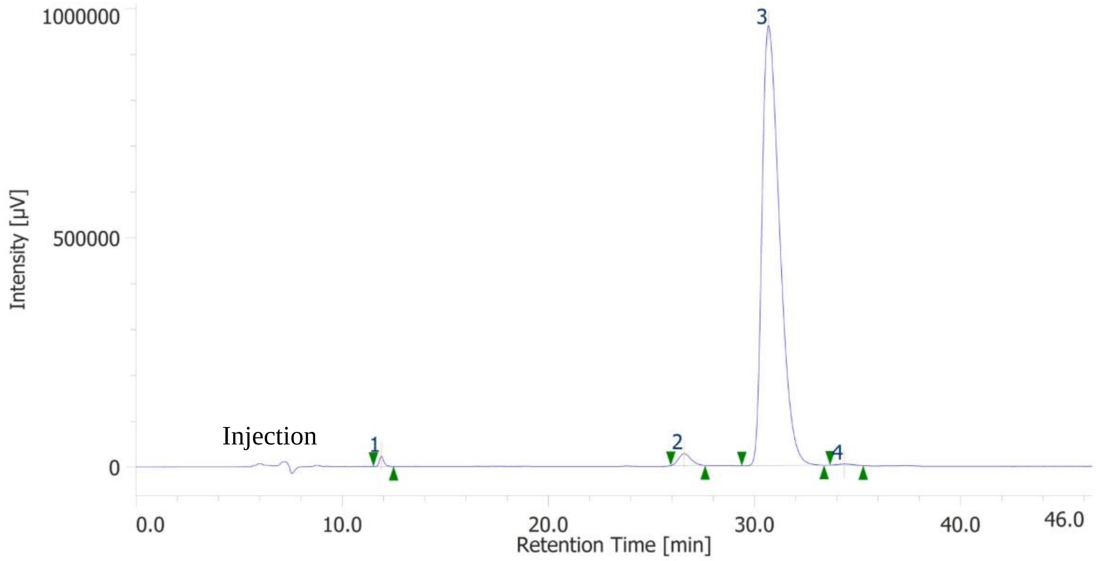

Peak No.	Retention Time (min)	Area (µV·sec)	Height (µV)	Area %
1	11.902	329081	22102	0.563
2	26.573	1094777	25806	1.873
3	30.678	56880810	959870	97.306
4	34.343	151133	2736	0.259
Total				100.000

HPLC for compound **33**

The purity of **33** was 96.27%. (Peaks 7 and 8 are enantiomers of compound **33**.)

HPLC conditions:

Column: CHIRALPAK® IG, Eluent: 20% EtOH in Hex, Flow rate: 0.5 mL/min, Temp: 25 °C,

Wavelength: 270 nm

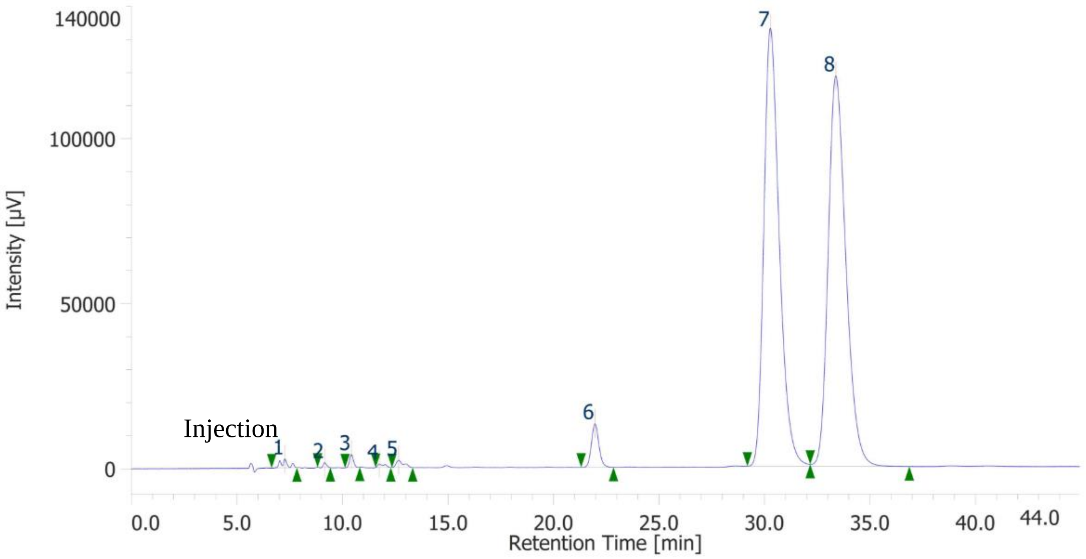

Peak No.	Retention Time (min)	Area (µV·sec)	Height (µV)	Area %
1	7.273	59682	2724	0.421
2	9.162	15475	1432	0.109
3	10.428	50997	3942	0.360
4	11.753	24888	1100	0.175
5	12.662	53527	2194	0.377
6	21.965	323842	13207	2.283
7	30.278	6747756	132730	47.573
8	33.382	6907793	118302	48.701
Total				100.000

HPLC for compound (+)-**33**

The purity of (+)-**33** was 98.74%.

HPLC conditions:

Column: CHIRALPAK® IG, Eluent: 20% EtOH in Hex, Flow rate: 0.5 mL/min, Temp: 25 °C,

Wavelength: 270 nm

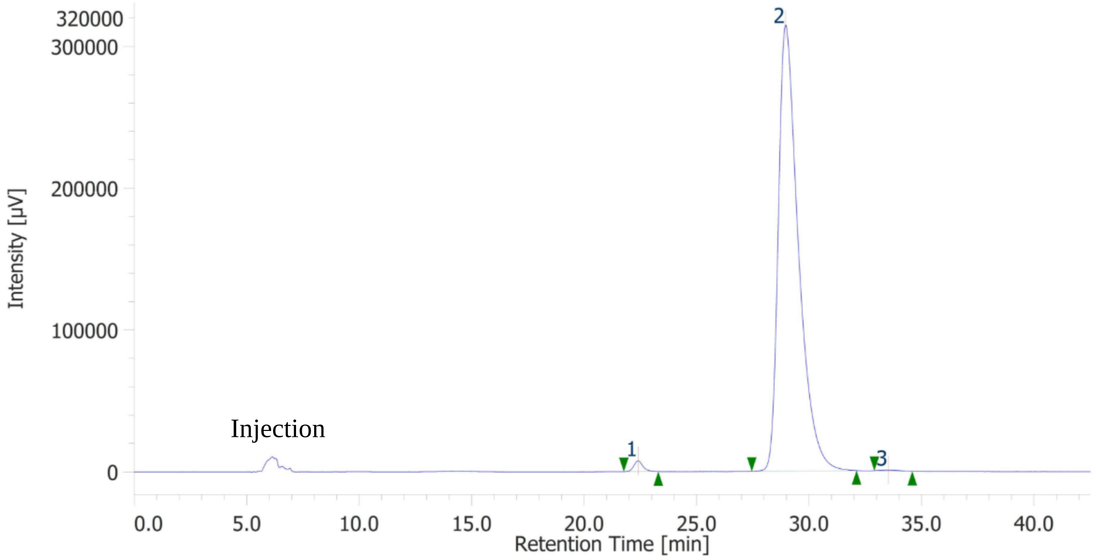

Peak No.	Retention Time (min)	Area (μV·sec)	Height (μV)	Area %
1	22.400	217905	7633	1.102
2	28.953	19529591	314583	98.744
3	33.525	30445	549	0.154
Total				100.000

HPLC for compound (–)-**33**

The purity of (–)-**33** was 97.38%.

HPLC conditions:

Column: CHIRALPAK® IG, Eluent: 20% EtOH in Hex, Flow rate: 0.5 mL/min, Temp: 25 °C,

Wavelength: 270 nm

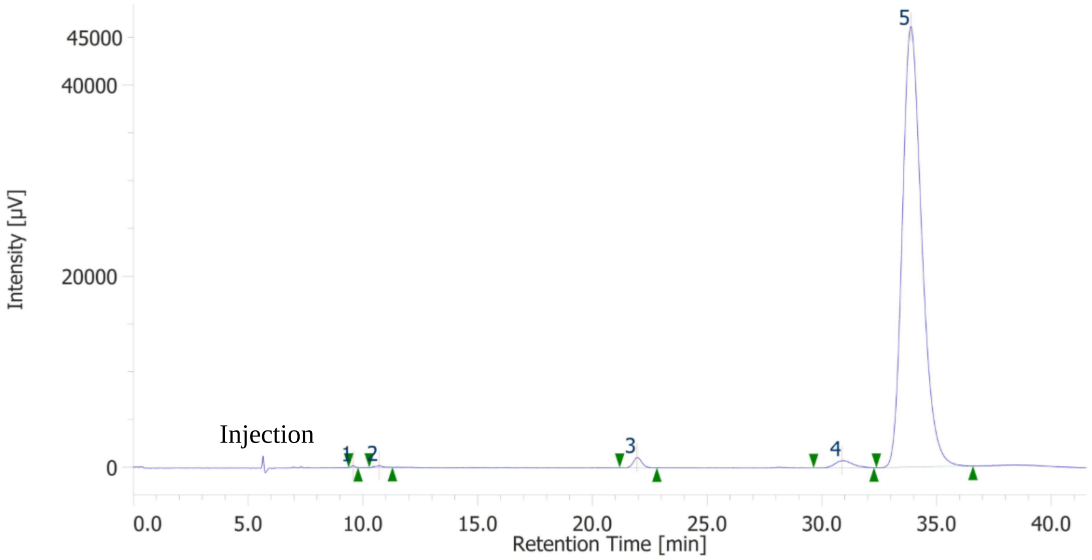

Peak No.	Retention Time (min)	Area (µV·sec)	Height (µV)	Area %
1	9.582	1928	195	0.069
2	10.707	3477	199	0.125
3	21.942	27910	1070	1.001
4	30.883	39786	751	1.427
5	33.875	2715253	46091	97.378
Total				100.000

HPLC for compound **35**

The purity of **35** was 99.57%. (Peaks 3 and 4 are enantiomers of compound **35**.)

HPLC conditions:

Column: CHIRALPAK® IG, Eluent: 30% EtOH in Hex, Flow rate: 0.5 mL/min, Temp: 25 °C,

Wavelength: 270 nm

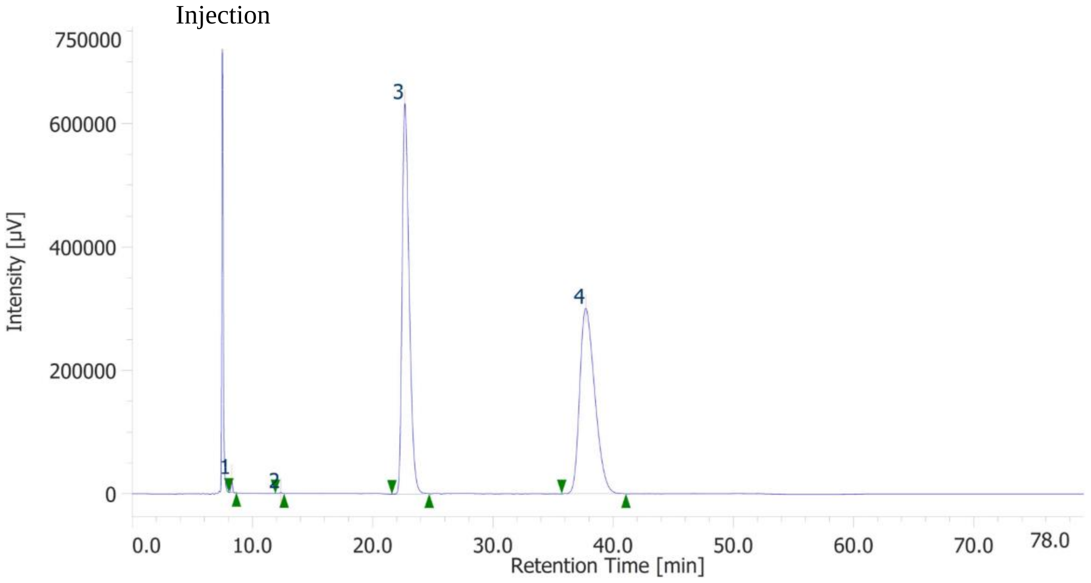

Peak No.	Retention Time (min)	Area (µV·sec)	Height (µV)	Area %
1	8.268	203045	22722	0.387
2	12.367	24660	1742	0.047
3	22.685	25909195	632614	49.438
4	37.718	26270045	300533	50.127
Total				100.000

HPLC for compound (+)-**35**

The purity of (+)-**35** was 95.45%.

HPLC conditions:

Column: CHIRALPAK® IG, Eluent: 30% EtOH in Hex, Flow rate: 0.5 mL/min, Temp: 25 °C,

Wavelength: 270 nm

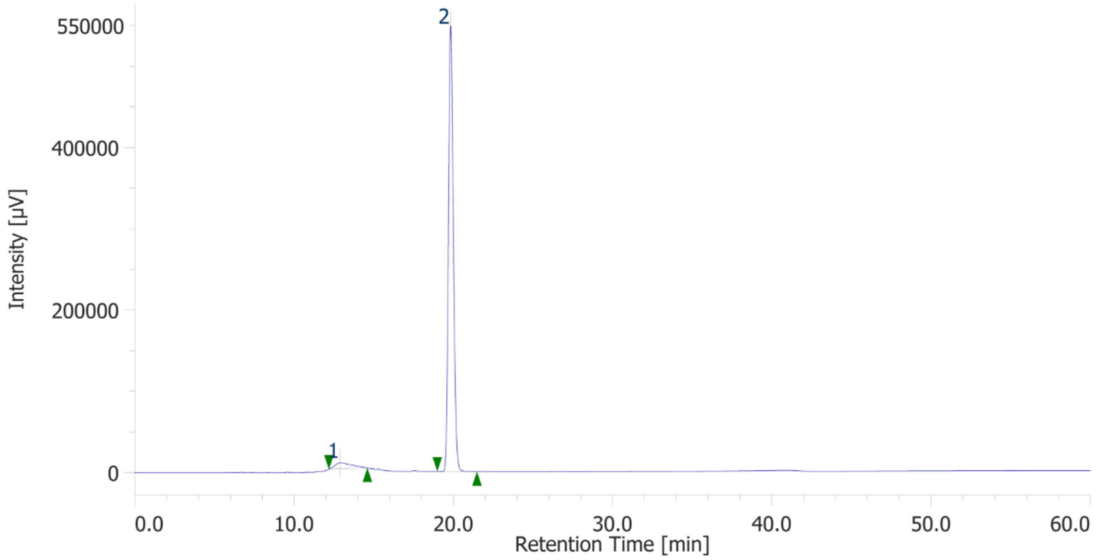

Peak No.	Retention Time (min)	Area (μV·sec)	Height (μV)	Area %
1	12.882	553512	7706	4.509
2	19.825	11723117	548467	95.491
Total				100.000

HPLC for compound (–)-**35**

The purity of (–)-**35** was 99.29%.

HPLC conditions:

Column: CHIRALPAK® IG, Eluent: 30% EtOH in Hex, Flow rate: 0.5 mL/min, Temp: 25 °C,

Wavelength: 210 nm

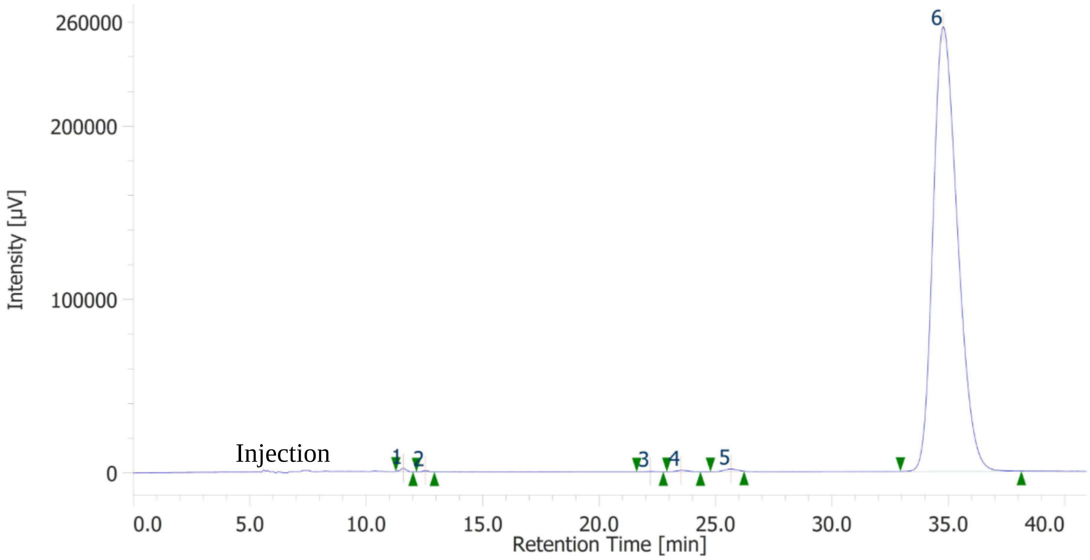

Peak No.	Retention Time (min)	Area (µV·sec)	Height (µV)	Area %
1	11.592	29999	1786	0.159
2	12.535	13554	741	0.072
3	22.198	9642	319	0.051
4	23.513	29216	803	0.155
5	25.663	51416	1383	0.273
6	34.778	18683450	256331	99.289
Total				100.000

HPLC for compound **36**

The purity of **36** was 95.86%. (Peaks 3 and 4 are enantiomers of compound **36**.)

HPLC conditions:

Column: CHIRALPAK® IG, Eluent: 30% EtOH in Hex, Flow rate: 0.5 mL/min, Temp: 25 °C,

Wavelength: 210 nm

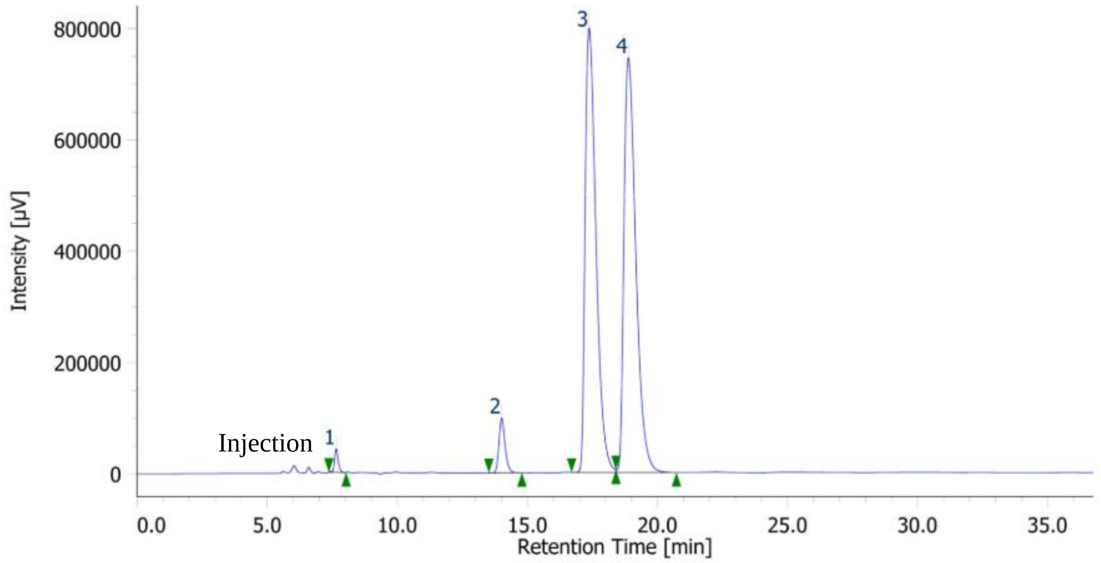

Peak No.	Retention Time (min)	Area (µV·sec)	Height (µV)	Area %
1	7.650	419383	42219	0.859
2	14.007	1604420	98608	3.285
3	17.368	23171533	798420	47.440
4	18.880	23648939	745146	48.417
Total				100.000

HPLC for compound **37**

The purity of **37** was 98.78%. (Peak 5 is enantiomers of compound **37**.)

HPLC conditions:

Column: CHIRALPAK® IG, eluent: 30% EtOH in Hex, flow rate: 0.5 mL/min, temp: 25 °C,

Wavelength: 270 nm

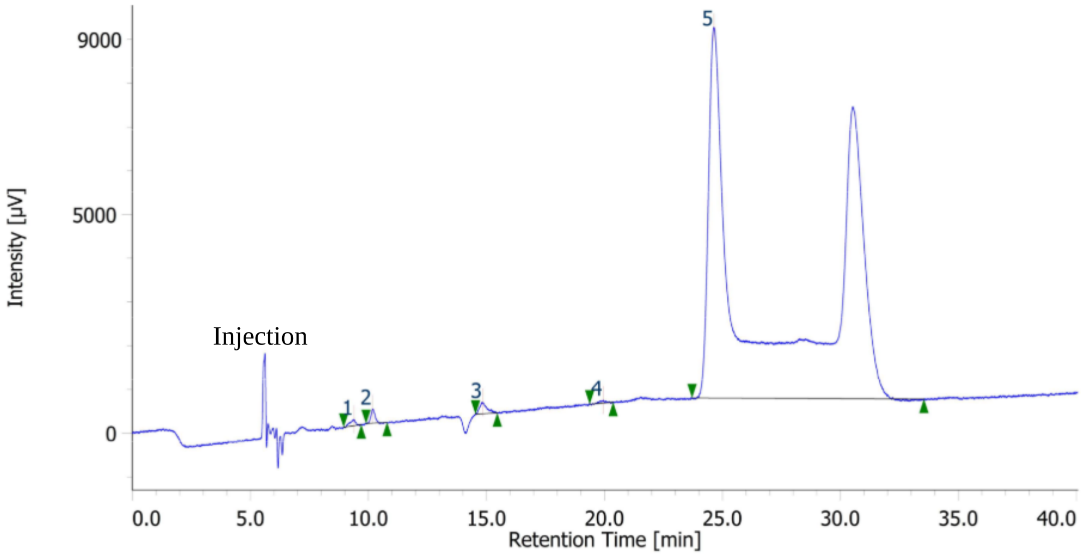

Peak No.	Retention Time (min)	Area (µV·sec)	Height (µV)	Area %
1	9.390	2362	148	0.222
2	10.172	3893	321	0.365
3	14.840	5651	258	0.530
4	19.945	1094	65	0.103
5	24.638	1052901	8470	98.780
Total				100.000

HPLC for compound **38**

The purity of **38** was 98.93%. (Peaks 2 and 3 are enantiomers of compound **38**.)

HPLC conditions:

Column: CHIRALPAK® IG, Eluent: 30% EtOH in Hex, Flow rate: 0.5 mL/min, Temp: 25 °C,

Wavelength: 270 nm

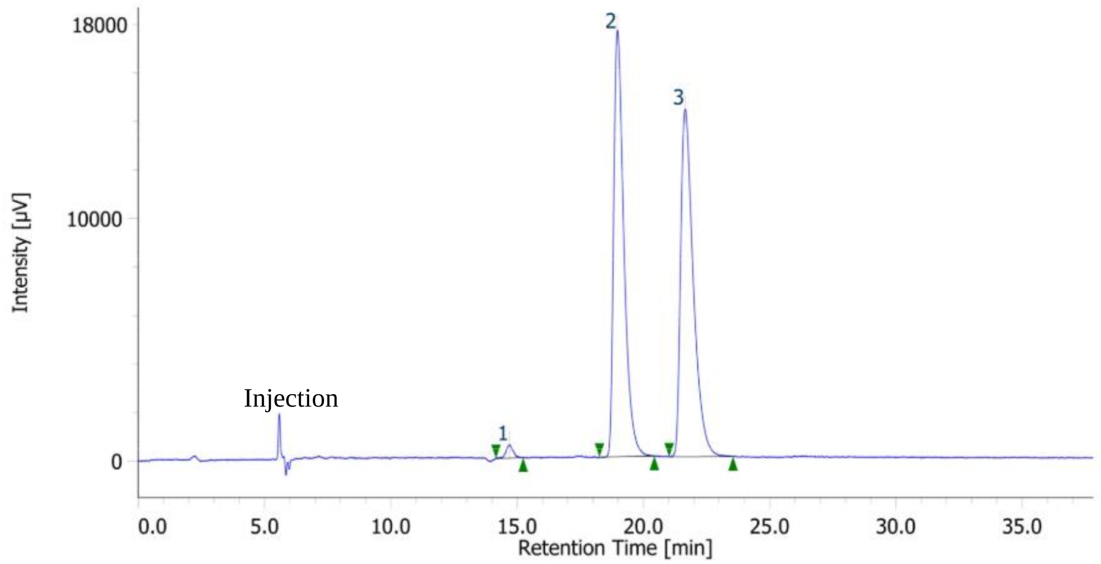

Peak No.	Retention Time (min)	Area (μV·sec)	Height (μV)	Area %
1	14.693	10791	559	1.071
2	18.968	496454	17597	49.266
3	21.652	500454	14332	49.663
Total				100.000
